# Second-generation probes for biosynthetic intermediate capture: towards a comprehensive profiling of polyketide assembly†
†Electronic supplementary information (ESI) available: General methods for the synthesis of chemical probes and LC-HRMS analysis of the biosynthetic intermediates isolated from *S. lasaliensis*. See DOI: 10.1039/c6cc04681a



**DOI:** 10.1039/c6cc04681a

**Published:** 2016-08-02

**Authors:** Ina Wilkening, Silvia Gazzola, Elena Riva, James S. Parascandolo, Lijiang Song, Manuela Tosin

**Affiliations:** a Department of Chemistry , University of Warwick , Library Road , CV4 7AL , UK . Email: M.Tosin@warwick.ac.uk ; Tel: +442476572878; b Dipartimento di Scienza ed Alta Tecnologia , Universita' dell'Insubria , Via Valleggio 11 , 22100 Como , Italy

## Abstract

Malonyl carba(dethia) *N*-decanoyl cysteamine methyl esters and novel acetoxymethyl esters were utilised as second-generation probes for polyketide intermediate capture. The use of these tools *in vivo* led to the characterisation of an almost complete set of biosynthetic intermediates from a modular assembly line, providing a first kinetic overview of intermediate processing leading to complex natural product formation.

## Abstract 2

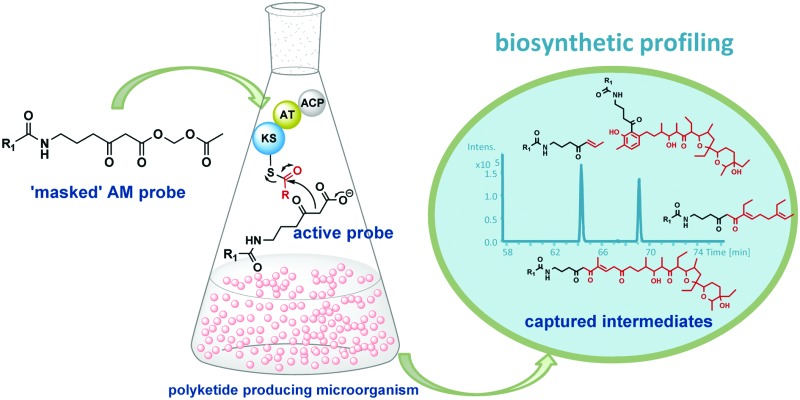
Novel chemical probes provide kinetic insights into polyketide assembly.

Polyketide natural products are ubiquitous in their distribution and remarkably varied in structure and biological function.^1^ Amongst them we encounter established potent antibiotics (*e.g.* fidaxomicin),^2,3^ anticancer agents (*e.g.* doxorubicin)^4^ and cholesterol-lowering agents (*e.g.* statins),^5^ as well as promising leads for the development of modulators of stem cell behaviour (*e.g.* salinomycin).^6^ Polyketide biosynthesis proceeds *via* the decarboxylative Claisen condensation of acyl carrier protein (ACP) bound-malonyl units with ketosynthase (KS)-bound acyl intermediates (Fig. 1, A): the resulting polyketide chain is subjected to variable ketoreduction, dehydration and enoyl reduction throughout chain extension, before being released from the polyketide synthase (PKS) enzyme (typically *via* thioester hydrolysis) and further modified by post-PKS enzymes (*e.g.* methyltransferases, glycosyltransferases, cytochrome P450s, *etc.*).^7–9^ The detailed elucidation of polyketide biosynthesis is of the utmost priority in view of enzyme engineering for novel synthetic biology aiming at polyketide production:^10–12^ indeed the knowledge of intermediate processing is crucial to improve the functioning of current pathways and to design *de novo* pathways leading to both high-value^13^ and commodity chemicals.^14^ Advances in molecular biology, synthetic chemistry and analytical techniques have allowed the probing of PKS pathways *via* reconstitution of enzyme activity *in vitro* ^15^ and *in vivo* genetic manipulation leading to the accumulation of intermediate or shunt products,^16^ for which the detection and the characterisation of small molecule intermediates/products by advanced MS and NMR have proved crucial.^17–19^ Nonetheless, many challenges concerning the ability to monitor polyketide biocatalysis stepwise, especially *in vivo* and in relation to whole assembly kinetics, remain. One of the major hurdles is constituted by the covalent attachment of biosynthetic intermediates to the biosynthetic PKS enzymes throughout PKS assembly. In recent years we have developed a general strategy for probing polyketide biocatalysis based on the use of chemical probes (Fig. 1): nonhydrolysable small-molecule mimics of malonate units recruited in polyketide formation compete in the natural decarboxylative Claisen condensation (A) to capture prematurely truncated biosynthetic intermediates (B) which would otherwise remain covalently bound to PKSs.^20–25^ This approach has proved successful for the isolation and characterisation of intermediate species from both modular^20–23^ and iterative polyketide synthases^24,25^ *in vitro* and *in vivo*, allowing the gathering of information on the timing and the mechanism of single catalytic events otherwise inaccessible. Lately we also demonstrated that this methodology is amenable for the generation of unnatural polyketide derivatives: for instance, novel polyether species were obtained in low titre from feeding experiments of mutant strains of *Streptomyces lasaliensis* (harbouring the modular PKS responsible the production of the polyether antibiotic lasalocid A) with functionalised chemical probes (Fig. 1).^23^ Moreover the use of the probes with strains harbouring 6-methylsalicylic acid synthase (a type I iterative PKS) has led to the generation of unnatural functionalised pentaketides.^25^ During our studies towards the improvement of polyketide capture, it occurred to us that probe bioavailability could be a crucial limiting factor: indeed the hydrolysis of the methyl ester probes **1–4** by cellular esterases has been estimated to be in the range of 5–70%, depending on the nature of the probe (Fig. 1) and of the polyketide producing-microorganism. Therefore we decided to investigate whether, by changing the probe ester protection, a higher concentration of the ‘active’ carboxylate species **5–8** could be systematically achieved *in vivo*, ultimately leading to improved intermediate capture and kinetic insights on polyketide assembly. In exploring possible protecting groups that would be easily hydrolysed *in vivo*, we considered the use of acetoxymethyl ester (AM) moieties. AM esters, notably introduced by Tsien for loading fluorescent indicators into cells,^26^ are widely employed in prodrugs^27^ and chemical probes^28^ in eukaryotic cells to mask hydrophilic/charged bioactive functionalities, allowing effective compound cellular uptake through membrane permeation. The AM functionality and structural variants of it have been used to protect, directly and indirectly, a wide variety of groups, including alcohols,^29^ amines,^30^ phosphates^31^ and carboxylic acids.^32,33^ To the best of our knowledge, it has not been yet utilised for the protection of β-keto carboxylic acid derivatives, and reports on the use of AM protected substrates in prokaryote cells are scarce. We first targeted the preparation of esters **15a–b** according to Scheme 1. Briefly, the carbonyl groups of the methyl esters **1a–b** were protected by conversion either to the ketals **13a–b** by reaction with 1-phenyl-1,2-ethanediol and chlorotrimethylsilane in reflux conditions (route 1),^34^ or to thioketals **17a** by reaction with 1,2-ethanediol and boron trifluoride diethyl etherate (route 2). **13a–b** and **16a** were then hydrolysed to the corresponding carboxylates by treatment with potassium trimethyl silanolate,^35^ and converted to the acetoxymethyl esters **14a–b** and **17a** by reaction with bromomethyl acetate in dry THF. Finally, the desired compounds **15a–b** were obtained by hydrogenation of **14a–b** over Pd(OAc)_2_ and Pd(OTf)_2_ catalysts in dry ethyl acetate (route 1),^36^ or by treatment of **17a** with [bis-(trifluoroacetoxy)iodo]benzene (route 2). AM esters are known to spontaneously hydrolyse over time, even in neutral conditions. Therefore **15a–b** were promptly purified by HPLC and stored for a limited amount of time at low temperature and in a lyophilised form prior to their use. *S. lasaliensis* ACP12 (S970A), for which late-stage intermediates of lasalocid A have been extensively characterised,^22,23^ was chosen as our *in vivo* model system to evaluate the efficiency of the ‘second-generation’ AM ester probes **15a–b** in comparison to the first-generation methyl esters **1a–b**. LC-HRMS analyses of the ethyl acetate extracts of *S. lasaliensis* ACP12 (S970A) grown with gradual addition of probes **15a–b** (0.4–0.8 mM) over 5 days revealed their quantitative hydrolysis to **5a–b** and the presence of significant higher amounts of the unnatural polyethers **9a–b** (Fig. 2). Several careful repetitions of these experiments and LC-MS analyses optimisation have allowed us to estimate that up to ten times more of **9a** was generated using the AM probes **15a** in comparison to its methyl ester counterparts **1a**. Whereas the AM probes **15a–b** led to increased amounts of **9a–b**, no additional polyketide species could be detected in the organic extracts. In a variety of previous *in vivo* experiments, we noticed that *N*-decanoyl probes methyl ester probes such as **3** (Fig. 1) seemed capable of off-loading relatively short intermediates from both modular and iterative PKSs,^23–25^ possibly due to increased hydrophobicityof the probe or a better mimicking of the phosphopantetheine cofactor. Therefore we decided to pursue the preparation of *N*-decanoyl AM ester probe **20** following route 2 of Scheme 1. When both **3** and **20** were utilised in feeding experiments, further enhancement in the titre of captured intermediates was clearly observed in comparison to those deriving from short chain probes **1** and **15**: this seemed to mirror the higher concentrations of active probe **7** generated *in vivo*, especially from the AM precursor **20** (∼95%, Fig. S26, ESI†). More crucially, biosynthetic intermediates of different chain length and degree of processing were off-loaded from all the lasA modules with the exception of module 10 (Fig. 3 and ESI†). As well as expected species which were previously undetected, we also observed a number of unexpected overly processed dodecaketides (Fig. 3 and ESI†). These were unequivocally identified by the *m*/*z* 377 fragment (typical of lasalocid derivatives) obtained by high resolution MS^2^ (Fig. S47 and S48). Similar intermediates were obtained and confirmed from the use of the azido probe **8** and further intermediate functionalisation by Staudinger-phosphite reaction as previously reported^23^ (ESI†). The mechanism and the timing of transformations leading to lasalocid A assembly have been previously investigated by us and by others.^22,37^ Also, spontaneous offloading of PKS biosynthetic intermediates has been occasionally reported from the fermentation of wild-type and engineered bacterial strains.^38,39^ Herein, we have been able to obtain for the first time a comprehensive and stepwise picture of PKS modular assembly as a result of an improved chemical chain termination strategy. Diketides, pentaketides and dodecaketides were the most frequently and abundantly observed species for *S. lasaliensis* ACP12 (S970A) (highlighted in Fig. 3, see also ESI†), whereas the capture of intermediates from modules 9 to 11 proved particularly challenging. In this context the use of the AM ester probe **20** proved more advantageous than the corresponding methyl ester **3** in that it lead to increased amounts of captured species aiding their identification and characterisation. The accumulation of intermediates in higher amounts from specific modules of the lasA PKS possibly arises from: (1) slower enzymatic steps taking place within particular modules and within the whole PKS, including different extent of KS site priming and inter-domain intermediate translocation, and/or (2) different active site accessibility and capability of diffusion for the probe and the off-loaded intermediates. Despite the abundance of kinetic data available for modular enzymatic constructs *in vitro*,^40–42^ the rate-determining steps of whole complex PKS biosynthetic pathways remain unclear. In our experiments, we observed condensation products of the chemical probes with KS-bound intermediates in most cases, however the abundance of these species varies across modules, suggesting that either the different Claisen condensation steps proceed at different rates, or that KS-priming varies within a whole PKS as a result of intermediate chain transfers across modules. In addition, the intermediates captured from different modules present different degree and extent of processing, which likely result from different rates of the individual KR-, DH- and ER-catalysed steps and/or from differential substrate accessibility to their active sites. On the basis of the data herein gathered, which show the detection of diketide, pentaketide and dodecaketide species in higher frequency and abundance, we propose that intermediate processing between modules 1 and 2, 4 and 5, and 11 and 12 (including intermediate aromatisation) is slower compared to that occurring at other stages of PKS assembly: this is related either to the rapidity of intra- or inter-modular processing, or likely to a combination of both. For modular PKSs a ‘turnstile’^42^ or ‘retardation’^43^ mechanism control has been proposed, for which any ketosynthase of a modular PKS is not primed until completion of intermediate processing and transfer of the module product to the downstream KS: this would account for the unidirectionality of intermediate processing in co-linear pathways and may be controlled by the release of carbon dioxide following decarboxylative Claisen condensation.^42^ Also, a number of structural models for selected PKS modules have recently become available on the basis of cryo-electron microscopy^44,45^ and small-angle-X-ray-scattering analyses:^46,47^ these preliminary results show a single reaction chamber into which most of the domain active sites face. The unexpected presence of overly processed dodecaketides, formally deriving from ketoredution and dehydration reactions of an expected dodecaketide yet to be aromatised (see Fig. 3 and ESI†), suggests that dodecaketide diffusion into the adjacent module 11 bearing active KR and the DH domains may have taken place. Whereas substrate diffusion across adjacent domains has been documented *in vitro*,^41^ free substrate diffusion to adjacent modules *in vivo* has yet to be reported and is currently under investigation.

**Fig. 1:**
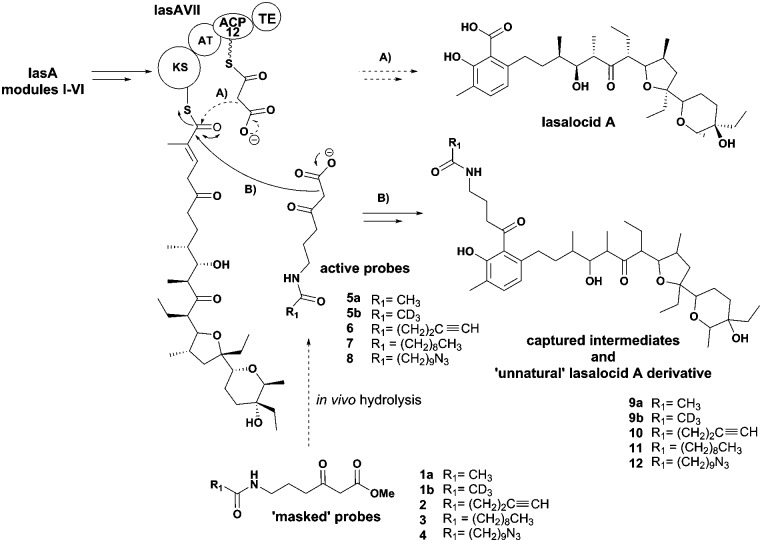
Polyketide intermediate capture by chemical probes **5–8**: these interfere with the normal biosynthetic pathway (A) and undergo competitive decarboxylative Claisen condensation with KS-bound intermediates to off-load them (B). Advanced captured species constitute also novel unnatural product derivatives (*e.g.* **9–12**).^23^ las A = lasalocid A polyketide synthase; KS = ketosynthase; AT = acyltransferase; ACP = acyl carrier protein; TE = thioesterase.

**Scheme 1:**
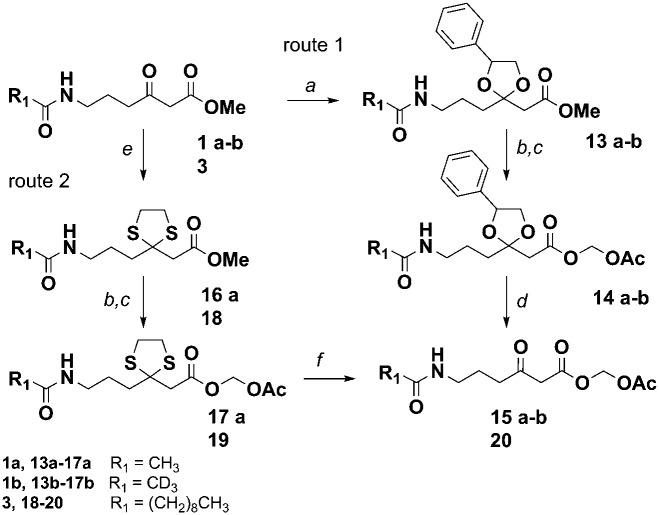
Synthesis of novel acetoxymethyl ester probes **15a–b** and **20**. Reagents and conditions: (a) PhCH(OH)CH_2_OH, TMSCl, CH_2_Cl_2_, reflux, 90%; (b) KOTMS, THF, 3 h, 86–100%; (c) BrCH_2_OAc, THF, 40–98%; (d) Pd(OAc)_2_, Pd(OTf)_2_, H_2_(g), EtOAc, 48 h, 40% (after HPLC purification); (e) HS(CH_2_)_2_SH, BF_3_·Et_2_O, CH_2_Cl_2_, reflux, 61–90%; (f) (CF_3_CO_2_)_2_IC_6_H_5_, CH_3_CN, H_2_O, 78–82% (after HPLC purification).

**Fig. 2:**
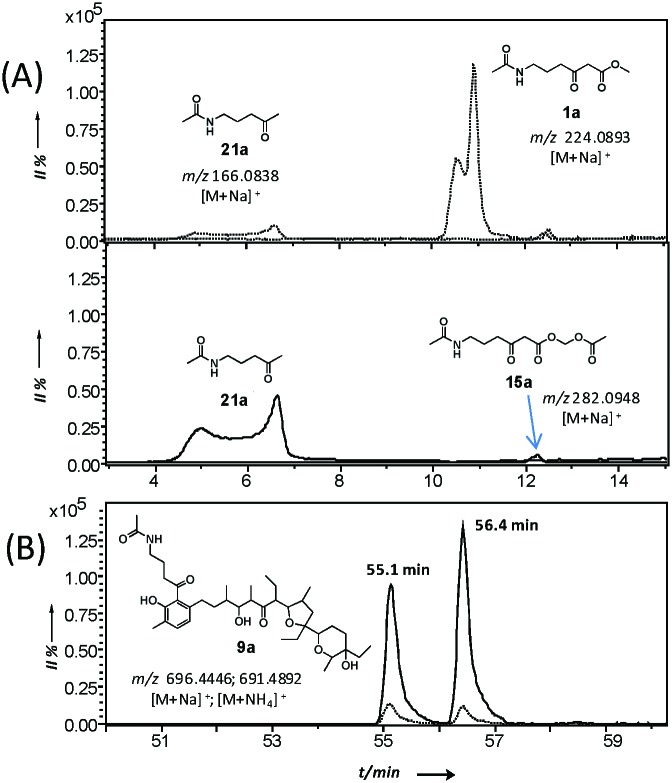
Comparative micro LC-HRMS analysis (Bruker MaXis UHR-ESI-TOF) of the organic extracts of *S. lasaliensis* ACP12 (S970A) grown in the presence of **1a** and **15a**. (A) [M + Na]^+^ extracted ion traces for probes **1a** (top) and **15a** (bottom), and their hydrolysis/decarboxylation product **21a** after 5 days of fermentation; (B) detection of species **9a** (sum of [M + Na]^+^ and [M + NH_4_]^+^ adducts): the amount captured by **15a** (continuous line) is approximately one order of magnitude higher than that obtained by **1a** (dashed line). The detection of a double peak for **9a** is under investigation.

**Fig. 3:**
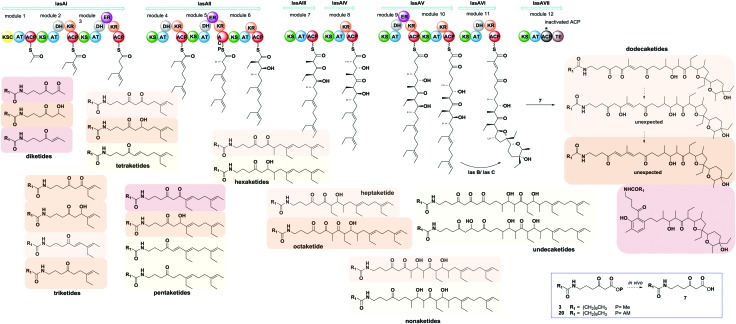
Overview of intermediates captured from *S. lasaliensis* ACP12 (S970A) *via* ester probes **3** and **20**. Coloured boxes have been used to differentiate the different species on the basis of their relative abundance (red boxes for the most abundant/recurrent species, light yellow for the least abundant/recurrent).

In conclusion, we developed novel chemical probes of enhanced bioavailability that allowed the gathering of preliminary kinetic insights into complex natural product assembly *in vivo*: these hold intriguing implications for the development of novel synthetic biology aiming at novel polyketide production.^10–12,48–50^

We gratefully acknowledge BBSRC (project grant BB/J007250/1 to M. T.), FP7 (Marie Curie Intraeuropean Fellowship to I. W.); Università degli Studi dell'Insubria (PhD studentship to S. G.); EPSRC (DTA PhD studentship to J. S. P.); the Institute of Advanced Studies (IAS) at Warwick (Postdoctoral Fellowship to E. R.); Dr Sue Slade and Dr Cleidiane Zampronio (School of Life Sciences, Warwick) for assistance with LC-HRMS^n^ analyses performed on an Orbitrap Fusion instrument; Prof Greg Challis (University of Warwick) for the use of a MaXis Bruker Impact instrument; and Prof. Peter F. Leadlay (University of Cambridge) for the kind gift of *S. lasaliensis* ACP12 (S970A).
