# Supplementary material for: Second-generation probes for biosynthetic intermediate capture: towards a comprehensive profiling of polyketide assembly
Source: Chem Commun (Camb). 2016 Aug 2;52(68):10392–5. doi: 10.1039/c6cc04681a (PMC5050551; doi:10.1039/c6cc04681a)

Electronic Supplementary Material (ESI) for ChemComm.
This journal is © The Royal Society of Chemistry 2016

# Supporting Information

## Second-generation probes for biosynthetic intermediate capture: towards a comprehensive profiling of polyketide assembly

Ina Wilkening,* Silvia Gazzola,* Elena Riva, James S. Parascandolo, Lijiang Song and Manuela Tosin**

Department of Chemistry

University of Warwick

Library Road, Coventry CV4 7AL, UK

**M.Tosin@warwick.ac.uk**

Ina Wilkening,* Silvia Gazzola,* Elena Riva, James S. Parascandolo, Lijiang Song and Manuela Tosin**

# Table of Content

*1. Synthesis of novel chemical probes ____ 3*

1.1. General methods ____ 3

1.2. Synthesis of probes 15a-b ____ 4

1.2.1. Methyl 2-(2-(3-acetamidopropyl)-4-phenyl-1,3-dioxolan-2-yl)acetate (**13a**) ____ 5

1.2.2. Methyl 2-(2-(3-$d_3$-acetamidopropyl)-4-phenyl-1,3-dioxolan-2-yl)acetate (**13b**) ____ 5

1.2.3. Acetoxymethyl 2-(2-(3-acetamidopropyl)-4-phenyl-1,3-dioxolan-2-yl)acetate (**14a**) ____ 6

1.2.4. Acetoxymethyl 2-(2-($d_3$-acetamidopropyl)-4-phenyl-1,3-dioxolan-2-yl)acetate (**14b**) ____ 7

1.2.5. Methyl 2-[2-(3-acetamidopropyl)-1,3-dithiolan-2-yl]acetate (**16a**) ____ 8

1.2.6. Acetoxymethyl 2-[2-(3-acetamidopropyl)-1,3-dithiolan-2-yl]acetate (**17a**) ____ 9

1.2.7. Acetoxymethyl 6-acetamido-3-oxohexanoate (**15a**) ____ 9

1.2.8. Acetoxymethyl 6-$d_3$-acetamido-3-oxohexanoate (**15b**) ____ 10

1.3. Synthesis of *N*-(4,6-dioxoheptyl)decanamide (20) ____ 10

1.3.9. Methyl-2-(2-(3-decanamidopropyl)-1,3-dithiolan-2-yl)acetate (**18**) ____ 11

1.3.10. Potassium 2-(2-(3-decanamidopropyl)-1,3-dithiolan-2-yl)acetate (**26**) ____ 12

1.3.11. Acetoxymethyl-2-(2-(3-decanamidopropyl)-1,3-dithiolan-2-yl)acetate (**19**) ____ 12

1.3.12. Acetoxymethyl-6-decanamido-3-oxohexanoate (**20**) ____ 13

1.3.13. Synthesis of methyl 6-(10-azidodecanamido)-3-oxohexanoate (**4**) ____ 13

*2. Growth of S. lasaliensis ACP12 (S970A) and mass spectrometry analysis ____ 14*

2.1. Summary of captured intermediates for *S. lasaliensis* ACP12(S970A) ____ 16

2.2. Intermediates captured by methyl 6-decanamido-3-oxohexanoate (3) ____ 19

2.3. Intermediate capture by *N*-(4,6-dioxoheptyl)decanamide (20) ____ 42

2.4. Intermediate capture by methyl 6-(10-azidodecanamido)-3-oxohexanoate (4) ____ 65

2.5. Staudinger-phosphite derivatisation of azido intermediates ____ 80

*3. Spectra ____ 93*

3.1. $^1$H- and $^{13}$C-NMR of compound 13a (400 MHz, $CDCl_3$) ____ 93

3.2. $^1$H- and $^{13}$C-NMR of compound 13b (400 MHz, $CDCl_3$) ____ 94

3.3. $^1$H- and $^{13}$C-NMR of compound 14a (400 MHz, $CDCl_3$) ____ 95

3.4. $^1$H- and $^{13}$C-NMR of compound 14b (400 MHz, $CDCl_3$) ____ 96

3.5. $^1$H- and $^{13}$C-NMR of compound 16a (400 MHz, $CDCl_3$) ____ 97

**3.6. $^{1}$H- and $^{13}$C-NMR of compound 17a (400 MHz, $CDCl_3$) ____ 98**

**3.7. $^{1}$H- and $^{13}$C-NMR of compound 15a (400 MHz, $CDCl_3$) ____ 99**

**3.8. $^{1}$H- and $^{13}$C-NMR of compound 15b (400 MHz, $CDCl_3$) ____ 100**

**3.9. $^{1}$H- and $^{13}$C-NMR of compound 18 (400 MHz, $CDCl_3$) ____ 101**

**3.10. $^{1}$H- and $^{13}$C-NMR of compound 24 (400 MHz, $D_2O$) ____ 102**

**3.11. $^{1}$H- and $^{13}$C-NMR of compound 19 (400 MHz, $CDCl_3$) ____ 103**

**3.12 $^{1}$H- and $^{13}$C-NMR of compound 20 ____ 104**

# 1. Synthesis of novel chemical probes

## 1.1. General methods

**Solvents and reagents:** Unless specified otherwise, chemicals were purchased from Sigma Aldrich, Fisher Scientific, Carbosynth or Alfa Aesar and were used without further purification. Anhydrous dichloromethane and tetrahydrofurane were purchased from VWR International (AR grade) and dried using solvent towers. Anhydrous ethyl acetate and methanol were purchased from Fisher Scientific or Sigma Aldrich. Reagent grade dichloromethane, ethyl acetate, methanol, acetonitrile, cyclohexane and chloroform were purchased from Fisher Scientific.

**Chromatography:** Analytical thin-layer chromatography (TLC) was performed on aluminium sheets precoated with silica gel 60 (F254, Merck) and visualized under ultra-violet light (short and long-wave) and using potassium permanganate ($KMnO_4$) or vanillin stains. Silica gel was purchased from Sigma Aldrich (Tech Grade, pore size 60 Å, 230-400 mesh).

**NMR Spectroscopy:** $^1$H and $^{13}$C NMR spectra were recorded in $d^4$-MeOD, $CDCl_3$ or $D_2O$ on the following Bruker Avance instruments: DPX-400 400 MHz, DRX-500 500 MHz, AV III-500 HD 500 MHz or AV-600 600 MHz.

**LC-HRMS:** High-resolution mass spectra (HRMS) of newly made compounds were obtained using electrospray ionization (ESI) on a MaXis UHR-TOF (Bruker Daltonics) or on Bruker MaXis (ESI-HR-MS).

**HPLC:** Compounds were purified by semipreparative HPLC on a Phenomenex synergi™ Polar RP 80 Å (250 x 10.0 mm, 4 µm) column. The mobile phase consisted of a gradient of water and acetonitrile (HPLC grade, containing 0.1% (v/v) trifluoroacetic acid) at a flow rate of 2.5 mL/min, with UV detection at 210, 254 and 280 nm.

Ina Wilkening,* Silvia Gazzola,* Elena Riva, James S. Parascandolo, Lijiang Song and Manuela Tosin**

## 1.2. Synthesis of probes 15a-b

### Route 1

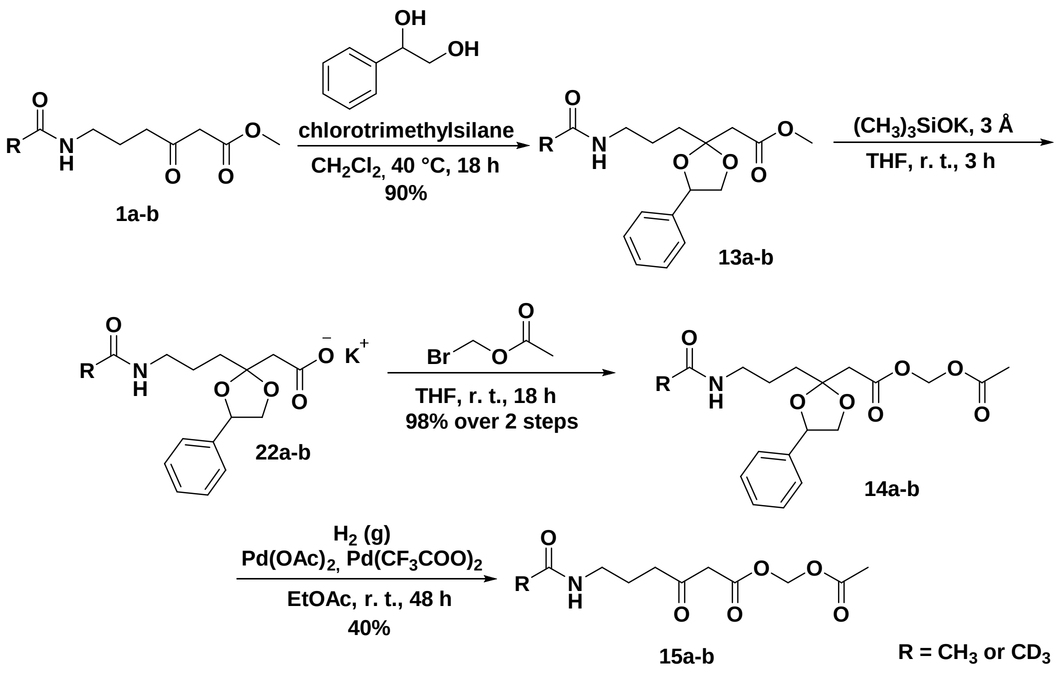


### Route 2

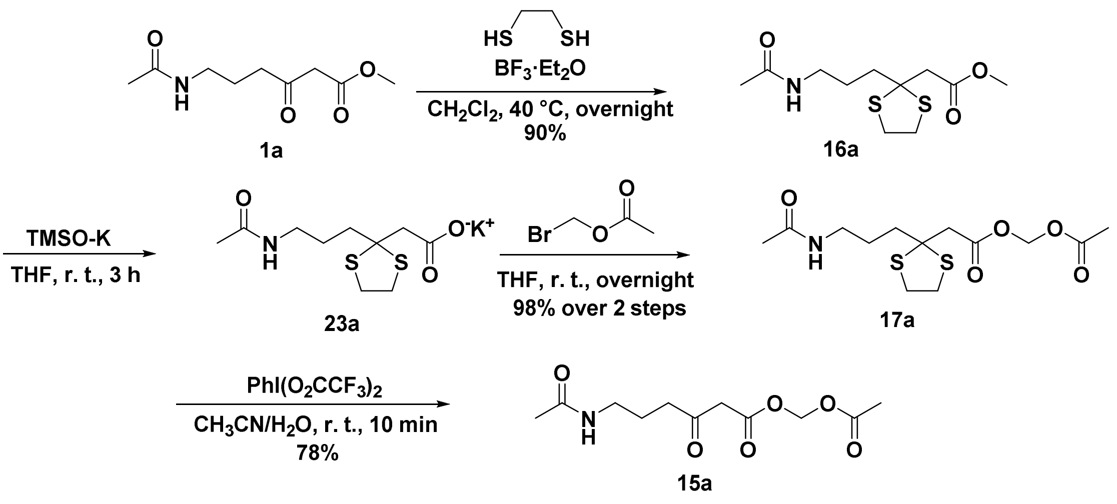

### 1.2.1. Methyl 2-(2-(3-acetamidopropyl)-4-phenyl-1,3-dioxolan-2-yl)acetate (13a)

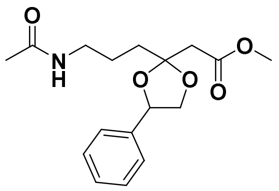

To a solution of the methyl ester **1a**[1] (1.40 g, 7.00 mmol) in dry dichloromethane (40 mL), 1-phenyl-1,2-ethanediol (1.92 g, 13.9 mmol) and chlorotrimethylsilane (3.51 mL, 27.9 mmol) were added dropwise under argon atmosphere. The reaction mixture was heated under reflux at 40 °C for 18 h.[2] The solvent was removed *in vacuo*, the resulting residue was dissolved in EtOAc (40 mL) and washed with 5% (w/v) $NaHCO_3$ (20 mL). The organic layer was dried over $MgSO_4$ and concentrated affording **13a** as a yellow oil (2.01 g, 90%; $R_f$ = 0.60 in 9:1 $CH_2Cl_2$:MeOH), which was used directly in the next step. A sample of this crude material was purified by semipreparative HPLC for accurate NMR characterisation ($R_t$ = 23 min, with a gradient elution from 0 to 100% MeCN over 30 minutes). A double set of signals was observed for two major isomers present in 1:1 ratio. **$^1$H-NMR** (400 MHz, $CDCl_3$) δ = 1.68-1.79 (4H, m, $CH_2CH_2CH_2$), 1.95 and 1.97 (each 3H, s, $CH_3$CONH), 1.92-2.04 (4H, m, $CH_2CO_2$), 2.80 and 2.80 (each 2H, d, *J* = 15.0 Hz, $CH_2$CO), 3.27-3.33 (4H, m, $CH_2$NH), 3.68-3.73 (2H, m, $CH_2$O), 3.71 (6H, s, $OCH_3$), 4.31-4.37 (2H, m, $CH_2$O), 5.05 (1H, dd, *J* = 6.0, 9.3 Hz, CH), 5.15 (1H, dd, *J* = 6.5, 8.4 Hz, CH), 5.95 and 5.98 (each 1H, br s, NH), 7.29-7.37 (10H, m, ArH); **$^{13}$C-NMR** (125 MHz, $CDCl_3$) δ = 23.1 ($CH_3$CO), 23.1 ($CH_3$CO), 23.5 ($CH_2CH_2CH_2$), 23.6 ($CH_2CH_2CH_2$), 35.1 ($CH_2CO_2$), 35.2 ($CH_2CO_2$), 39.4 ($CH_2$NH), 39.5 ($CH_2$NH), 42.4 ($CH_2$CO), 43.1 ($CH_2$CO), 51.8 ($CH_3$O), 51.8 ($CH_3$O), 71.9 ($CH_2$O), 71.9 ($CH_2$O), 78.0 (CHO), 78.9 (CHO), 109.8 ($CO_2$), 109.9 ($CO_2$), 126.1 (CH arom), 126.3 (CH arom), 128.2 (CH arom), 128.3 (CH arom), 128.5 (CH arom), 128.6 (CH arom), 137.5 (C arom), 137.7 (C arom), 169.7 (CONH), 169.8 (CONH), 170.3 ($CO_2$Me), 170.3 ($CO_2$Me); ***m/z* (HR-ESI-MS)**: found $[M+Na]^+$ 344.1470, $C_{17}H_{23}NO_5Na^+$ requires 344.1468.

### 1.2.2. Methyl 2-(2-(3-d$_3$-acetamidopropyl)-4-phenyl-1,3-dioxolan-2-yl)acetate (13b):

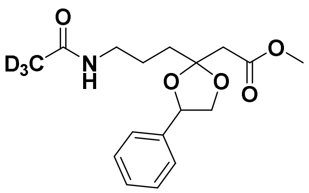

---

[1] (a) Tosin, M.; Betancor, L.; Stephens, E.; Li, W. M. A.; Spencer, J. B.; Leadlay, P. F. *ChemBioChem,* **2010**, *11*, 539-546; (b) Tosin, M.; Demdychuk, Y.; Parascandolo, J. S.; Blasco Per, C.; Leeper, F. J.; Leadlay, P. F. *Chem. Commun.,* **2011**, *47*, 3460-3462.

[2] Chan, T. H.; Brook, M. A.; Chaly, T. *Synthesis,* **1983**, *3*, 203-205.

Compound **13b** was prepared in the same way as **13a** from methyl 6-($d_3$-acetamido)-3-oxohexanoate **1b**.[1b]
**$^1$H-NMR** (400 MHz, $CDCl_3$) δ = 1.66-1.80 (4H, m, $CH_2CH_2CH_2$), 1.90-2.05 (4H, m, $CH_2$), 2.80 and 2.81 (each 2H, d, *J* = 15.0 Hz, $CH_2CO$), 3.27-3.34 (4H, m, $CH_2NH$), 3.71 (6H, s, $OCH_3$), 3.71 (2H, t, *J* =8.5 Hz, $CH_2O$), 4.32-4.38 (2H, m, $CH_2O$), 5.06 (1H, dd, *J* = 9.2, 5.9 Hz, CH), 5.16 (1H, dd, *J* = 8.5, 6.5 Hz, CH), 5.90 and 5.94 (each 1H, br s, NH), 7.28-7.39 (10H, m, ArH); **$^{13}$C-NMR** (125 MHz, $CDCl_3$) δ = 23.1 ($CD_3$), 23.1 ($CD_3$), 23.5 ($CH_2CH_2CH_2$), 23.6 ($CH_2CH_2CH_2$), 35.1 ($CH_2CO_2$), 35.2 ($CH_2CO_2$), 39.4 ($CH_2NH$), 39.5 ($CH_2NH$), 42.4 ($CH_2CO$), 43.2 ($CH_2CO$), 51.8 ($CH_3O$), 51.8 ($CH_3O$), 71.9 ($CH_2O$), 72.0 ($CH_2O$), 78.0 (CHO), 79.0 (CHO), 109.8 ($CO_2$), 109.9 ($CO_2$), 126.1 (CH arom), 126.3 (CH arom), 128.3 (CH arom), 128.4 (CH arom), 128.5 (CH arom), 128.6 (CH arom), 137.5 (C arom), 137.7 (C arom), 169.8 (CONH), 169.8 (CONH), 170.4 (COMe), 170.4 (COMe); ***m/z* (HR-ESI-MS)**: found $[M+Na]^+$ 347.1651, $C_{17}H_{20}D_3NO_5Na$ requires 347.1657.

### 1.2.3. Acetoxymethyl 2-(2-(3-acetamidopropyl)-4-phenyl-1,3-dioxolan-2-yl)acetate (14a)

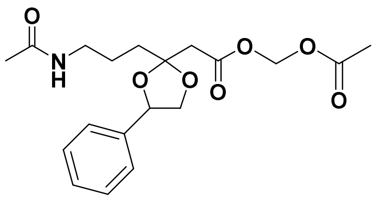

To a solution of **13a** (2.00 g, 6.2 mmol) in dry THF (40 mL) containing 3 Å molecular sieves, potassium trimethylsilanolate (3.19 g, 24.9 mmol) was added. The reaction mixture was stirred under argon atmosphere and at room temperature for 3 h.[3] The reaction was then filtered and the solvent removed *in vacuo*. The residue was partitioned between EtOAc (20 mL) and water (20 mL), the aqueous layer was lyophilised affording crude potassium 2-(2-(3-acetamidopropyl)-4-phenyl-1,3-dioxolan-2-yl)acetate (**22a**, 2.14 g, 100%), which was carried forward to the next step without further purification.

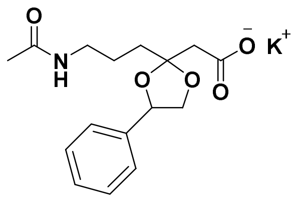

For this intermediate a double set of signals was observed for two major isomers present in 1:1 ratio; **$^1$H-NMR** (600 MHz, $D_2O$) δ = 1.65-1.78 (4H, m, $CH_2CH_2CH_2$), 1.93-2.03 (4H, m, $CH_2$), 1.94 and 1.95 (each 3H, s, $CH_3CONH$), 2.48-2.64 (4H, m, $CH_2CO$), 3.22 (4H, t, *J* = 6.7 Hz, $CH_2NH$), 3.78-3.86 (2H, m, $CH_2O$), 4.34-4.45 (2H, m, $CH_2O$), 5.15 (1H, dd, *J* = 6.2, 8.9 Hz, CH), 5.24-5.31 (1H, m, CH), 7.36-7.53 (10H, m, ArH); **$^{13}$C-NMR** (125 MHz, $D_2O$) δ = 22.6 ($CH_3$), 22.6 ($CH_3$), 23.6 ($CH_2CH_2CH_2$), 23.6 ($CH_2CH_2CH_2$), 36.2 ($CH_2CO_2$), 36.4

[3] Barrett, A. G. M.; Peña, M.; Willardsen, J. A. *J. Org. Chem.*, **1996**, *61*, 1082–1100.

($CH_2CO_2$), 40.1 ($CH_2CO$), 40.1 ($CH_2CO$), 46.4 ($CH_2NH$), 46.9 ($CH_2NH$), 71.9 ($CH_2O$), 71.9 ($CH_2O$), 78.5 (CH), 79.3 (CH), 111.7 ($CO_2$), 111.8 ($CO_2$), 127.1 (CH arom), 127.6 (CH arom), 128.9 (CH arom), 129.4 (CH arom), 129.5 (CH arom), 129.6 (CH arom), 138.0 (C arom), 138.3 (C arom), 176.3 (CONH), 176.4 (CONH), 180.3 ($COO^-$), 180.3 ($COO^-$); ***m/z*** **(HR-ESI-MS)**: found $[M+Na]^+$ 330.1311, $C_{16}H_{21}NO_5Na$ requires 330.1312).

To a solution of crude potassium 2-(2-(3-acetamidopropyl)-4-phenyl-1,3-dioxolan-2-yl)acetate (**22a**, 2.10 g, 6.1 mmol) in dry THF (40 mL), bromomethyl acetate (650 µl, 6.7 mmol) was added and the reaction mixture was stirred for 18 h at room temperature. The solvent was removed *in vacuo* to give **14a** as a yellow oil (2.27 g, 98%), used for the next step without further purification, $R_f$ = 0.25 (EtOAc). A sample of this crude material was purified by semipreparative HPLC for accurate NMR characterisation ($R_t$ = 37 min, with a gradient elution from 0 to 50% MeCN over 30 min, then 50 to 100% MeCN over 15 min). A double set of signals was observed for two major isomers present in 1:1 ratio. **$^1$H-NMR** (400 MHz, $CDCl_3$) δ = 1.65-1.79 (4H, m, $CH_2CH_2CH_2$), 1.95 and 1.97 (each 3H, s, $CH_3CONH$), 1.92-2.04 (4H, m, $CH_2$), 2.04 and 2.05 (each 3H, s, $CH_3COO$) 2.83 and 2.83 (each 2H, d, *J* = 13.0 Hz, $CH_2CO$), 3.26-3.33 (4H, m, $CH_2NH$), 3.70 (2H, t, *J* = 8.3 Hz, $CH_2O$), 4.29-4.37 (2H, m, $CH_2O$), 5.04 (1H, dd, *J* = 9.4, 5.9 Hz, CH), 5.15 (1H, dd, *J* = 8.4, 6.7 Hz, CH), 5.73-5.78 (4H, m, $OCH_2O$), 6.00 and 6.03 (each 1H, br s, NH), 7.28-7.38 (10H, m, ArH); **$^{13}$C-NMR** (125 MHz, $CDCl_3$) δ = 20.6 ($CH_3$), 20.6 ($CH_3$), 23.1 ($CH_3$), 23.1 ($CH_3$), 23.4 ($CH_2CH_2CH_2$), 23.5 ($CH_2CH_2CH_2$), 35.2 ($CH_2CO_2$), 35.3 ($CH_2CO_2$), 39.3 ($CH_2NH$), 39.4 ($CH_2NH$), 42.4 ($CH_2CO$), 43.1 ($CH_2CO$), 71.9 ($CH_2O$), 72.0 ($CH_2O$), 78.1 (CHO), 79.1 (CHO), 79.1 ($OCH_2O$), 79.1 ($OCH_2O$), 109.6 ($CO_2$), 109.7 ($CO_2$), 126.1 (CH arom), 126.2 (CH arom), 128.3 (CH arom), 128.4 (CH arom), 128.6 (CH arom), 128.6 (CH arom), 137.2 (C arom), 137.6 (C arom), 167.9 (C arom), 167.9 (C arom), 169.6 (CONH), 169.7 (CONH), 170.3 (CO), 170.3 (CO); ***m/z*** **(HR-ESI-MS)**: found $[M+Na]^+$ 402.1521, $C_{19}H_{25}NO_7Na$ requires 402.1523.

### 1.2.4. Acetoxymethyl 2-(2-(d$_3$-acetamidopropyl)-4-phenyl-1,3-dioxolan-2-yl)acetate (14b)

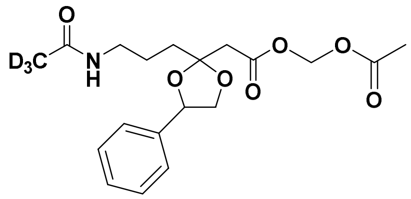

Compound **14b** was prepared in the same way as **14a** from **13b** as starting material, through potassium 2-(2-(3-d$_3$-acetamidopropyl)-4-phenyl-1,3-dioxolan-2-yl)acetate (**22b**) as intermediate:

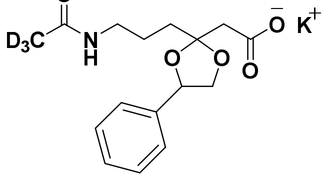

Intermediate characterisation of **22b**: **$^1$H-NMR** (400 MHz, MeOD) δ = 1.65-1.82 (4H, m, $CH_2CH_2CH_2$), 1.95-2.08 (4H, m, $CH_2$), 2.48-2.64 (4H, m, $CH_2CO$), 3.18-3.25 (4H, m, $CH_2NH$), 3.54-3.65 (2H, m, $CH_2O$), 4.31 (1H, dd, *J* = 6.0, 8.2 Hz, $CH_2O$), 4.36 (1H, dd, *J* = 6.5, 7.7 Hz, $CH_2O$), 5.03 (1H, dd, *J* = 7.0, 11.9 Hz, CH), 5.23 (1H, dd, *J* = 6.5, 8.4 Hz, CH), 7.36-7.53 (10H, m, ArH); **$^{13}$C-NMR** (125 MHz, $D_2O$) δ = 24.4 ($CH_2CH_2CH_2$), 24.4 ($CH_2CH_2CH_2$), 24.9 ($CD_3$), 24.9 ($CD_3$), 36.4 ($CH_2CH$), 36.7 ($CH_2CH$), 40.9 ($CH_2NH$), 40.9 ($CH_2NH$), 47.4 ($CH_2CO_2$), 48.0 ($CH_2CO_2$), 73.0 ($CH_2O$), 73.2 ($CH_2O$), 79.1 (CH), 80.1 (CH), 112.3 ($CO_2$), 112.5 ($CO_2$), 127.6 (CH arom), 127.7 (CH arom), 129.2 (CH arom), 129.4 (CH arom), 129.6 (CH arom), 129.7 (CH arom), 140.0 (C arom), 140.3 (C arom), 171.8 (CONH), 171.8 (CONH), 176.4 ($COO^-$), 176.4 ($COO^-$); ***m/z* (HR-ESI-MS)**: found $[M+Na]^+$ 333.1487, $C_{16}H_{18}D_3NO_5Na^+$ requires 333.1500.

For **14b**: **$^1$H-NMR** (500 MHz, $CDCl_3$) δ = 1.65-1.80 (4H, m, $CH_2CH_2CH_2$), 1.92-2.04 (4H, m, $CH_2$), 2.05 and 2.06 (each 3H, s, $CH_3COO$), 2.83 and 2.84 (each 2H, d, *J* = 16.0 Hz, $CH_2CO$), 3.28-3.35 (4H, m, $CH_2NH$), 3.70 (2H, t, *J* = 8.3 Hz, $CH_2O$), 4.32-4.36 (2H, m, $CH_2O$), 5.04 (1H, dd, *J* = 6.6, 8.4 Hz, CH), 5.15 (1H, dd, *J* = 6.0, 9.3 Hz, CH), 5.73-5.78 (4H, m, $OCH_2O$), 6.08 and 6.13 (each 1H, br s, N*H*), 7.29-7.39 (10H, m, Ar*H*); **$^{13}$C-NMR** (125 MHz, $CDCl_3$) δ = 20.6 ($CH_3$), 20.6 ($CH_3$), 23.0 ($CD_3$), 23.0 ($CD_3$), 23.4 ($CH_2CH_2CH_2$), 23.5 ($CH_2CH_2CH_2$), 35.2 ($CH_2CO_2$), 35.3 ($CH_2CO_2$), 39.4 ($CH_2CO$), 39.4 ($CH_2CO$), 42.4 ($CH_2NH$), 43.1 ($CH_2NH$), 71.9 ($CH_2O$), 72.0 ($CH_2O$), 78.1 ($CH_2CH$), 79.1 ($CH_2CH$), 79.1 ($OCH_2O$), 79.1 ($OCH_2O$), 109.6 ($CO_2$), 109.7 ($CO_2$), 126.1 (CH arom), 126.3 (CH arom), 128.3 (CH arom), 128.4 (CH arom), 128.6 (CH arom), 128.6 (CH arom), 137.3 (C arom), 137.6 (C arom), 167.9 (CO), 167.9 (CO), 169.7 (CONH), 169.8 (CONH), 170.5 (CO), 170.5 (CO); ***m/z* (HR-ESI-MS)**: found $[M+Na]^+$ 405.1701, $C_{19}H_{22}D_3NO_7Na^+$ requires 405.1682.

### 1.2.5. Methyl 2-[2-(3-acetamidopropyl)-1,3-dithiolan-2-yl]acetate (16a)

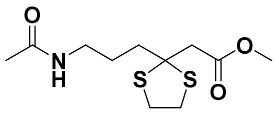


To a solution of **1a** (968 mg, 4.8 mmol) in dry $CH_2Cl_2$ (30 mL), 1,2-ethanedithiol (2.02 mL, 24.1 mmol) was added. The reaction mixture was cooled to 0°C before dropwise addition of $BF_3{\cdot}Et_2O$ (2.97 mL, 24.1 mmol) then the reaction was heated under reflux at 40 °C for 18 h. The reaction was quenched with saturated $NaHCO_3$ solution, the organic layer was dried over $MgSO_4$ and concentrated *in vacuo*. The crude product was purified by flash chromatography (9:1 DCM:MeOH, $R_f$ = 0.59) to give **16a** (1.20 g, 90%). A sample of this crude material was purified by semipreparative HPLC for accurate NMR characterisation ($R_t$ = 24.5 min, with a gradient elution 0 to 40% over 15 min, then 40 to 50 over 30 min, then 50 to 100% MeCN over 5 min). **$^1$H-NMR** (400 MHz, $CDCl_3$) δ = 1.62-1.80 (2H, m, $CH_2CH_2CH_2$), 2.00 (3H, s, $CH_3CONH$), 2.11-2.16 (2H, m, $CH_2CS_2$), 3.05 (2H, s, $CH_2$), 3.20-3.24 (2H, m, $CH_2NH$), 3.30 (4H, s, $SCH_2CH_2S$), 3.70 (3H, s, $OCH_3$), 5.90 (1H, br s, NH); **$^{13}$C-NMR** (125 MHz, $CDCl_3$) δ = 23.0 ($CH_3$), 26.8 ($CH_2CH_2CH_2$), 39.3 ($CH_2$), 39.5 ($CH_2$), 39.8 ($SCH_2CH_2S$),

48.2 ($CH_2CO$), 51.7 ($CH_3O$), 66.6 ($CS_2$), 170.4 (CONH), 170.4 (COMe); ***m/z*** **(HR-ESI-MS)**: found $[M+Na]^+$ 300.0701, $C_{11}H_{19}NO_3S_2Na^+$ requires 300.0699.

### 1.2.6. Acetoxymethyl 2-[2-(3-acetamidopropyl)-1,3-dithiolan-2-yl]acetate (17a)

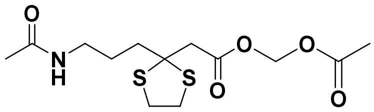

Compound **17a** was prepared in the same way as **14a** from **16a** as starting material, proceeding through the intermediate potassium 2-[2-(3-acetamidopropyl)-1,3-dithiolan-2-yl]acetate (**23a**):

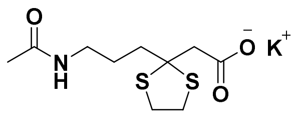

**23a**: $^1$H-NMR (700 MHz, MeOD) δ = 1.72-1.78 (2H, m, $CH_2CH_2CH_2$), 1.92 (3H, s, $CH_3$CONH), 2.16-2.19 (2H, m, $CH_2$), 2.89 (2H, s, $CH_2$CO), 3.16 (2H, t, *J* = 7.0, $CH_2$NH), 3.21-3.26 (4H, m, $SCH_2CH_2S$); $^{13}$C-NMR (176 MHz, MeOD) δ 24.4 ($CH_3$), 24.4 ($CH_2$), 40.1 ($CH_2$), 40.2 ($CH_2$), 40.8 ($CH_2$), 41.5 ($CH_2$), 53.0 ($CH_2$CO), 69.4 ($CS_2$), 173.3 (CONH), 178.3 ($COO^-$); ***m/z*** **(HR-ESI-MS)**: found $[M+H]^+$ 286.0542, $C_{10}H_{17}NO_3S_2Na^+$ requires 286.0542.
A sample of **17a** was purified by semipreparative HPLC for accurate NMR characterisation ($R_t$ = 19.5 min, with a gradient elution 0 to 65% MeCN over 15 min, then 65 to 70 MeCN over 15 min, then 70 to 100% MeCN over 5 min). $^1$**H-NMR** (400 MHz, $CDCl_3$) δ = 1.69-1.77 (2H, m, $CH_2CH_2CH_2$), 1.98 (3H, s, $CH_3$CONH), 2.08-2.14 (2H, m, $CH_2CS_2$), 2.11 (3H, s, $CH_3$COO), 3.06 (2H, s, $CH_2CO_2R$), 3.26-3.31 (2H, m, $CH_2$NH), 3.28 (4H, s, $SCH_2CH_2S$), 5.72 (2H, s, $CH_2$), 6.01 (1H, br s, NH); $^{13}$**C-NMR** (125 MHz, $CDCl_3$) δ = 20.7 ($CH_3$), 23.1 ($CH_3$), 26.8 ($CH_2CH_2CH_2$), 39.3 ($CH_2$), 39.3 ($CH_2$), 39.3 ($CH_2$), 48.1 ($CH_2$CO), 66.2 ($CS_2$), 79.1 ($OCH_2O$), 168.5 (CONH), 169.7 (CO), 170.3 (CO); ***m/z*** **(HR-ESI-MS)**: found $[M+Na]^+$ 358.0753, $C_{13}H_{21}NO_5S_2Na^+$ requires 358.0753.

### 1.2.7. Acetoxymethyl 6-acetamido-3-oxohexanoate (15a)

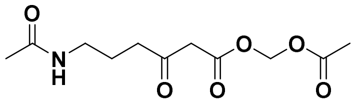

**Method A**

To a solution of **14a** (250 mg, 0.7 mmol) in dry EtOAc (20 mL) under argon atmosphere, $Pd(OAc)_2$ (200 mg, 0.9 mmol) and $Pd(CF_3COO)_2$ (100 mg, 0.3 mmol) were added [4]. $H_2$(g) (1 bar) was then flushed through and

[4] Conway, S. J.; Thuring, J. W.; Andreu, S.; Kvinlaug, B. T.; Roderick, H. L.; Bootman, M. D.; Holmes, A. B. *Aust. J. Chem.*, **2006**, *59*, 887–893.

the mixture was stirred at room temperature for 48 h. The reaction mixture was filtered through Celite and the solvent removed *in vacuo;* following HPLC purification (see conditions below), **15a** was obtained as a yellow oil (68 mg, 40%).

**Method B**

To a solution of **17a** (250 mg, 0.7 mmol) dissolved in a mixture of $CH_3CN$ (20 ml) and water (2 ml) [Bis(trifluoroacetoxy)iodo]benzene (864 mg, 2.1 mmol) was added. The reaction was stirred at room temperature for 20 minutes and then quenched with a 1:1:1 water/$NaHCO_3$/$Na_2SO_3$ mixture (20 ml in total). After an extraction with EtOAc (20 mL), the organic phase was dried over $MgSO_4$ and the solvent evaporated under reduced pressure. The crude residue was then purified by semipreparative HPLC ($R_t$ = 24 min, with a gradient elution from 0 to 50% MeCN over 30 min, then 50 to 100% MeCN over 15 min) affording **15a** as a yellow oil (125 mg, 78%). **$^1$H-NMR** (500 MHz, $(CD_3)_2SO$) δ = 1.57 (2H, app quin, *J* = 7.1 Hz, $CH_2\mathit{CH_2}CH_2$), 1.77 (3H, s, $\mathit{CH_3}CONH$), 2.07 (3H, s, $CH_3COO$), 2.52 (2H, t, *J* =7.3 Hz, $CH_2$), 2.97 (2H, m, $\mathit{CH_2}NH$), 3.68 (2H, s, $COCH_2CO$), 5.68 (2H, s, $OCH_2O$), 7.80 (1H, br s, NH); **$^{13}$C-NMR** (150 MHz, $(CD_3)_2SO$) δ = 20.4 ($CH_3$), 22.6 ($CH_3$), 23.0 ($CH_2\mathit{C}H_2CH_2$), 37.6 ($CH_2$), 39.5 ($CH_2$), 48.4 ($CH_2$), 79.1 ($OCH_2O$), 166.3 (CONH), 169.1 (CO), 169.2 (CO), 202.7 (CO); ***m/z*** **(HR-ESI-MS)**: found $[M+Na]^+$ 282.0942, $C_{11}H_{17}NO_6Na^+$ requires 282.0948.

### 1.2.8. Acetoxymethyl 6-d$_3$-acetamido-3-oxohexanoate (15b)

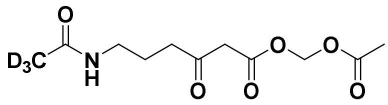

Compound **15b** was prepared in the same way as **15a** (see above) from **14b** as starting material.

**$^1$H-NMR** (500 MHz, $(CD_3)_2SO$) δ = 1.57 (2H, app quin, *J* = 7.1 Hz, $CH_2\mathit{CH_2}CH_2$), 2.08 (3H, s, $CH_3COO$), 2.52 (2H, t, *J* = 7.3 Hz, $CH_2$), 2.97 (2H, app q, *J* = 6.6, $\mathit{CH_2}NH$), 3.68 (2H, s, $COCH_2CO$), 5.68 (2H, s, $OCH_2O$), 7.80 (1H, br s, NH); **$^{13}$C-NMR** (150 MHz, $(CD_3)_2SO$) δ = 20.4 ($CH_3$), 22.6 ($CH_3$), 23.0 ($CH_2\mathit{C}H_2CH_2$), 37.6 ($CH_2$), 39.6 ($CH_2$), 48.4 ($COCH_2$), 79.1 ($OCH_2O$), 166.3 (CONH), 169.1 (CO), 169.2 (CO), 202.7 (CO); ***m/z*** **(HR-ESI-MS)**: found $[M+Na]^+$ 285.1132, $C_{11}H_{14}D_3NO_6Na^+$ requires 285.1136.

## 1.3. Synthesis of *N*-(4,6-dioxoheptyl)decanamide (20)

Compound **3** was prepared as reported previously.[5]

[5] E. Riva, I. Wilkening, Ina, S. Gazzola, W. M. A. Li, L Smith, P. F. Leadlay, M. Tosin, *Angew. Chem. Int. Ed.*, **2014**, *53*, 11944—11949.

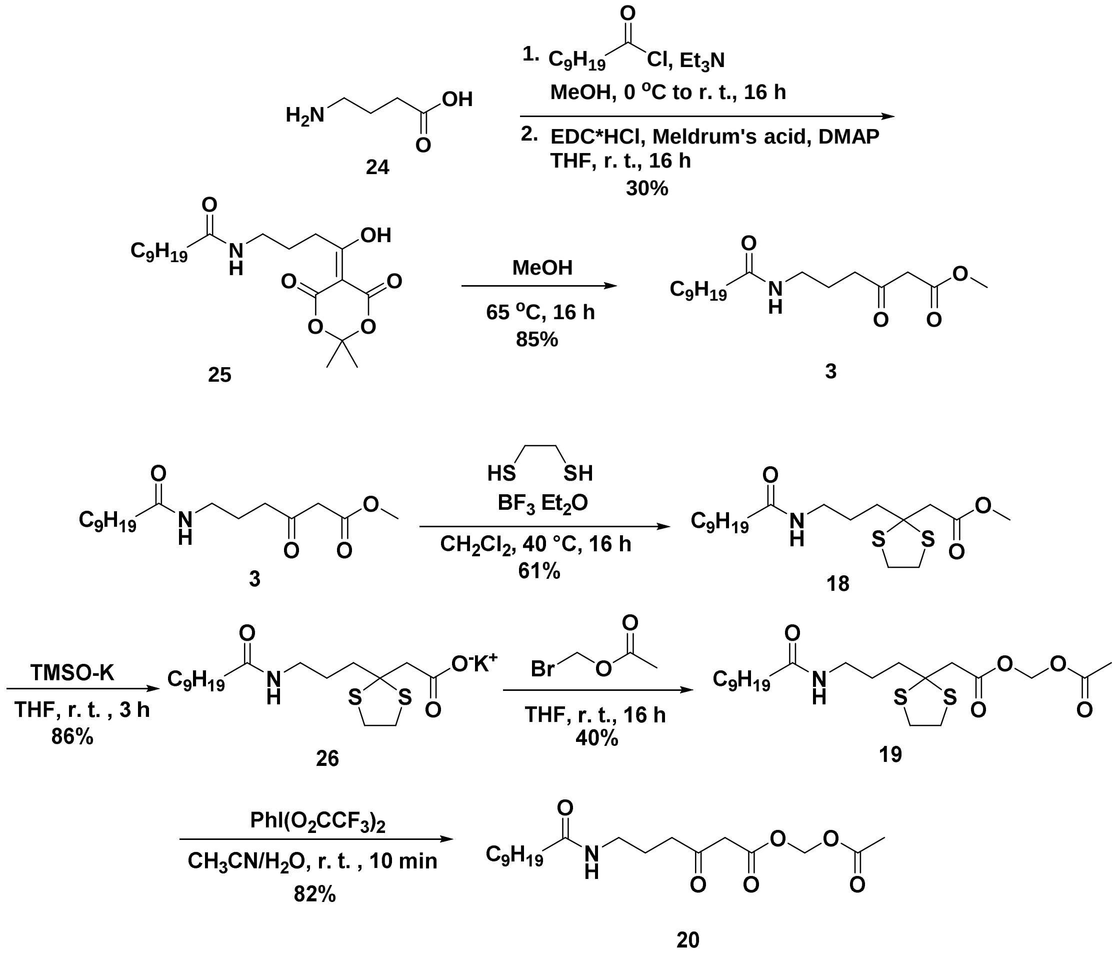

### 1.3.9. Methyl-2-(2-(3-decanamidopropyl)-1,3-dithiolan-2-yl)acetate (18)

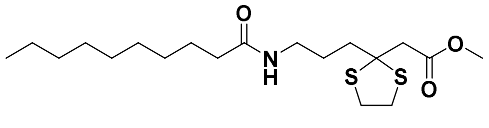

Compound **3** (800 mg, 2.55 mmol) and 1,2-ethanthiol (1.07 mL, 12.8 mmol) were dissolved in dichloromethane (16 mL) under argon atmosphere. The solution was cooled to 0 °C before $BF_3{\cdot}OEt_2$ (1.57 mL, 12.8 mmol) was slowly added. The reaction was stirred under reflux overnight. Then, a saturated solution of $NaHCO_3$ was added at 0 °C up to pH 9 and the mixture was extracted with $CH_2Cl_2$ (3x10 mL). The crude product was purified by column chromatography (cyclohexane: EtOAc, 7:3 to 1:1)) and **18** was obtained as a white powder (600 mg, 61%).

**$^1$H-NMR** (400 MHz, $CDCl_3$): δ = 0.87 (3H, t, $J$ = 6.5 Hz, $CH_3$), 1.27 (12H, br s, $(CH_2)_6$), 1.58 (2H, m, $CH_2CH_2CONH$), 1.75 (2H, m, $NHCH_2CH_2$), 2.14 (2H, m, $CH_2CS_2$), 2.14 (2H, m, $CH_2CONH$), 3.04 (2H, s, $CH_2COO$), 3.29 (2H, m, $NHCH_2$), 3.30 (4H, s, $SCH_2CH_2S$), 3.70 (3H, s, $OCH_3$), 5.51 (1H, br s, NH); **$^{13}$C-NMR** (101 MHz, $CDCl_3$): δ = 14.1 ($CH_3$), 22.6 ($CH_2CH_3$), 22.6 ($CH_2CH_2CS_2$), 25.8 ($CH_2$), 27.1($CH_2$), 29.3 ($CH_2$), 29.3 ($CH_2$), 29.4 ($CH_2$), 29.4 ($CH_2$), 36.9 ($CH_2CONH$), 39.1 ($CH_2CS_2$), 39.4 ($SCH_2CH_2S$), 39.8 ($NHCH_2$), 48.2 ($CH_2COO$), 51.7

($OCH_3$), 66.6 ($CS_2$), 170.5 (C=O), 173.1 (C=O); ***m/z* (HR-ESI-MS)**: found $[M+H]^+$ 412.1945, $C_{19}H_{36}NO_3S_2^+$ requires 412.1951.

### 1.3.10. Potassium 2-(2-(3-decanamidopropyl)-1,3-dithiolan-2-yl)acetate (26)

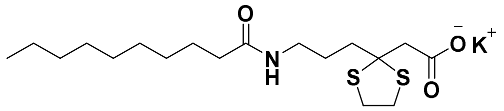

To a solution of compound **18** (600 mg, 1.54 mmol) in dry THF (10 mL) containing 3 Å molecular sieves, was added potassium trimethilsilanolate (789 mg, 6.16 mmol). The reaction was stirred under argon atmosphere for 3 h at room temperature. Then the reaction was filtered under vacuum and concentrated. The residue was dissolved in water (5 mL), EtOAc (5 mL) was added, and the two phases were separated. The aqueous layer was freeze-dried affording **26** as a white powder (565 mg, 86%).

**$^1$H-NMR** (400 MHz, $D_2O$): δ = 0.79 (3H, br s, $CH_3$), 1.22 (12H, br s, $(CH_2)_6$), 1.53 (2H, br s, $CH_2CH_2CONH$), 1.64 (2H, br s, $NHCH_2CH_2$), 1.84 (4H, s, $CH_2CS_2$), 2.01 (2H, m, $CH_2CO$), 2.17 (2H, t, *J* = 7.2 Hz, $CH_2COOK$), 3.13 (2H, m, $CH_2NH$), 3.22 (4H, m, $SCH_2CH_2S$); **$^{13}$C-NMR** (101 MHz, $D_2O$): δ = 13.2 ($CH_3$), 21.9 ($CH_2CH_3$), 25.3 ($CH_2CH_2CS_2$), 26.1 ($CH_2$), 28.2 ($CH_2$), 28.4 ($CH_2$), 28.5 ($CH_2$), 28.7 ($CH_2$), 31.1 ($CH_2CONH$), 35.5 ($CH_2CONH$), 38.2 ($CH_2CS_2$), 38.7 ($SCH_2CH_2S$), 39.4 ($SCH_2CH_2S$), 50.1 ($CH_2COO$), 67.1 ($CS_2$), 175.7 (C=O), 177.4 (C=O), 180.9 (C=O); ***m/z* (ESI-MS)**: found $[M-H]^-$ 374.2, $C_{18}H_{32}NO_3S_2^-$ requires 374.2.

### 1.3.11. Acetoxymethyl-2-(2-(3-decanamidopropyl)-1,3-dithiolan-2-yl)acetate (19)

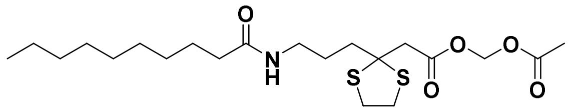

Compound **26** (545 mg, 1.32 mmol) was suspended in dry THF (9 mL) and bromoethylacetate (140 µL, 1.58 mmol) was added. The reaction was stirred at room temperature overnight and afterwards concentrated under vacuum. The crude product was purified by column chromatography (EtOAc/CycloHex 6:4) to yield **19** as a white powder (220 mg, 40%).

**$^1$H-NMR** (400 MHz, $CDCl_3$): δ = 0.88 (3H, t, *J* = 6.4 Hz, $CH_3$), 1.29 (12H, br s, $(CH_2)_6$), 1.27 (2H, m, $CH_2CH_2CONH$), 1.56 (2H, m, $NHCH_2CH_2$), 2.12 (3H, s, $COCH_3$), 2.13 (2H, m, $CH_2CS_2$), 2.13 (2H, m, $CH_2CONH$), 3.08 (2H, s, $CH_2COCH_3$), 3.30 (2H, m, $NHCH_2$), 3.31 (4H, s, $SCH_2CH_2S$), 5.55 (1H, br s, NH), 5.75 (1H, s, $OCH_2O$); **$^{13}$C-NMR** (101 MHz, $CDCl_3$): δ = 14.1 ($CH_3$), 20.7 ($COCH_3$), 22.7 ($CH_2CH_3$), 22.7 ($CH_2CH_2CS_2$), 25.8 ($CH_2$), 27.1 ($CH_2$), 29.3 ($CH_2$), 29.3 ($CH_2$), 29.4 ($CH_2$), 29.5 ($CH_2$), 36.9 ($CH_2CONH$), 39.1 ($CH_2CS_2$), 39.4 ($SCH_2CH_2S$), 39.8 ($NHCH_2$), 48.2 ($CH_2COO$), 66.4 ($CS_2$), 79.2 ($OCH_2O$), 168.6 (C=O), 169.9 (C=O),173.2 (C=O); ***m/z* (HR-ESI-MS)**: found $[M+Na]^+$ 470.2006, $C_{21}H_{37}NNaO_5S_2^+$ requires 470.2005.

### 1.3.12. Acetoxymethyl-6-decanamido-3-oxohexanoate (20)

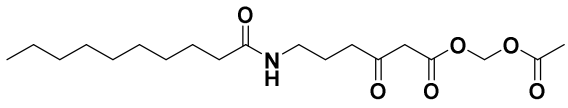

Compound **19** (200 mg, 0.45 mmol) was dissolved in acetonitrile (12 mL) and water (1.12 mL) and [bis(trifluoroacetoxy)iodo]benzene (576 mg, 1.34 mmol) was added. The reaction was stirred at room temperature for 10 minutes, quenched with $H_2O$/$NaHCO_3$ sat./$Na_2S_2O_3$ sat. solution (1:1:1, 12 mL). The organic solvent was separated and removed under reduced pressure. The final mixture was extracted with dichloromethane (3x5 mL), the organic layer was dried, filtered, concentrated and the crude was purified by column chromatography (eluent 7:3 EtOAc/CycloHex). **20** was obtained as a white powder (80 mg, 40%). The compound was further purified by semipreparative HPLC ($R_t$ = 17.7 min, with a gradient elution from 30 to 70% MeCN over 10 min, 70% MeCN for 10 min, then 70 to 100% MeCN over 5 min).

**$^1$H-NMR** (400 MHz, $CDCl_3$): δ = 0.86 (3H, t, *J* = 6.1 Hz, $CH_3$), 1.25 (12H, br s, $(CH_2)_6$), 1.59 (2H, m, $CH_2CH_2CONH$), 1.80 (2H, m, $CH_2CH_2CO$), 2.12 (3H, s, $COCH_3$), 2.14 (2H, m, $CH_2CONH$), 2.60 (2H, t, *J* = 6.8 Hz, $CH_2CO$), 3.24 (2H, m, $NHCH_2$), 3.50 (2H, s, $COCH_2CO$), 5.73 (1H, br s, NH), 5.75 (1H, s, $OCH_2O$); **$^{13}$C-NMR** (101 MHz, $CDCl_3$): δ = 14.1 ($CH_3$), 20.5 ($COCH_3$), 22.1 ($CH_2CH_3$), 23.9 ($CH_2$), 25.8 ($CH_2$), 29.3 ($CH_2$), 29.3 ($CH_2$), 29.4 ($CH_2$), 31.8 ($CH_2$), 31.8 ($CH_2$), 38.5 ($CH_2CONH$), 40.4 ($NHCH_2$), 40.4 ($CH_2COCH_2$), 48.7 ($COCH_2CO$), 79.5 ($OCH_2O$), 166.1 (C=O), 169.7 (C=O), 173.4 (C=O), 200.8 (C=O); ***m/z* (HR-ESI-MS)**: found $[M+Na]^+$ 394.2202, $C_{19}H_{33}NNaO_6^+$ requires 394.2200.

### 1.3.13. Synthesis of methyl 6-(10-azidodecanamido)-3-oxohexanoate (4)

Compound **4** was prepared as reported previously.[5]

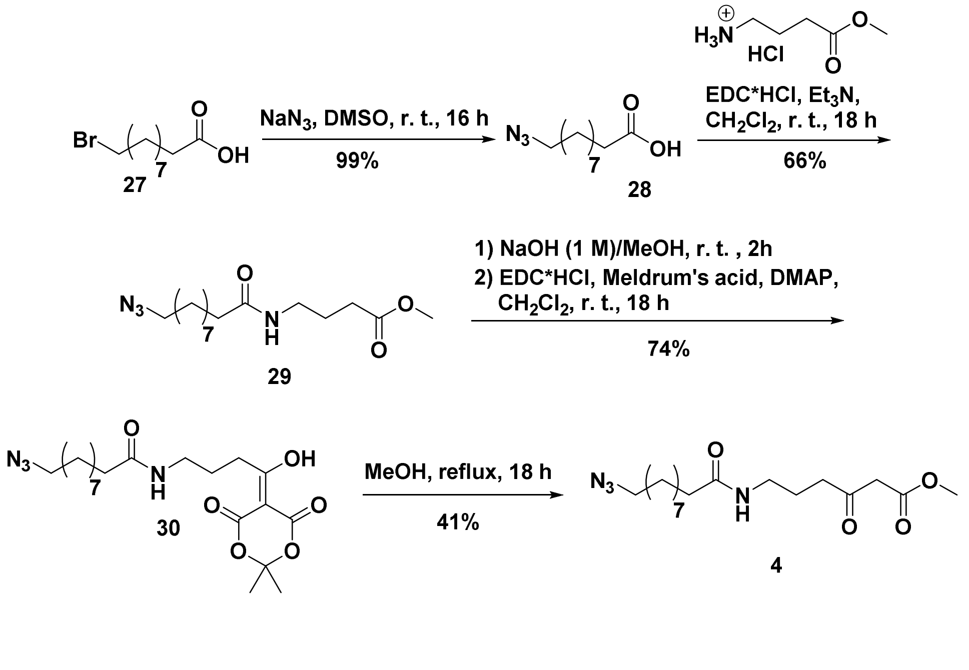

# 2. Growth of *S. lasaliensis* ACP12 (S970A) and mass spectrometry analysis

*S. lasaliensis* ACP12 (S970A)[6] was grown in M79 medium (10 mL) for 3 days at 30 ºC. This seed culture was used to inoculate MYM liquid cultures (10 mL, in duplicate copy). These were incubated at 30°C and shaken at 180 rpm for a total of 5 days. After the first day of incubation, the 0.01 mmol of probes (**3**, **21**, **8**) were added daily in 50 µL of MeOH. Control liquid cultures in absence of the probes were also prepared. After incubation all the cultures were extracted twice with ethyl acetate (10 mL x 2). The extracts were concentrated and the residues were redissolved in HPLC-grade methanol (1 mL) for mass spectrometry analysis. The Staudinger reaction on organic extracts of feeding experiments with probe **8** was performed as described preiviously.[5]

HPLC-HR-ESI-MS analyses of *S. lasaliensis ACP12 (S970A)* extracts were performed on:

1) a MaXis Impact UHR-ESI-TOF (Bruker Daltonics). Method 1: 10% B 0-2.7 min; 10-100% B 2.7-42.7 min; 100% B 42.7-62.7 min; 100-10% B 62.7-65.7 min; 10% B 65.7-77.7 min, using an Acquity UPLC HSS T3 column at a flow rate of 0.05 mL/min. Method 2: 5% B 0-5.3 min; 5-100% B 5.3-17.3 min; 100% B 17.3-22.3 min; 100-5% B 22.3-25.3 min; 5% B 25.3-35.3 min, using Agilent Eclipse C18 at a flow rate of 0.2 mL/min. Spectra were recorded in positive ionisation mode, scanning from *m/z* 50 to 2000, with Capillary Voltage set at 3500 V, Dry Heater set at 180°C and UV Lamp set at 210 nm. Selected ion search within 5 ppm was performed, as well as high resolution fragmentation for the putative biosynthetic intermediates.

2) a Thermo Orbitrap Fusion (Q-OT-qIT, Thermo) instrument. Reversed phase chromatography was used to separate the mixtures prior to MS analysis. Two columns were utilized: an Acclaim PepMap µ-precolumn cartridge 300 µm i.d. x 5 mm 5 µm 100 Å and an Acclaim PepMap RSLC 75 µm x 15 cm 2 µm 100 Å (Thermo Scientific). The columns were installed on an Ultimate 3000 RSLCnano system (Dionex). Mobile phase buffer A was composed of 0.1% (v/v) aqueous formic acid and mobile phase B was composed of 100% acetonitrile containing 0.1% (v/v) formic acid. Samples were loaded onto the µ-precolumn equilibrated in 2% aqueous acetonitrile containing 0.1% (v/v) trifluoroacetic acid for 8 min at 10 µL $min^{-1}$ after which compounds were eluted onto the analytical column following a 75 min gradient for which the mobile phase B concentration was increased from 50% B to 99.5% over 15 min, then maintained at 99.5% B for 35 minutes, then decreased to 50% over 16 min, followed by a 9 min wash at 50% B. Eluting cations were converted to gas-phase ions by electrospray ionization and analyzed. Survey scans of precursors from 150 to 1500 *m/z* were performed at 60K resolution (at 200 *m/z*) with a $5 \times 10^5$ ion count target. Tandem MS was performed by isolation at 0.7 Th with the quadrupole, HCD fragmentation with normalized collision energy of 30, and rapid scan MS analysis in the ion trap. The $MS^2$ ion count target was set to $10^4$ and the maximum injection time was 35 ms. A filter targeted inclusion mass list was used to select the precursor ions. The dynamic

---

[6] M. Tosin, L. Smith, P. F. Leadlay *Angew. Chem. Int. Ed*, **2011**, *50*, 11930-11933.

exclusion duration was set to 45 s with a 10 ppm tolerance around the selected precursor and its isotopes. Monoisotopic precursor selection was turned on. The instrument was run in top speed mode with 5 s cycles, meaning the instrument would continuously perform $MS^2$ events until the list of nonexcluded precursors diminishes to zero or 5 s, whichever is shorter. Fusion runs were performed with Survey scans of precursors from 150 to 1500 *m*/*z* 60K resolution (at 200 *m*/*z*) with a $1 \times 10^6$ ion count target. Tandem MS was performed by isolation at 1.8 Th with the ion-trap, CAD fragmentation with normalized collision energy of 32, and 15K resolution scan MS analysis in the Orbitrap. The data dependent top 20 precursors were selected for $MS^2$. $MS^2$ ion count target was set to $4 \times 10^6$ and the max injection time was 50 ms. The dynamic exclusion duration was set to 40 s with a 10 ppm tolerance around the selected precursor and its isotopes.

## 2.1. Summary of captured intermediates for *S. lasaliensis* ACP12(S970A)

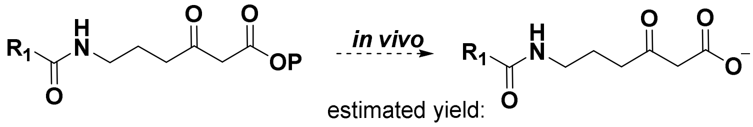


| | $R_1$ | P | | |
|---|---|---|---|---|
| **1a** | $CH_3$ | Me | 5-10% | **5a** |
| **1b** | $CD_3$ | Me | 5-10% | **5b** |
| **3** | $(CH_2)_8CH_3$ | Me | 20-30% | **7** |
| **4** | $(CH_2)_9N_3$ | Me | 50-70% | **8** |
| **15a** | $CH_3$ | AM | ~95% | **5a** |
| **15b** | $CD_3$ | AM | ~95% | **5b** |
| **20** | $(CH_2)_8CH_3$ | AM | ~95% | **7** |

**Figure 1S**: Overview of ester probes utilized for *in vivo* intermediate capture in *S. lasaliensis* ACP12 (S970A) together with estimated *in vivo* deprotection yields (qualitative estimation by LC-MS).

**Table 1S:** Overview of intermediates captured and characterized from *S. lasaliensis* ACP12 (S970A) *via* probes **1a-b**, **3**, **4**, **15a-b** and **20** (detected on Maxis impact, MI, and Orbitrap Fusion, OF).

| **intermediate** | **putative structure** | **probes 1 a-b[c]** $R_1$= $CH_3/CD_3$ P= $CH_3$ short chain/ Me | **probe 3[b]** $R_1$= $(CH_2)_8CH_3$ P= $CH_3$ deca chain/ Me | **probe 4[c]** $R_1$= $(CH_2)_8N_3$ P= $CH_3$ $N_3$ deca/ Me | **probes 15 a-b[d]** $R_1$= $CH_3/CD_3$ P= $CH_2CO_2CH_3$ short chain/ AM | **probe 20[d]** $R_1$= $(CH_2)_8CH_3$ P= $CH_2CO_2CH_3$ deca chain/ AM |
|---|---|---|---|---|---|---|
| **Diketides** | 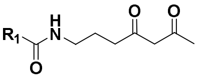 | ✓ | ✓ (MI, OF) | ✓ (MI, OF) | | ✓ (MI, OF) |
| | 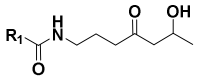 | ✓ | ✓ (OF) | ✓ (MI, OF) | | ✓ (MI, OF) |
| | 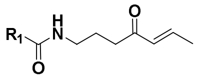 | ✓ | ✓ (OF) | ✓ | ✓ | ✓ (MI, OF) |
| **Triketides** | 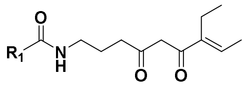 | | ✓ (OF) | ✓ (MI, OF) | | ✓ (OF) |
| | 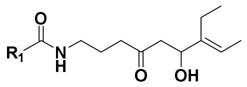 | | ✓ (OF) | | | ✓ (MI, OF) |
| | 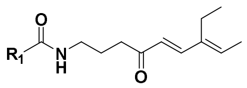 | | ✓ (OF) | | | ✓ (MI, OF) |

| | | | | | | |
|---|---|---|---|---|---|---|
| | 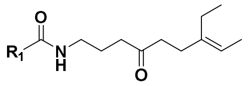 | | ✓ (MI, OF) | ✓ (OF) | | ✓ (MI, OF) |
| **Tetraketides** | 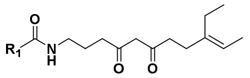 | | ✓ (MI, OF) | | | ✓ (MI, OF) |
| | 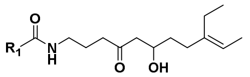 | | ✓ (OF) | ✓ (MI, OF) | | ✓ (MI, OF) |
| | 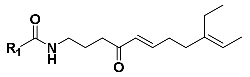 | | ✓ (OF) | ✓ (MI, OF) | | ✓ (OF) |
| **Pentaketides** | 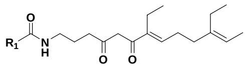 | | ✓ (MI, OF) | ✓ (MI, OF) | | ✓ (MI, OF) |
| | 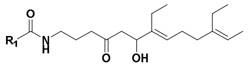 | | ✓ (MI, OF) | ✓ (OF) | | ✓ (OF) |
| | 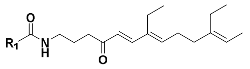 | | ✓ (MII) | ✓ (MI) | | |
| | 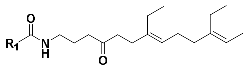 | | ✓ (OF) | ✓ (MI, OF) | | ✓ (MI, OF) |
| **Hexaketides** | 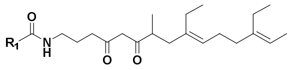 | | ✓ (MI, OF) | | | ✓ (MI, OF) |
| | 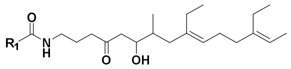 | | ✓ (MI, OF) | | | |
| **Heptaketides** | 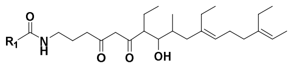 | | ✓ (MI) | | | ✓ (MI) |
| **Octaketides** | 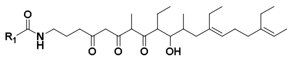 | | ✓ (MI, OF) | | | ✓ (MI, OF) |
| **Nonaketides** | 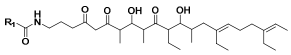 | | ✓ (MI) | ✓ (MI) | | ✓ (MI) |
| | 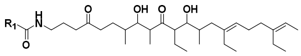 | | | ✓ (MI) | | ✓ (MI) |

| | | | | | | |
|---|---|---|---|---|---|---|
| **Undecaketides** | 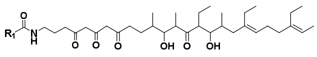 | | (MI) ✓ | | | |
| | 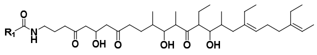 | | | | | ✓ (MI |
| **Dodecaketides** | 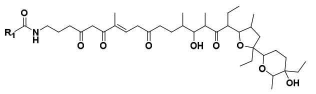 | | ✓ (MI) | ✓ (MI) | | ✓ (MI) |
| | 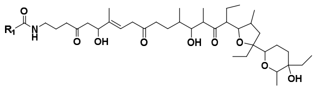 | | | | | ✓ (MI) |
| | 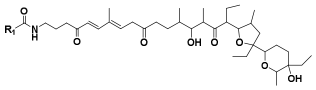 | | ✓ (MI) | ✓ (MI) | | ✓ (MI) |
| | 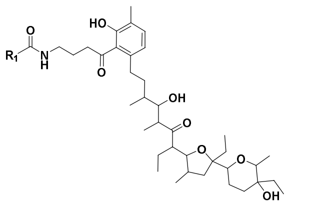 | | ✓ (MI) | ✓ (MI) | | ✓ (MI) |

## 2.2. Intermediates captured by methyl 6-decanamido-3-oxohexanoate (3)

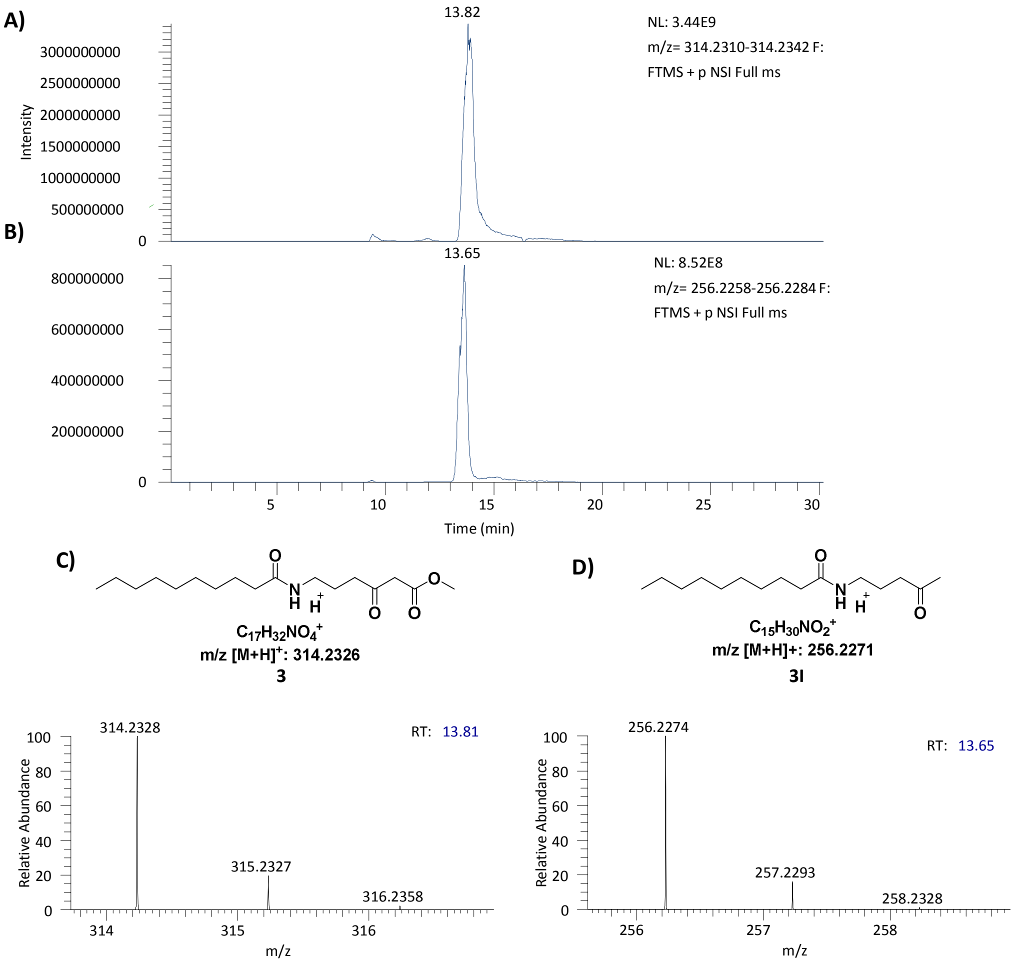


**Figure 2S:** (A) LC-HRMS analysis (Orbitrap Fusion) of the organic extracts of *S. lasaliensis* ACP12 (S970A) grown in the presence of **3** (final concentration 4 mM): $[M+H]^+$ extracted ion chromatogram (EIC) for probe **3** (Rt = 13.82 min) and (B) EIC for the decarboxylated probe **31** (Rt = 13.65 min) are shown. (C) The high resolution masses of probe **3** and (D) of the hydrolysed-decarboxylated probe **31** are shown.

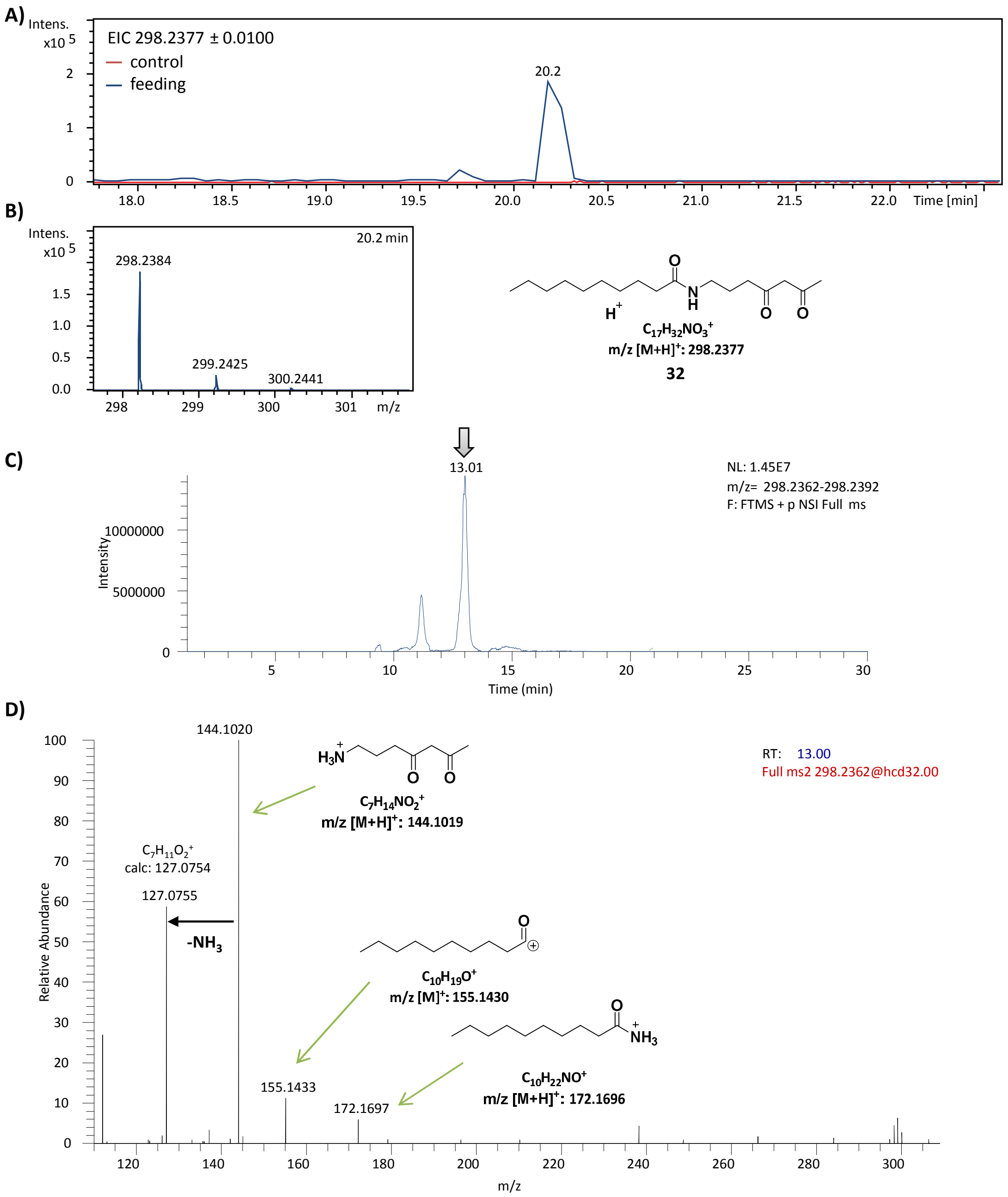


**Figure 3S:** (A) LC-HRMS analysis (MaXis Impact, method 2) of the organic extracts of *S. lasaliensis* ACP12 (S970A) grown in the absence (red) and in the presence (blue) of **3** (final concentration 4 mM): $[M+H]^+$ extracted ion chromatogram (EIC) and (B) high resolution mass are shown for the putative intermediate **32** (Rt = 20.2 min). (C) LC-HRMS analysis (Orbitrap Fusion) of the organic extracts of *S. lasaliensis* ACP12 (S970A) grown in the presence of **3** (final concentration 4 mM)**:** EIC (Rt = 13.01 min) and (D) fragmentation of **32** with putative fragment structural assignment.

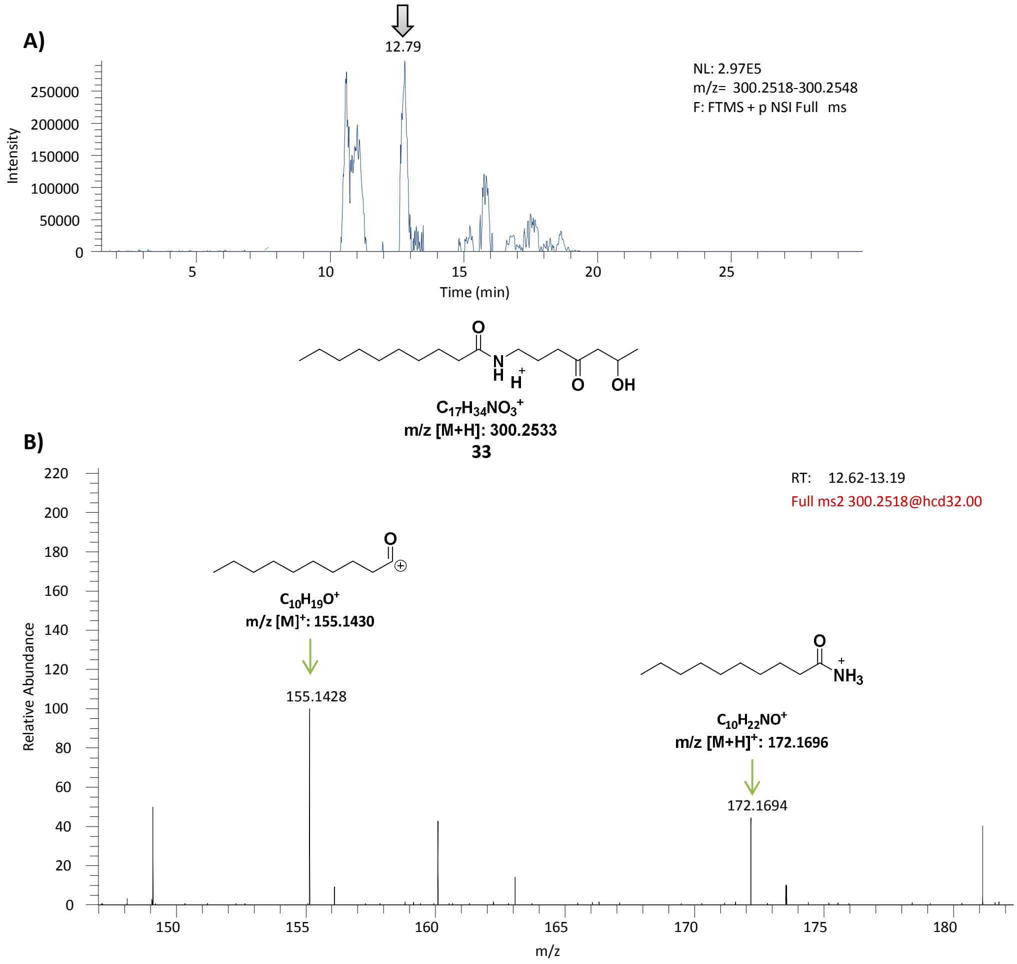


**Figure 4S**: (A) LC-HRMS analysis (Orbitrap Fusion) of the organic extracts of *S. lasaliensis* ACP12 (S970A) grown in the presence of **3** (final concentration 4 mM)**:** EIC (Rt = 12.79 min) and (B) fragmentation of putative intermediate **33** with putative fragment structural assignment.

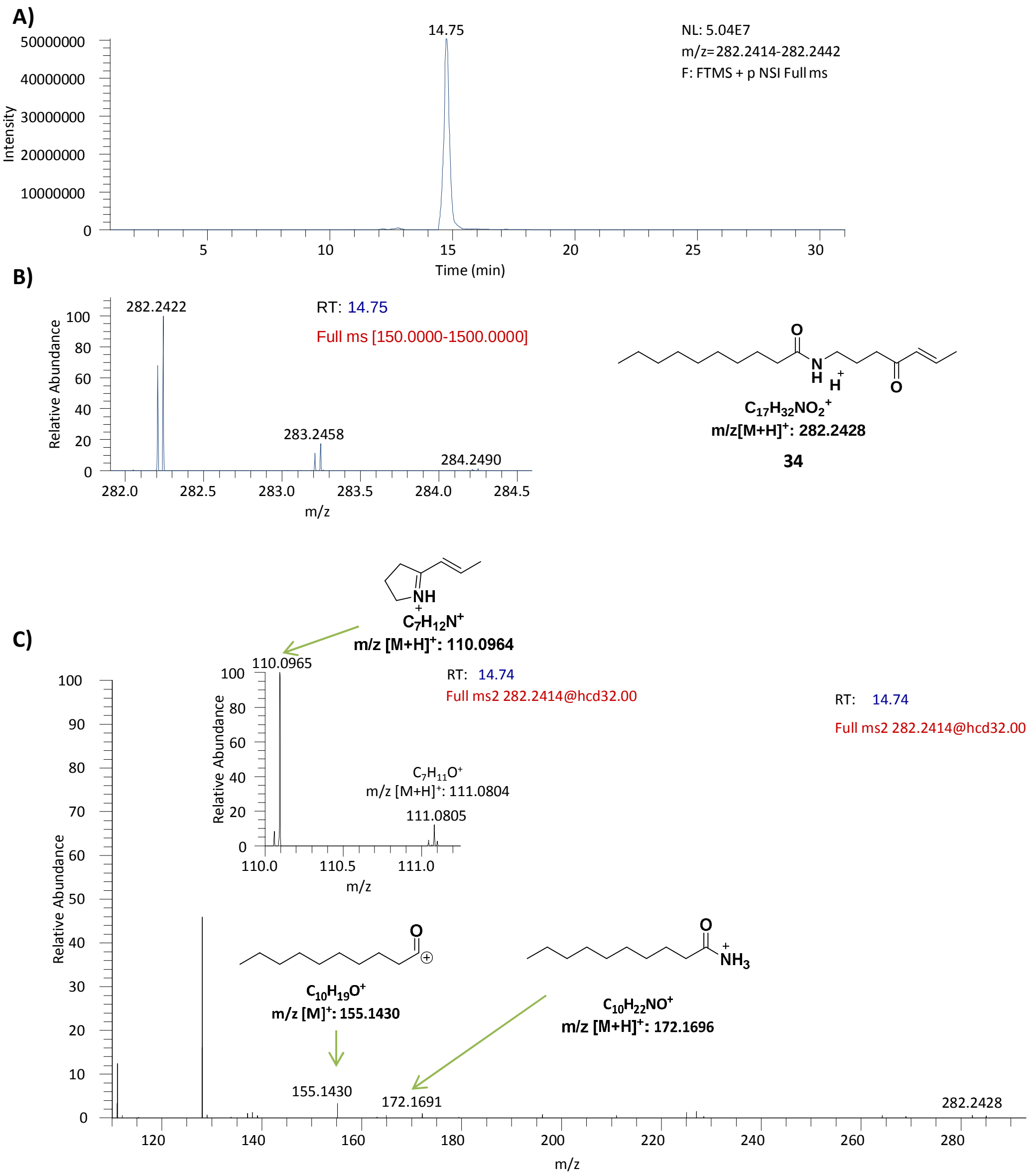


**Figure 5S:** (A) LC-HRMS analysis (Orbitrap Fusion) of the organic extracts of *S. lasaliensis* ACP12 (S970A) grown in the in the presence of **3** (final concentration 4 mM): $[M+H]^+$ extracted ion chromatogram (EIC), (B) high resolution mass (Rt = 14.75 min) and (C) fragmentation of putative intermediate **34** with putative fragment structural assignment are shown.

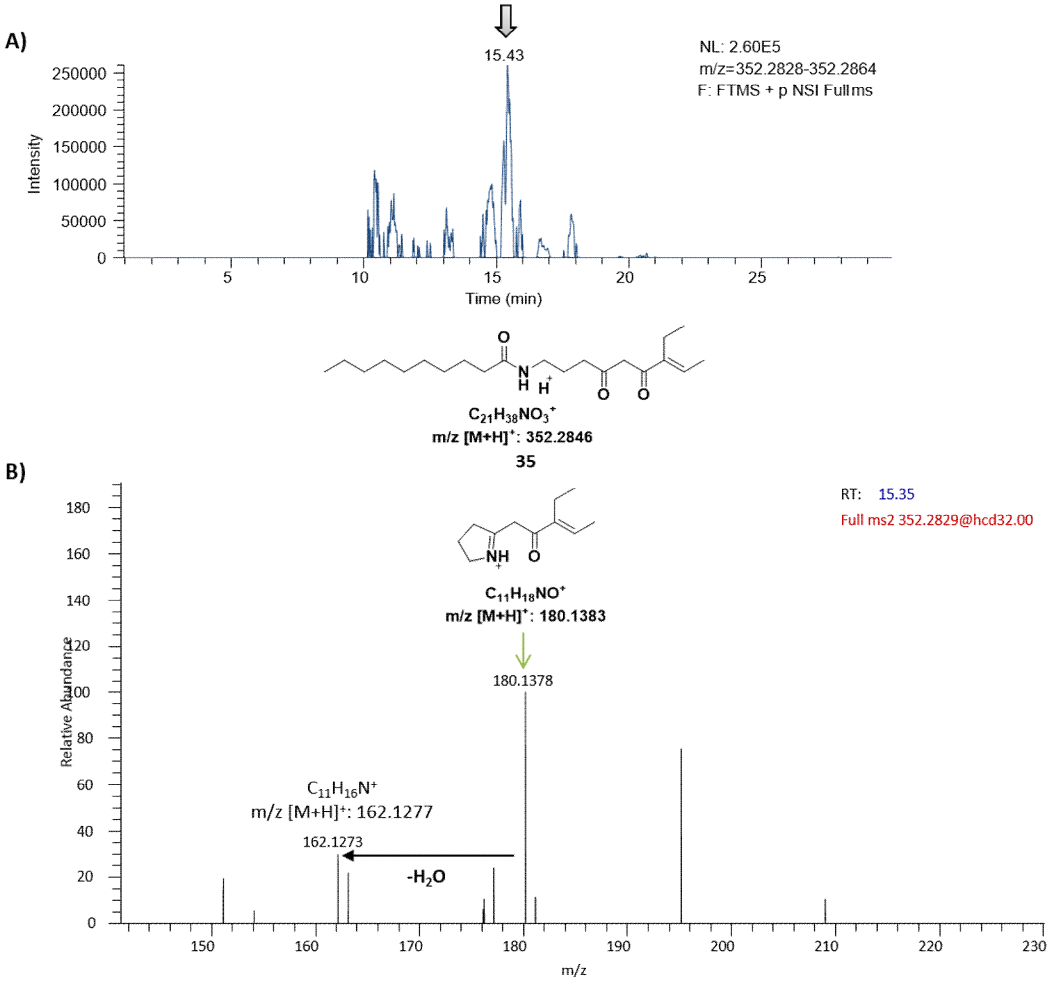


**Figure 6S:** (A) LC-HRMS analysis (Orbitrap Fusion) of the organic extracts of *S. lasaliensis* ACP12 (S970A) grown in the presence of **3** (final concentration 4 mM)**:** EIC (Rt = 15.43 min) and (B) fragmentation of putative intermediate **35** with putative fragment structural assignment.

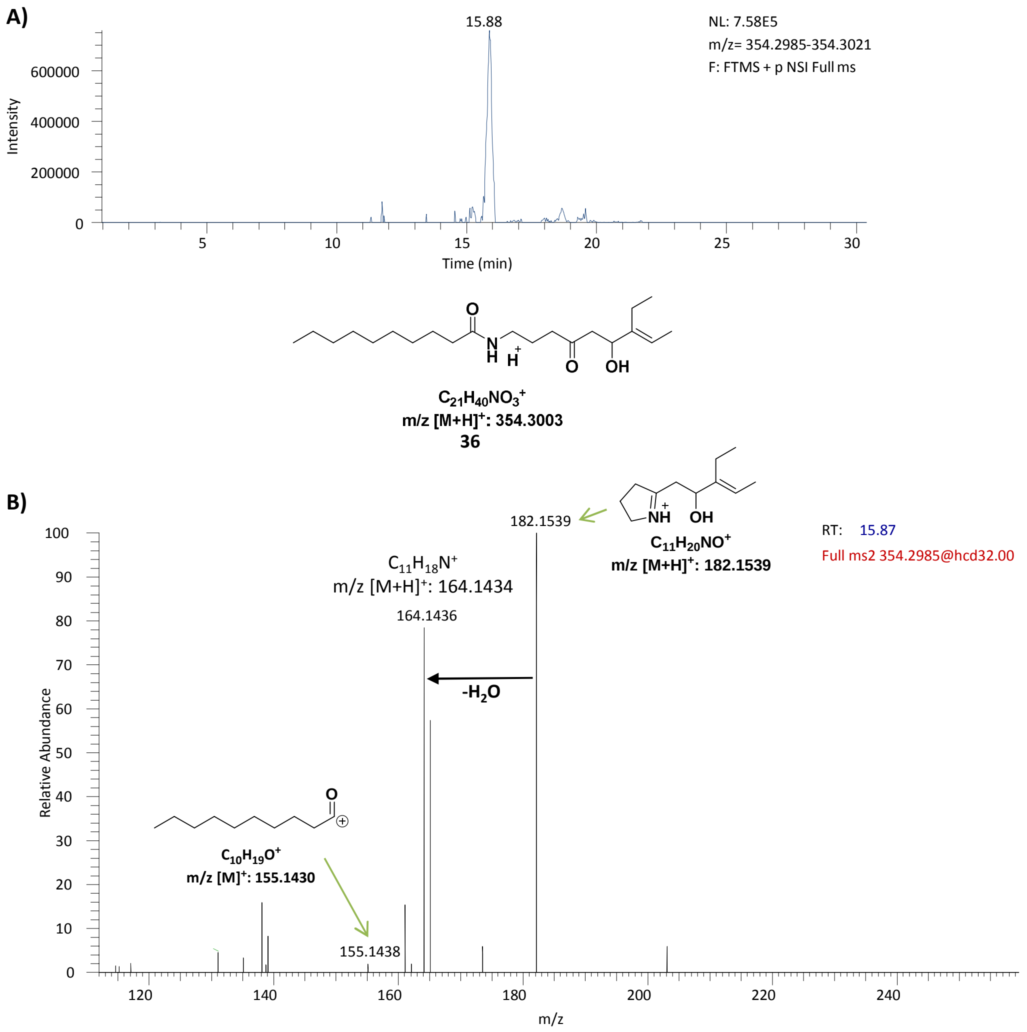


**Figure 7S**: A) LC-HRMS analysis (Orbitrap Fusion) of the organic extracts of *S. lasaliensis* ACP12 (S970A) grown in the presence of **3** (final concentration 4 mM)**:** EIC (Rt = 15.88 min) and (B) fragmentation of putative intermediate **36** with putative fragment structural assignment.

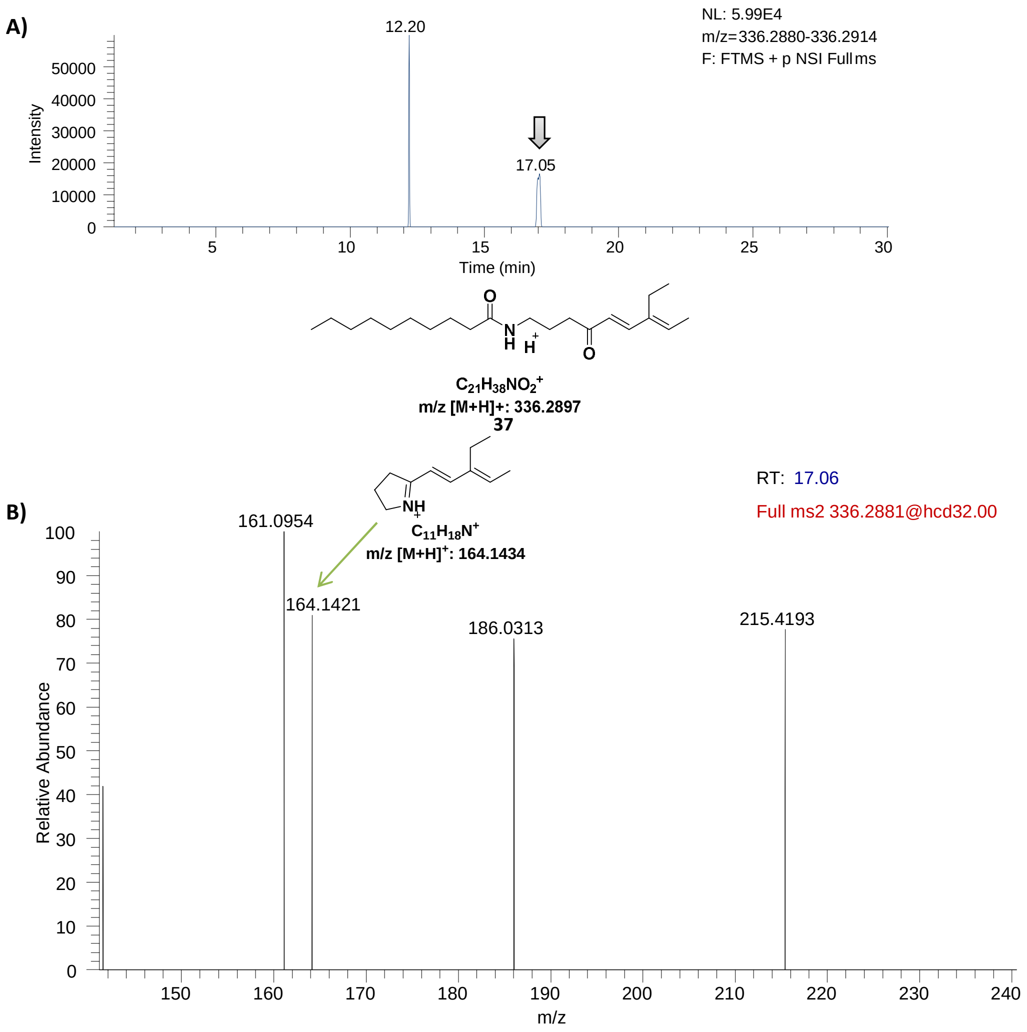


**Figure 8S:** A) LC-HRMS analysis (Orbitrap Fusion) of the organic extracts of *S. lasaliensis* ACP12 (S970A) grown in the presence of **3** (final concentration 4 mM)**:** EIC (Rt = 17.05 min) and (B) fragmentation of putative intermediate **37** with putative fragment structural assignment.

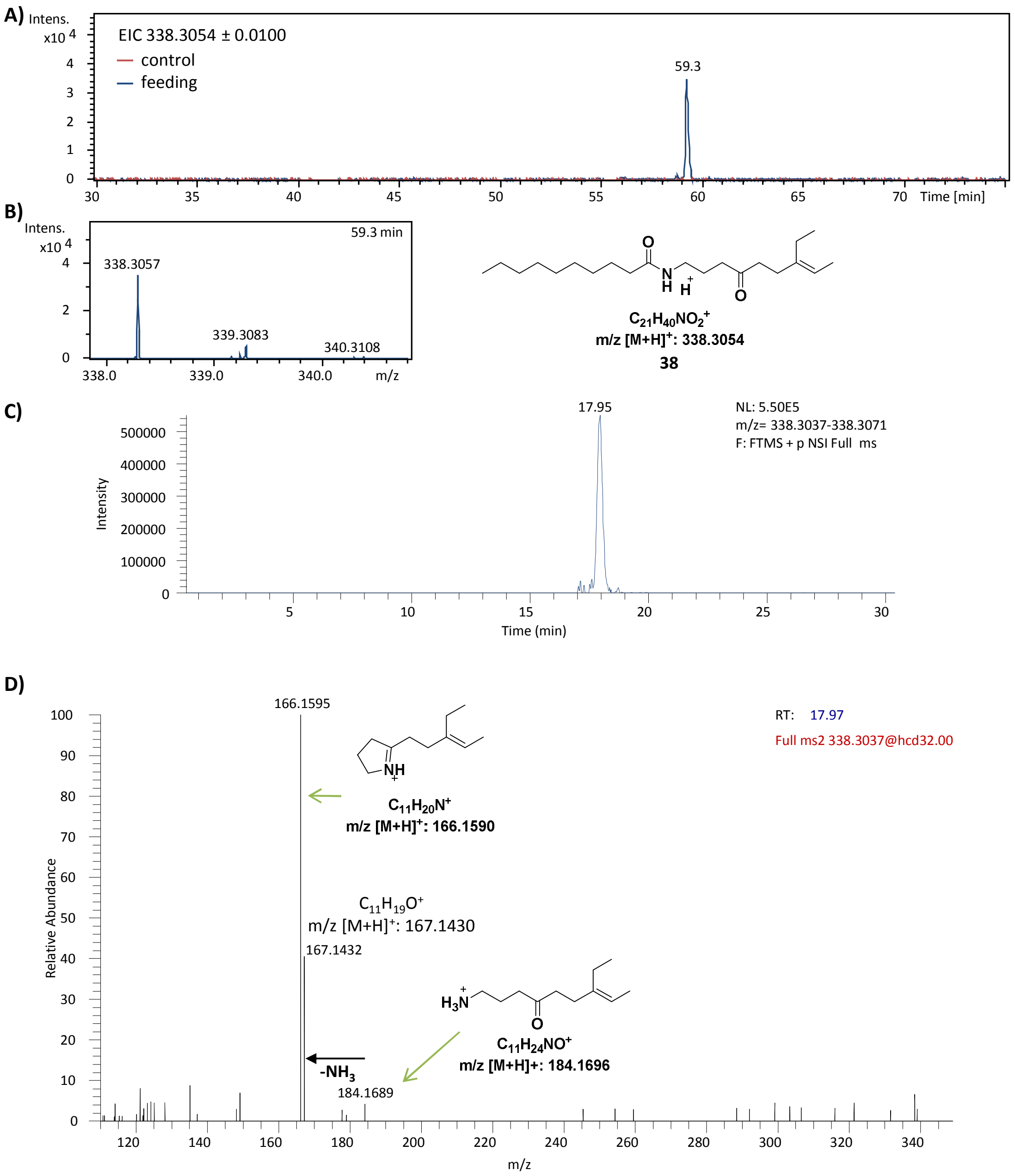


**Figure 9S:** (A) LC-HRMS analysis (MaXis Impact, method 2) of the organic extracts of *S. lasaliensis* ACP12 (S970A) grown in the absence (red) and in the presence (blue) of **3** (final concentration 4 mM): $[M+H]^+$ extracted ion chromatogram (EIC) and (B) high resolution mass are shown for the putative intermediate **38** (Rt = 59.3 min). (C) LC-HRMS analysis (Orbitrap Fusion) of the organic extracts of *S. lasaliensis* ACP12 (S970A) grown in the presence of **3** (final concentration 4 mM)**:** EIC (Rt = 17.95min) and (D) fragmentation of **38** with putative fragment structural assignment.

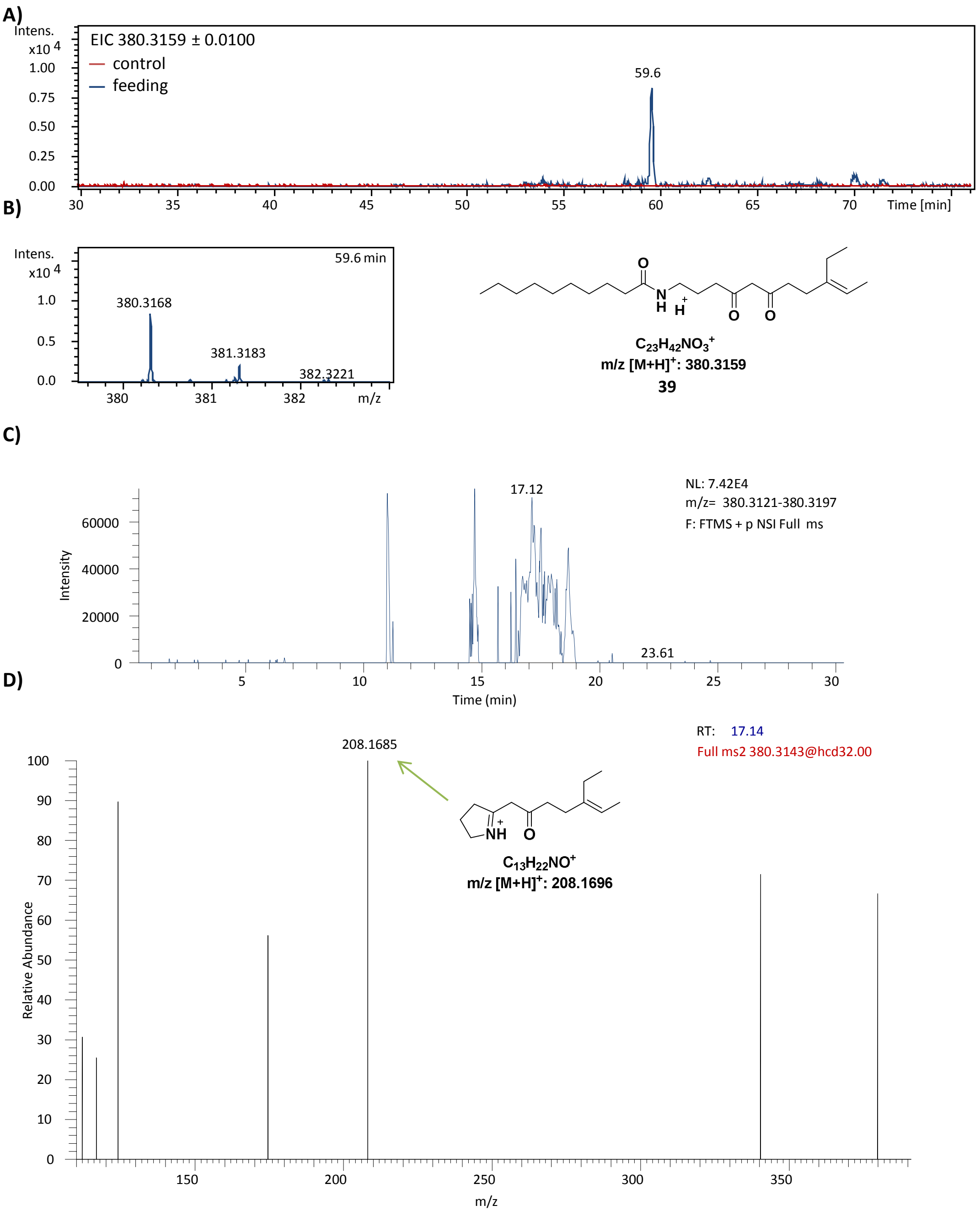


**Figure 10S:** (A) LC-HRMS analysis (MaXis Impact, method 2) of the organic extracts of *S. lasaliensis* ACP12 (S970A) grown in the absence (red) and in the presence (blue) of **3** (final concentration 4 mM): $[M+H]^+$ extracted ion chromatogram (EIC) and (B) high resolution mass are shown for the putative intermediate **39** (Rt = 59.6 min). (C) LC-HRMS analysis (Orbitrap Fusion) of the organic extracts of *S. lasaliensis* ACP12 (S970A) grown in the presence of **3** (final concentration 4 mM)**:** EIC (Rt = 17.12 min) and (D) fragmentation of **39** with putative fragment structural assignment.

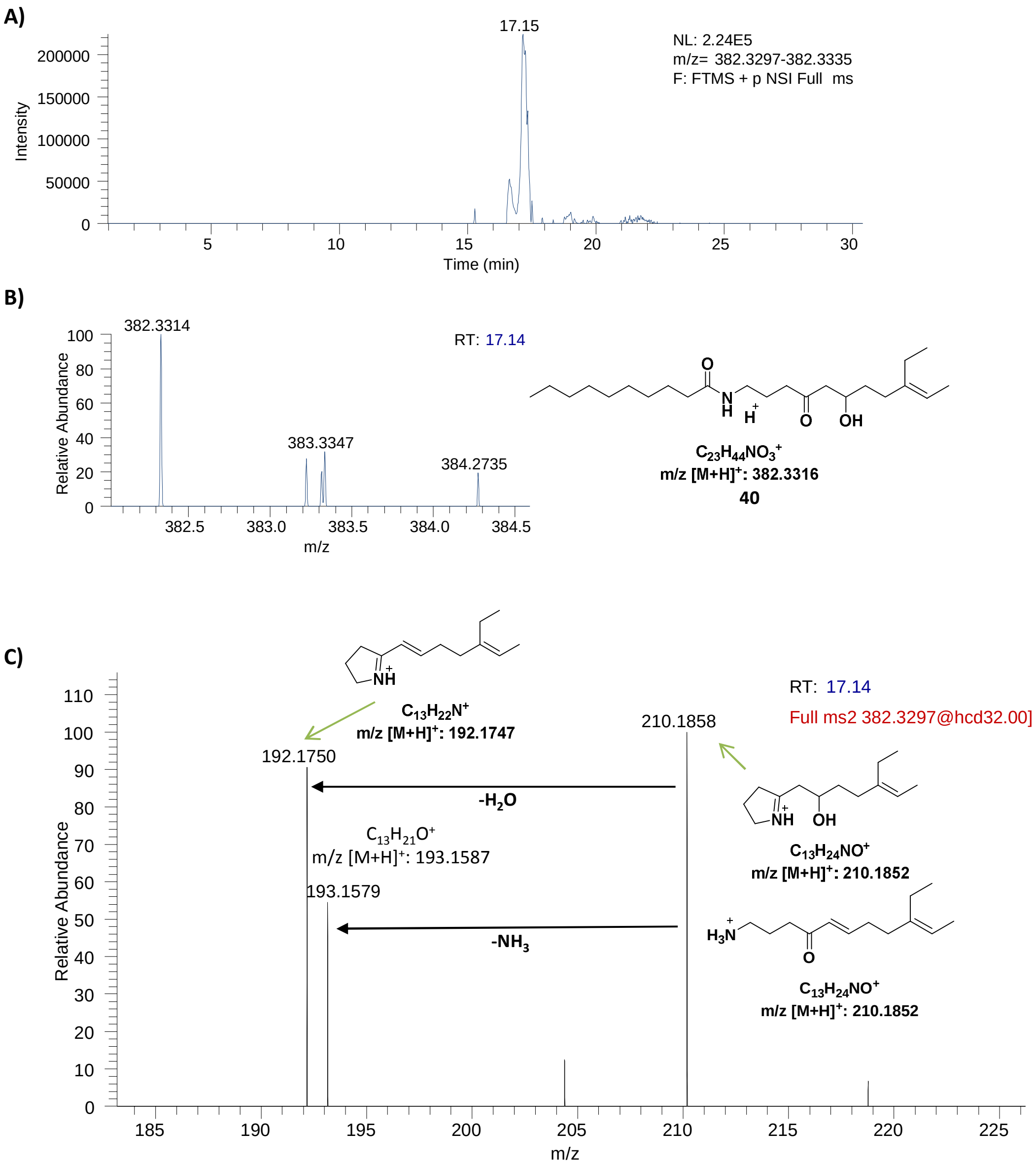


**Figure 11S:** (A) LC-HRMS analysis (Orbitrap Fusion) of the organic extracts of *S. lasaliensis* ACP12 (S970A) grown in the in the presence of **3** (final concentration 4 mM): $[M+H]^+$ extracted ion chromatogram (EIC) (Rt = 17.15 min), (B) high resolution mass and (C) fragmentation of putative intermediate **40** with putative fragment structural assignment are shown.

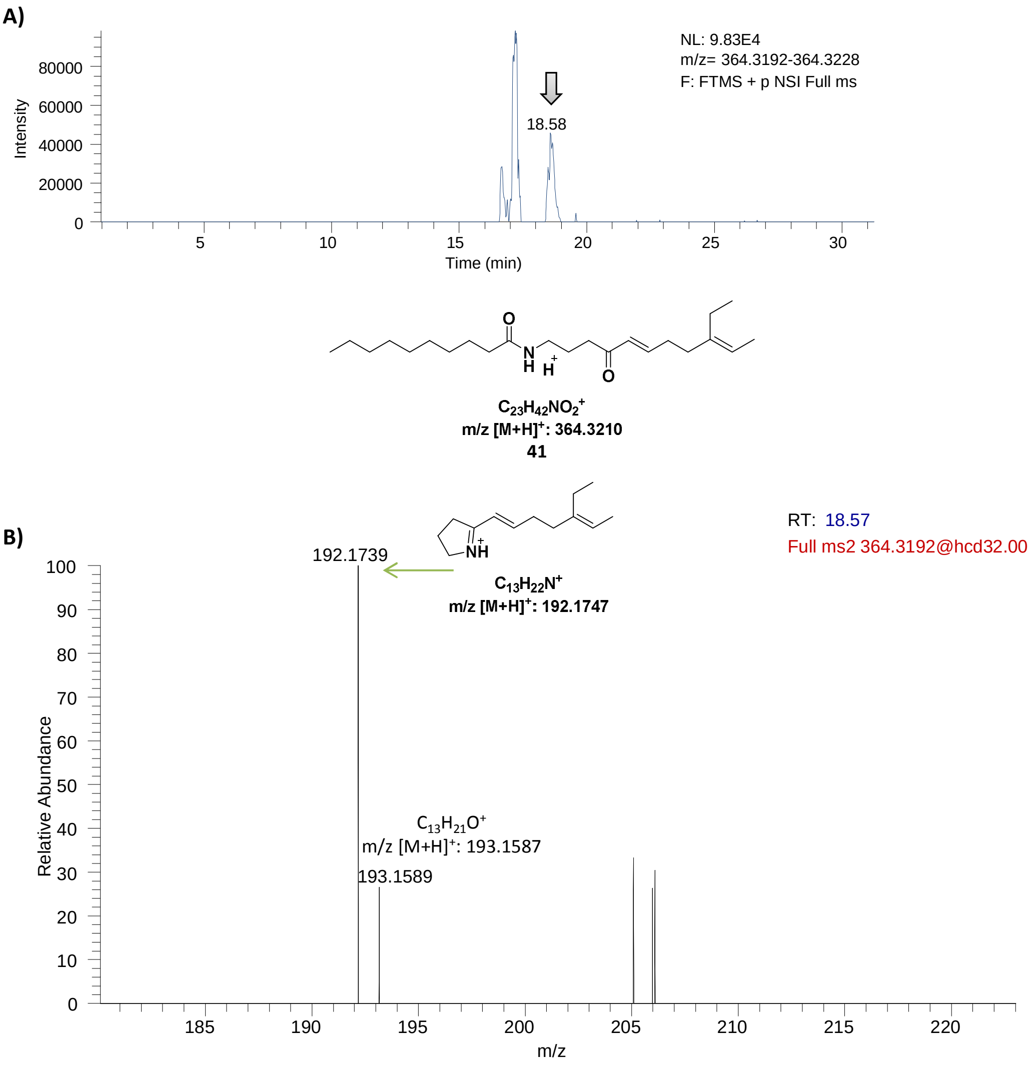


**Figure 12S:** A) LC-HRMS analysis (Orbitrap Fusion) of the organic extracts of *S. lasaliensis* ACP12 (S970A) grown in the presence of **3** (final concentration 4 mM)**:** EIC (Rt = 18.58 min) and (B) fragmentation of putative intermediate **41** with putative fragment structural assignment.

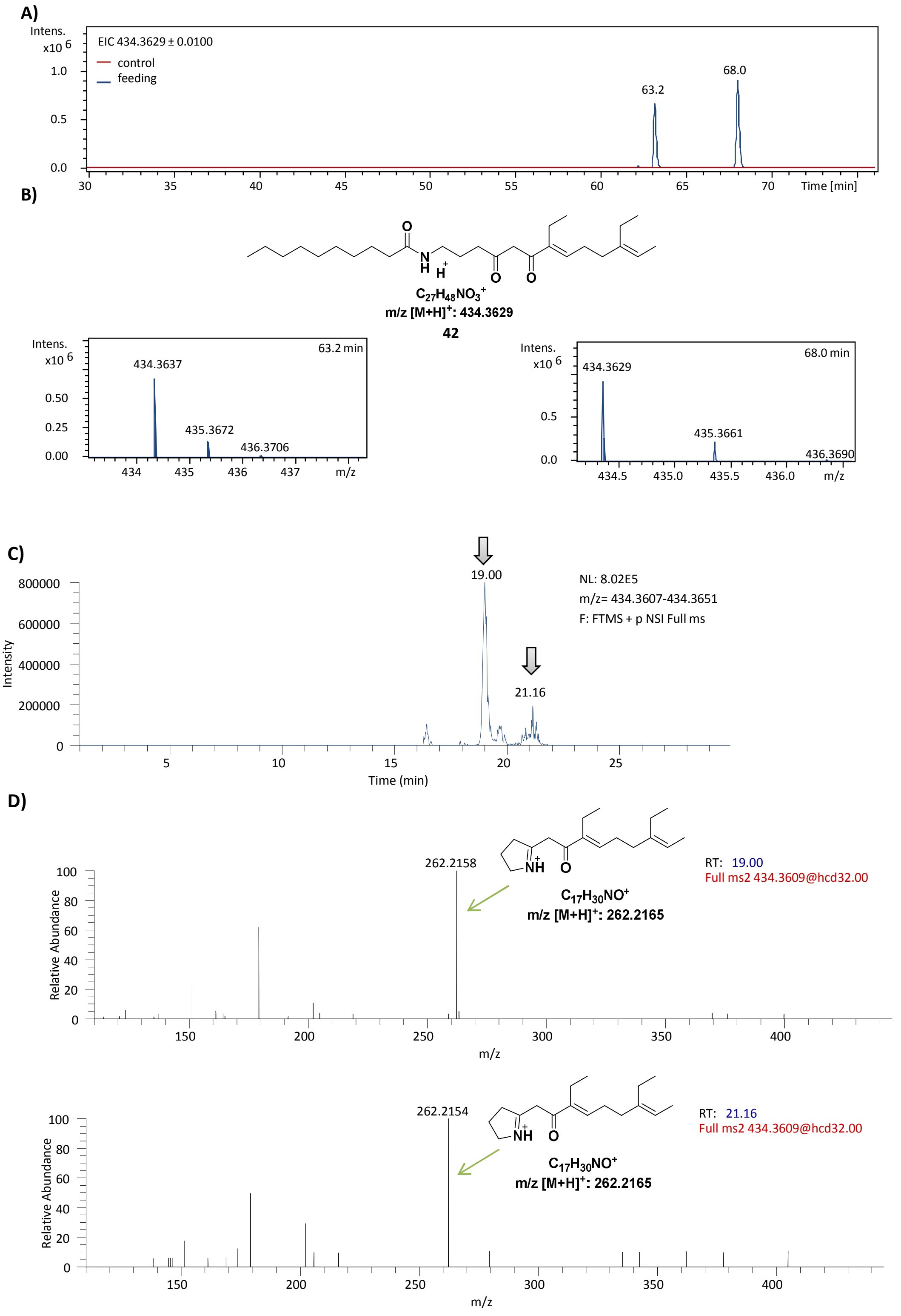


**Figure 13S:** (A) LC-HRMS analysis (MaXis Impact, method 2) of the organic extracts of *S. lasaliensis* ACP12 (S970A) grown in the absence (red) and in the presence (blue) of **3** (final concentration 4 mM): $[M+H]^+$ extracted ion chromatogram (EIC) and (B) high resolution mass are shown for the putative intermediate **42** (Rt = 63.2 and 68.0 min). (C) LC-HRMS analysis (Orbitrap Fusion) of the organic extracts of *S. lasaliensis* ACP12 (S970A) grown in the presence of **3** (final concentration 4 mM)**:** EIC (Rt = 19.00 and 21.16 min) and (D) fragmentation of **42** with putative fragment structural assignment. Double peaks may arise from isomerisation (currently under investigation).

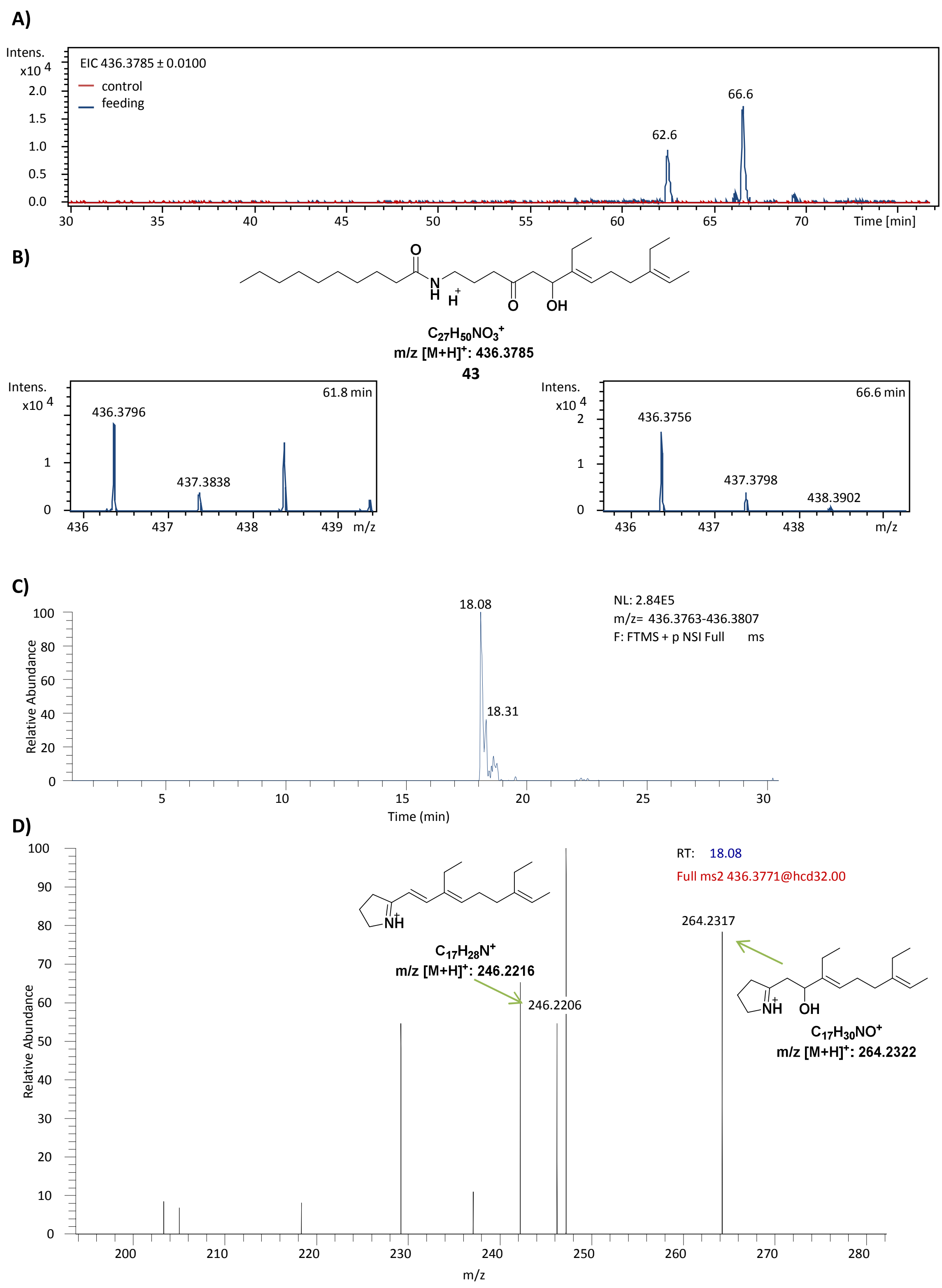


**Figure 14S:** (A) LC-HRMS analysis (MaXis Impact, method 2) of the organic extracts of *S. lasaliensis* ACP12 (S970A) grown in the absence (red) and in the presence (blue) of **3** (final concentration 4 mM): $[M+H]^+$ extracted ion chromatogram (EIC) and (B) high resolution mass are shown for the putative intermediate **43** (Rt = 62.6 and 66.6 min). (C) LC-HRMS analysis (Orbitrap Fusion) of the organic extracts of *S. lasaliensis* ACP12 (S970A) grown in the presence of **3** (final concentration 4 mM)**:** EIC (Rt = 18.08 and 18.31 min) and (D) fragmentation of **43** with putative fragment structural assignment. Double peaks may arise from isomerisation (currently under investigation).

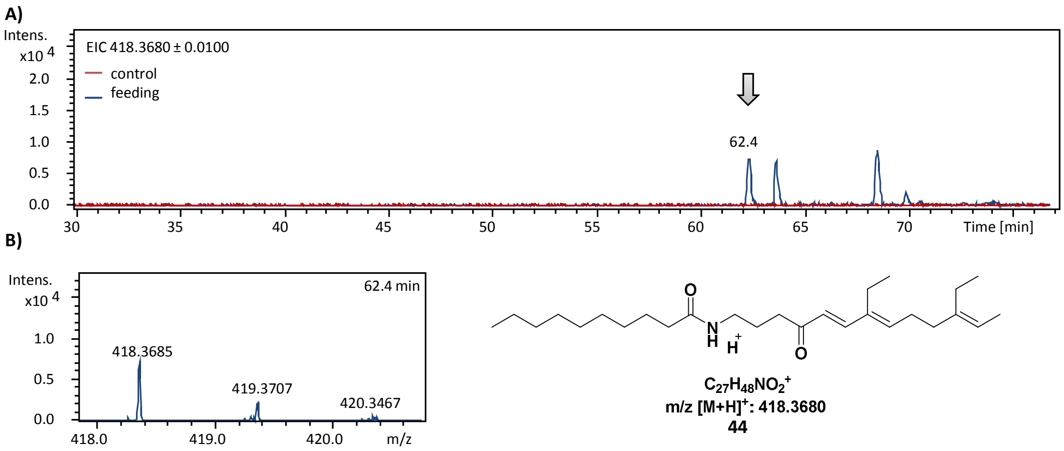


**Figure 15S:** (A) LC-HRMS analysis (MaXis Impact, method 2) of the organic extracts of *S. lasaliensis* ACP12 (S970A) grown in the absence (red) and in the presence (blue) of **3** (final concentration 4 mM): $[M+H]^+$ extracted ion chromatogram (EIC) and (B) high resolution mass are shown for the putative intermediate **44** (Rt = 62.4 min).

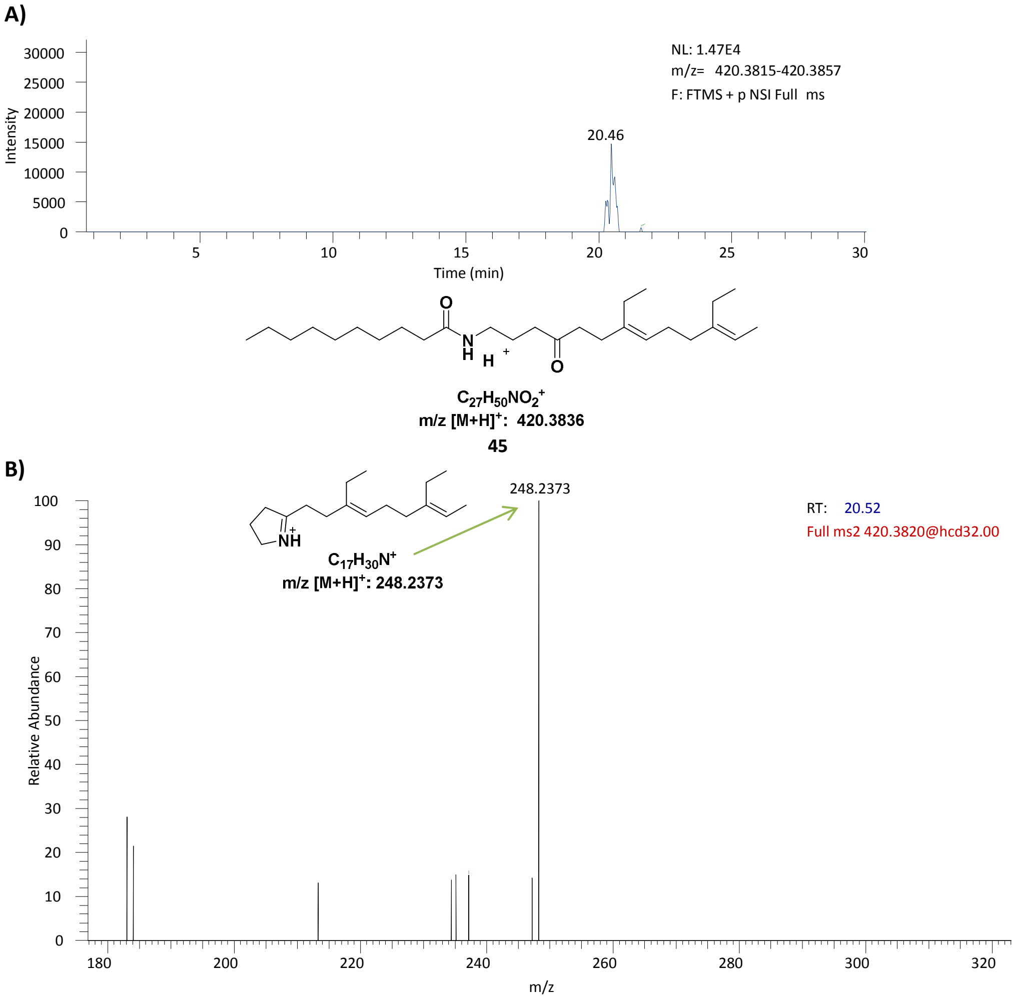


**Figure 16S:** A) LC-HRMS analysis (Orbitrap Fusion) of the organic extracts of *S. lasaliensis* ACP12 (S970A) grown in the presence of **3** (final concentration 4 mM)**:** EIC (Rt = 20.46 min) and (B) fragmentation of putative intermediate **45** with putative fragment structural assignment.

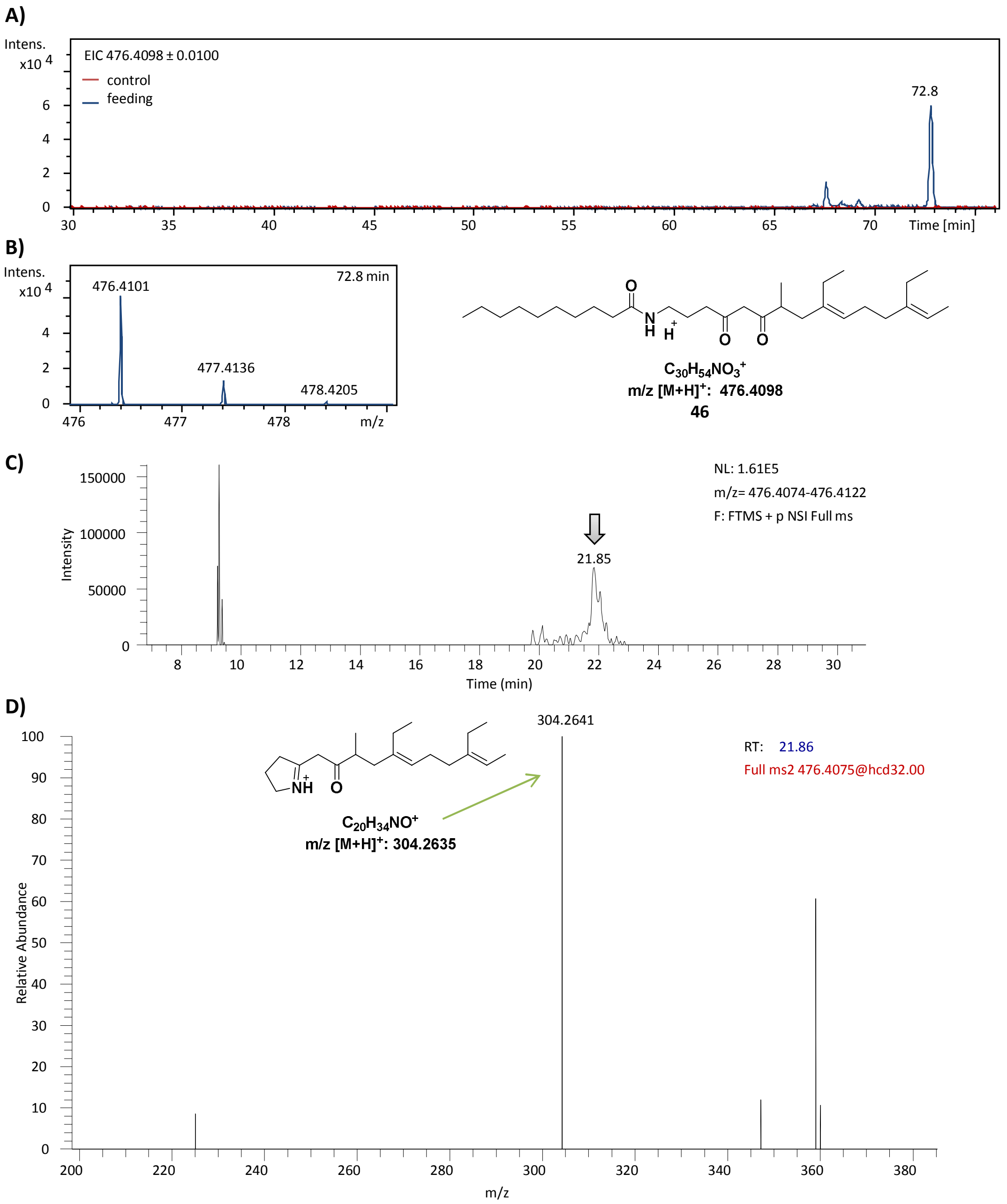


**Figure 17S:** (A) LC-HRMS analysis (MaXis Impact, method 2) of the organic extracts of *S. lasaliensis* ACP12 (S970A) grown in the absence (red) and in the presence (blue) of **3** (final concentration 4 mM): $[M+H]^+$ extracted ion chromatogram (EIC) and (B) high resolution mass are shown for the putative intermediate **46** (Rt = 72.8 min). (C) LC-HRMS analysis (Orbitrap Fusion) of the organic extracts of *S. lasaliensis* ACP12 (S970A) grown in the presence of **3** (final concentration 4 mM)**:** EIC (Rt = 21.85 min) and (D) fragmentation of **46** with putative fragment structural assignment.

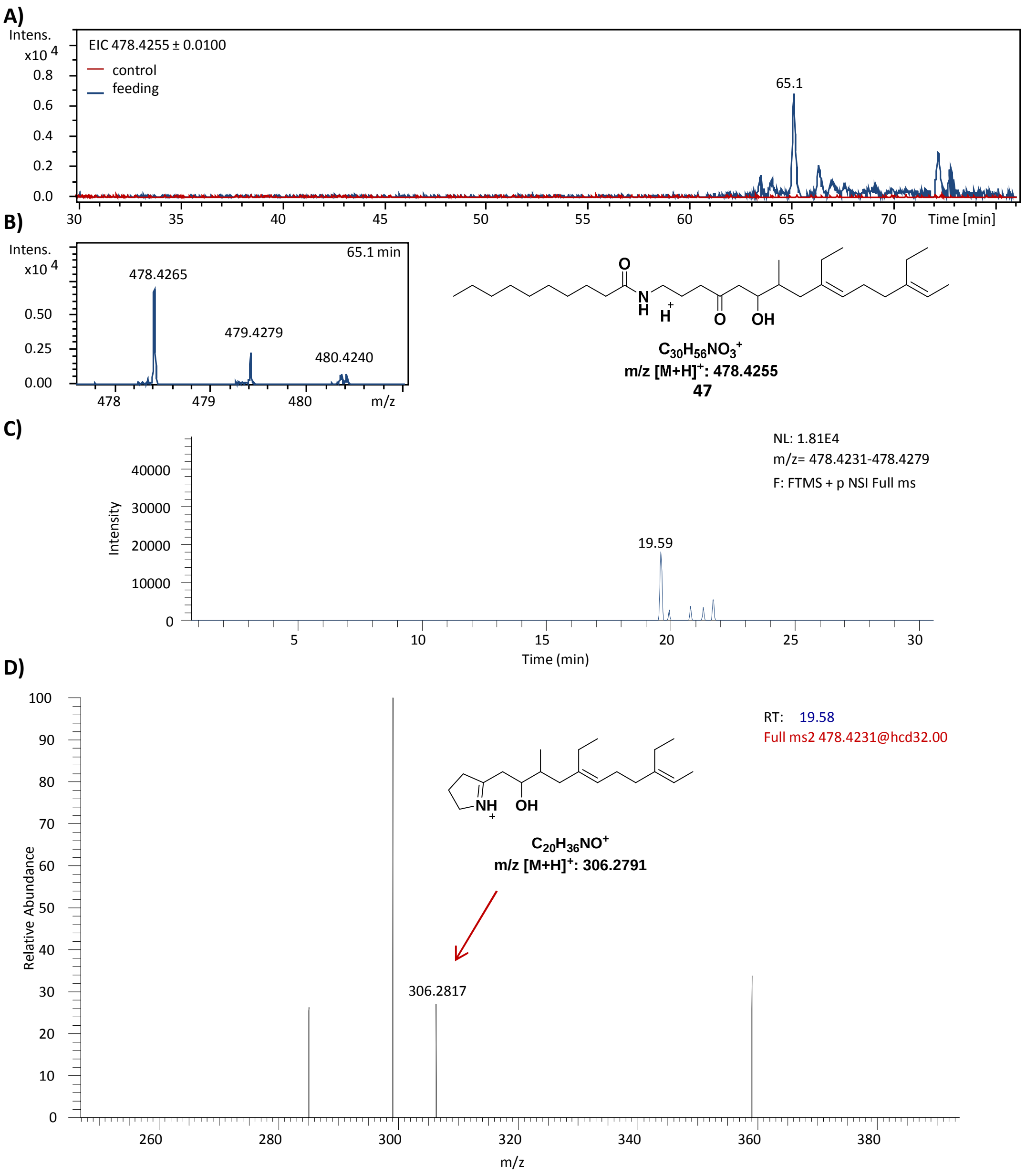


**Figure 18S:** (A) LC-HRMS analysis (MaXis Impact, method 2) of the organic extracts of *S. lasaliensis* ACP12 (S970A) grown in the absence (red) and in the presence (blue) of **3** (final concentration 4 mM): $[M+H]^+$ extracted ion chromatogram (EIC) and (B) high resolution mass are shown for the putative intermediate **47** (Rt = 65.1 min). (C) LC-HRMS analysis (Orbitrap Fusion) of the organic extracts of *S. lasaliensis* ACP12 (S970A) grown in the presence of **3** (final concentration 4 mM)**:** EIC (Rt = 19.59 min) and (D) fragmentation of **47** with putative fragment structural assignment.

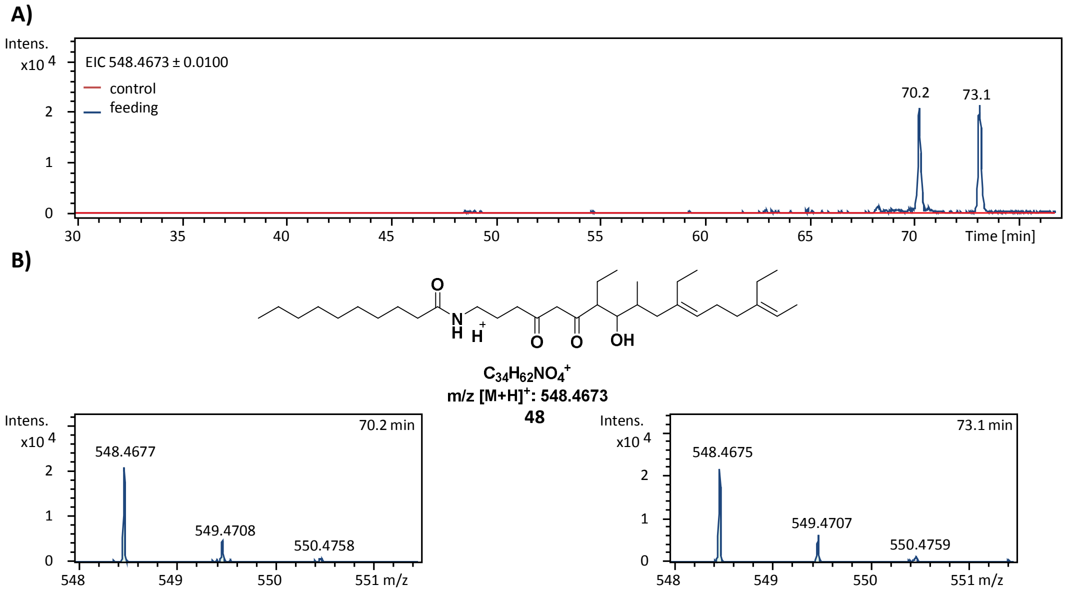


**Figure 19S:** (A) LC-HRMS analysis (MaXis Impact, method 2) of the organic extracts of *S. lasaliensis* ACP12 (S970A) grown in the absence (red) and in the presence (blue) of **3** (final concentration 4 mM): $[M+H]^+$ extracted ion chromatogram (EIC) and (B) high resolution mass are shown for the putative intermediate **48** (Rt = 70.2 and 73.1 min). Double peaks may arise from intramolecular cyclisation or isomerisation (currently under investigation).

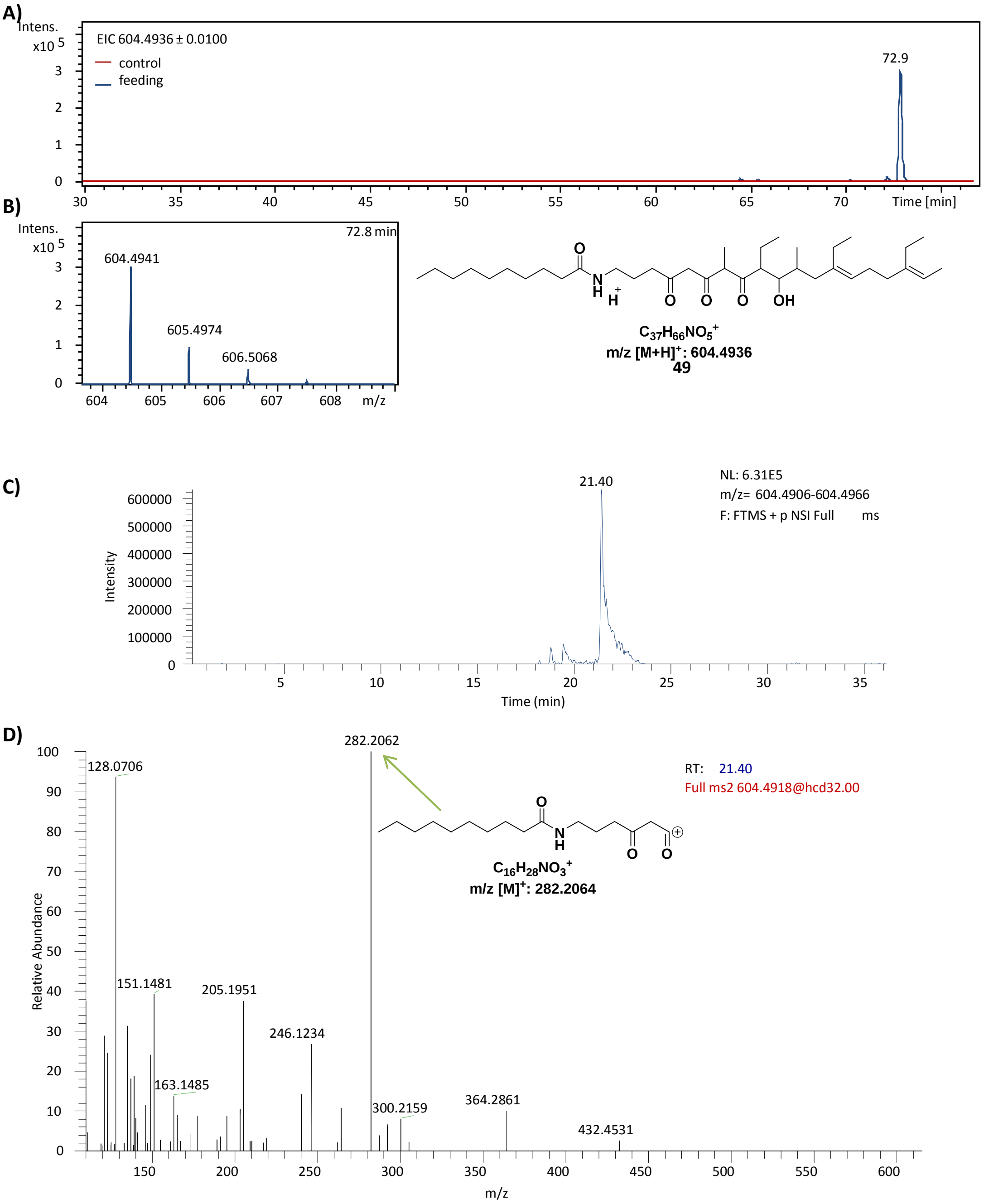


**Figure 20S:** (A) LC-HRMS analysis (MaXis Impact, method 2) of the organic extracts of *S. lasaliensis* ACP12 (S970A) grown in the absence (red) and in the presence (blue) of **3** (final concentration 4 mM): $[M+H]^+$ extracted ion chromatogram (EIC) and (B) high resolution mass are shown for the putative intermediate **49** (Rt = 72.9 min). (C) LC-HRMS analysis (Orbitrap Fusion) of the organic extracts of *S. lasaliensis* ACP12 (S970A) grown in the presence of **3** (final concentration 4 mM): EIC (Rt = 21.40 min) and (D) fragmentation of **49** with putative fragment structural assignment. A compound of *m/z* [M+H]= 586.4830 ($C_{37}H_{64}NO_4^+$) resulting from the loss of water from **49** was also detected at an identical retention time (and characterised by the same fragment highlighted in D)).

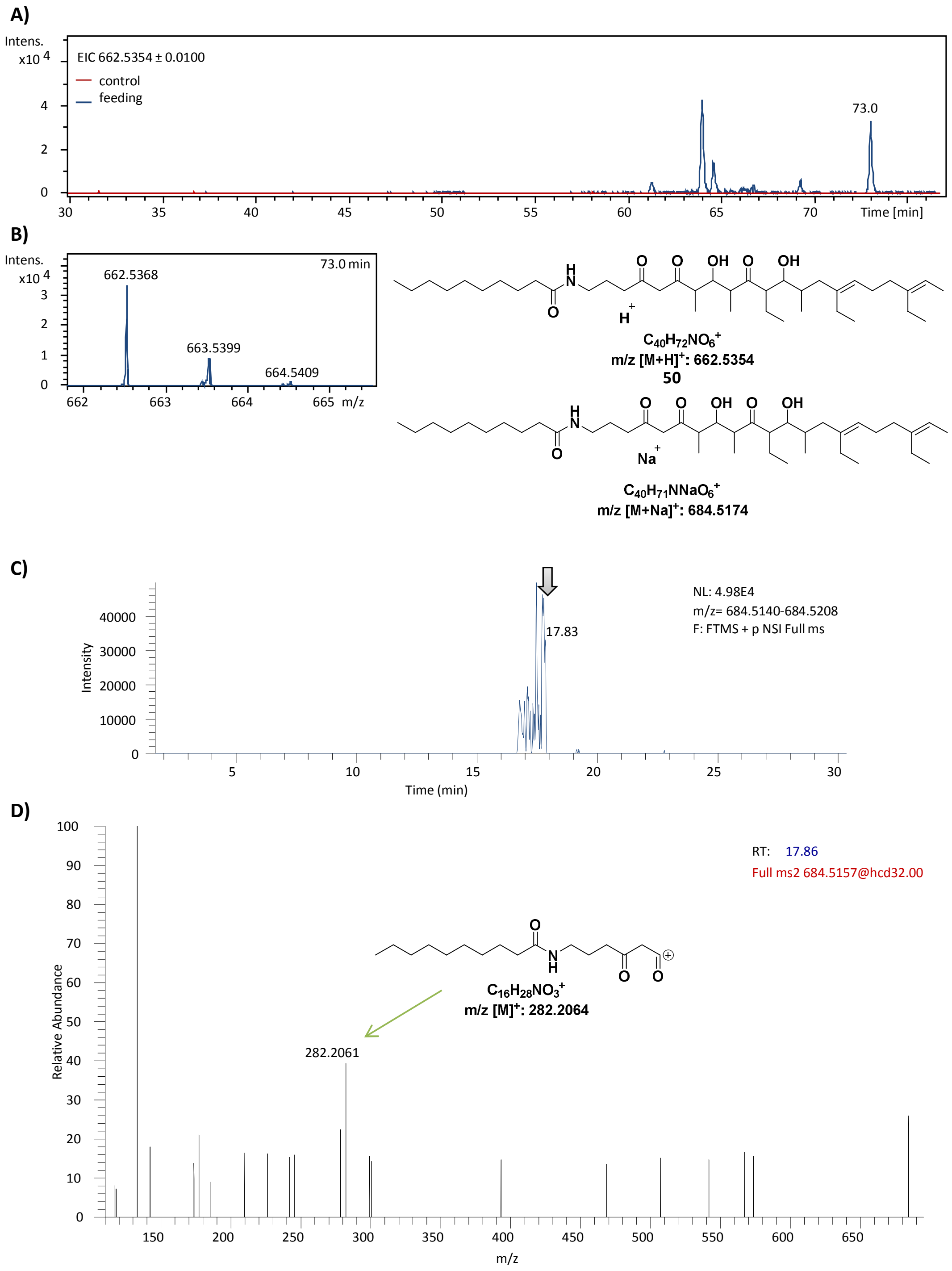


**Figure 21S:** (A) LC-HRMS analysis (MaXis Impact, method 2) of the organic extracts of *S. lasaliensis* ACP12 (S970A) grown in the absence (red) and in the presence (blue) of **3** (final concentration 4 mM): $[M+H]^+$ extracted ion chromatogram (EIC) and (B) high resolution mass are shown for the putative intermediate **50** (Rt = 73.0 min). (C) LC-HRMS analysis (Orbitrap Fusion) of the organic extracts of *S. lasaliensis* ACP12 (S970A) grown in the presence of **3** (final concentration 4 mM)**:** EIC (Rt = 17.83 min) and (D) fragmentation of **50** with putative fragment structural assignment.

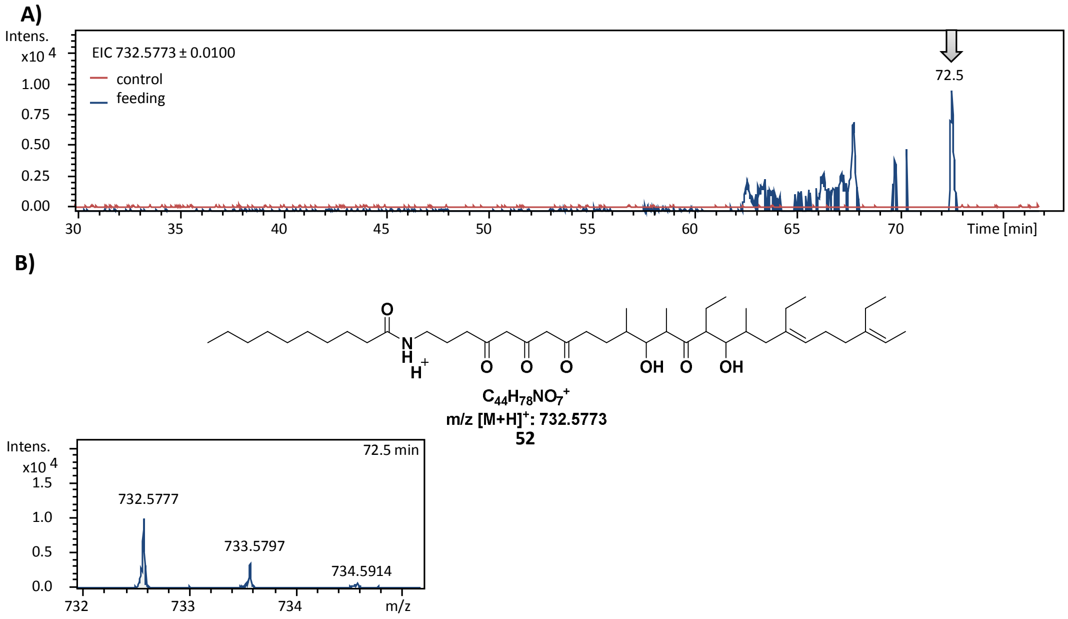


**Figure 22S:** (A) LC-HRMS analysis (MaXis Impact, method 2) of the organic extracts of *S. lasaliensis* ACP12 (S970A) grown in the absence (red) and in the presence (blue) of **3** (final concentration 4 mM): $[M+H]^+$ extracted ion chromatogram (EIC) and (B) high resolution mass are shown for the putative intermediate **52** (Rt = 72.5 min).

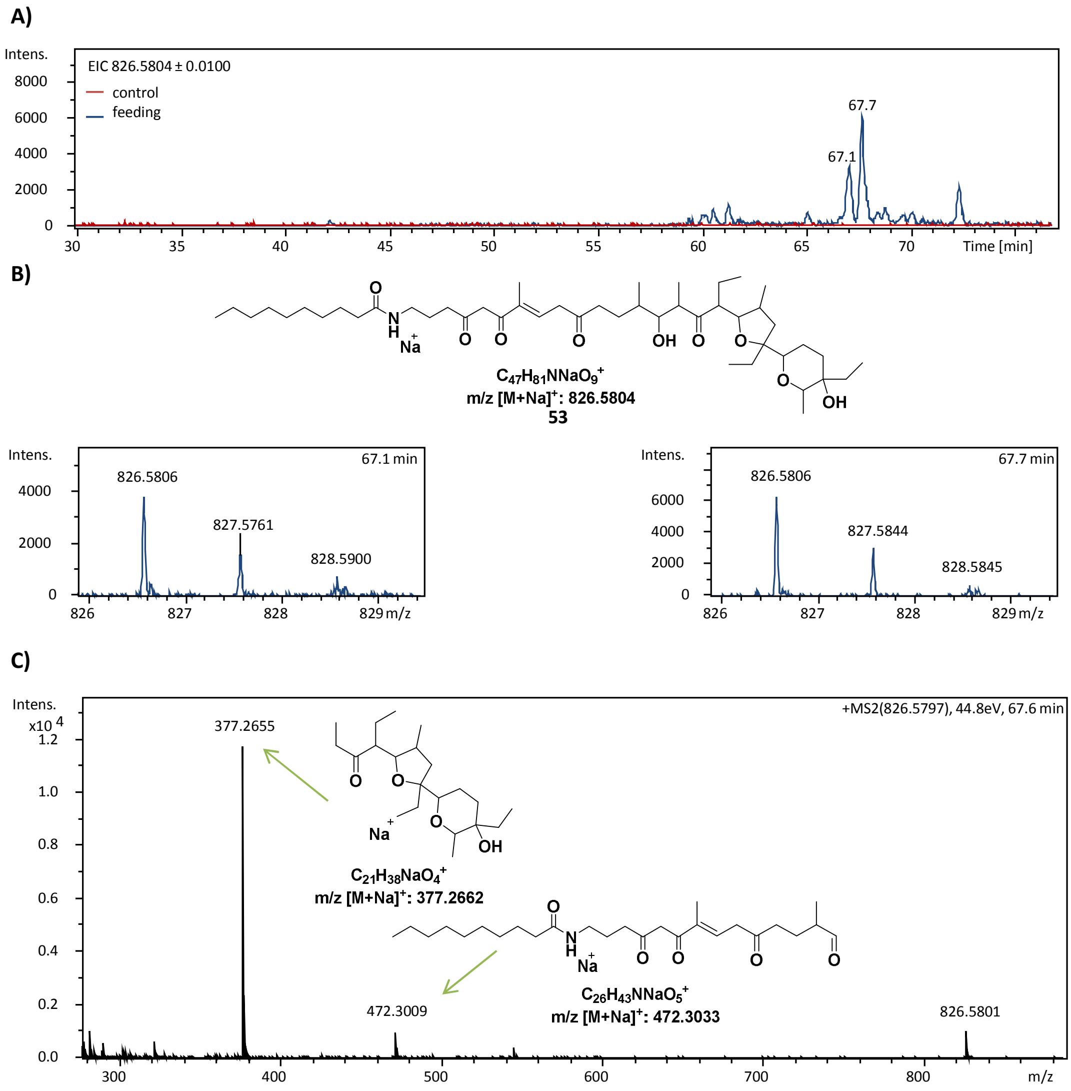


**Figure 23S:** (A) LC-HRMS analysis (MaXis Impact, method 2) of the organic extracts of *S. lasaliensis* ACP12 (S970A) grown in the absence (red) and in the presence (blue) of **3** (final concentration 4 mM): $[M+H]^+$ extracted ion chromatogram (EIC), (B) high resolution mass are shown for the putative intermediate **53** (Rt = 67.1 and 67.7 min) and (C) fragmentation of **53** with putative fragment structural assignment. Double peaks may arise from intramolecular cyclisation or isomerisation (currently under investigation).

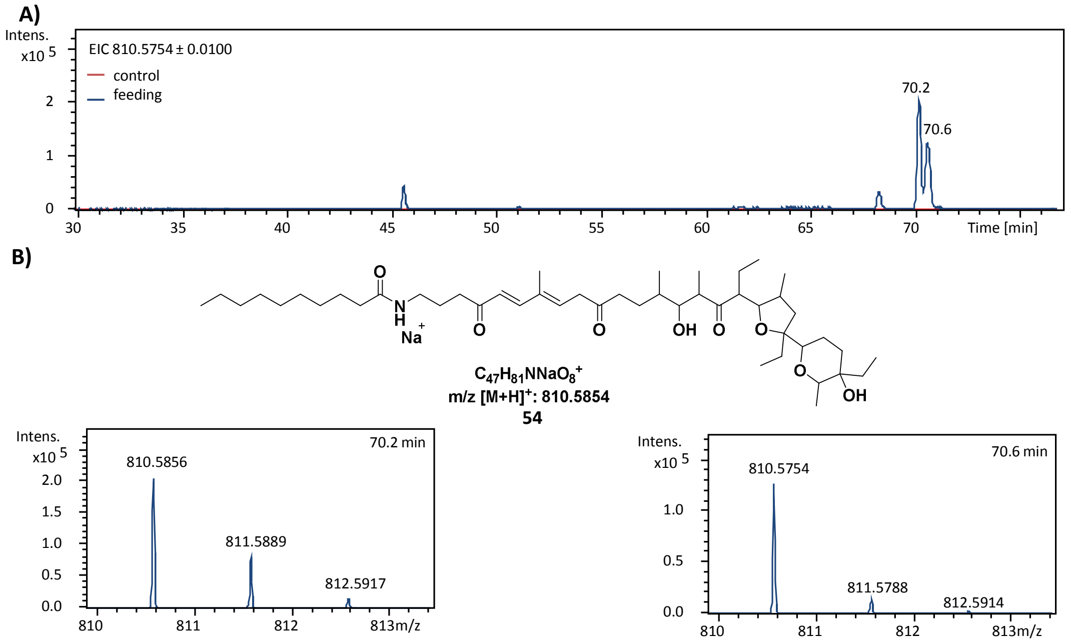


**Figure 24S:** (A) LC-HRMS analysis (MaXis Impact, method 2) of the organic extracts of *S. lasaliensis* ACP12 (S970A) grown in the absence (red) and in the presence (blue) of **3** (final concentration 4 mM): $[M+H]^+$ extracted ion chromatogram (EIC) and (B) high resolution mass are shown for the putative intermediate **54** (Rt = 70.2 and 70.6 min). Double peaks may arise from intramolecular cyclisation or isomerisation (currently under investigation).

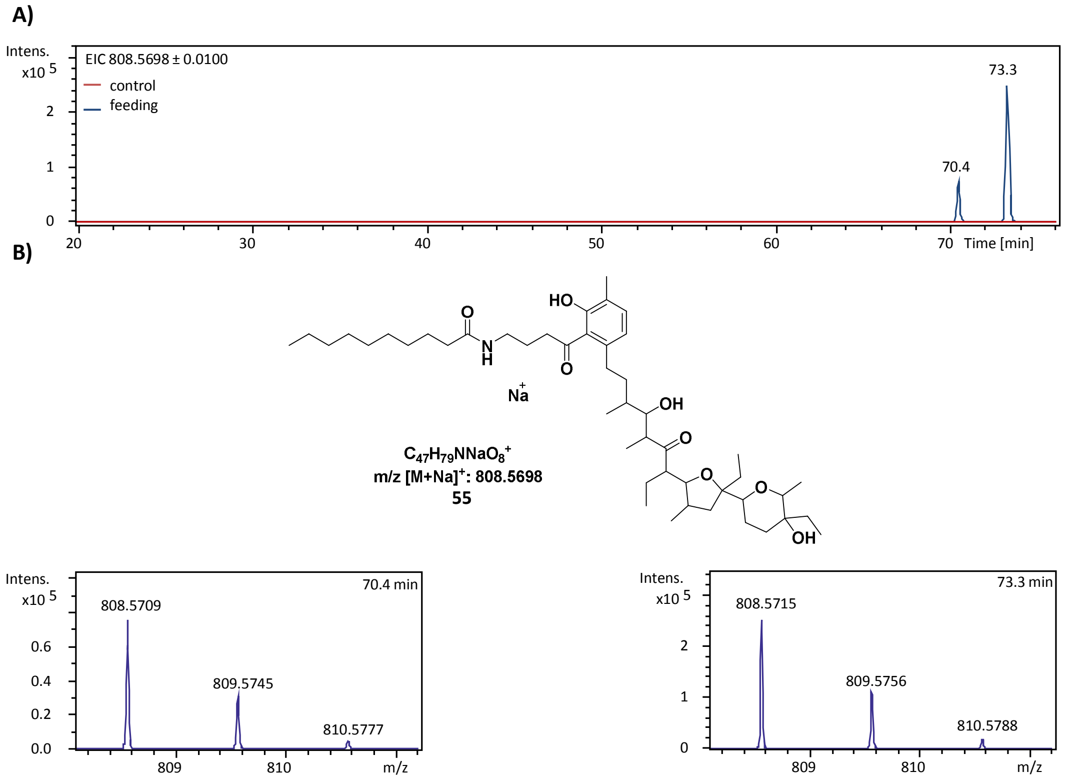

**C)**

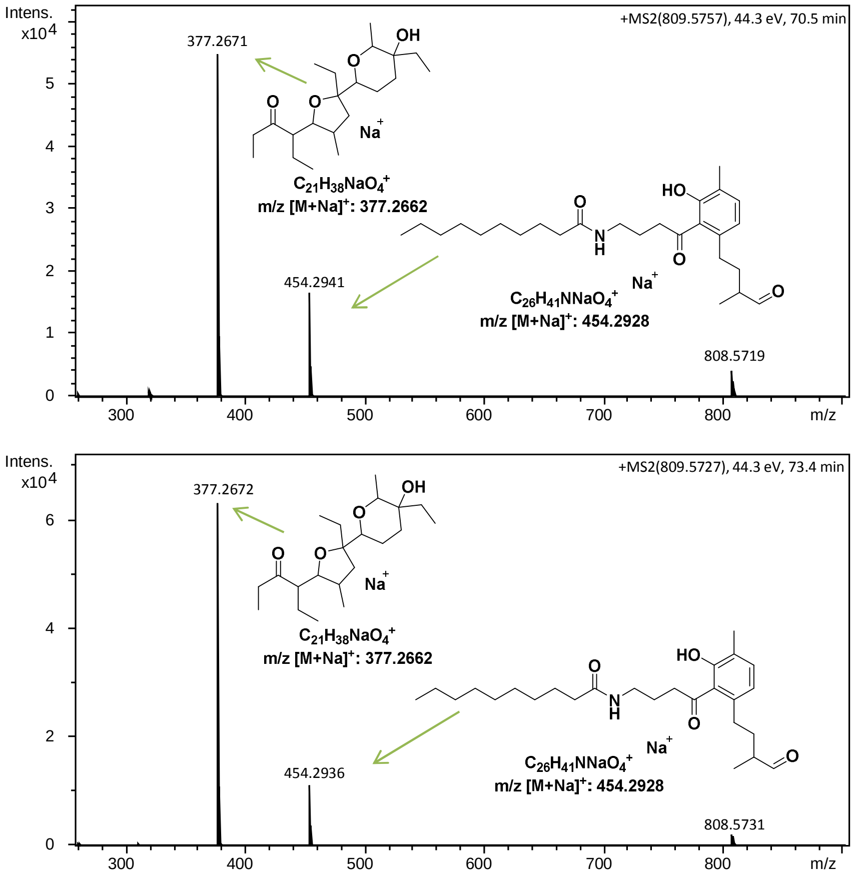


**Figure 25S:** (A) LC-HRMS analysis (MaXis Impact, method 2) of the organic extracts of *S. lasaliensis* ACP12 (S970A) grown in the absence (red) and in the presence (blue) of **3** (final concentration 4 mM): $[M+H]^+$ extracted ion chromatogram (EIC), (B) high resolution mass are shown for the putative intermediate **55** (Rt = 70.5 and 73.4 min) and (C) fragmentation of **55** with putative fragment structural assignment. Double peaks may arise from intramolecular cyclisation or isomerisation (currently under investigation).

## 2.3. Intermediate capture by *N*-(4,6-dioxoheptyl)decanamide (20)

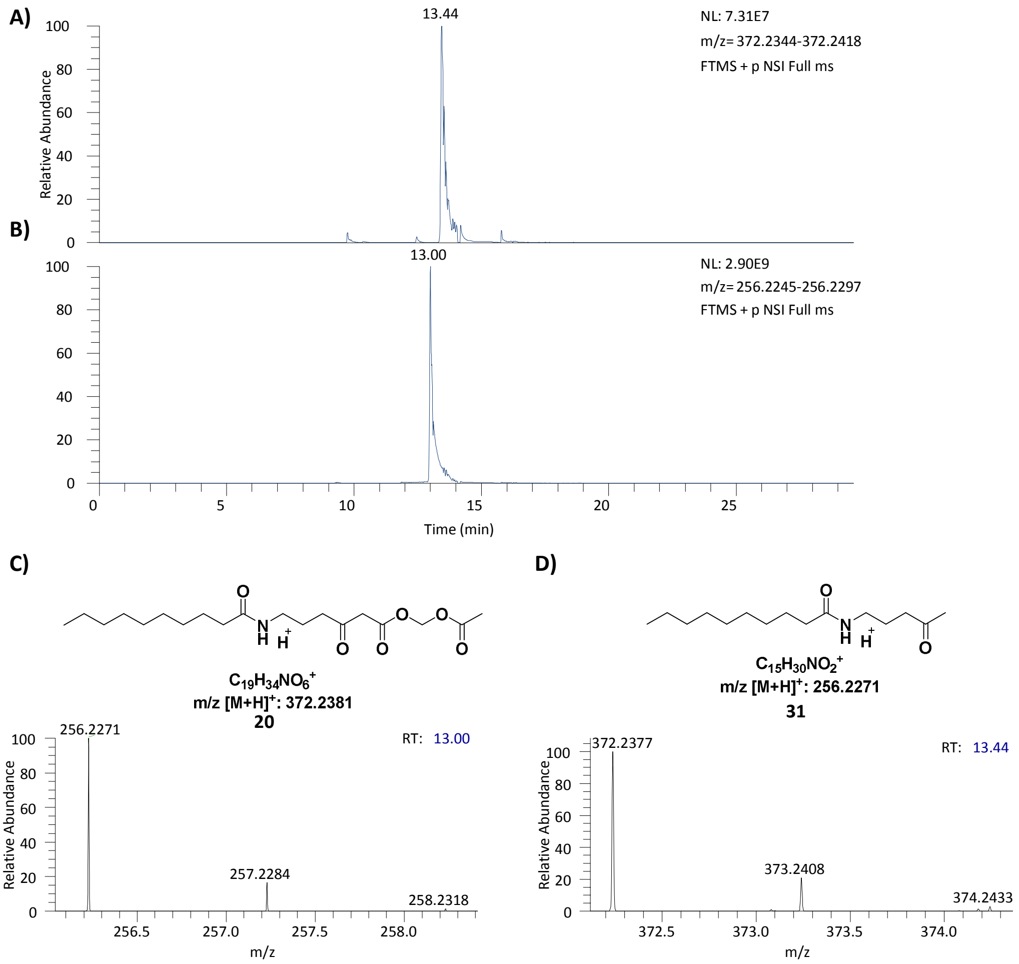


**Figure 26S:** (A) LC-HRMS analysis (Orbitrap Fusion) of the organic extracts of *S. lasaliensis* ACP12 (S970A) grown in the in the presence of **20** (final concentration 4 mM): $[M+H]^+$ extracted ion chromatogram (EIC) for probe **20** (Rt = 13.44 min) and (B) EIC for the decarboxylated probe **31** (Rt = 13.00 min) are shown. (C) The high resolution masses of probe **20** and (D) of the hydrolysed- decarboxylated probe **31** are shown.

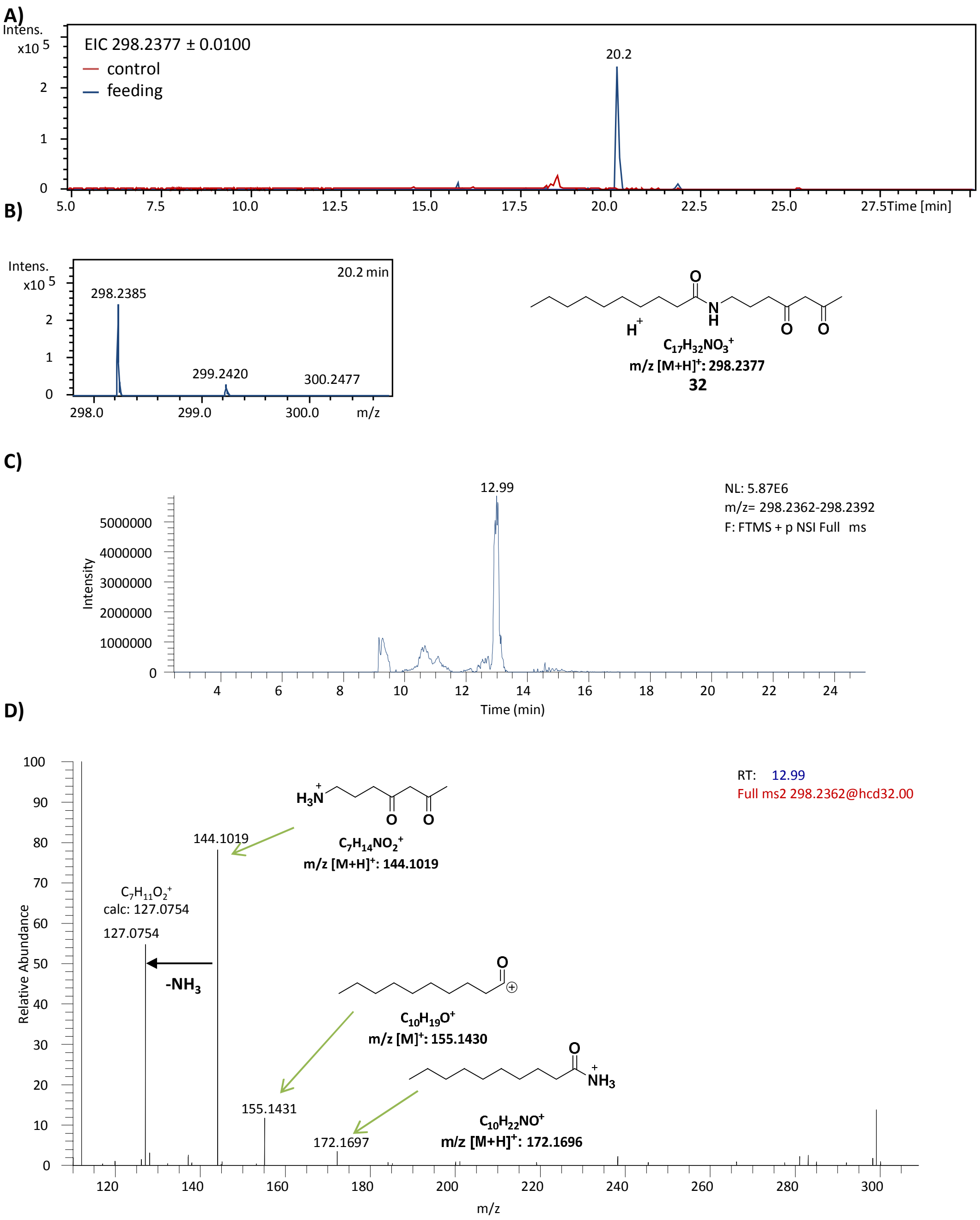


**Figure 27S:** (A) LC-HRMS analysis (MaXis Impact, method 2) of the organic extracts of *S. lasaliensis* ACP12 (S970A) grown in the absence (red) and in the presence (blue) of **20** (final concentration 4 mM): $[M+H]^+$ extracted ion chromatogram (EIC) and (B) high resolution mass are shown for the putative intermediate **32** (Rt = 20.2 min). (C) LC-HRMS analysis (Orbitrap Fusion) of the organic extracts of *S. lasaliensis* ACP12 (S970A) grown in the presence of **20** (final concentration 4 mM): EIC (Rt = 12.99 min) and (D) fragmentation of **32** with putative fragment structural assignment.

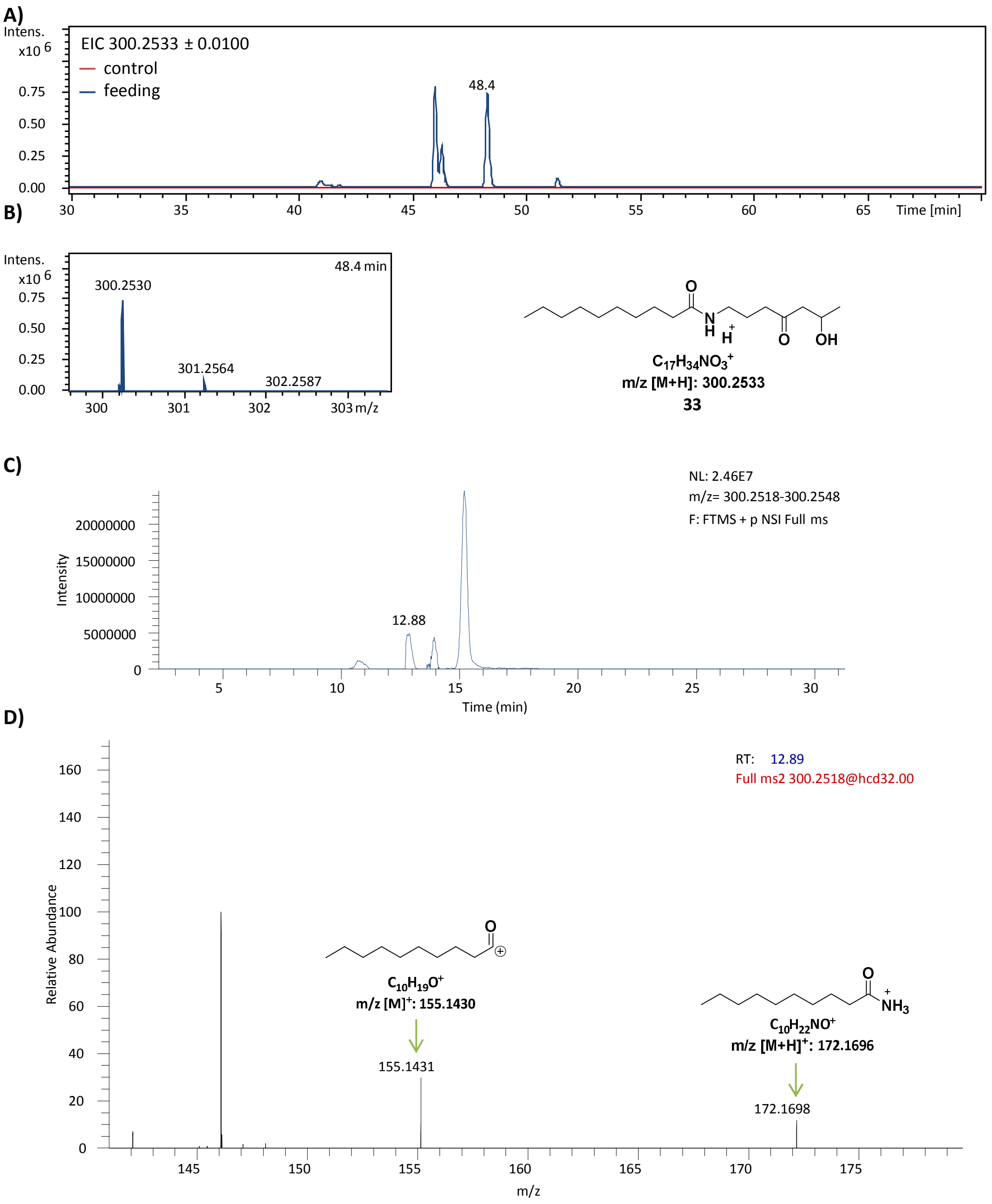


**Figure 28S:** (A) LC-HRMS analysis (MaXis Impact, method 2) of the organic extracts of *S. lasaliensis* ACP12 (S970A) grown in the absence (red) and in the presence (blue) of **20** (final concentration 4 mM): $[M+H]^+$ extracted ion chromatogram (EIC) and (B) high resolution mass are shown for the putative intermediate **33** (Rt = 48.4 min). (C) LC-HRMS analysis (Orbitrap Fusion) of the organic extracts of *S. lasaliensis* ACP12 (S970A) grown in the presence of **20** (final concentration 4 mM)**:** EIC (Rt = 12.88 min) and (D) fragmentation of **33** with putative fragment structural assignment.

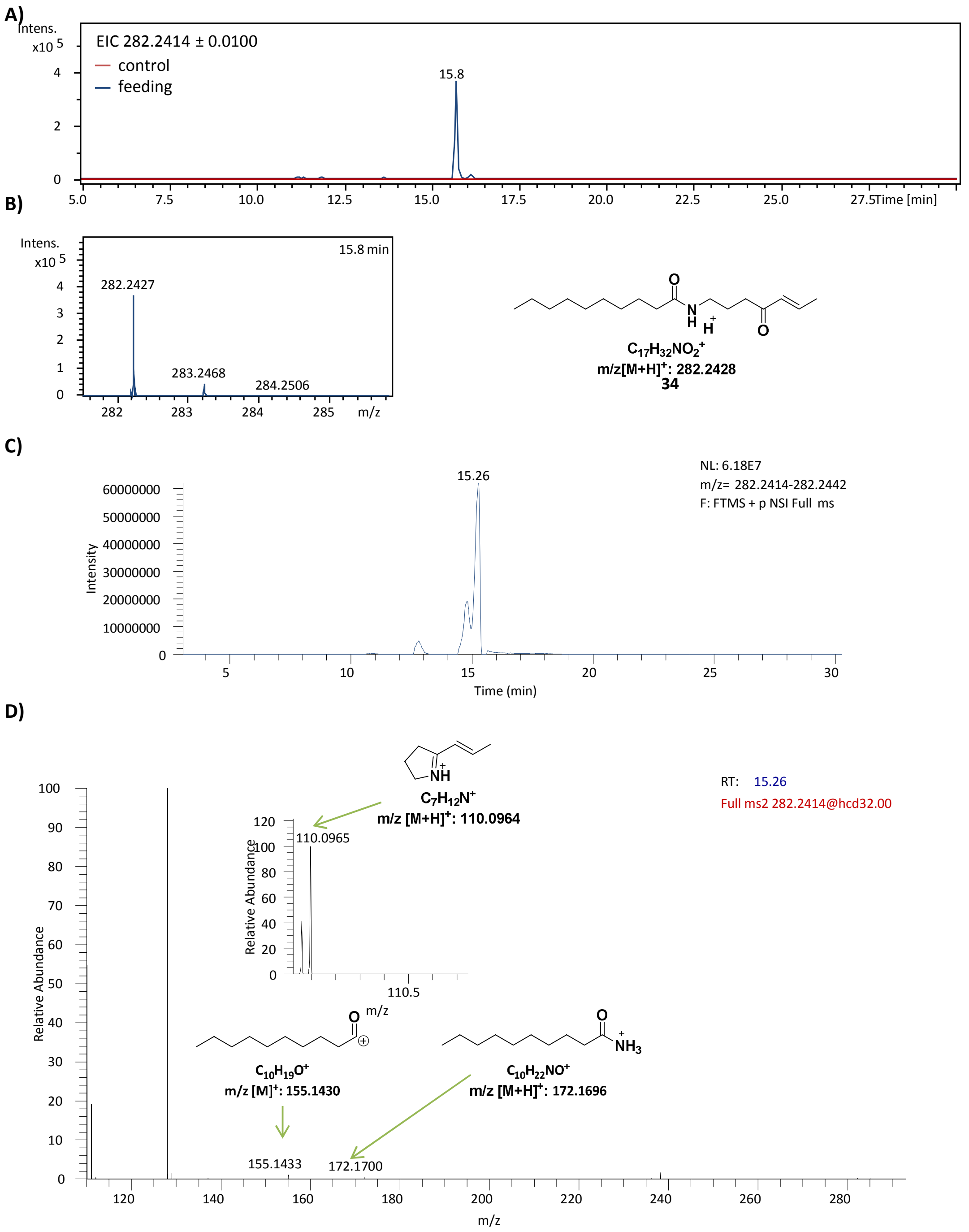


**Figure 29S:** A) LC-HRMS analysis (MaXis Impact, method 2) of the organic extracts of *S. lasaliensis* ACP12 (S970A) grown in the absence (red) and in the presence (blue) of **20** (final concentration 4 mM): $[M+H]^+$ extracted ion chromatogram (EIC) and (B) high resolution mass are shown for the putative intermediate **34** (Rt = 15.8 min). (C) LC-HRMS analysis (Orbitrap Fusion) of the organic extracts of *S. lasaliensis* ACP12 (S970A) grown in the presence of **20** (final concentration 4 mM)**:** EIC (Rt = 15.26 min) and (D) fragmentation of **34** with putative fragment structural assignment.

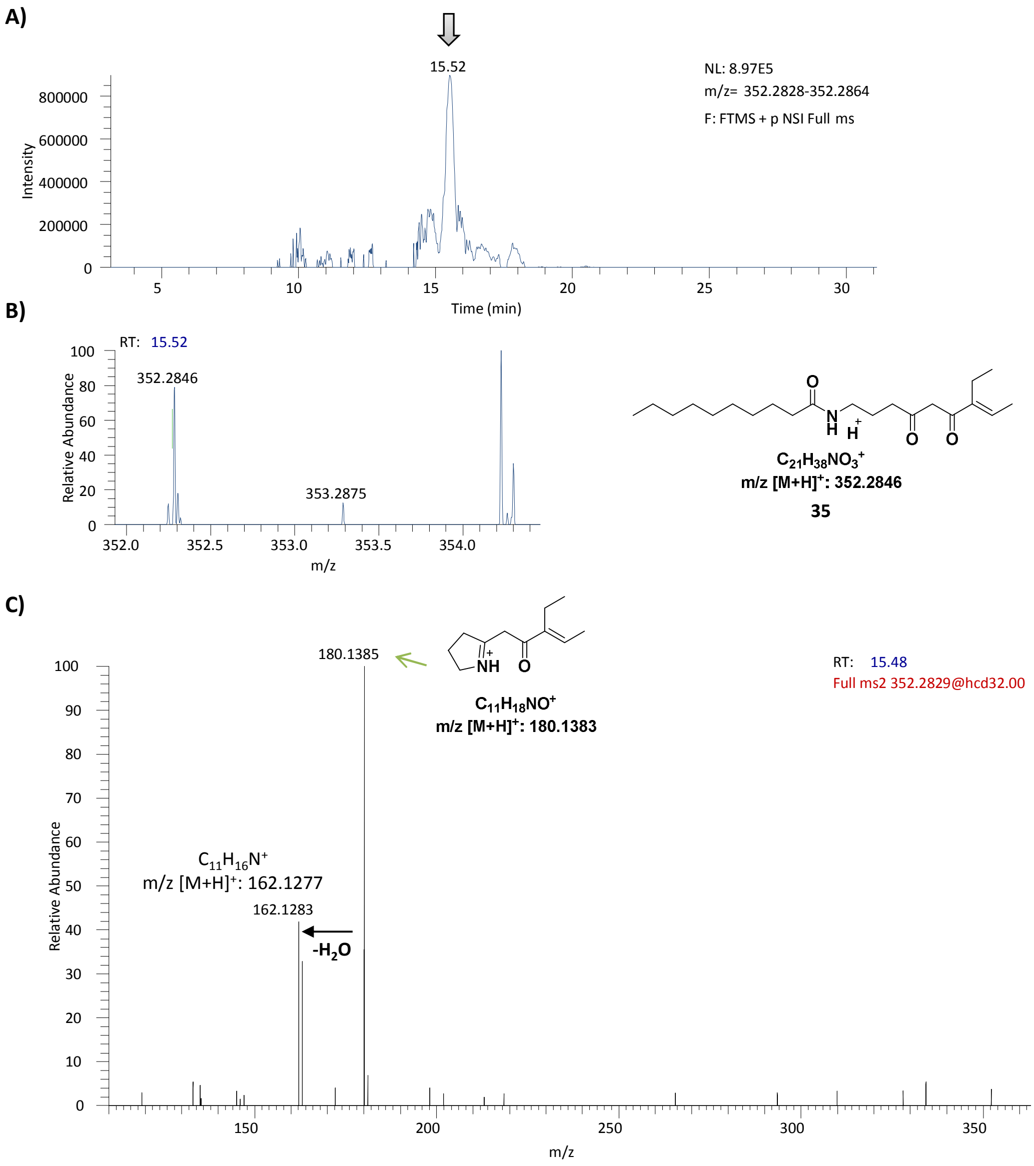


**Figure 30S:** (A) LC-HRMS analysis (Orbitrap Fusion) of the organic extracts of *S. lasaliensis* ACP12 (S970A) grown in the in the presence of **20** (final concentration 4 mM): $[M+H]^+$ extracted ion chromatogram (EIC) (Rt = 15.52 min), (B) high resolution mass and (C) fragmentation of putative intermediate **35** with putative fragment structural assignment are shown.

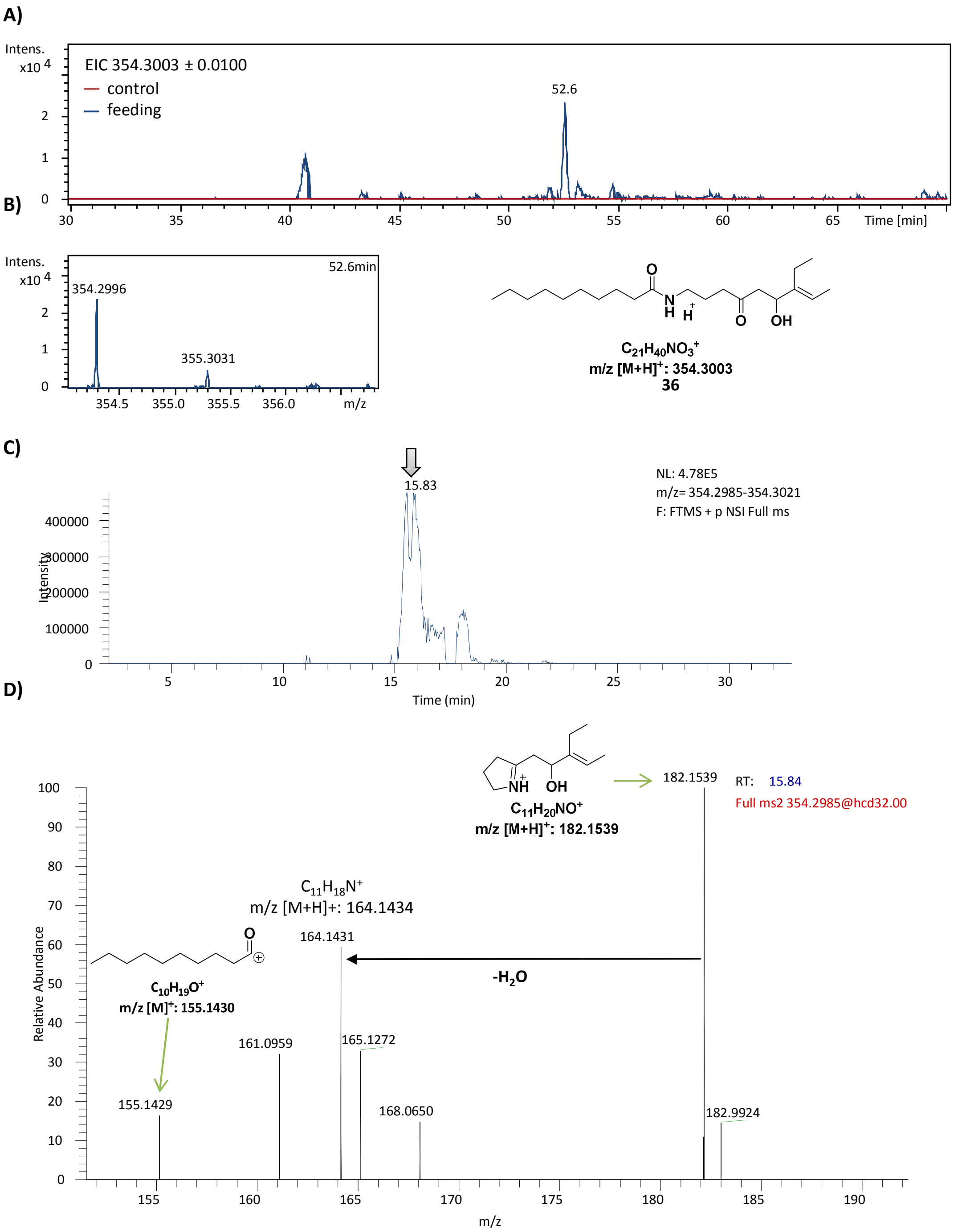


**Figure 31S:** A) LC-HRMS analysis (MaXis Impact, method 2) of the organic extracts of *S. lasaliensis* ACP12 (S970A) grown in the absence (red) and in the presence (blue) of **20** (final concentration 4 mM): $[M+H]^+$ extracted ion chromatogram (EIC) and (B) high resolution mass are shown for the putative intermediate **36** (Rt = 52.6 min). (C) LC-HRMS analysis (Orbitrap Fusion) of the organic extracts of *S. lasaliensis* ACP12 (S970A) grown in the presence of **20** (final concentration 4 mM)**:** EIC (Rt = 15.83 min) and (D) fragmentation of **36** with putative fragment structural assignment.

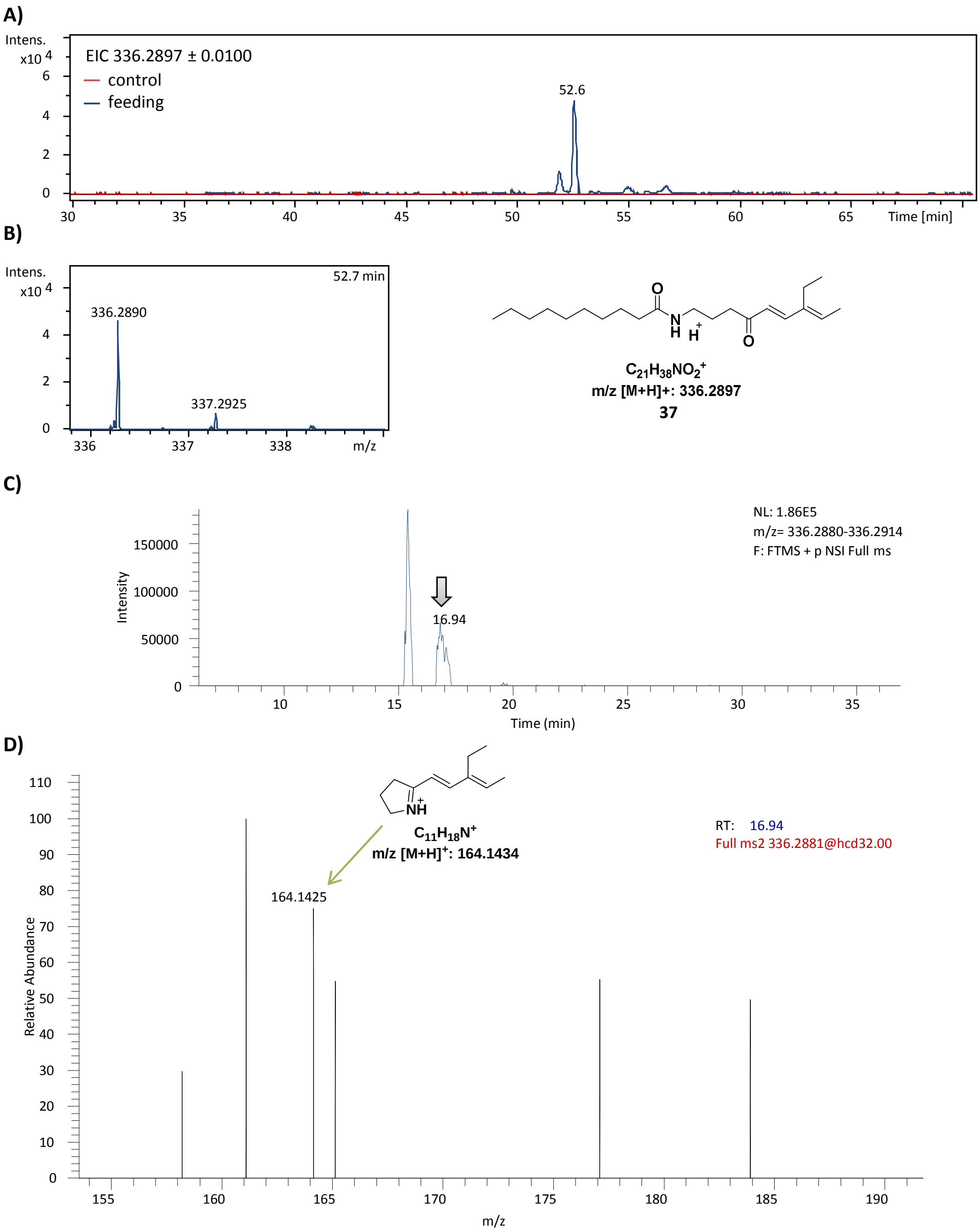


**Figure 32S:** A) LC-HRMS analysis (MaXis Impact, method 2) of the organic extracts of *S. lasaliensis* ACP12 (S970A) grown in the absence (red) and in the presence (blue) of **20** (final concentration 4 mM): $[M+H]^+$ extracted ion chromatogram (EIC) and (B) high resolution mass are shown for the putative intermediate **37** (Rt = 52.6 min). (C) LC-HRMS analysis (Orbitrap Fusion) of the organic extracts of *S. lasaliensis* ACP12 (S970A) grown in the presence of **20** (final concentration 4 mM)**:** EIC (Rt = 16.94 min) and (D) fragmentation of **37** with putative fragment structural assignment.

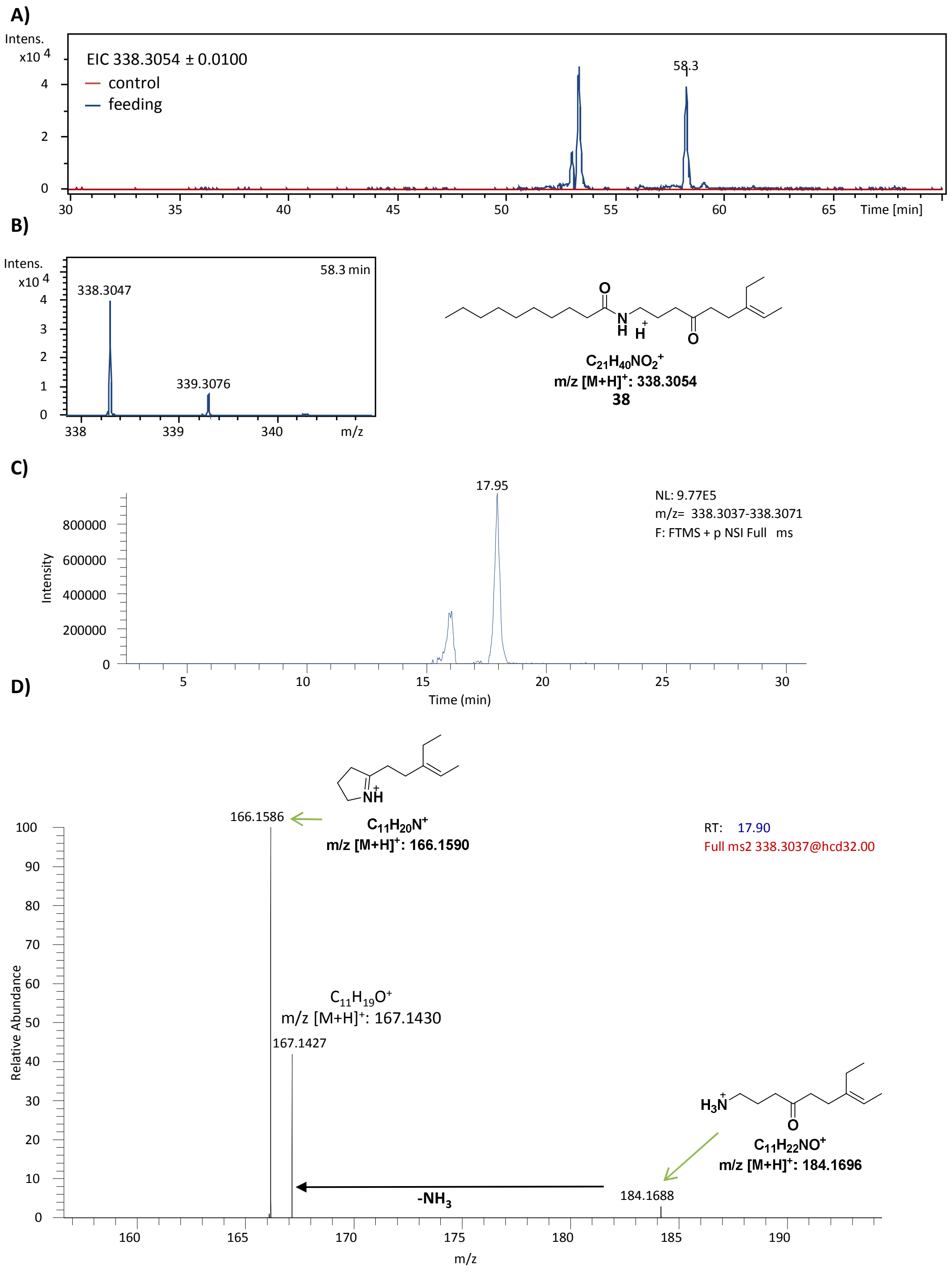


**Figure 33S:** A) LC-HRMS analysis (MaXis Impact, method 2) of the organic extracts of *S. lasaliensis* ACP12 (S970A) grown in the absence (red) and in the presence (blue) of **20** (final concentration 4 mM): $[M+H]^+$ extracted ion chromatogram (EIC) and (B) high resolution mass are shown for the putative intermediate **38** (Rt = 58.3 min). (C) LC-HRMS analysis (Orbitrap Fusion) of the organic extracts of *S. lasaliensis* ACP12 (S970A) grown in the presence of **20** (final concentration 4 mM)**:** EIC (Rt = 17.95 min) and (D) fragmentation of **38** with putative fragment structural assignment.

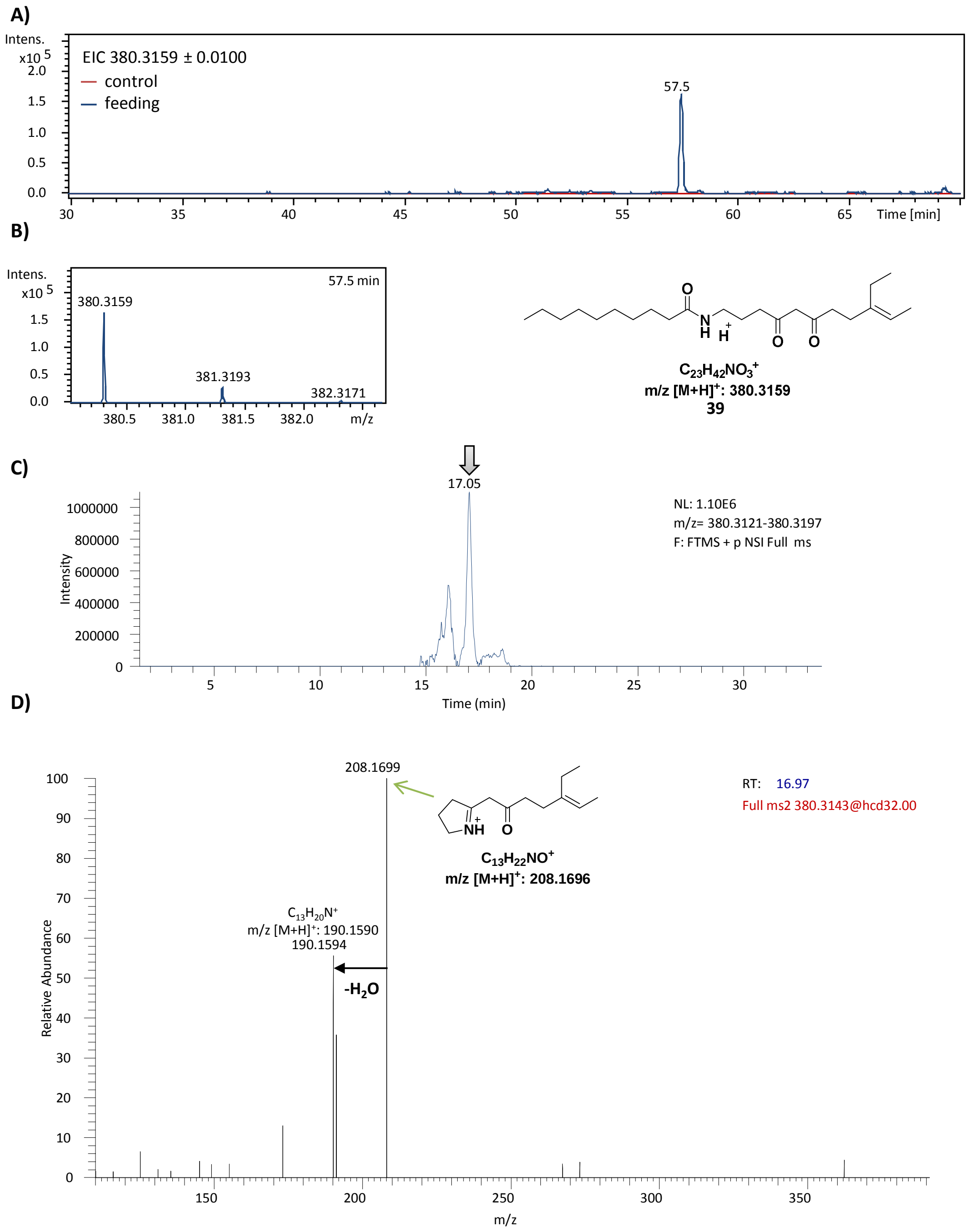


**Figure 34S**: A) LC-HRMS analysis (MaXis Impact, method 2) of the organic extracts of *S. lasaliensis* ACP12 (S970A) grown in the absence (red) and in the presence (blue) of **20** (final concentration 4 mM): $[M+H]^+$ extracted ion chromatogram (EIC) and (B) high resolution mass are shown for the putative intermediate **39** (Rt = 57.5 min). (C) LC-HRMS analysis (Orbitrap Fusion) of the organic extracts of *S. lasaliensis* ACP12 (S970A) grown in the presence of **20** (final concentration 4 mM)**:** EIC (Rt = 17.05 min) and (D) fragmentation of **39** with putative fragment structural assignment.

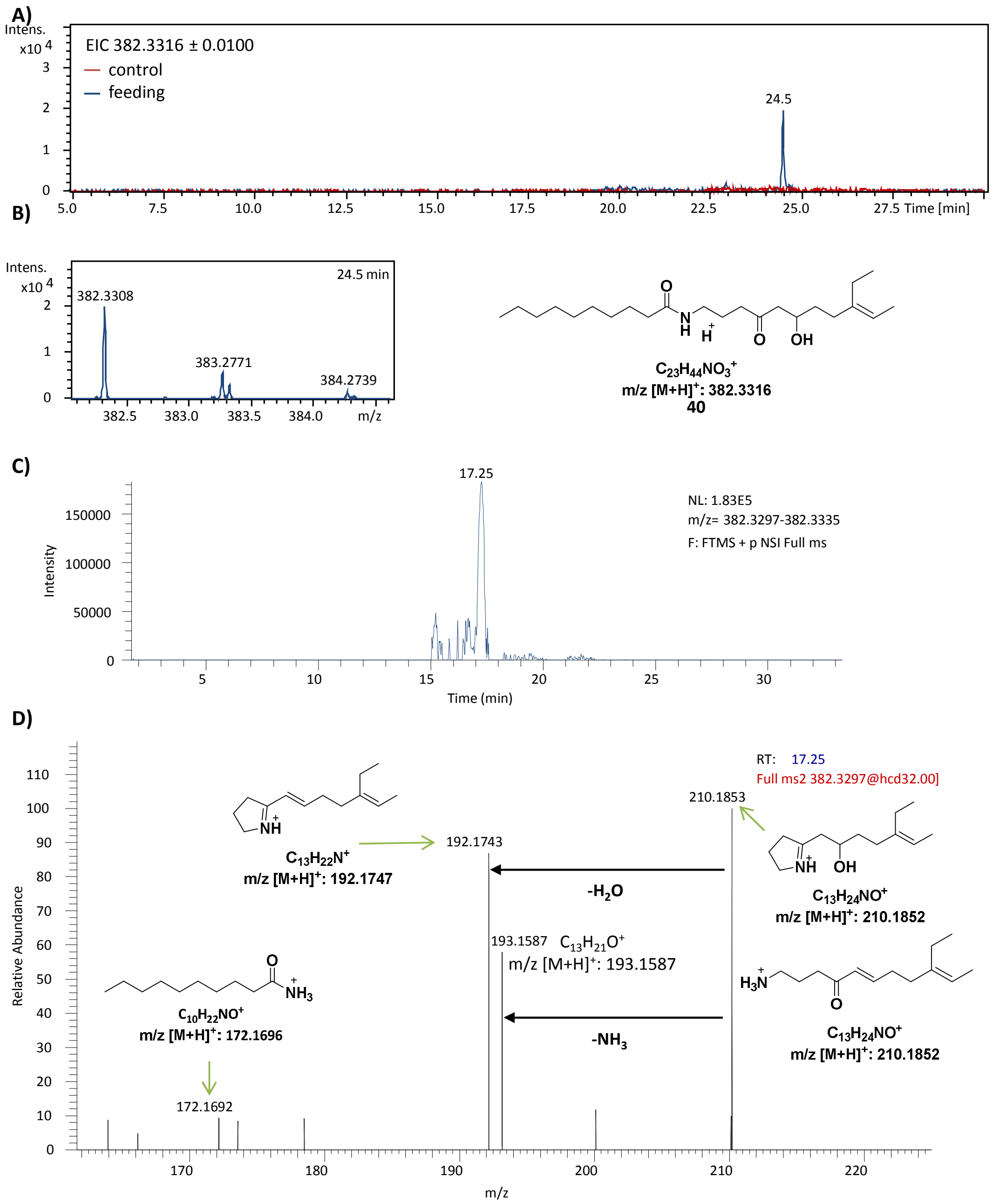


**Figure 35S:** A) LC-HRMS analysis (MaXis Impact, method 2) of the organic extracts of *S. lasaliensis* ACP12 (S970A) grown in the absence (red) and in the presence (blue) of **20** (final concentration 4 mM): $[M+H]^+$ extracted ion chromatogram (EIC) and (B) high resolution mass are shown for the putative intermediate **40** (Rt = 24.5 min). (C) LC-HRMS analysis (Orbitrap Fusion) of the organic extracts of *S. lasaliensis* ACP12 (S970A) grown in the presence of **20** (final concentration 4 mM)**:** EIC (Rt = 17.25 min) and (D) fragmentation of **40** with putative fragment structural assignment.

A)

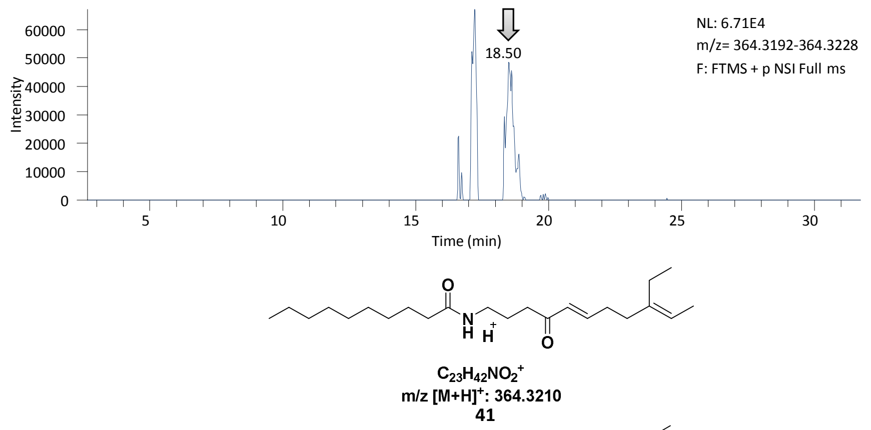


B)

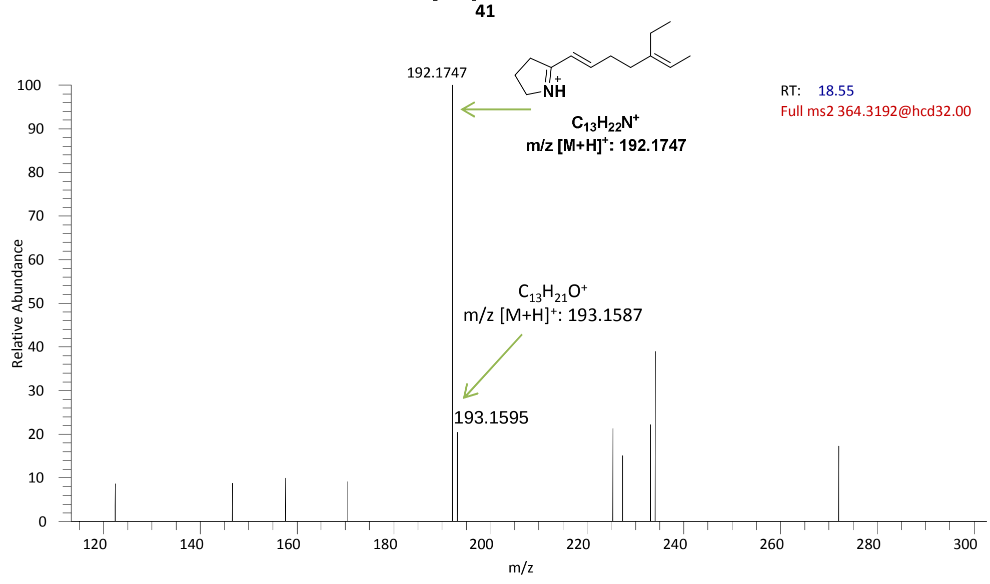


**Figure 36S:** A) LC-HRMS analysis (Orbitrap Fusion) of the organic extracts of *S. lasaliensis* ACP12 (S970A) grown in the presence of **20** (final concentration 4 mM)**:** EIC (Rt = 18.50 min) and (B) fragmentation of putative intermediate **41** with putative fragment structural assignment.

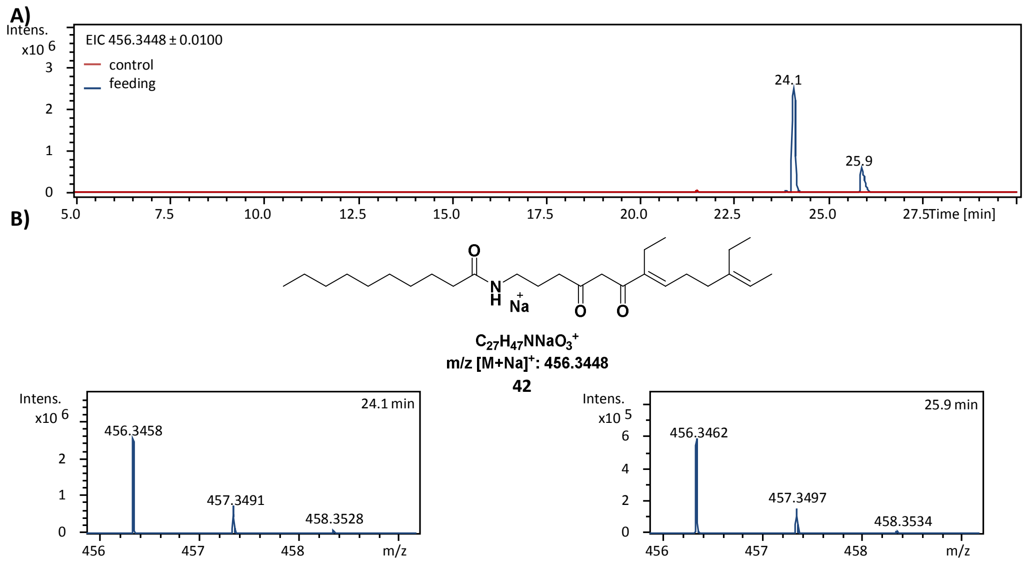

**C)**

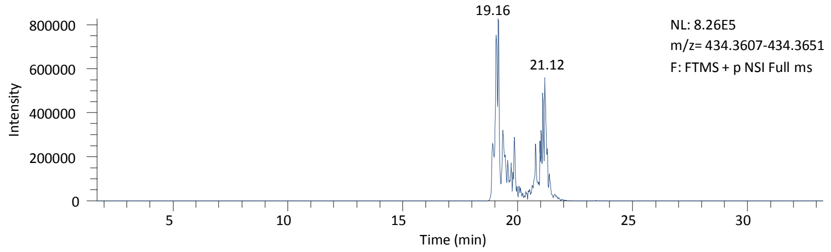


**D)**

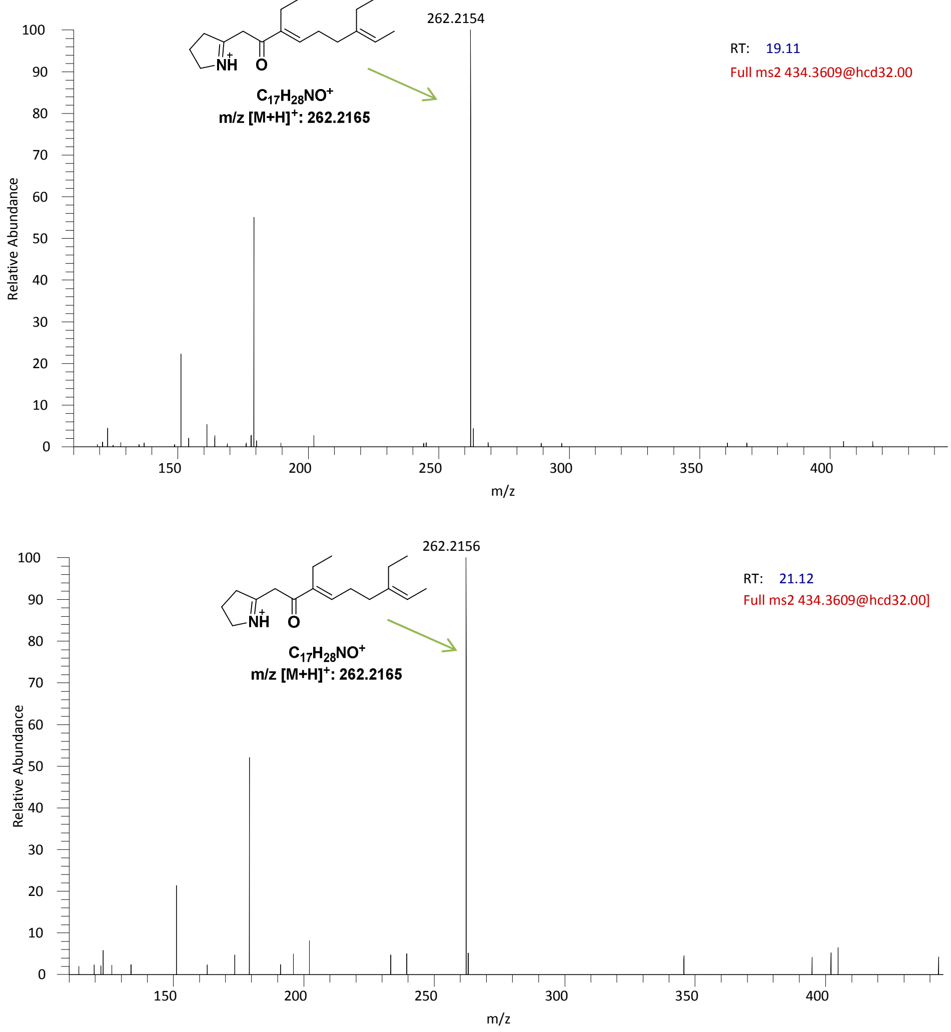


**Figure 37S:** A) LC-HRMS analysis (MaXis Impact, method 2) of the organic extracts of *S. lasaliensis* ACP12 (S970A) grown in the absence (red) and in the presence (blue) of **20** (final concentration 4 mM): $[M+H]^+$ extracted ion chromatogram (EIC) and (B) high resolution mass are shown for the putative intermediate **42** (Rt = 24.1 and 25.9 min). (C) LC-HRMS analysis (Orbitrap Fusion) of the organic extracts of *S. lasaliensis* ACP12 (S970A) grown in the presence of **20** (final concentration 4 mM)**:** EIC (Rt = 19.16 and 21.12 min) and (D) fragmentation of **42** with putative fragment structural assignment. Double peaks may arise from isomerisation (currently under investigation).

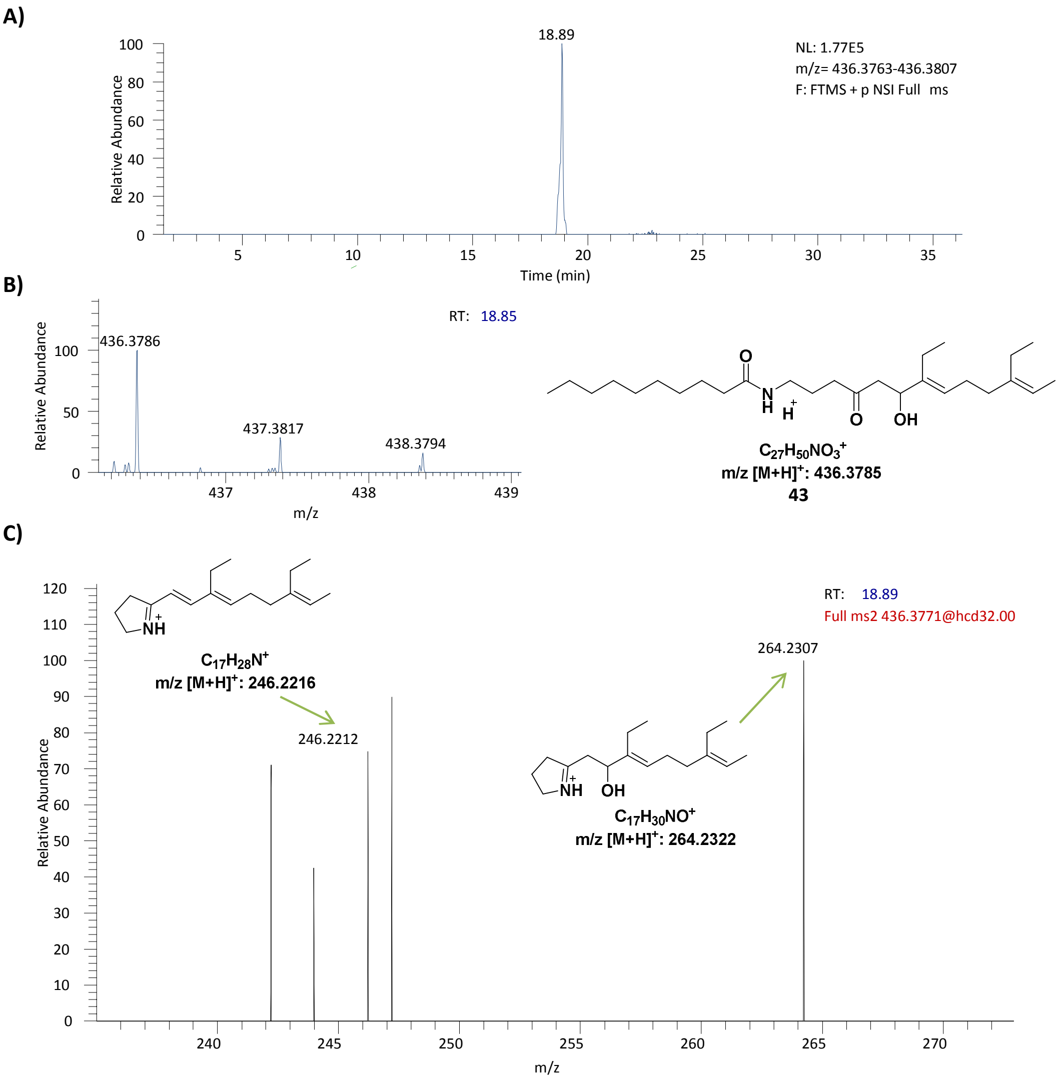


**Figure 38S:** (A) LC-HRMS analysis (Orbitrap Fusion) of the organic extracts of *S. lasaliensis* ACP12 (S970A) grown in the in the presence of **20** (final concentration 4 mM): $[M+H]^+$ extracted ion chromatogram (EIC) (Rt = 18.89 min), (B) high resolution mass and (C) fragmentation of putative intermediate **43** with putative fragment structural assignment are shown.

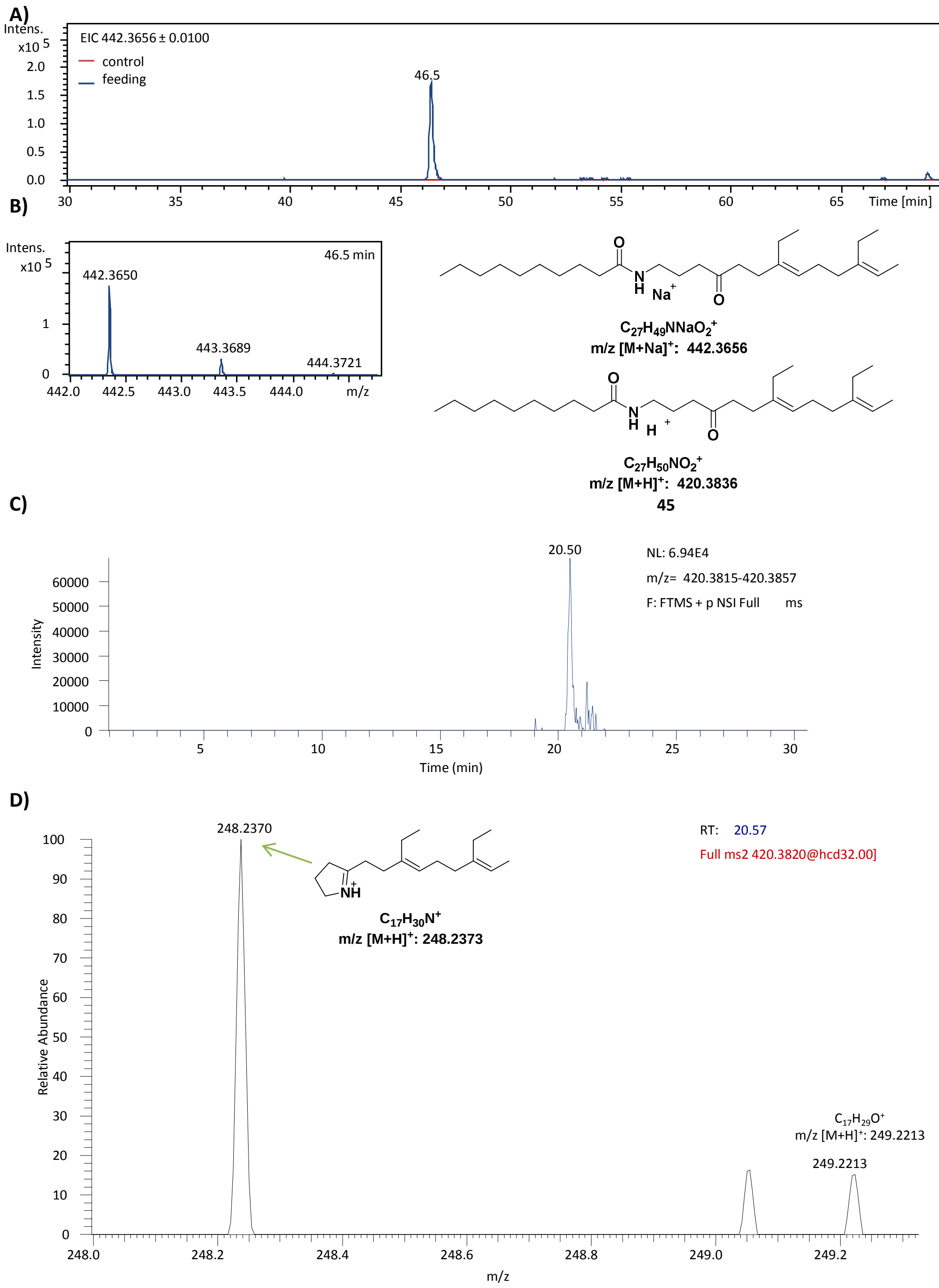


**Figure 39S:** A) LC-HRMS analysis (MaXis Impact, method 2) of the organic extracts of *S. lasaliensis* ACP12 (S970A) grown in the absence (red) and in the presence (blue) of **20** (final concentration 4 mM): $[M+H]^+$ extracted ion chromatogram (EIC) and (B) high resolution mass are shown for the putative intermediate **45** (Rt = 46.5 min). (C) LC-HRMS analysis (Orbitrap Fusion) of the organic extracts of *S. lasaliensis* ACP12 (S970A) grown in the presence of **20** (final concentration 4 mM)**:** EIC (Rt = 20.50 min) and (D) fragmentation of **45** with putative fragment structural assignment.

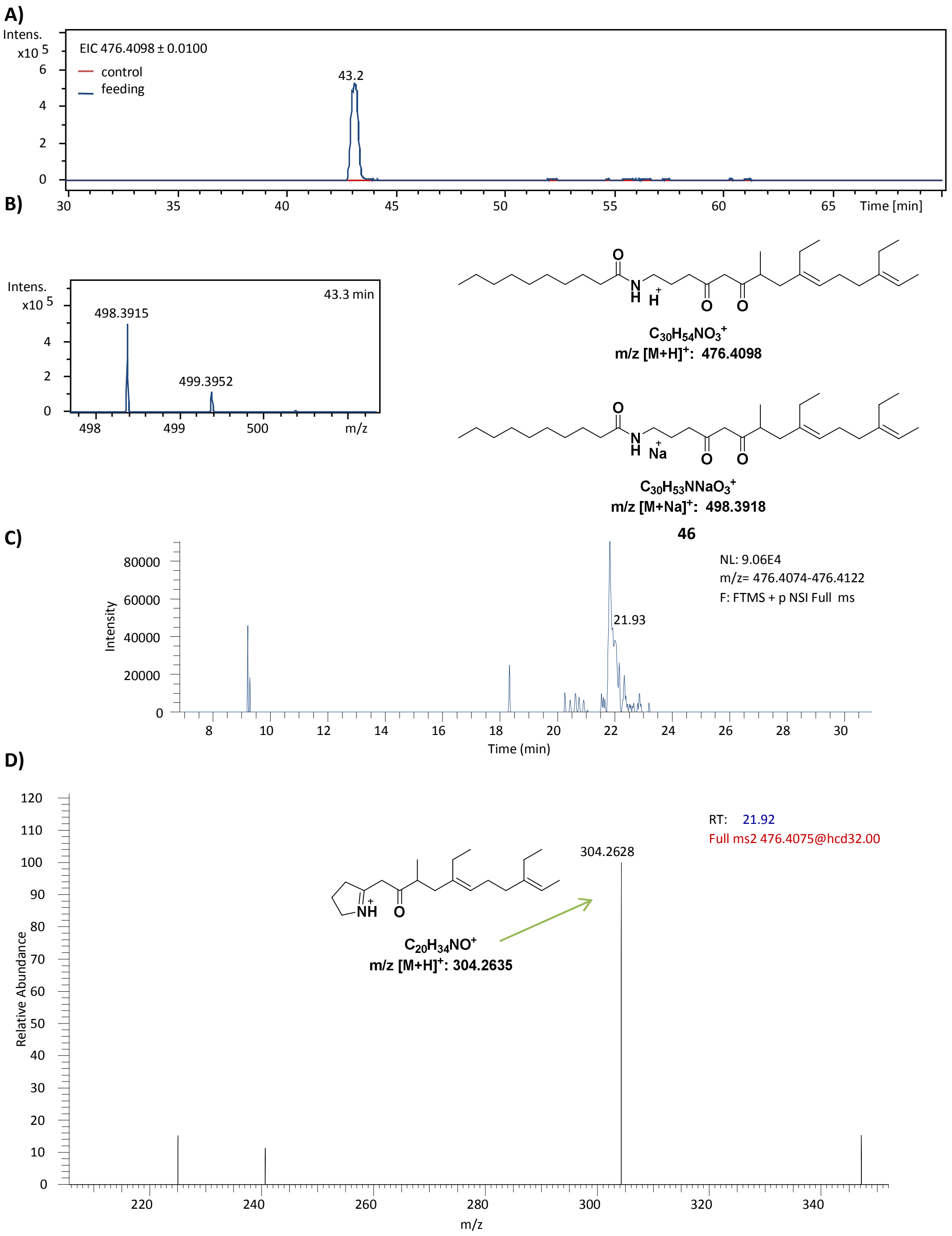


**Figure 40S:** A) LC-HRMS analysis (MaXis Impact, method 2) of the organic extracts of *S. lasaliensis* ACP12 (S970A) grown in the absence (red) and in the presence (blue) of **20** (final concentration 4 mM): $[M+H]^+$ extracted ion chromatogram (EIC) and (B) high resolution mass are shown for the putative intermediate **46** (Rt = 43.2 min). (C) LC-HRMS analysis (Orbitrap Fusion) of the organic extracts of *S. lasaliensis* ACP12 (S970A) grown in the presence of **20** (final concentration 4 mM): EIC (Rt = 21.93 min) and (D) fragmentation of **46** with putative fragment structural assignment.

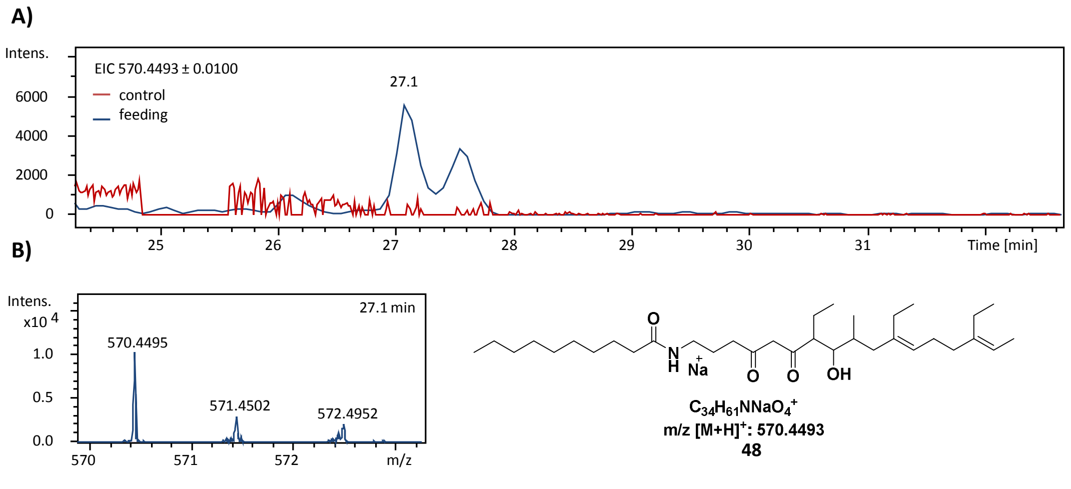


**Figure 41S:** (A) LC-HRMS analysis (MaXis Impact, method 2) of the organic extracts of *S. lasaliensis* ACP12 (S970A) grown in the absence (red) and in the presence (blue) of **20** (final concentration 4 mM): $[M+H]^+$ extracted ion chromatogram (EIC) and (B) high resolution mass are shown for the putative intermediate **48** (Rt = 27.1 min).

A)

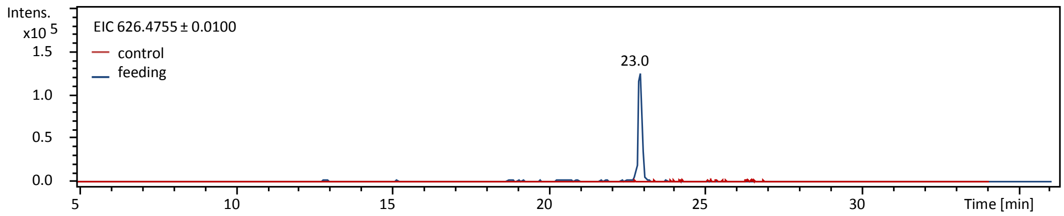


B)

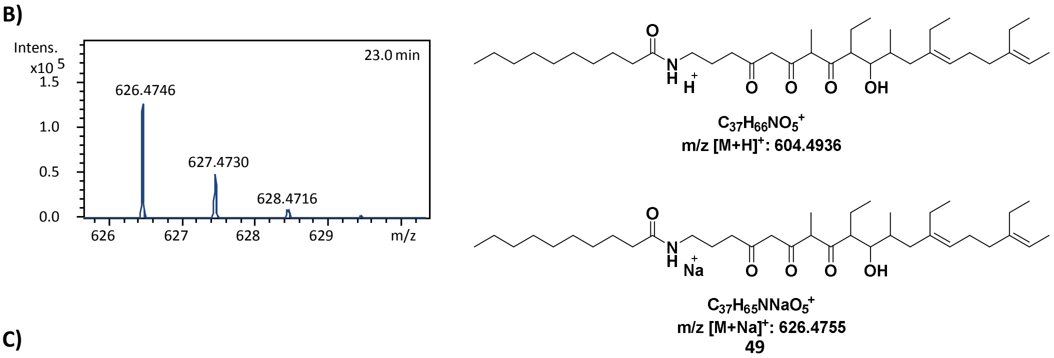


49

C)

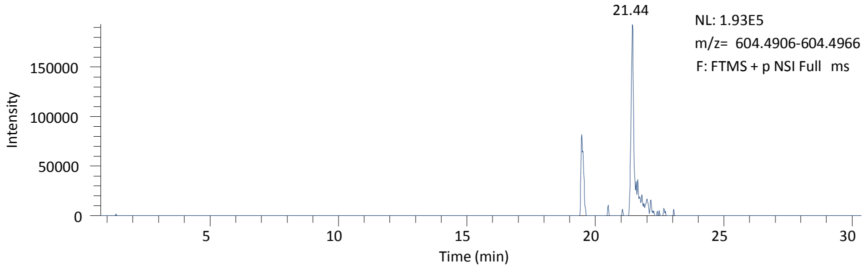


D)

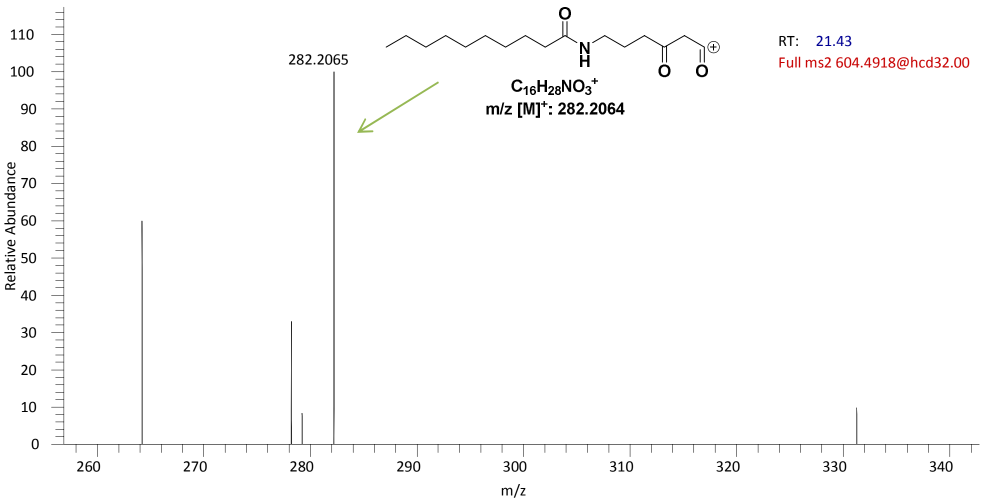


**Figure 42S:** A) LC-HRMS analysis (MaXis Impact, method 2) of the organic extracts of *S. lasaliensis* ACP12 (S970A) grown in the absence (red) and in the presence (blue) of **20** (final concentration 4 mM): [M+H]$^+$ extracted ion chromatogram (EIC) and (B) high resolution mass are shown for the putative intermediate **49** (Rt = 23.0 min). (C) LC-HRMS analysis (Orbitrap Fusion) of the organic extracts of *S. lasaliensis* ACP12 (S970A) grown in the presence of **20** (final concentration 4 mM)**:** EIC (Rt = 21.44 min) and (D) fragmentation of **49** with putative fragment structural assignment. A compound of *m/z* [M+H]= 586.4830 ($C_{37}H_{64}NO_4^+$) resulting from the loss of water from **49** was also detected at an identical retention time (and characterised by the same fragment highlighted in D)).

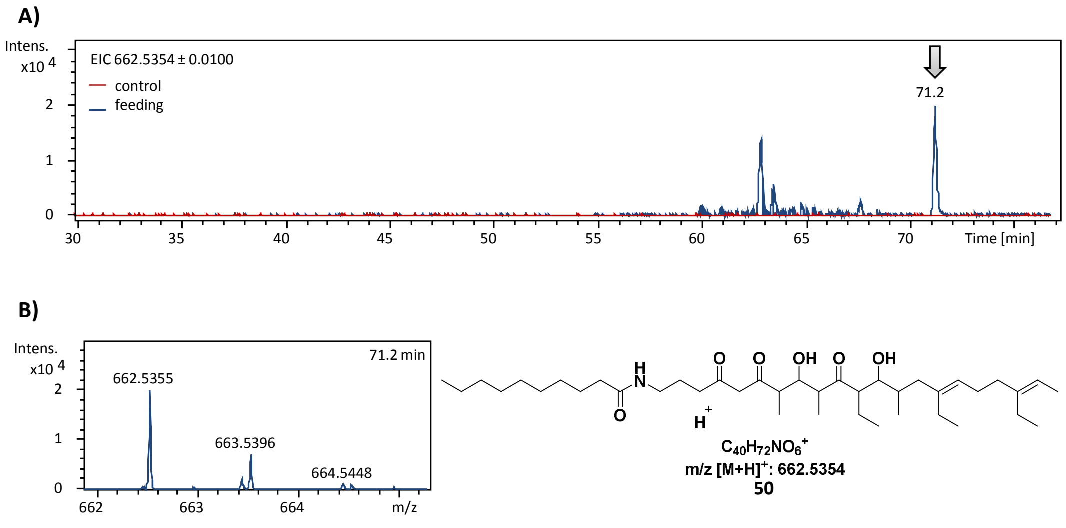


**Figure 43S:** (A) LC-HRMS analysis (MaXis Impact, method 2) of the organic extracts of *S. lasaliensis* ACP12 (S970A) grown in the absence (red) and in the presence (blue) of **20** (final concentration 4 mM): $[M+H]^+$ extracted ion chromatogram (EIC) and (B) high resolution mass are shown for the putative intermediate **50** (Rt = 71.2 min).

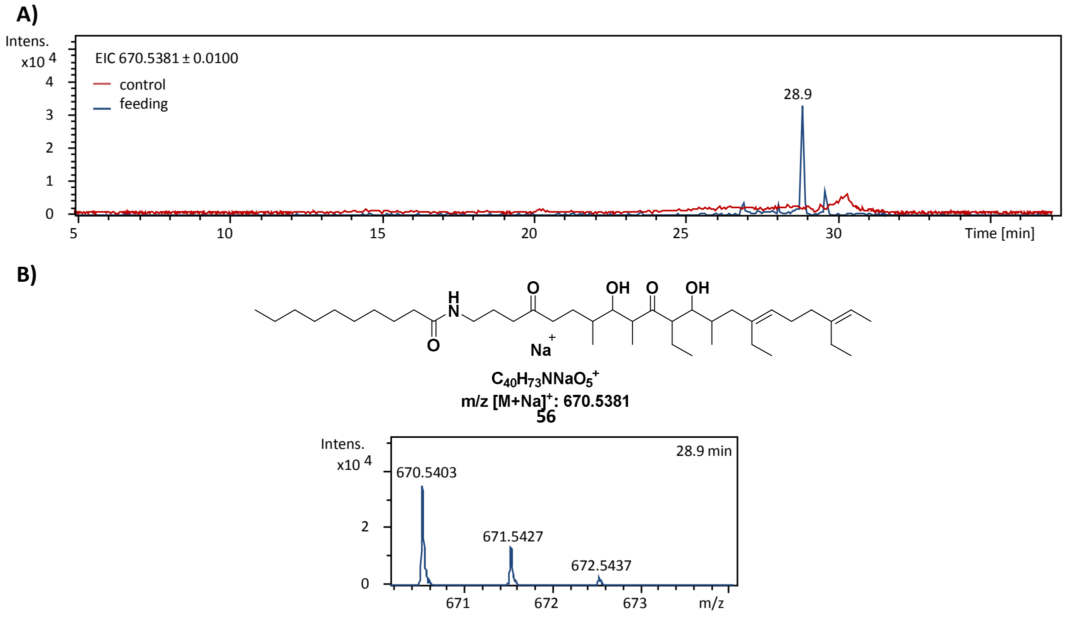


**Figure 44S:** (A) LC-HRMS analysis (MaXis Impact, method 2) of the organic extracts of *S. lasaliensis* ACP12 (S970A) grown in the absence (red) and in the presence (blue) of **20** (final concentration 4 mM): $[M+H]^+$ extracted ion chromatogram (EIC) and (B) high resolution mass are shown for the putative intermediate **56** (Rt = 28.9 min).

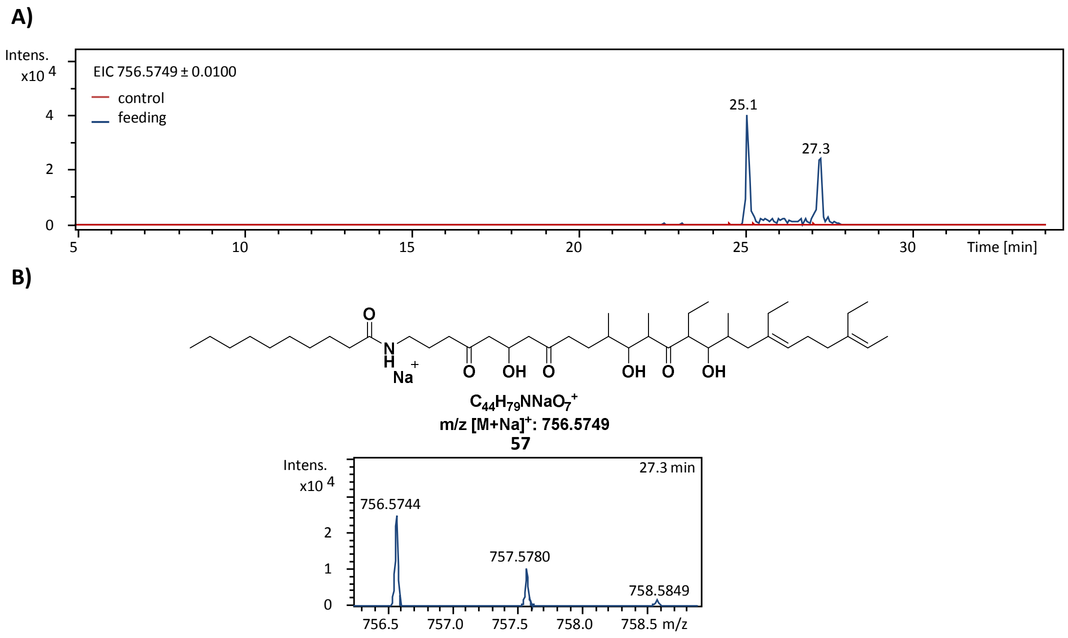


**Figure 45S:** (A) LC-HRMS analysis (MaXis Impact, method 2) of the organic extracts of *S. lasaliensis* ACP12 (S970A) grown in the absence (red) and in the presence (blue) of **20** (final concentration 4 mM): $[M+H]^+$ extracted ion chromatogram (EIC) and (B) high resolution mass are shown for the putative intermediate **57** (Rt = 27.3 min). Double peaks may arise from intramolecular cyclisation or isomerisation (currently under investigation).

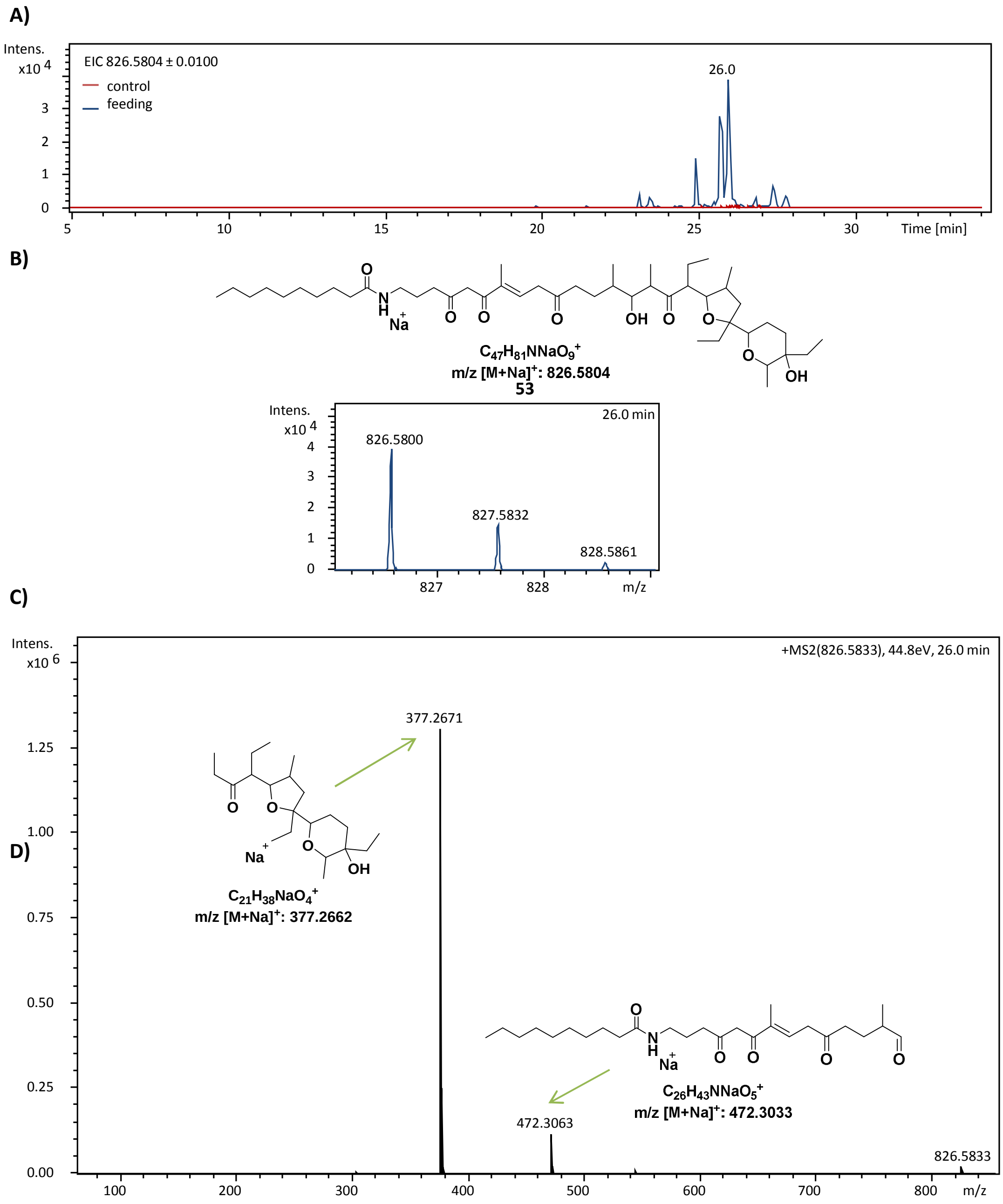


**Figure 46S:** (A) LC-HRMS analysis (MaXis Impact, method 2) of the organic extracts of *S. lasaliensis* ACP12 (S970A) grown in the absence (red) and in the presence (blue) of **20** (final concentration 4 mM): $[M+H]^+$ extracted ion chromatogram (EIC), (B) high resolution mass are shown for the putative intermediate **53** (Rt = 26.0 min) and (C) fragmentation of **53** with putative fragment structural assignment.

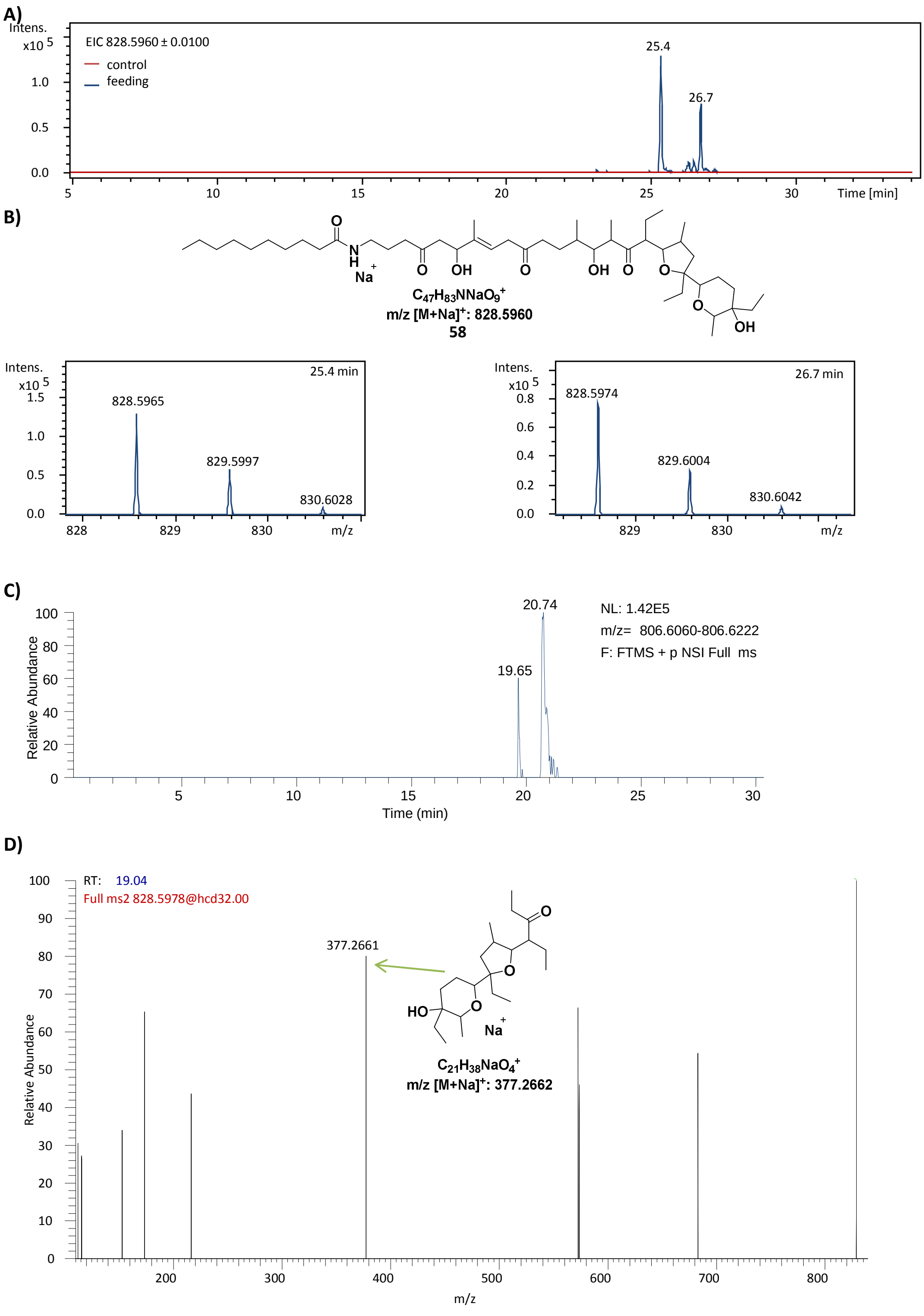


**Figure 47S:** LC-HRMS analysis (MaXis Impact) of the organic extracts of *S. lasaliensis ACP12 (S970A)* grown in the absence (red) and in the presence (blue) of **20** (final concentration 4 mM). $[M+H]^+$ extracted ion traces A) and high resolution mass B) are shown for a putative intermediate **58** (Rt = 25.4 and 26.7 min). Double peaks may arise from intramolecular cyclisation or isomerisation (currently under investigation).

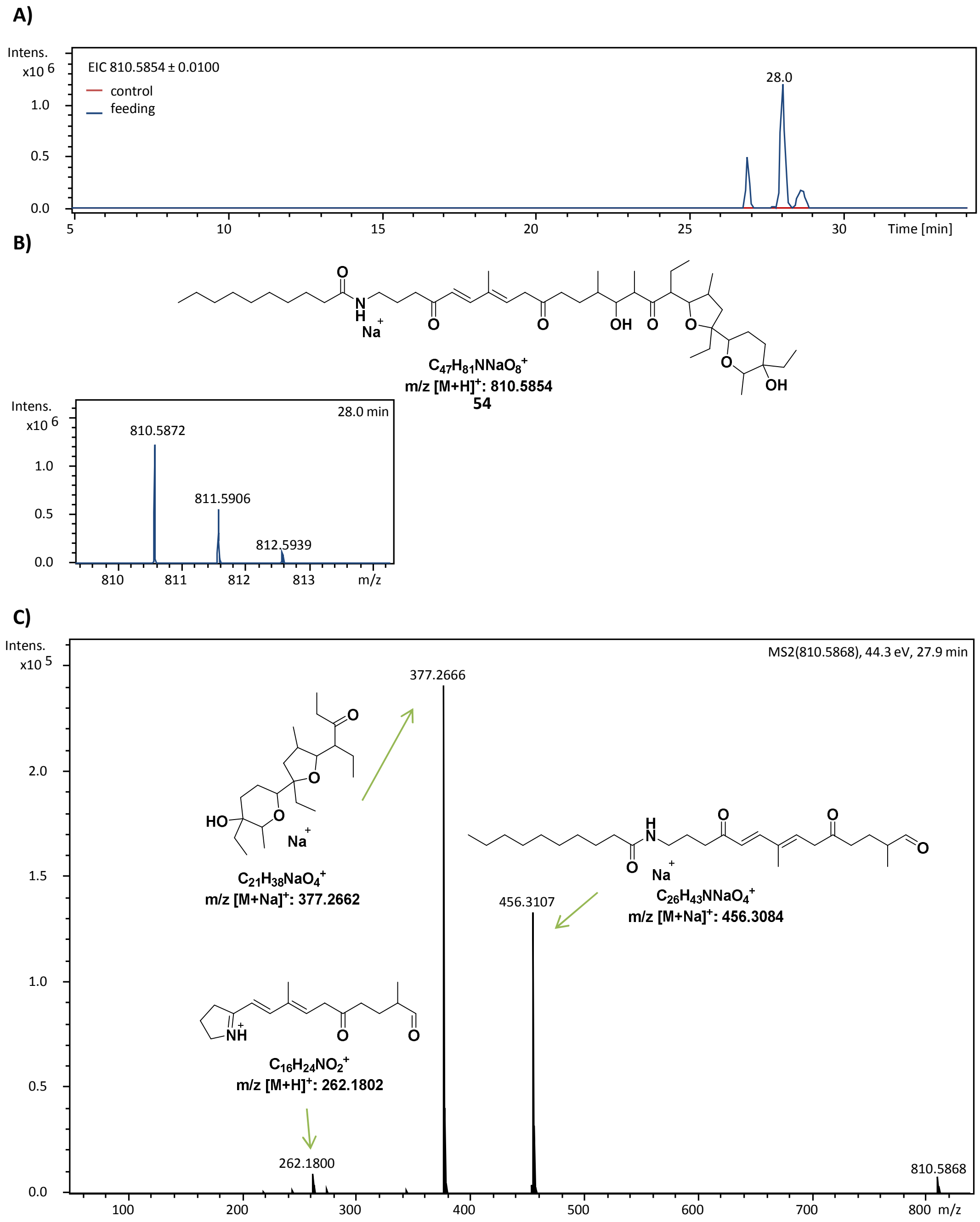


**Figure 48S:** (A) LC-HRMS analysis (MaXis Impact, method 2) of the organic extracts of *S. lasaliensis* ACP12 (S970A) grown in the absence (red) and in the presence (blue) of **20** (final concentration 4 mM): $[M+H]^+$ extracted ion chromatogram (EIC), (B) high resolution mass are shown for the putative intermediate **54** (Rt = 27.9 min) and (C) fragmentation of **54** with putative fragment structural assignment.

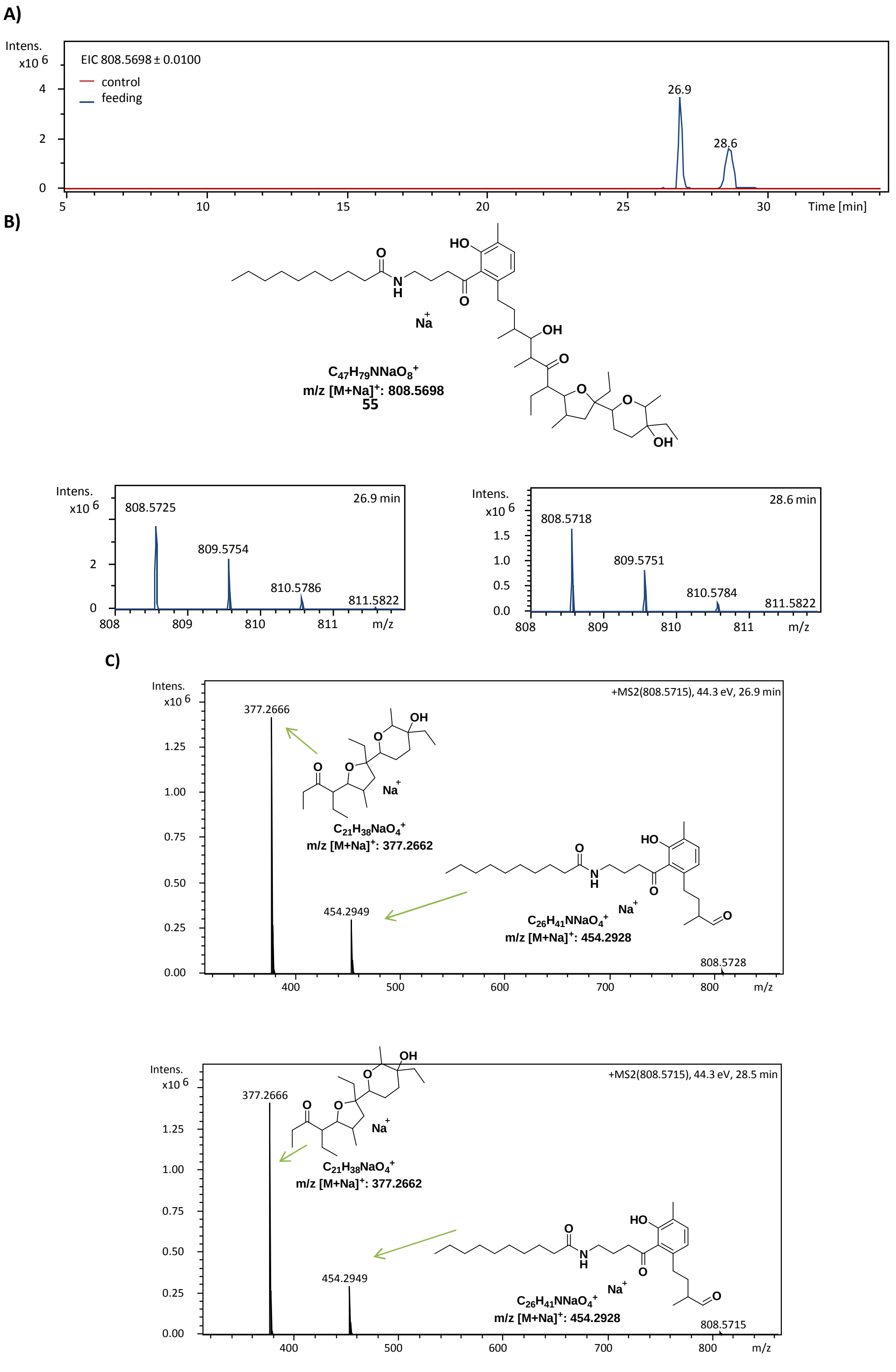


**Figure 49S:** (A) LC-HRMS analysis (MaXis Impact, method 2) of the organic extracts of *S. lasaliensis* ACP12 (S970A) grown in the absence (red) and in the presence (blue) of **20** (final concentration 4 mM): $[M+H]^+$ extracted ion chromatogram (EIC), (B) high resolution mass are shown for the putative intermediate **55** (Rt = 26.9 and 28.5min) and (C) fragmentation of **55** with putative fragment structural assignment. Double peaks may arise isomerisation (currently under investigation).

## 2.4. Intermediate capture by methyl 6-(10-azidodecanamido)-3-oxohexanoate (4)

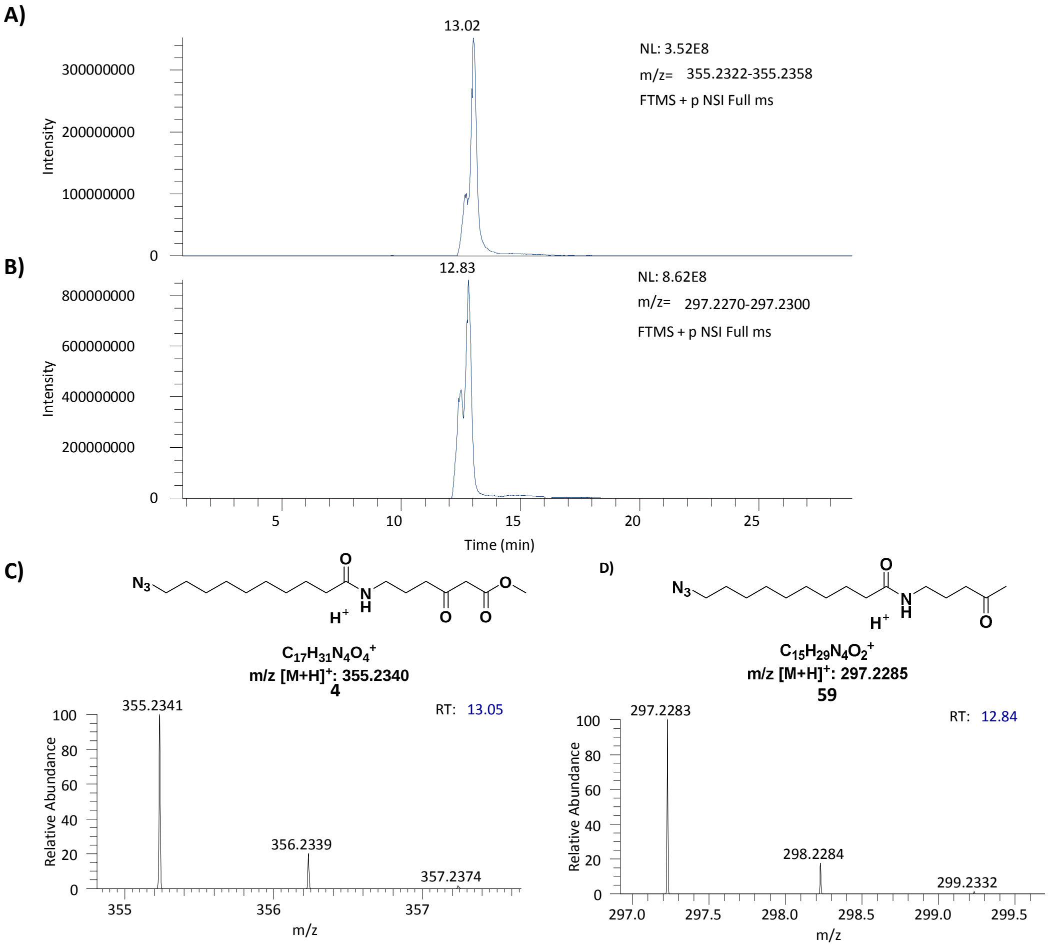


**Figure 50S:** (A) LC-HRMS analysis (Orbitrap Fusion) of the organic extracts of *S. lasaliensis* ACP12 (S970A) grown in the in the presence of **4** (final concentration 4 mM): $[M+H]^+$ extracted ion chromatogram (EIC) for probe **4** (Rt = 13.02 min) and (B) EIC for the decarboxylated probe **59** (Rt = 12.83 min) are shown. (C) High resolution masses of probe **4** and (D) of hydrolysed- decarboxylated probe **59** are shown.

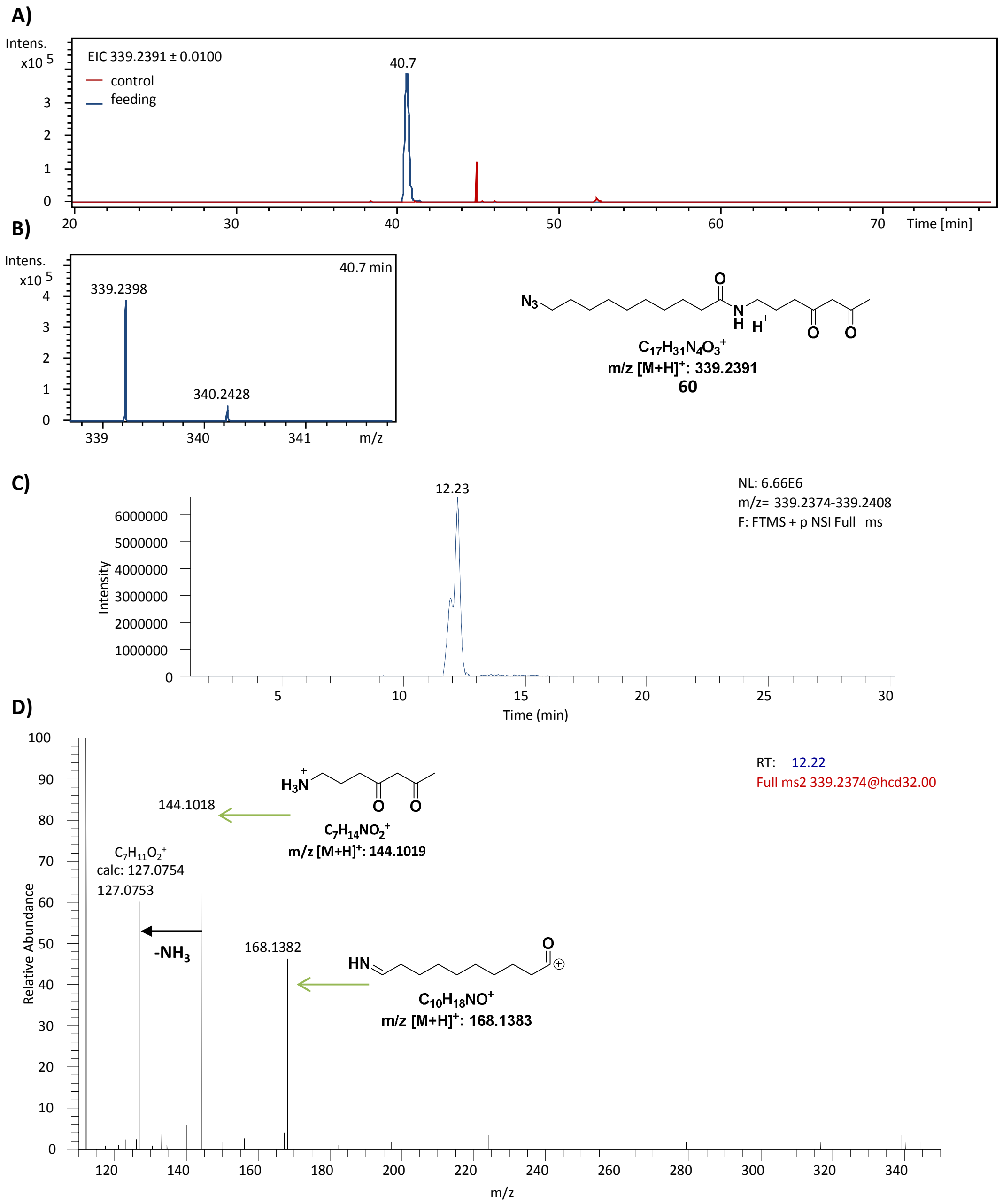


**Figure 51S:** (A) LC-HRMS analysis (MaXis Impact, method 2) of the organic extracts of *S. lasaliensis* ACP12 (S970A) grown in the absence (red) and in the presence (blue) of **4** (final concentration 4 mM): $[M+H]^+$ extracted ion chromatogram (EIC) and (B) high resolution mass are shown for the putative intermediate **60** (Rt = 40.7 min). (C) LC-HRMS analysis (Orbitrap Fusion) of the organic extracts of *S. lasaliensis* ACP12 (S970A) grown in the presence of **4** (final concentration 4 mM): EIC (Rt = 12.23 min) and (D) fragmentation of **60** with putative fragment structural assignment.

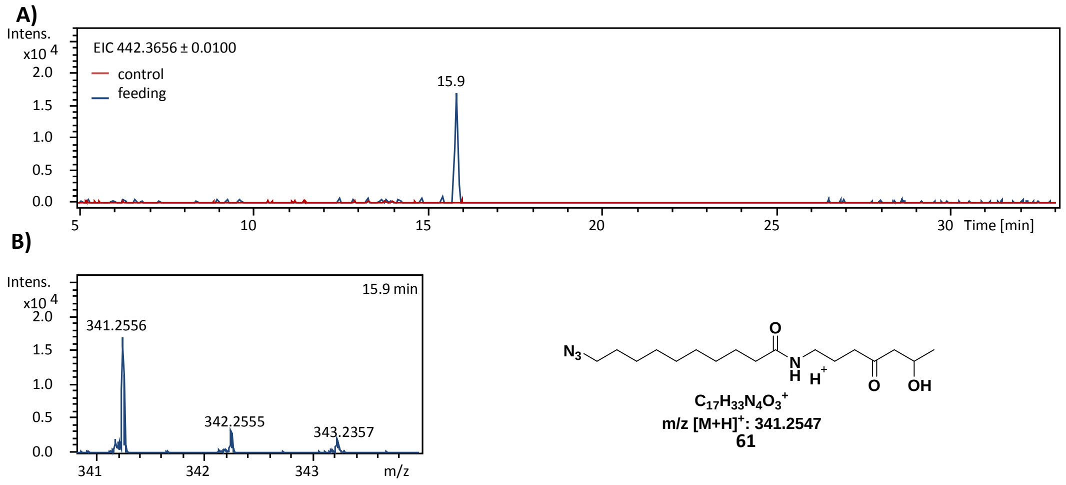


**Figure 52S:** (A) LC-HRMS analysis (MaXis Impact, method 2) of the organic extracts of *S. lasaliensis* ACP12 (S970A) grown in the absence (red) and in the presence (blue) of **4** (final concentration 4 mM): $[M+H]^+$ extracted ion chromatogram (EIC) and (B) high resolution mass are shown for the putative intermediate **61** (Rt = 15.9 min).

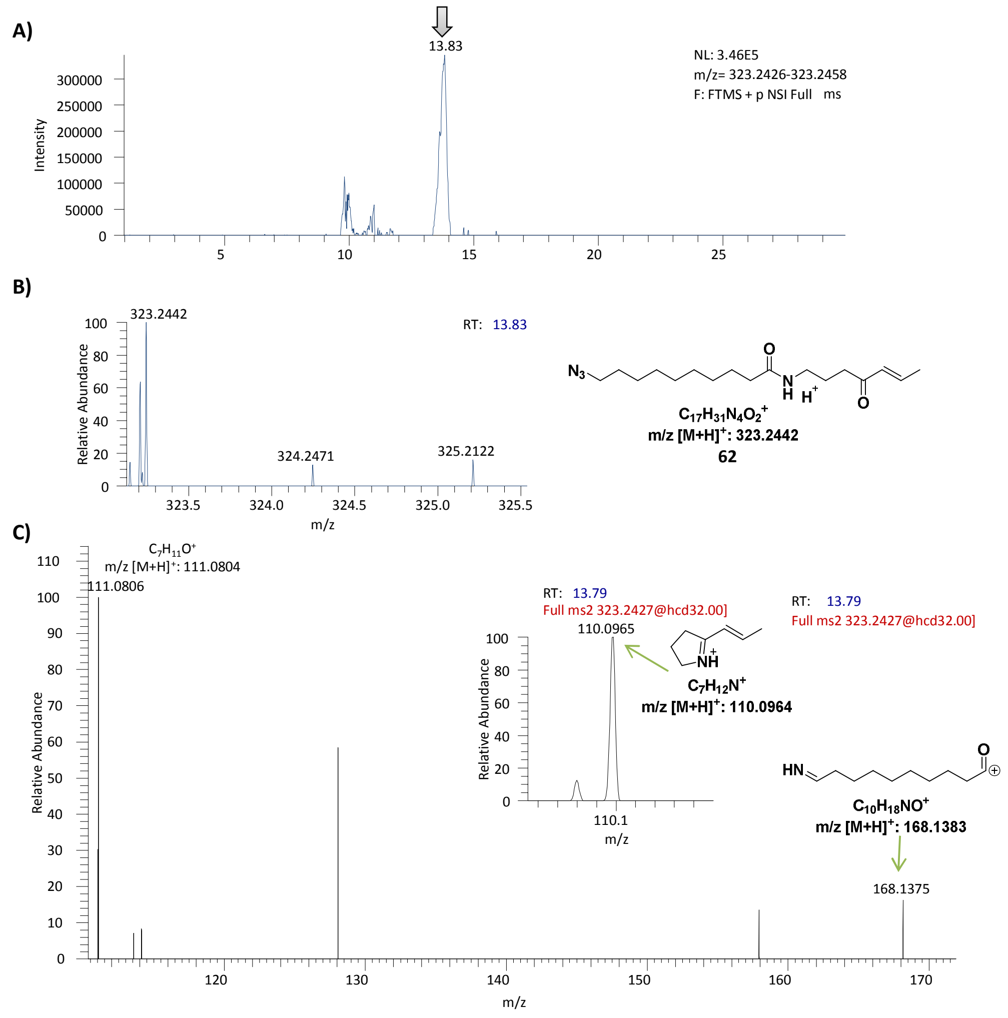


**Figure 53S:** (A) LC-HRMS analysis (Orbitrap Fusion) of the organic extracts of *S. lasaliensis* ACP12 (S970A) grown in the in the presence of **4** (final concentration 4 mM): $[M+H]^+$ extracted ion chromatogram (EIC) (Rt = 13.83 min), (B) high resolution mass and (C) fragmentation of putative intermediate **62** with putative fragment structural assignment are shown.

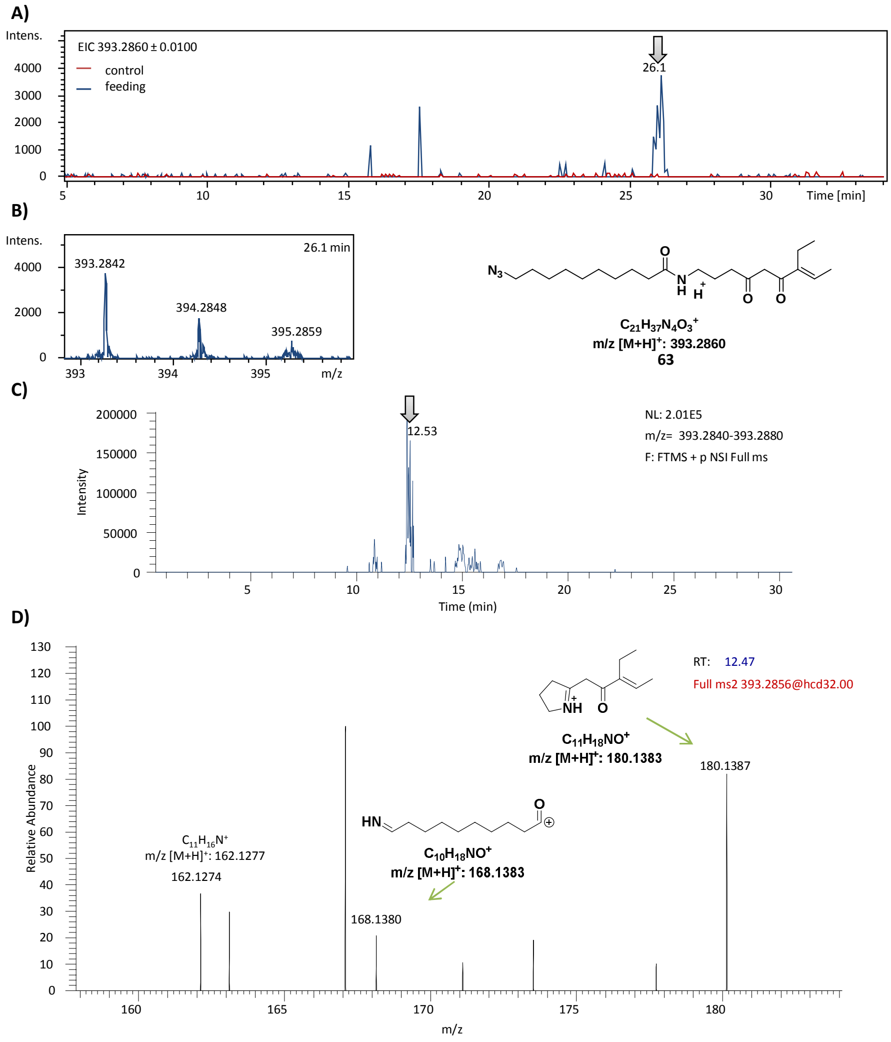


**Figure 54S:** (A) LC-HRMS analysis (MaXis Impact, method 2) of the organic extracts of *S. lasaliensis* ACP12 (S970A) grown in the absence (red) and in the presence (blue) of **4** (final concentration 4 mM): $[M+H]^+$ extracted ion chromatogram (EIC) and (B) high resolution mass are shown for the putative intermediate **63** (Rt = 26.1 min). (C) LC-HRMS analysis (Orbitrap Fusion) of the organic extracts of *S. lasaliensis* ACP12 (S970A) grown in the presence of **4** (final concentration 4 mM)**:** EIC (Rt = 12.53 min) and (D) fragmentation of **63** with putative fragment structural assignment.

**A)**

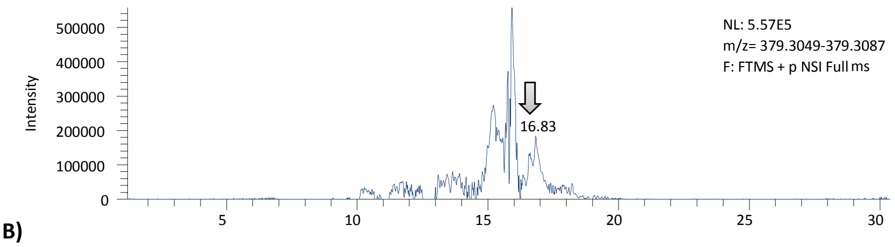


**B)**

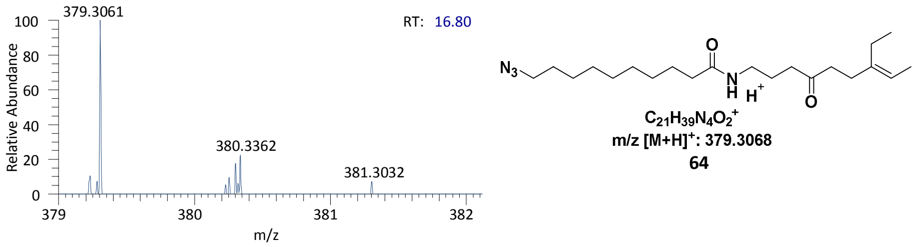


**C)**

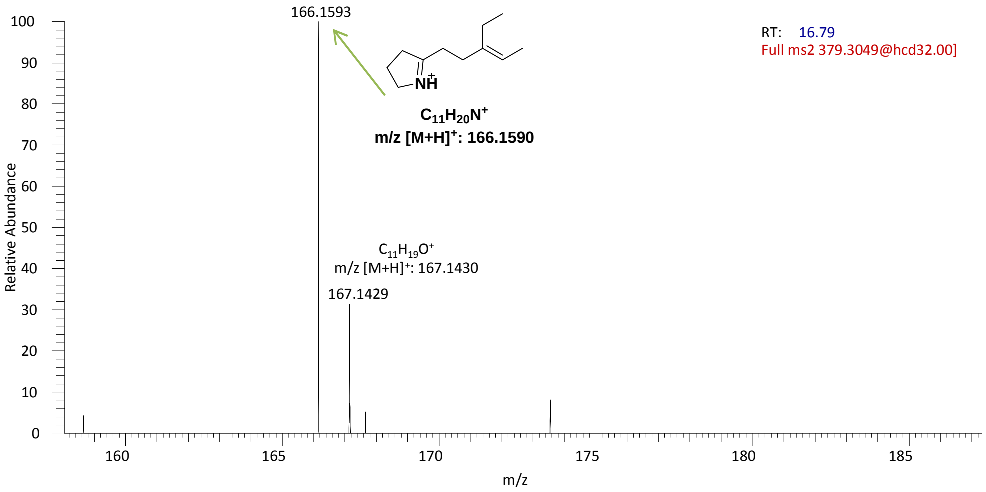


**Figure 55S:** (A) LC-HRMS analysis (Orbitrap Fusion) of the organic extracts of *S. lasaliensis* ACP12 (S970A) grown in the in the presence of **4** (final concentration 4 mM): $[M+H]^+$ extracted ion chromatogram (EIC) (Rt = 16.83 min), (B) high resolution mass and (C) fragmentation of putative intermediate **64** with putative fragment structural assignment are shown.

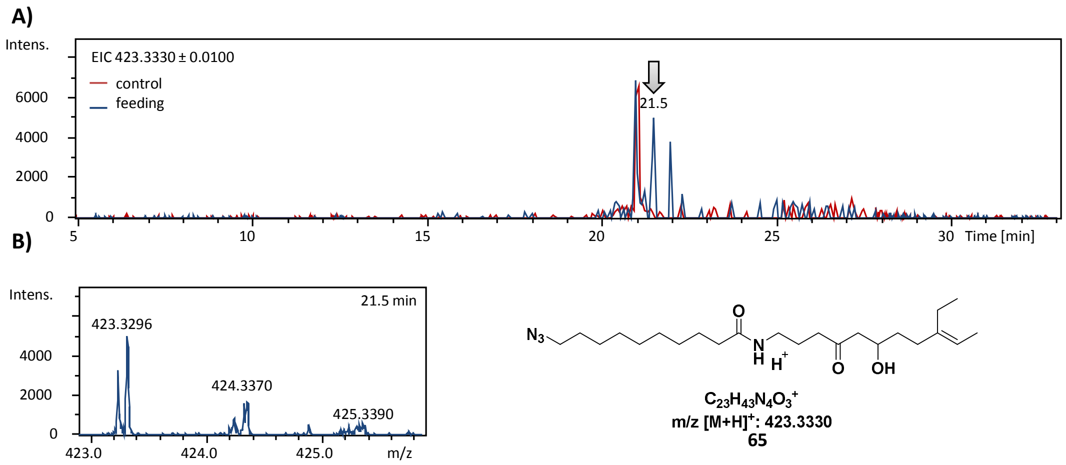


**Figure 56S:** (A) LC-HRMS analysis (MaXis Impact, method 2) of the organic extracts of *S. lasaliensis* ACP12 (S970A) grown in the absence (red) and in the presence (blue) of **4** (final concentration 4 mM): $[M+H]^+$ extracted ion chromatogram (EIC) and (B) high resolution mass are shown for the putative intermediate **65** (Rt = 21.5 min).

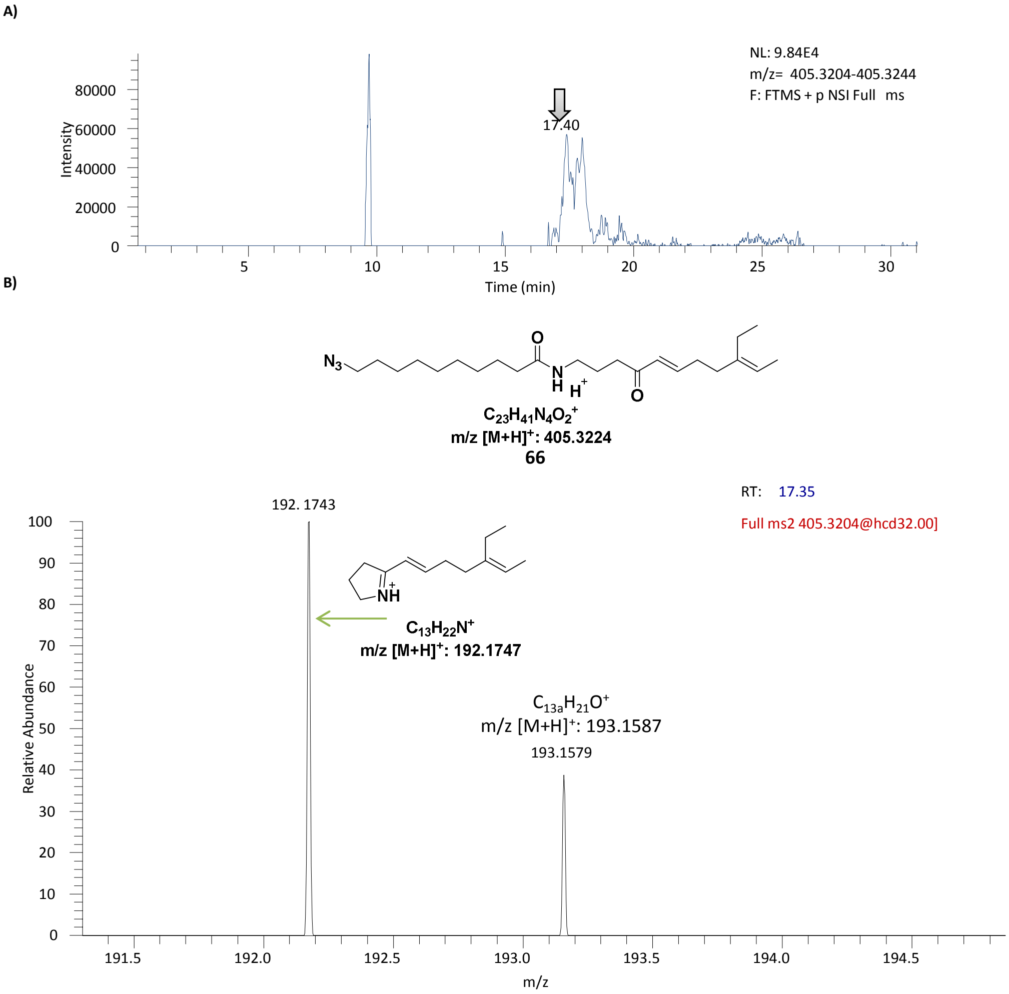


**Figure 57S:** (A) LC-HRMS analysis (Orbitrap Fusion) of the organic extracts of *S. lasaliensis* ACP12 (S970A) grown in the in the presence of **4** (final concentration 4 mM): $[M+H]^+$ extracted ion chromatogram (EIC) (Rt = 17.40 min) and (B) fragmentation of putative intermediate **66** with putative fragment structural assignment are shown.

**A)**

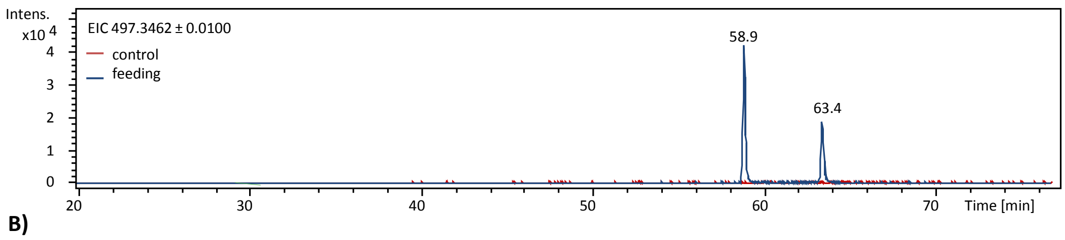


**B)**

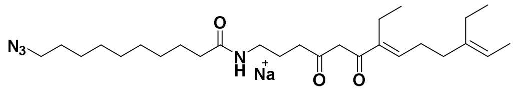


**$C_{27}H_{46}N_4NaO_3^+$**
**m/z $[M+Na]^+$: 497.3462**
**67**

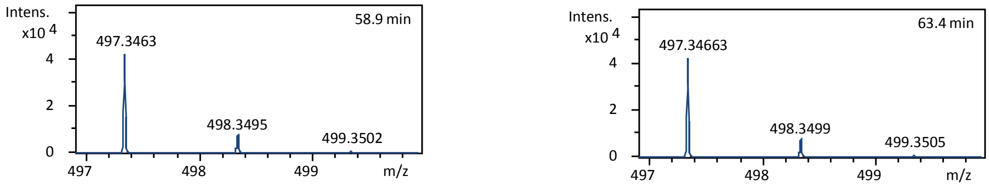

**C)**

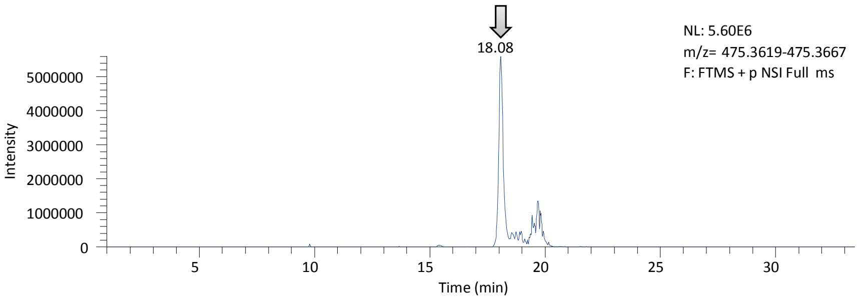


**D)**

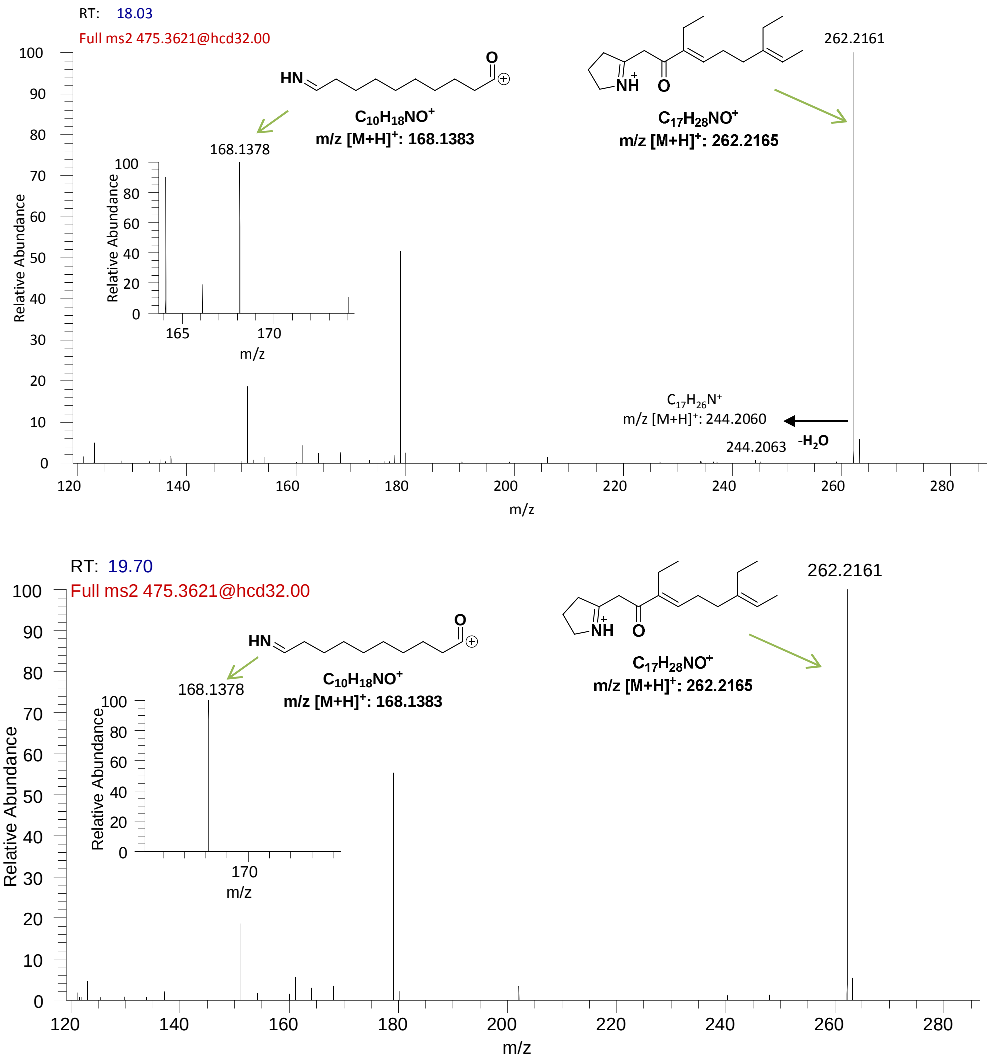


**Figure 58S:** (A) LC-HRMS analysis (MaXis Impact, method 2) of the organic extracts of *S. lasaliensis* ACP12 (S970A) grown in the absence (red) and in the presence (blue) of **4** (final concentration 4 mM): $[M+H]^+$ extracted ion chromatogram (EIC) and (B) high resolution mass are shown for the putative intermediate **67** (Rt = 58.9 and 63.4 min). (C) LC-HRMS analysis (Orbitrap Fusion) of the organic extracts of *S. lasaliensis* ACP12 (S970A) grown in the presence of **4** (final concentration 4 mM)**:** EIC (Rt = 18.08 and 19.70 min) and (D) fragmentation of **67** with putative fragment structural assignment. Double peaks may arise from isomerisation (currently under investigation).

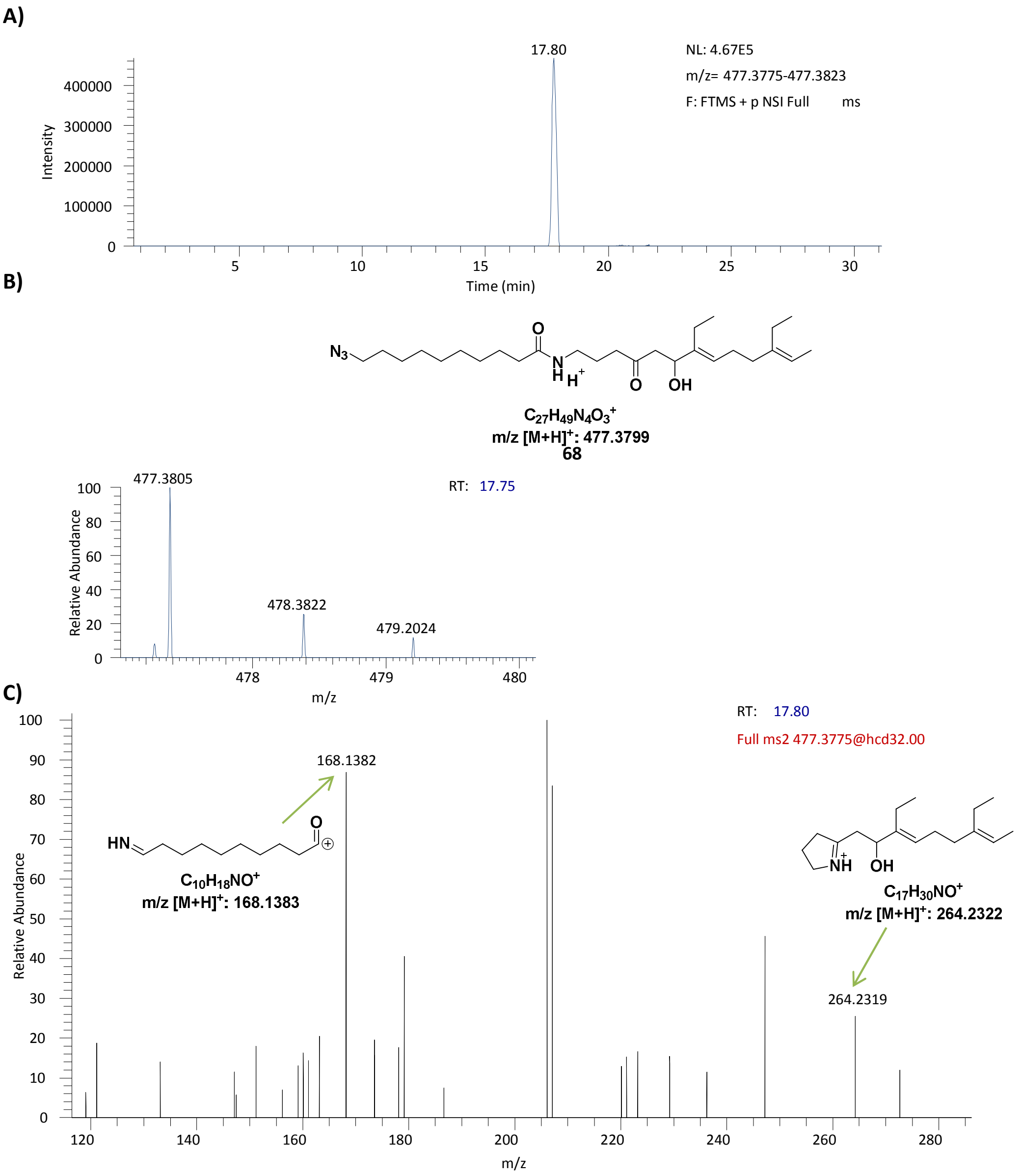


**Figure 59S:** (A) LC-HRMS analysis (Orbitrap Fusion) of the organic extracts of *S. lasaliensis* ACP12 (S970A) grown in the in the presence of **4** (final concentration 4 mM): $[M+H]^+$ extracted ion chromatogram (EIC) (Rt = 17.80 min), (B) high resolution mass and (C) fragmentation of putative intermediate **68** with putative fragment structural assignment are shown.

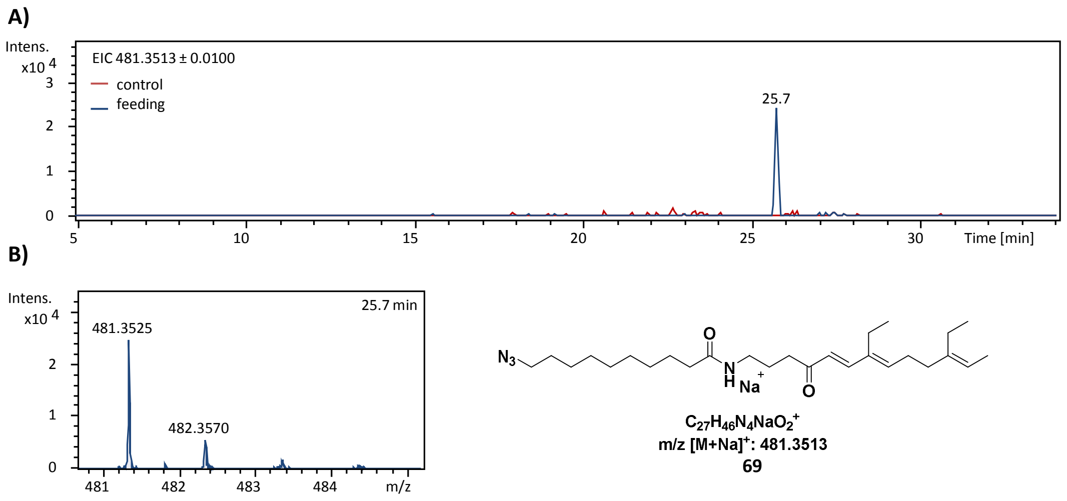


**Figure 60S:** (A) LC-HRMS analysis (MaXis Impact, method 2) of the organic extracts of *S. lasaliensis* ACP12 (S970A) grown in the absence (red) and in the presence (blue) of **4** (final concentration 4 mM): $[M+H]^+$ extracted ion chromatogram (EIC) and (B) high resolution mass are shown for the putative intermediate **69** (Rt = 25.7 min).

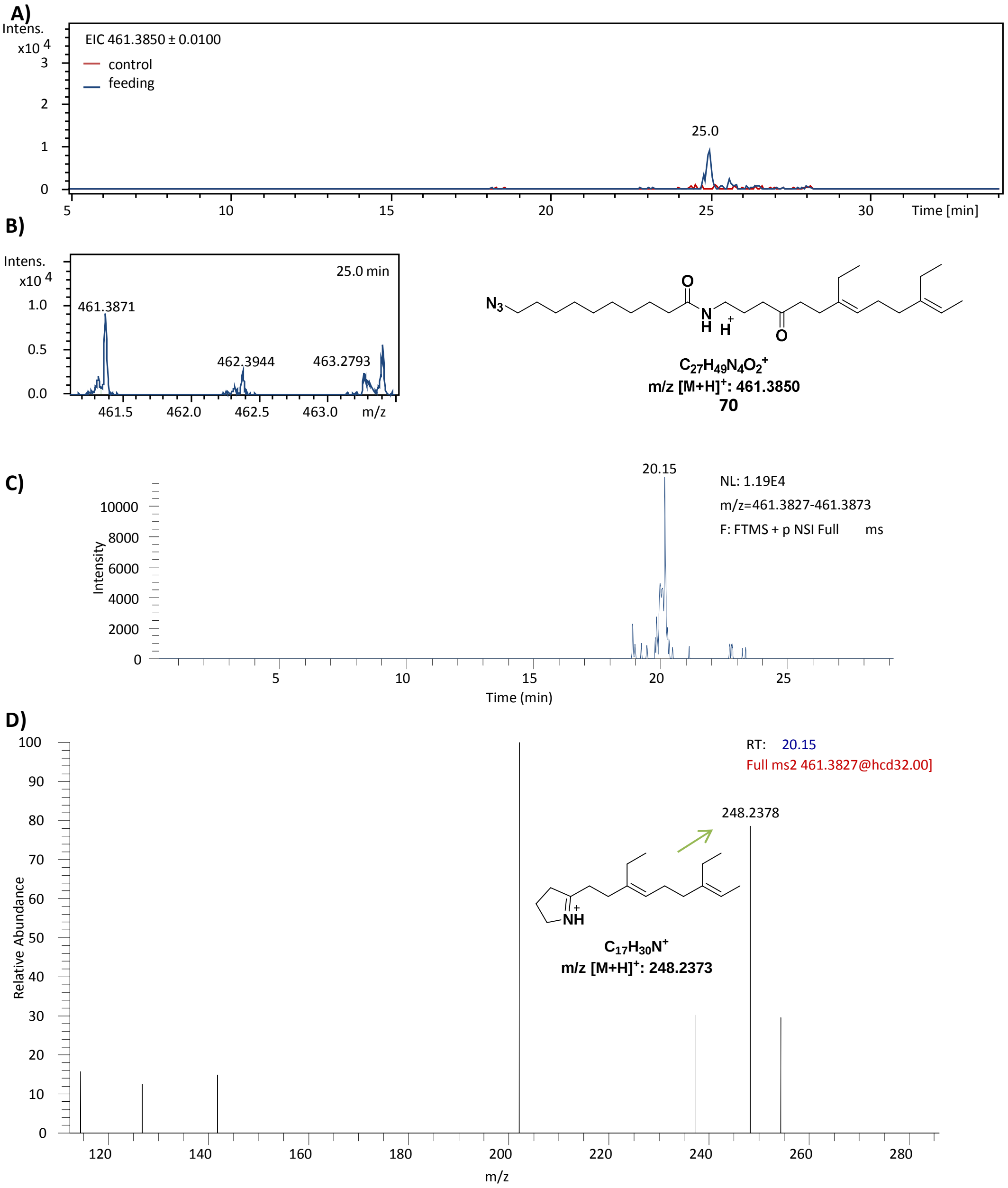


**Figure 61S:** (A) LC-HRMS analysis (MaXis Impact, method 2) of the organic extracts of *S. lasaliensis* ACP12 (S970A) grown in the absence (red) and in the presence (blue) of **4** (final concentration 4 mM): $[M+H]^+$ extracted ion chromatogram (EIC) and (B) high resolution mass are shown for the putative intermediate **70** (Rt = 25.0 min). (C) LC-HRMS analysis (Orbitrap Fusion) of the organic extracts of *S. lasaliensis* ACP12 (S970A) grown in the presence of **4** (final concentration 4 mM)**:** EIC (Rt = 20.15 min) and (D) fragmentation of **70** with putative fragment structural assignment.

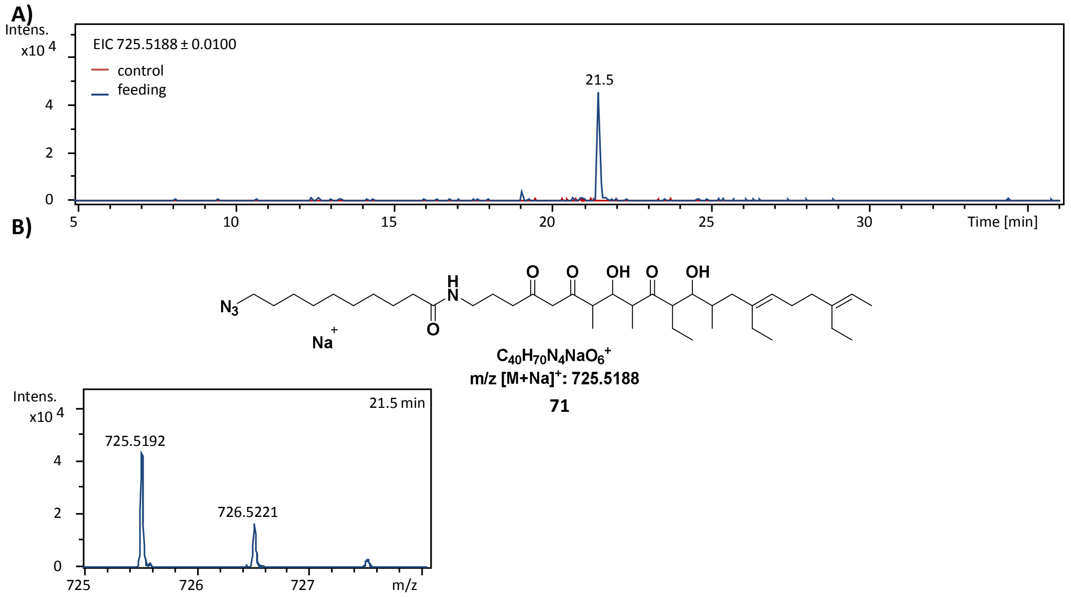


**Figure 62S:** (A) LC-HRMS analysis (MaXis Impact, method 2) of the organic extracts of *S. lasaliensis* ACP12 (S970A) grown in the absence (red) and in the presence (blue) of **4** (final concentration 4 mM): $[M+H]^+$ extracted ion chromatogram (EIC) and (B) high resolution mass are shown for the putative intermediate **71** (Rt = 21.5 min).

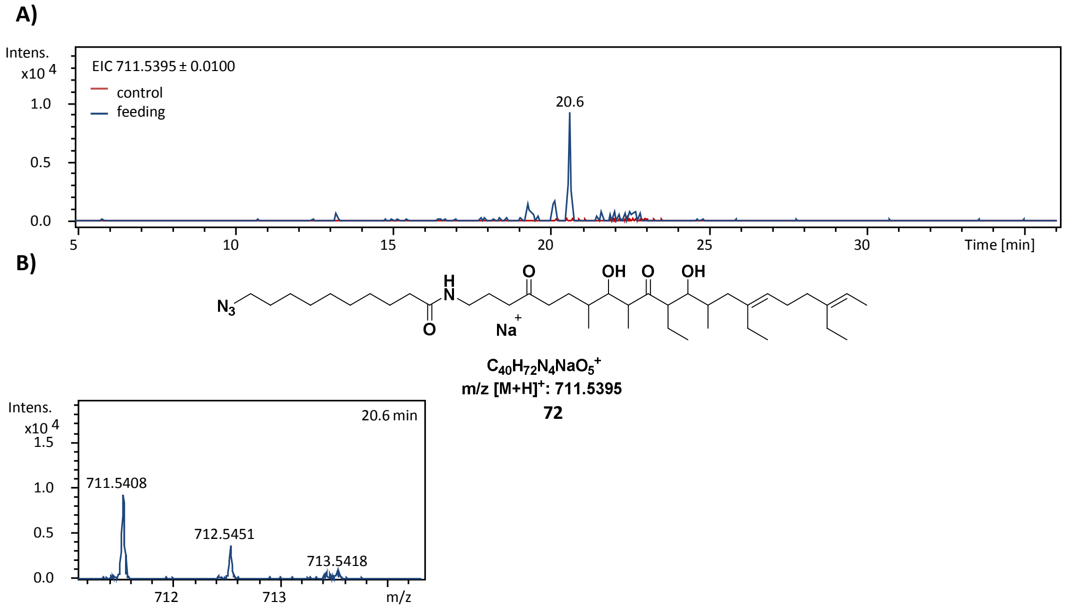


**Figure 63S:** (A) LC-HRMS analysis (MaXis Impact, method 2) of the organic extracts of *S. lasaliensis* ACP12 (S970A) grown in the absence (red) and in the presence (blue) of **4** (final concentration 4 mM): $[M+H]^+$ extracted ion chromatogram (EIC) and (B) high resolution mass are shown for the putative intermediate **72** (Rt = 20.6 min).

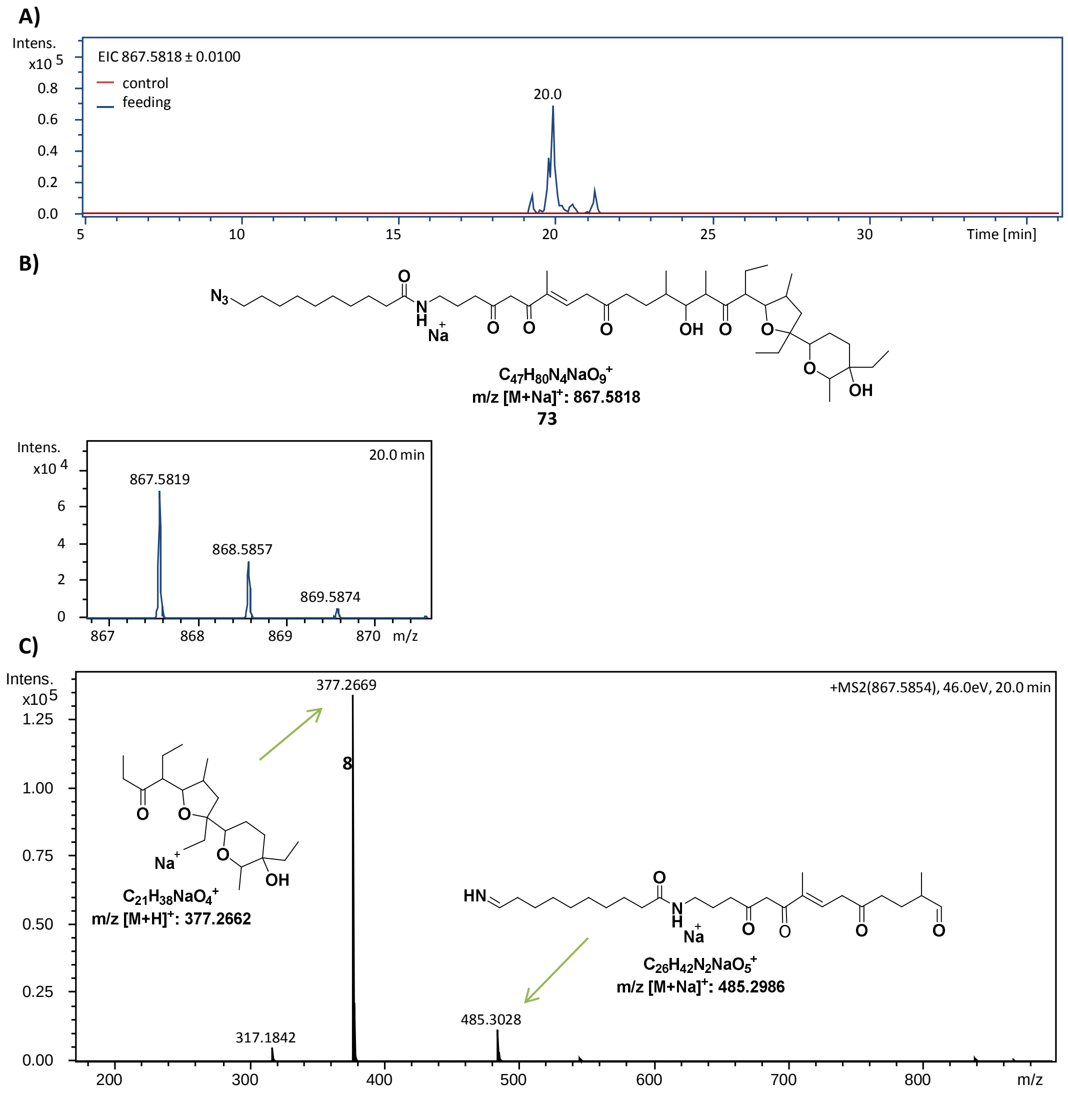


**Figure 64S:** (A) LC-HRMS analysis (MaXis Impact, method 2) of the organic extracts of *S. lasaliensis* ACP12 (S970A) grown in the absence (red) and in the presence (blue) of **4** (final concentration 4 mM): $[M+H]^+$ extracted ion chromatogram (EIC), (B) high resolution mass are shown for the putative intermediate **73** (Rt = 20.0 min) and (C) fragmentation of **73** with putative fragment structural assignment.

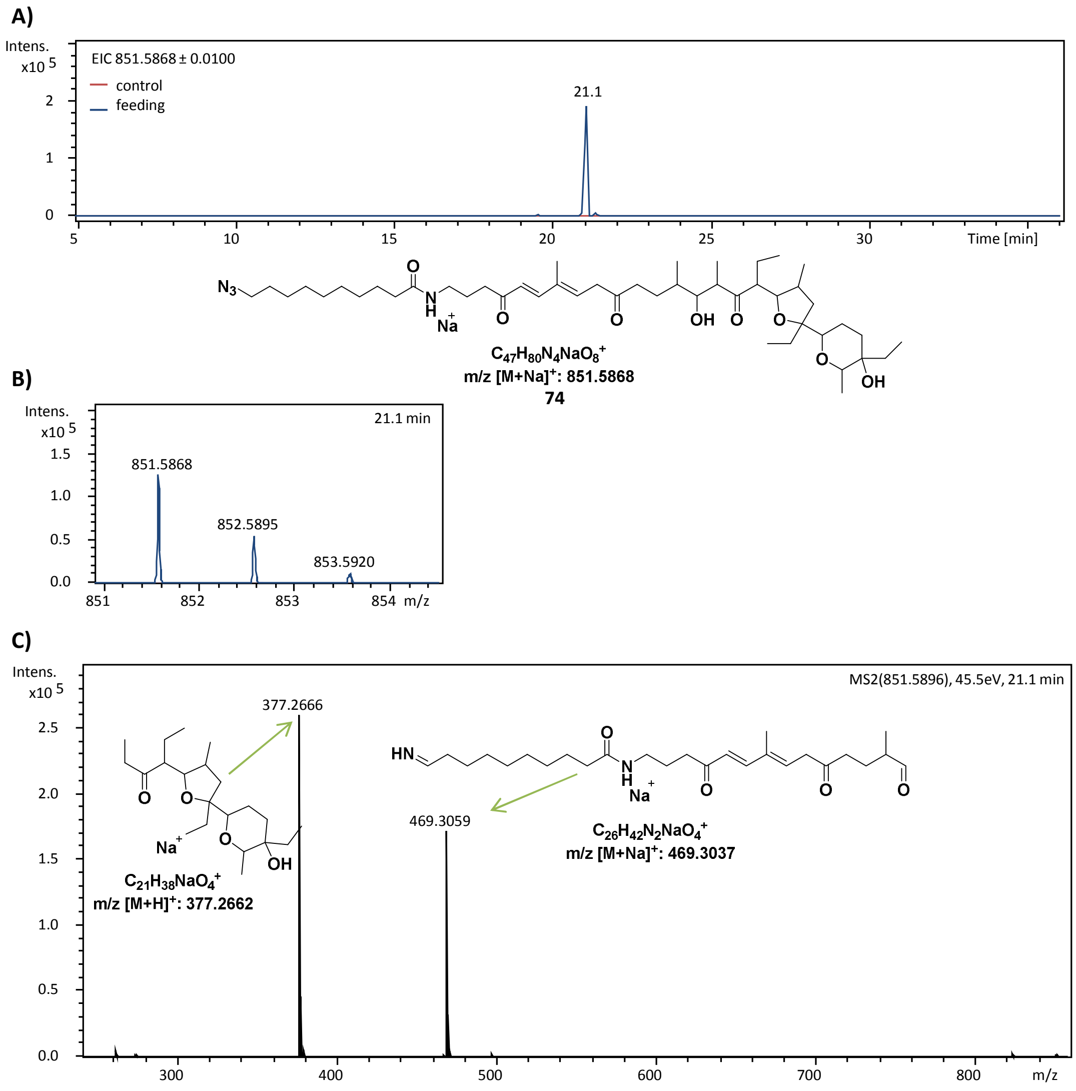


**Figure 65S:** (A) LC-HRMS analysis (MaXis Impact, method 2) of the organic extracts of *S. lasaliensis* ACP12 (S970A) grown in the absence (red) and in the presence (blue) of **4** (final concentration 4 mM): $[M+H]^+$ extracted ion chromatogram (EIC), (B) high resolution mass are shown for the putative intermediate **74** (Rt = 21.1 min) and (C) fragmentation of **74** with putative fragment structural assignment.

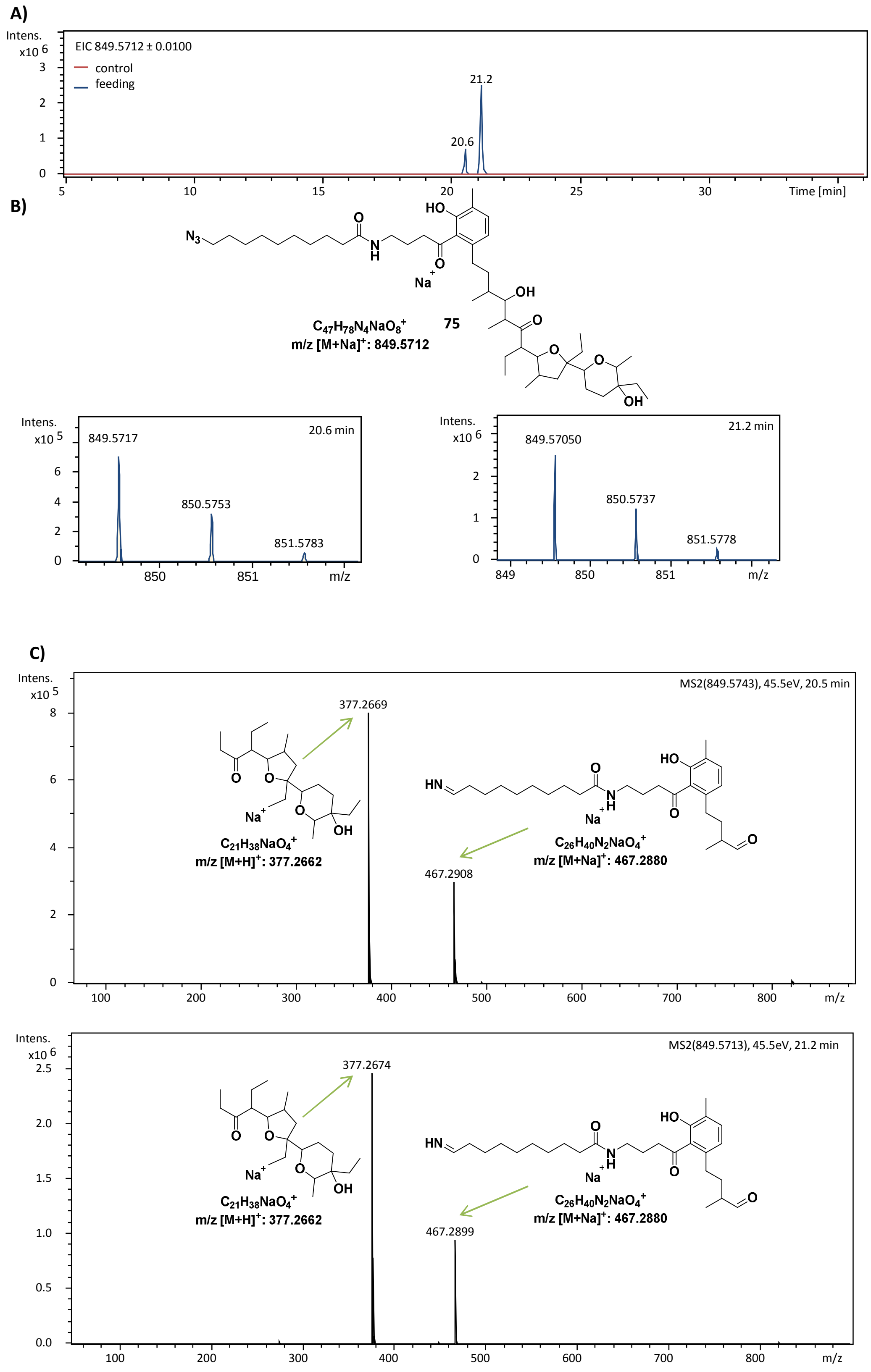


**Figure 66S:** (A) LC-HRMS analysis (MaXis Impact, method 2) of the organic extracts of *S. lasaliensis* ACP12 (S970A) grown in the absence (red) and in the presence (blue) of **4** (final concentration 4 mM): $[M+H]^+$ extracted ion chromatogram (EIC), (B) high resolution mass are shown for the putative intermediate **75** (Rt = 20.6 and 21.2 min) and (C) fragmentation of **75** with putative fragment structural assignment. Double peaks may arise from isomerisation (currently under investigation).

## 2.5. Staudinger-phosphite derivatisation of azido intermediates

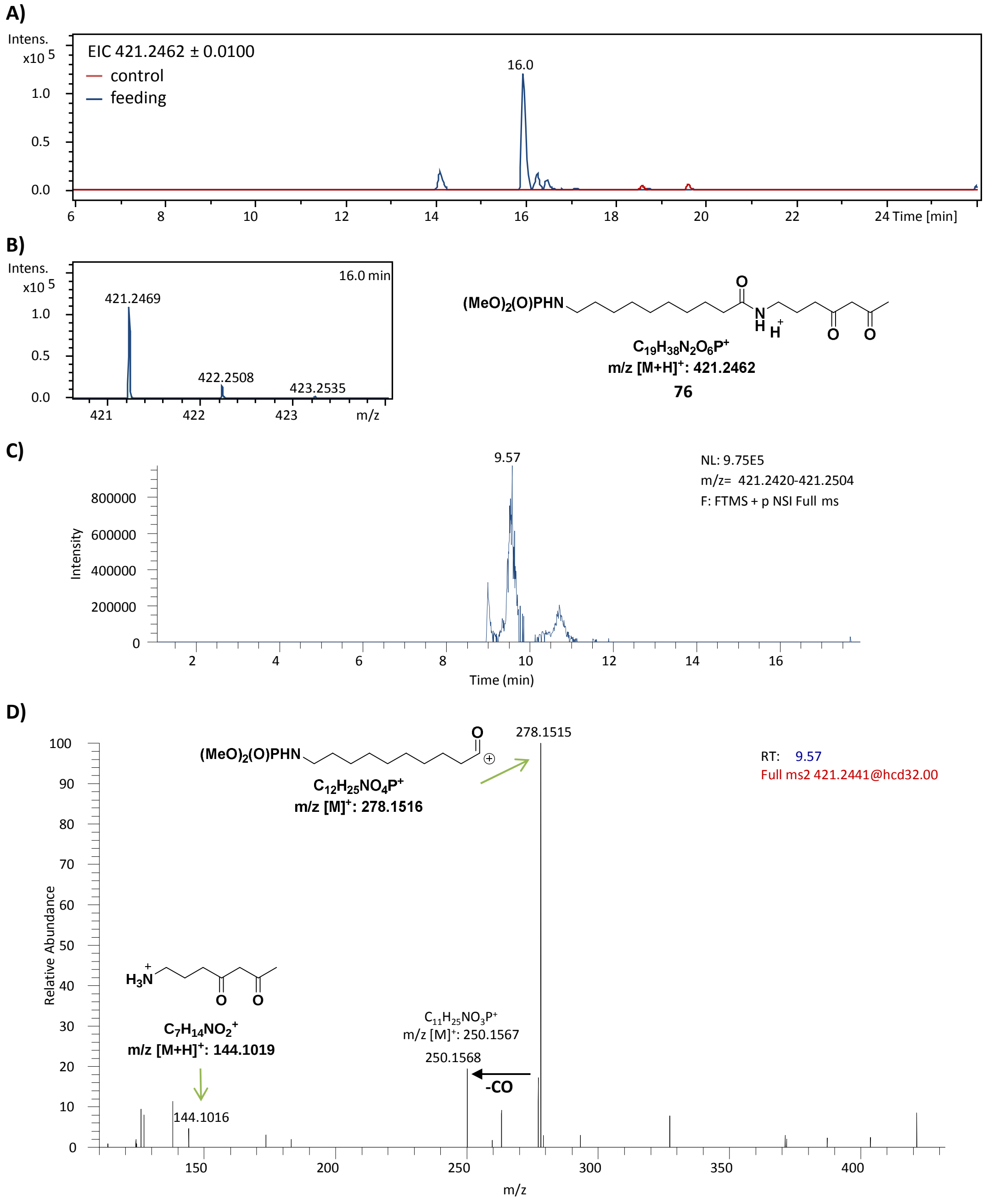


**Figure 67S:** (A) LC-HRMS analysis (MaXis Impact, method 2) of the organic extracts of *S. lasaliensis* ACP12 (S970A) grown in the absence (red) and in the presence (blue) of **4** after treatment with trimethyl phosphite: $[M+H]^+$ extracted ion chromatogram (EIC) and (B) high resolution mass are shown for the putative intermediate **76** (Rt = 16.0 min). (C) LC-HRMS analysis (Orbitrap Fusion) of the organic extracts of *S. lasaliensis* ACP12 (S970A) grown in the presence of **4** after treatment with trimethyl phosphite (final concentration 4 mM)**:** EIC (Rt = 9.57 min) and (D) fragmentation of **76** with putative fragment structural assignment.

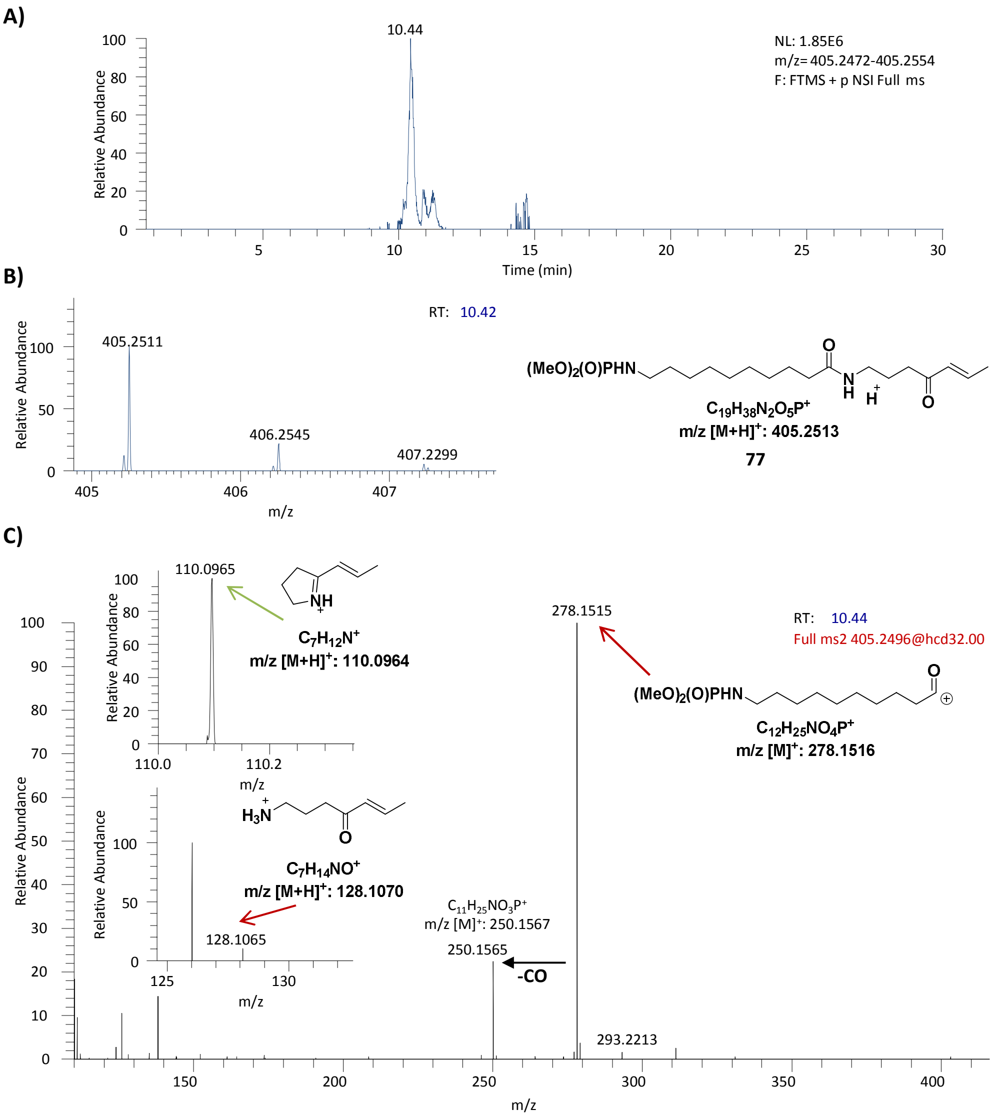


**Figure 68S:** (A) LC-HRMS analysis (Orbitrap Fusion) of the organic extracts of *S. lasaliensis* ACP12 (S970A) grown in the in the presence of **4** after treatment with trimethyl phosphite: $[M+H]^+$ extracted ion chromatogram (EIC) (Rt = 10.44 min min), (B) high resolution mass and (C) fragmentation of putative intermediate **77** with putative fragment structural assignment are shown.

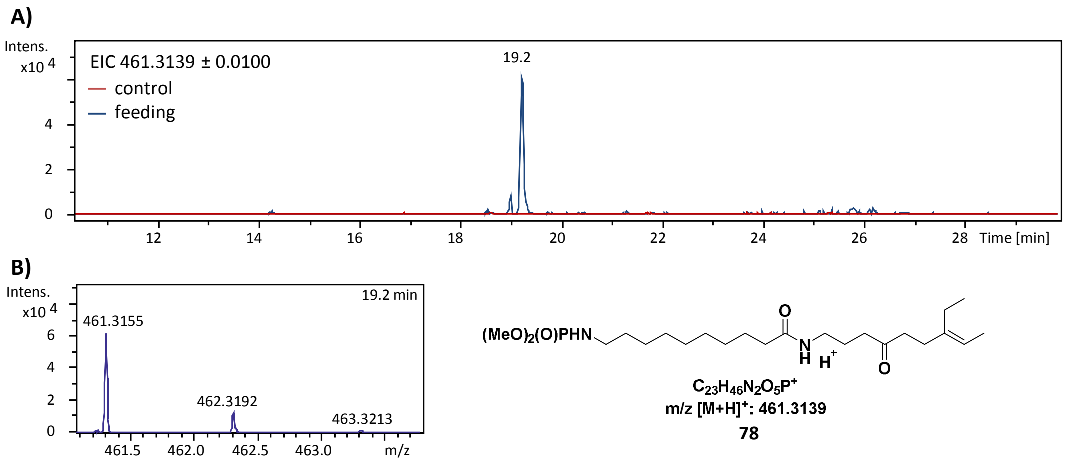

**D)**

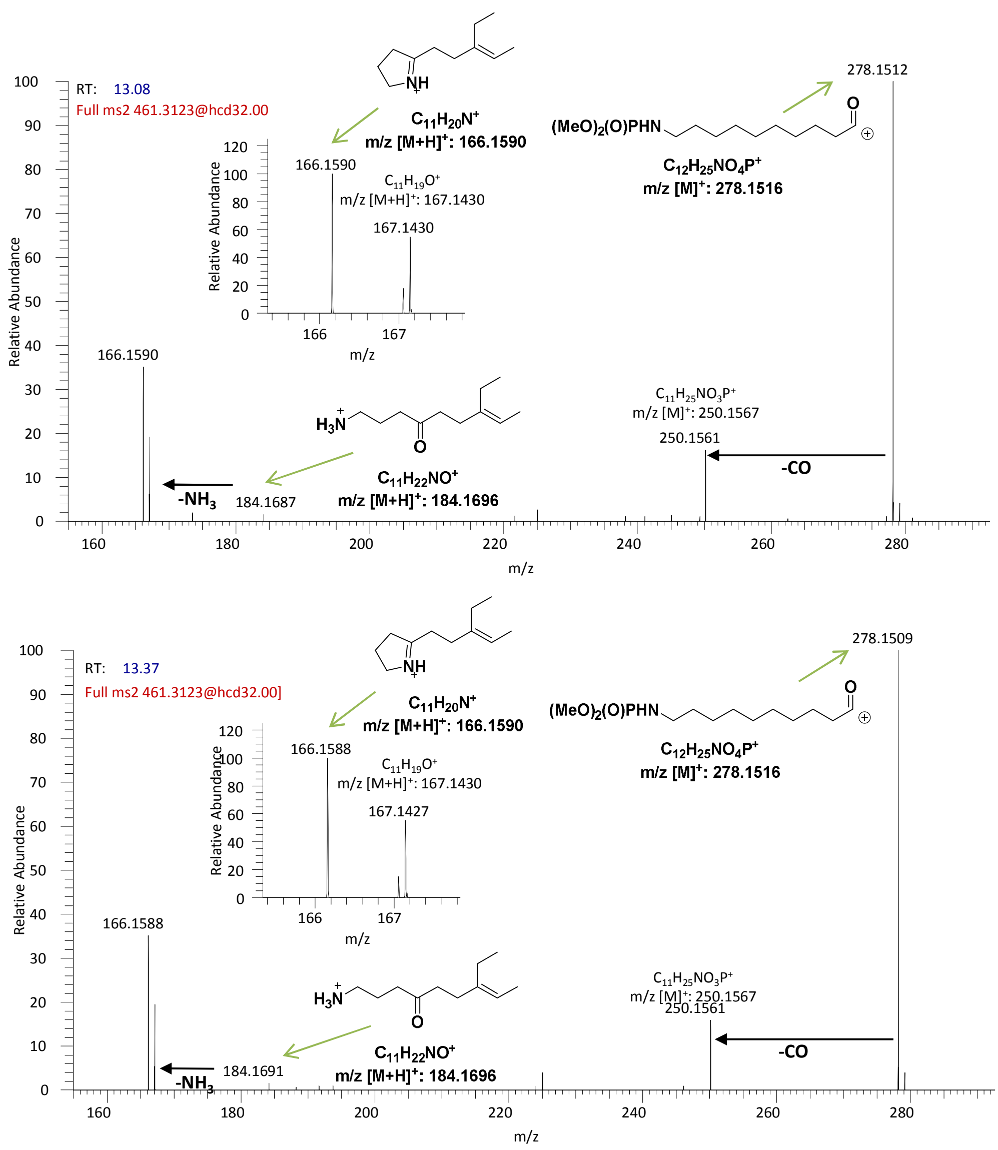


**Figure 69S:** (A) LC-HRMS analysis (MaXis Impact, method 2) of the organic extracts of *S. lasaliensis* ACP12 (S970A) grown in the absence (red) and in the presence (blue) of **4** after treatment with trimethyl phosphite: $[M+H]^+$ extracted ion chromatogram (EIC) and (B) high resolution mass are shown for the putative intermediate **78** (Rt = 19.2 min). (C) LC-HRMS analysis (Orbitrap Fusion) of the organic extracts of *S. lasaliensis* ACP12 (S970A) grown in the presence of **4** after treatment with trimethyl phosphite (final concentration 4 mM)**:** EIC (Rt = 13.09 and 13.37 min) and (D) fragmentation of **78** with putative fragment structural assignment.

**A)**

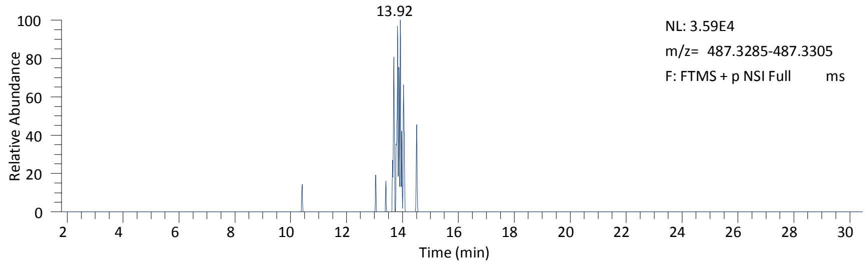


**B)**

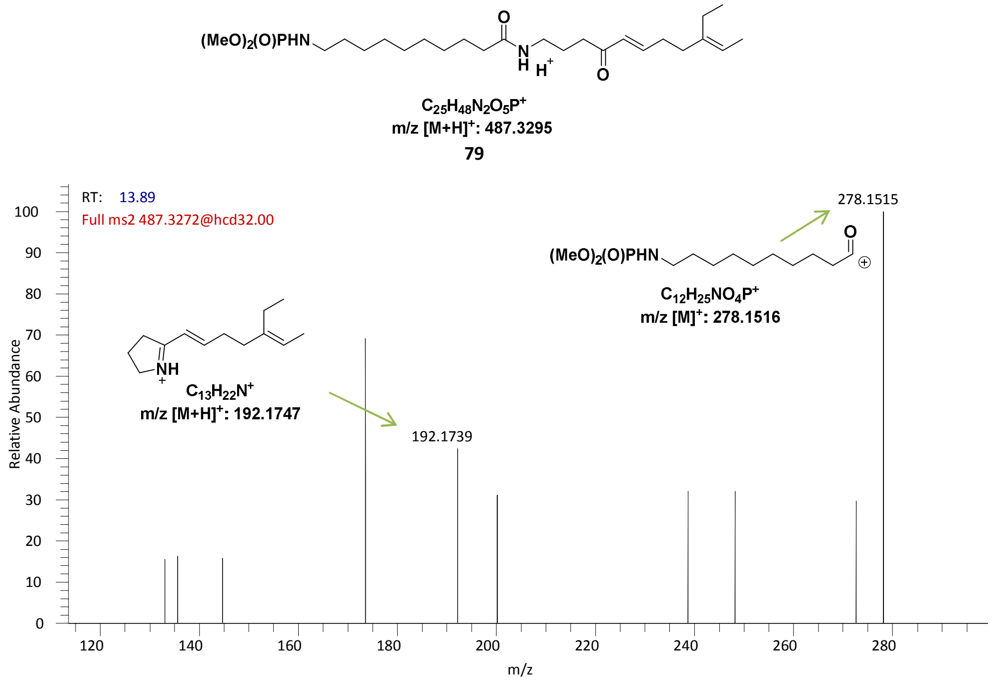


**Figure 70S:** (A) LC-HRMS analysis (Orbitrap Fusion) of the organic extracts of *S. lasaliensis* ACP12 (S970A) grown in the presence of **4** after treatment with trimethyl phosphite: $[M+H]^+$ extracted ion chromatogram (EIC) (Rt = 13.92 min min) and (B) fragmentation of putative intermediate **79** with putative fragment structural assignment are shown.

**A)**

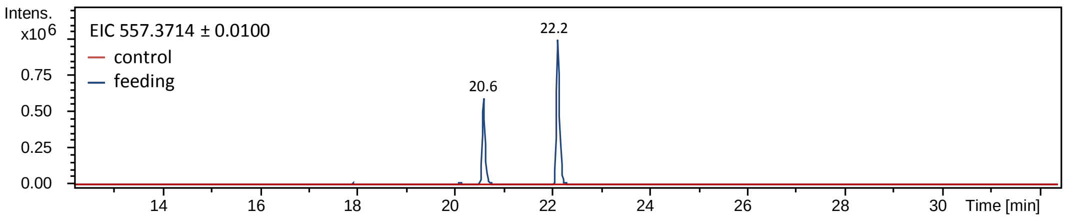


**B)**

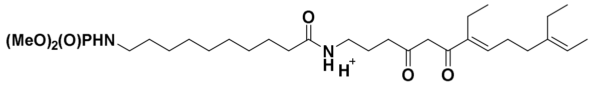


$C_{29}H_{54}N_2O_6P^+$

**m/z [M+H]$^+$: 557.3714**

**80**

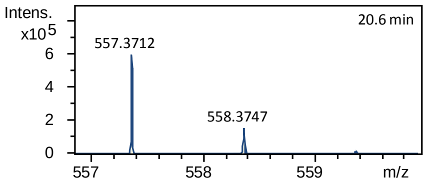


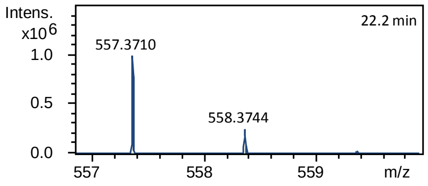

**C)**

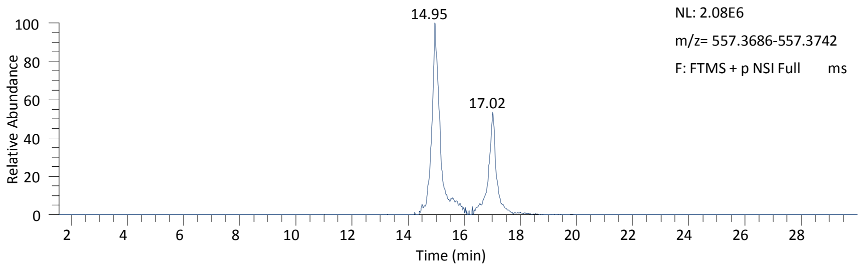


**D)**

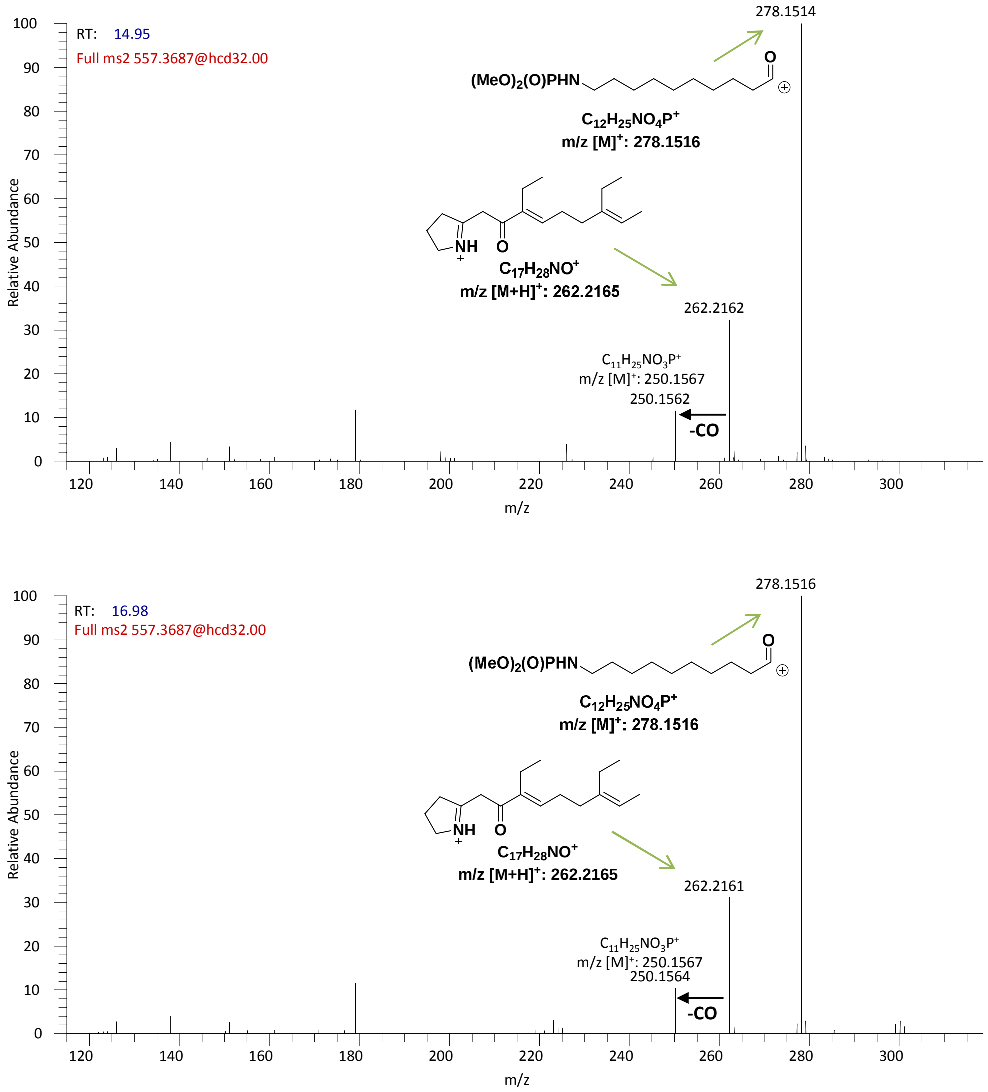


**Figure 71S:** (A) LC-HRMS analysis (MaXis Impact, method 2) of the organic extracts of *S. lasaliensis* ACP12 (S970A) grown in the absence (red) and in the presence (blue) of **4** after treatment with trimethyl phosphite: $[M+H]^+$ extracted ion chromatogram (EIC) and (B) high resolution mass are shown for the putative intermediate **80** (Rt = 20.6 and 22.2 min). (C) LC-HRMS analysis (Orbitrap Fusion) of the organic extracts of *S. lasaliensis* ACP12 (S970A) grown in the presence of **4** after treatment with trimethyl phosphite (final concentration 4 mM)**:** EIC (Rt = 14.95 and 17.02 min) and (D) fragmentation of **80** with putative fragment structural assignment. Double peaks may arise from isomerisation (currently under investigation).

**A)**

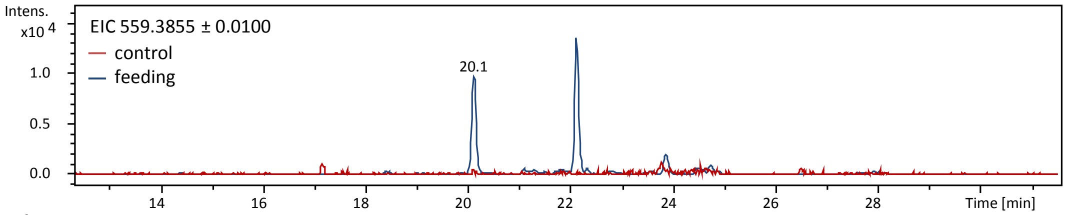


**B)**

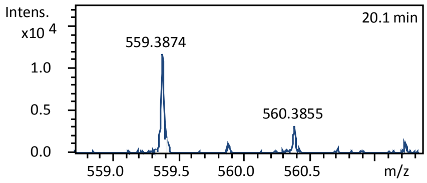


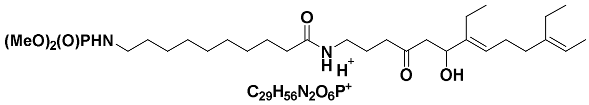


$C_{29}H_{56}N_2O_6P^+$

m/z $[M+H]^+$: 559.3871

**81**

**C)**

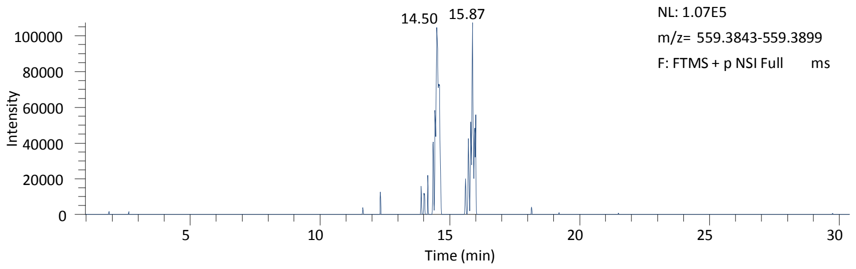


**D)**

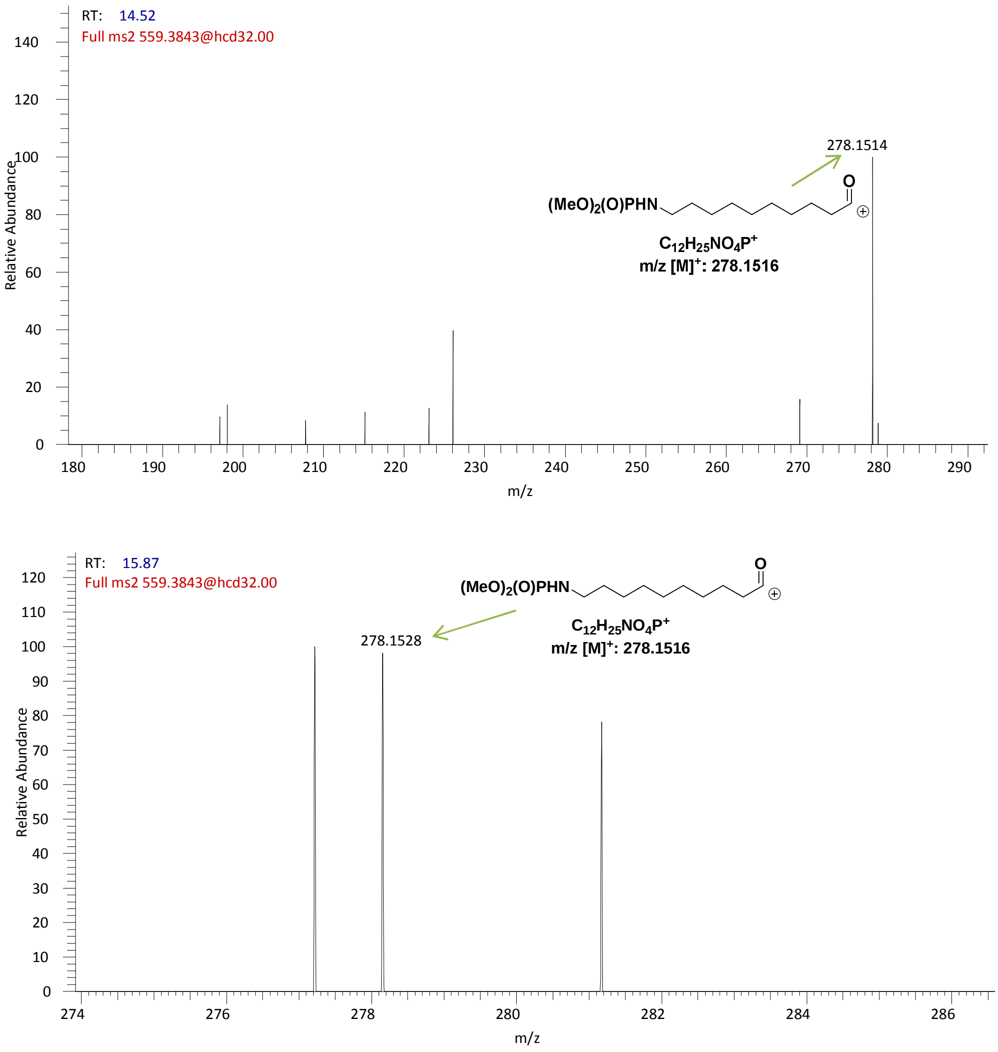


**Figure 72S:** (A) LC-HRMS analysis (MaXis Impact, method 2) of the organic extracts of *S. lasaliensis* ACP12 (S970A) grown in the absence (red) and in the presence (blue) of **4** after treatment with trimethyl phosphite: $[M+H]^+$ extracted ion chromatogram (EIC) and (B) high resolution mass are shown for the putative intermediate **81** (Rt = 20.1 min). (C) LC-HRMS analysis (Orbitrap Fusion) of the organic extracts of *S. lasaliensis* ACP12 (S970A) grown in the presence of **4** after treatment with trimethyl phosphite (final concentration 4 mM)**:** EIC (Rt = 14.50 and 15.87 min) and (D) fragmentation of **81** with putative fragment structural assignment. Double peaks may arise from isomerisation (currently under investigation).

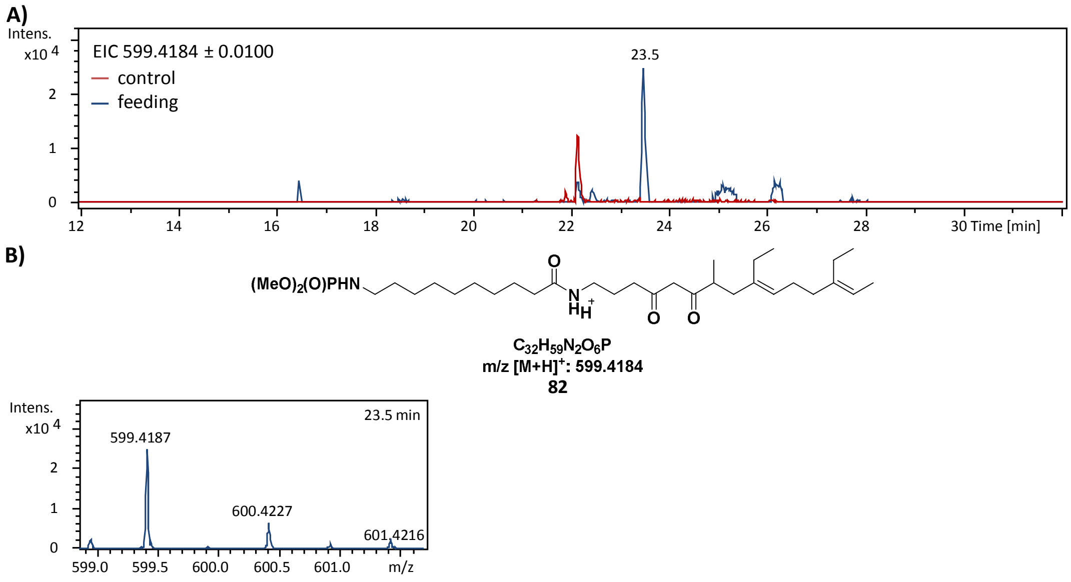


**Figure 73S:** (A) LC-HRMS analysis (MaXis Impact, method 2) of the organic extracts of *S. lasaliensis* ACP12 (S970A) grown in the absence (red) and in the presence (blue) of **4** treated with trimethyl phosphite: $[M+H]^+$ extracted ion chromatogram (EIC) and (B) high resolution mass are shown for the putative intermediate **82** (Rt = 23.5 min).

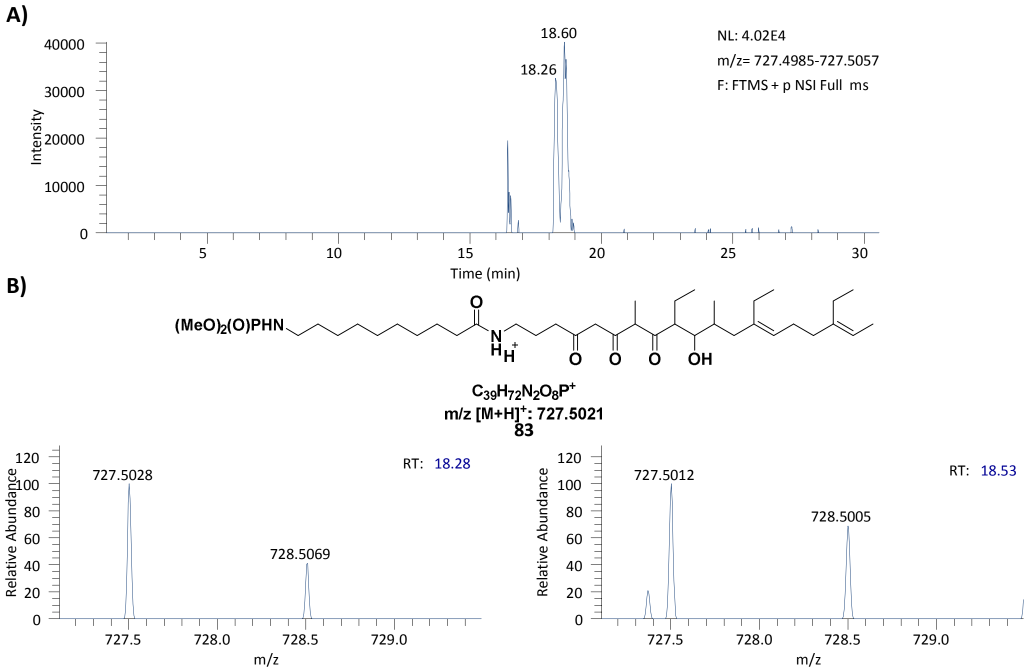

**C)**

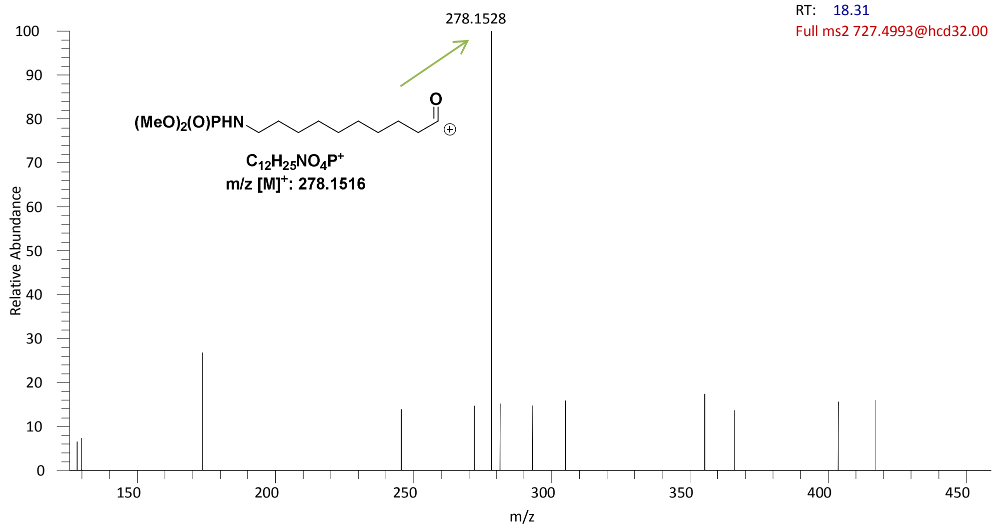


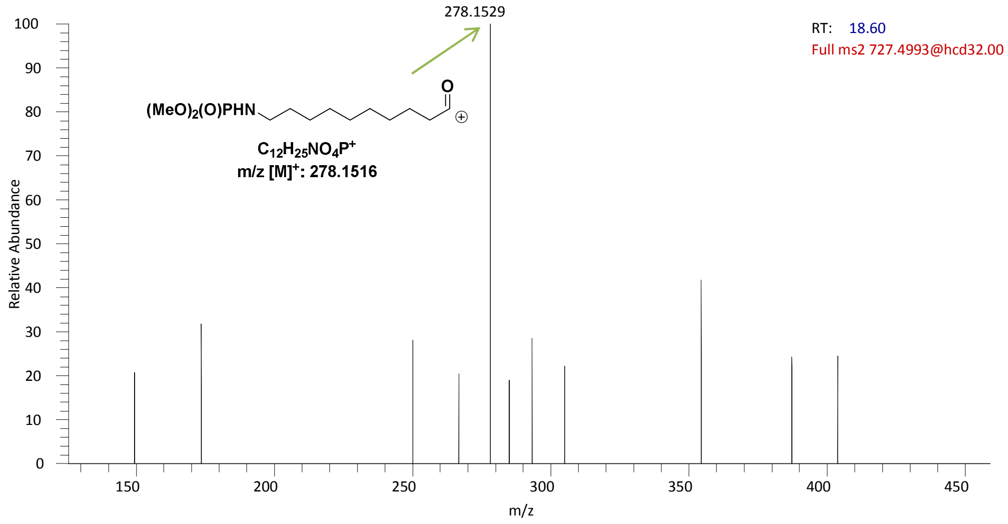


**Figure 74S:** (A) LC-HRMS analysis (Orbitrap Fusion) of the organic extracts of *S. lasaliensis* ACP12 (S970A) grown in the in the presence of **4** after treatment with trimethyl phosphite: $[M+H]^+$ extracted ion chromatogram (EIC) (Rt = 18.26 and 18.60 min), (B) high resolution mass and (C) fragmentation of putative intermediate **83** with putative fragment structural assignment are shown. Double peaks may arise from intramolecular cyclisation or isomerisation (currently under investigation).

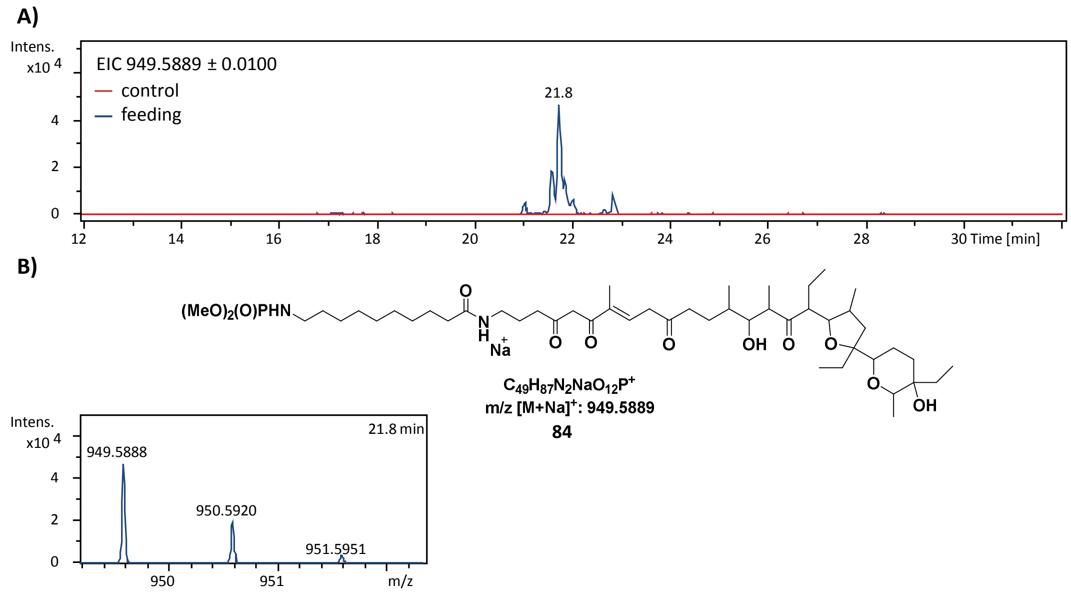


**Figure 75S:** (A) LC-HRMS analysis (MaXis Impact, method 2) of the organic extracts of *S. lasaliensis* ACP12 (S970A) grown in the absence (red) and in the presence (blue) of **4** treated with trimethyl phosphite (final concentration 4 mM): $[M+H]^+$ extracted ion chromatogram (EIC) and (B) high resolution mass are shown for the putative intermediate **84** (Rt = 21.8 min).

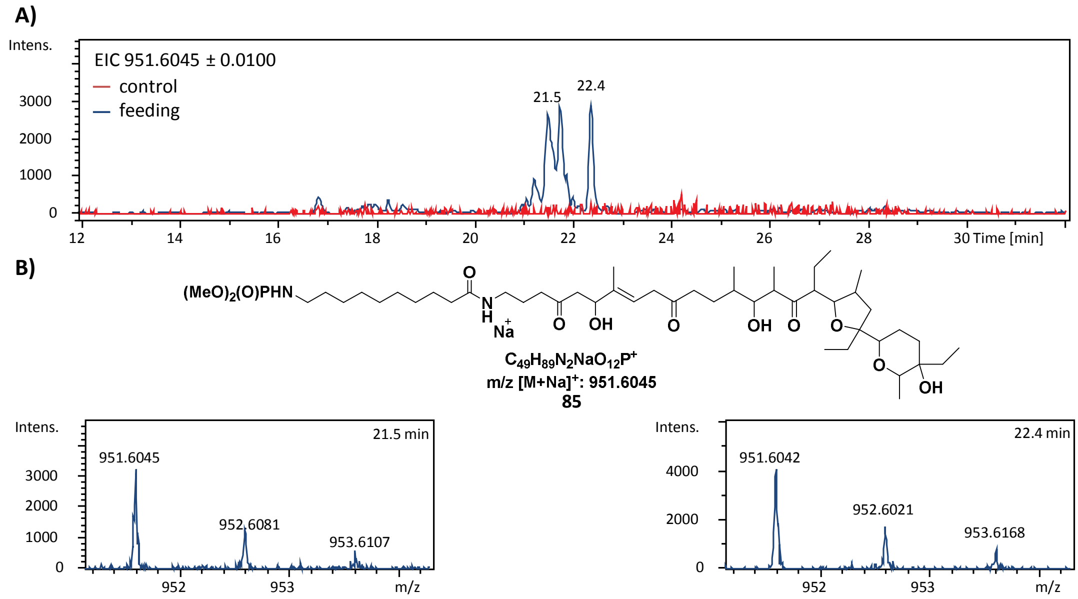


**Figure 76S:** (A) LC-HRMS analysis (MaXis Impact, method 2) of the organic extracts of *S. lasaliensis* ACP12 (S970A) grown in the absence (red) and in the presence (blue) of **4** treated with trimethyl phosphite (final concentration 4 mM): $[M+H]^+$ extracted ion chromatogram (EIC) and (B) high resolution mass are shown for the putative intermediate **85** (Rt = 21.5 and 22.4 min). Multiple peaks may arise from intramolecular cyclisation or isomerisation (currently under investigation).

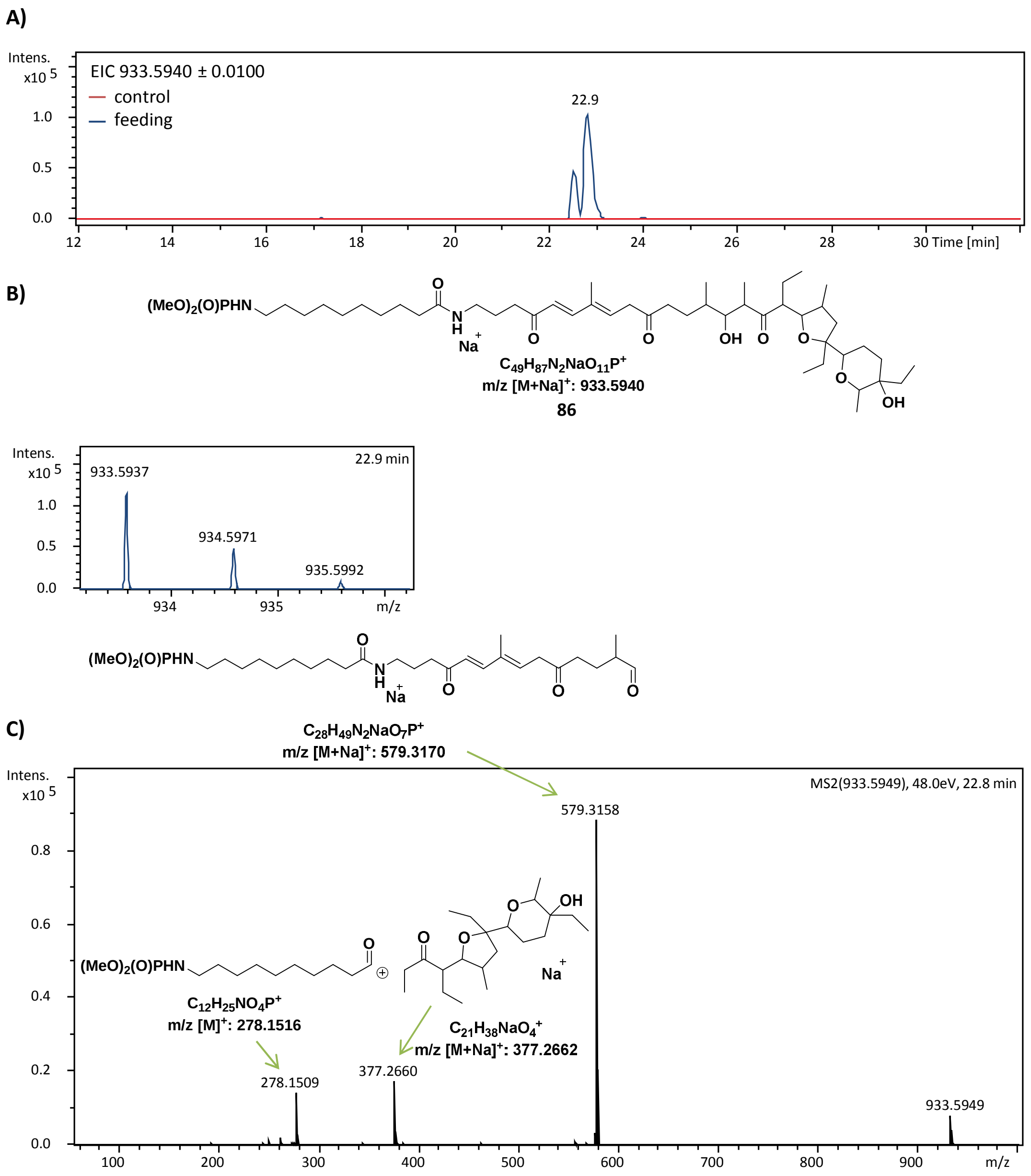


**Figure 77S:** (A) LC-HRMS analysis (MaXis Impact, method 2) of the organic extracts of *S. lasaliensis* ACP12 (S970A) grown in the absence (red) and in the presence (blue) of **4** treated with trimethyl phosphite (final concentration 4 mM): $[M+H]^+$ extracted ion chromatogram (EIC), (B) high resolution mass are shown for the putative intermediate **86** (Rt = 22.8 min) and (C) fragmentation of **86** with putative fragment structural assignment. Double peaks may arise from intramolecular cyclisation or isomerisation (currently under investigation).

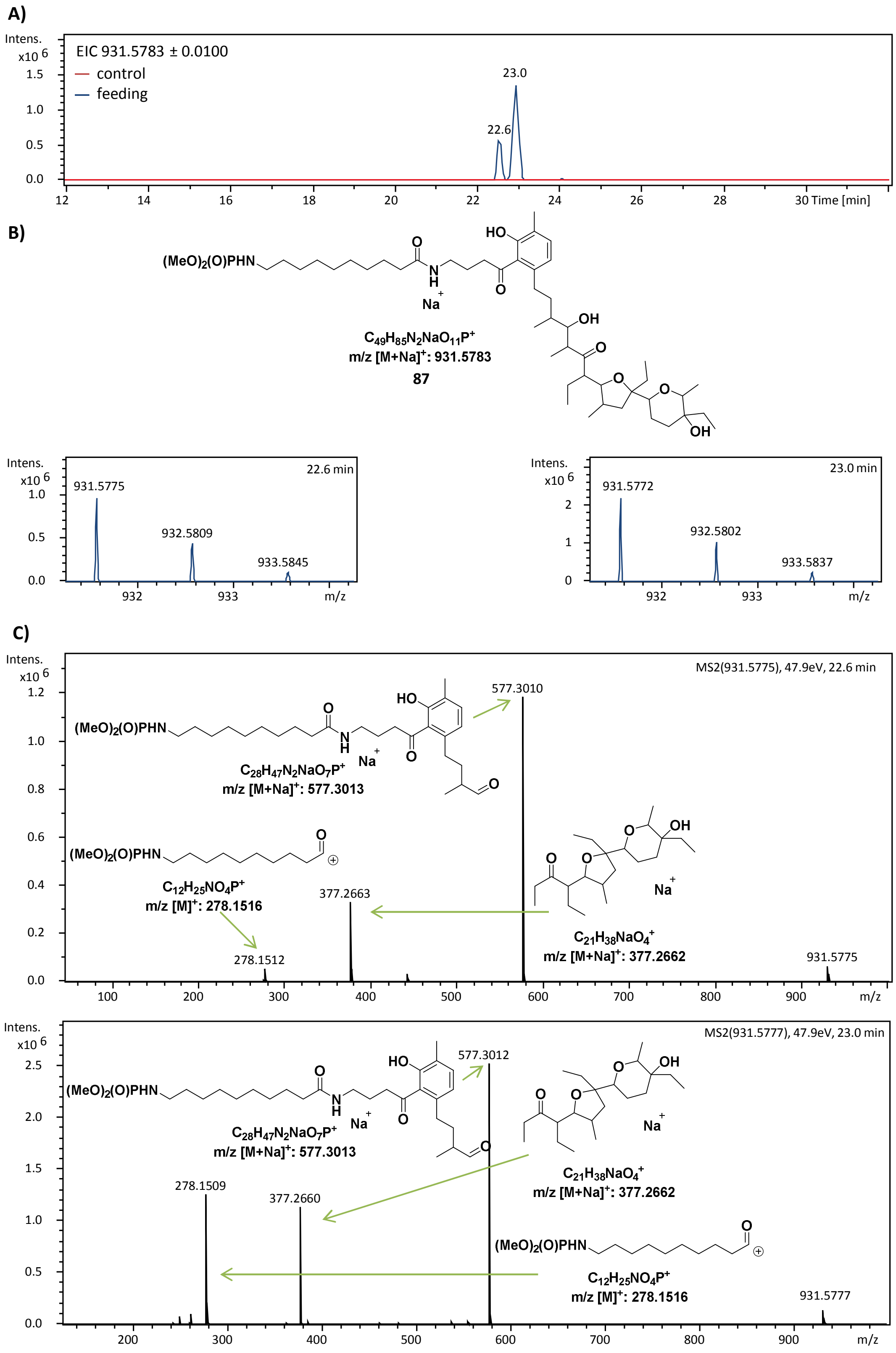


**Figure 78S:** (A) LC-HRMS analysis (MaXis Impact, method 2) of the organic extracts of *S. lasaliensis* ACP12 (S970A) grown in the absence (red) and in the presence (blue) of **4** treated with trimethyl phosphite (final concentration 4 mM): $[M+H]^+$ extracted ion chromatogram (EIC), (B) high resolution mass are shown for the putative intermediate **87** (Rt = 22.6 and 23.0 min) and (C) fragmentation of **87** with putative fragment structural assignment Double peaks may arise from isomerisation (currently under investigation).

# 3. Spectra

## 3.1. $^1$H- and $^{13}$C-NMR of compound 13a (400 MHz, $CDCl_3$)

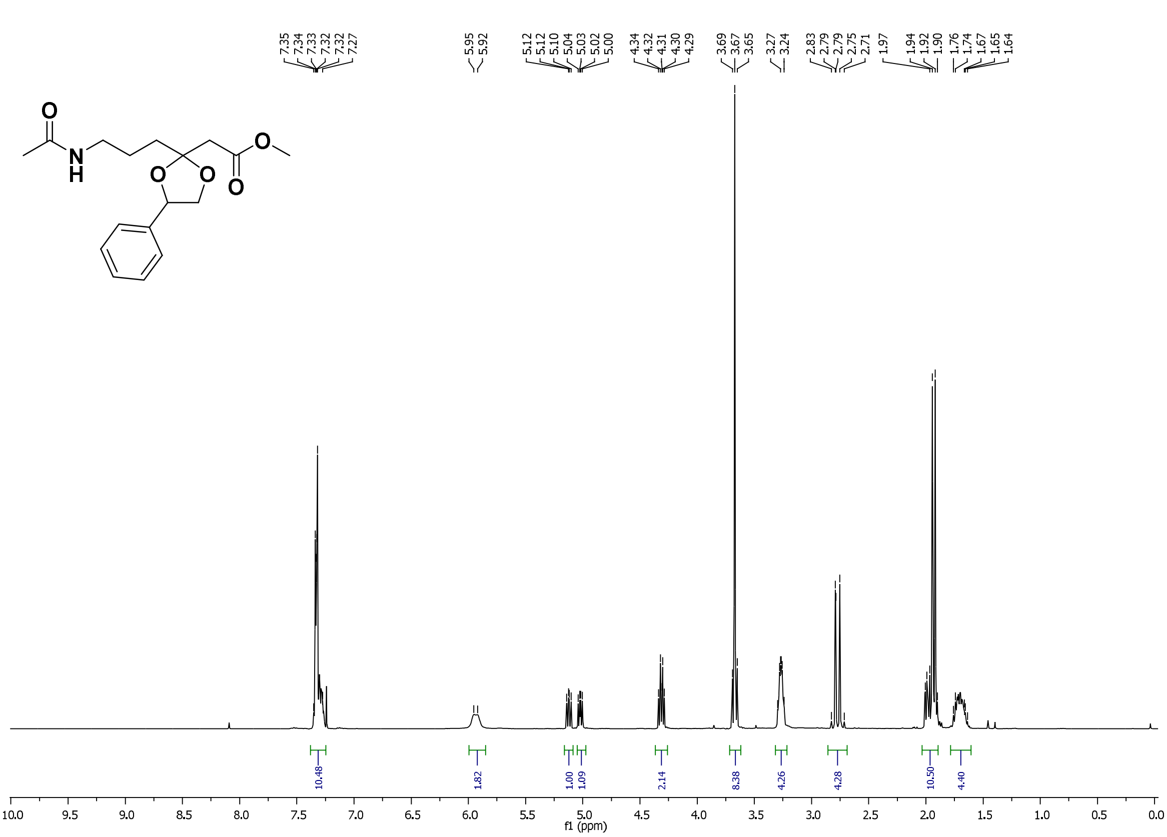


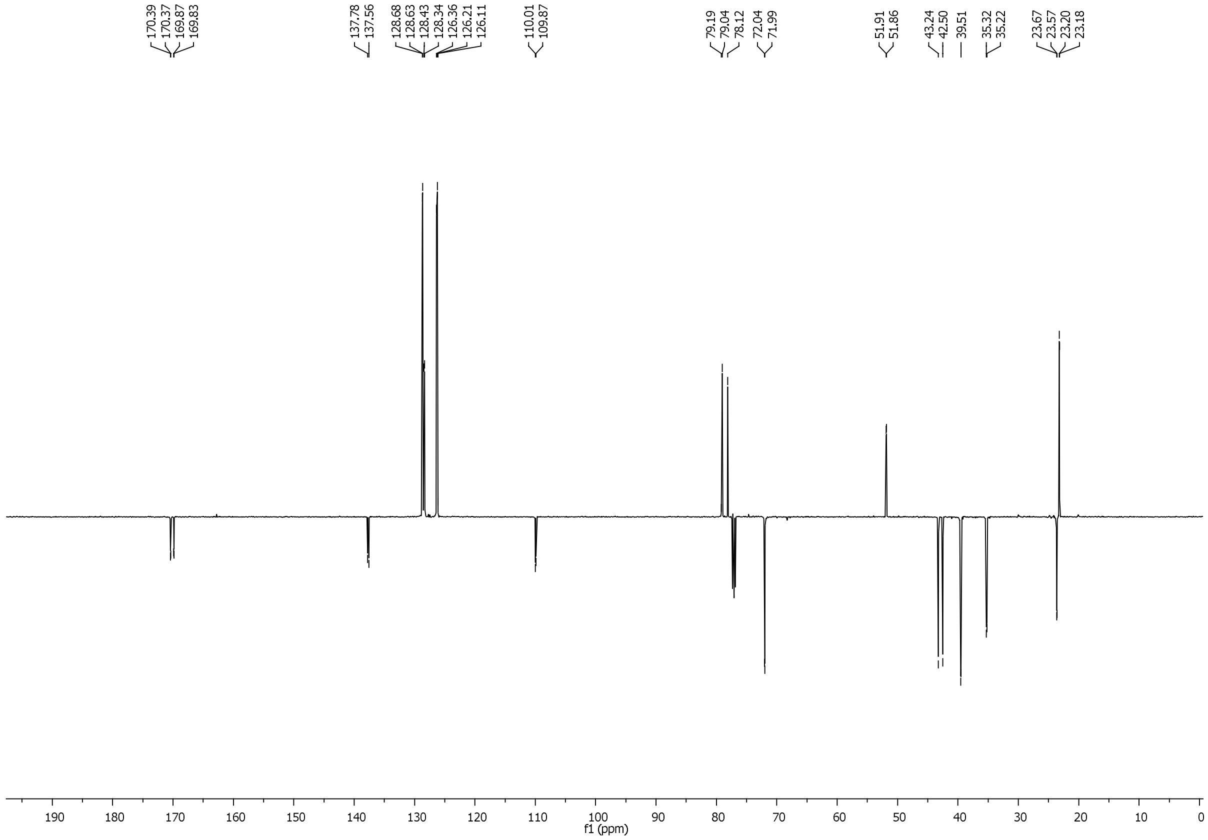

## 3.2. $^{1}$H- and $^{13}$C-NMR of compound 13b (400 MHz, $CDCl_3$)

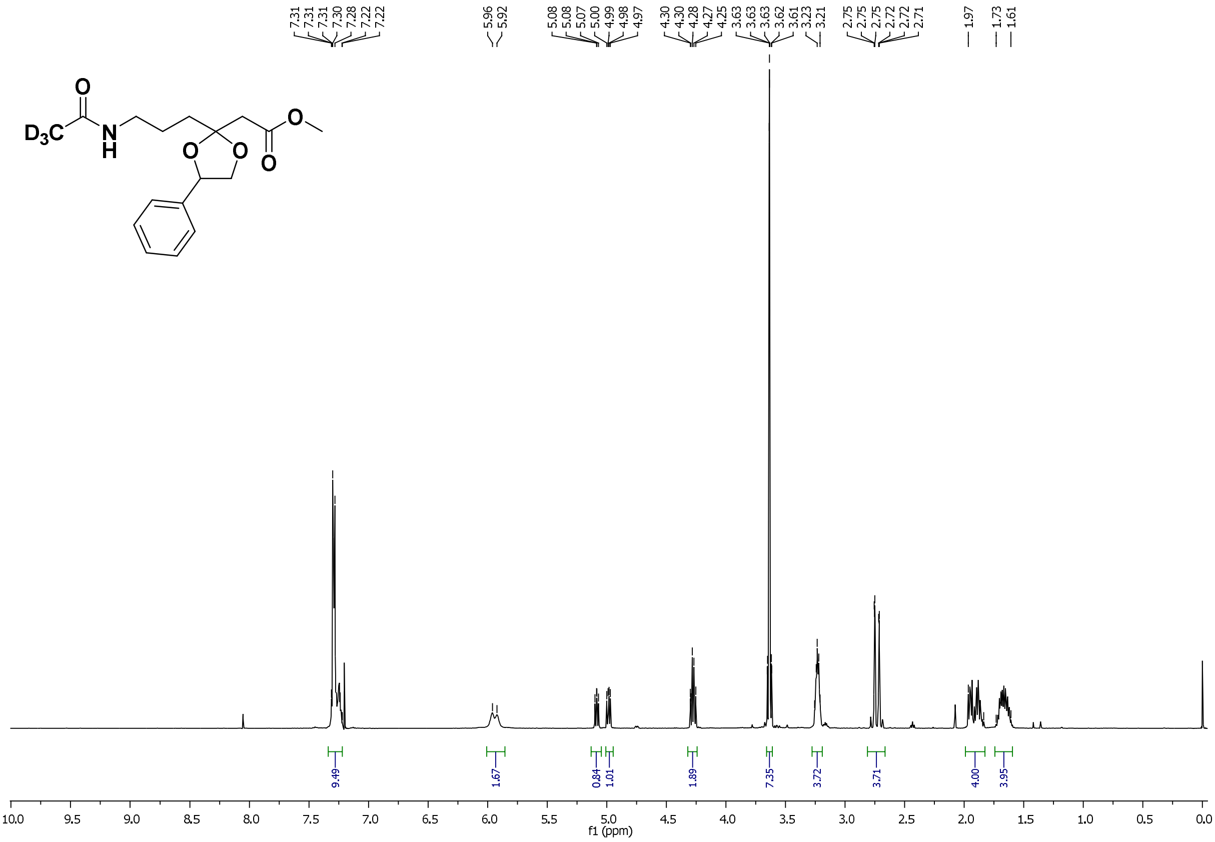


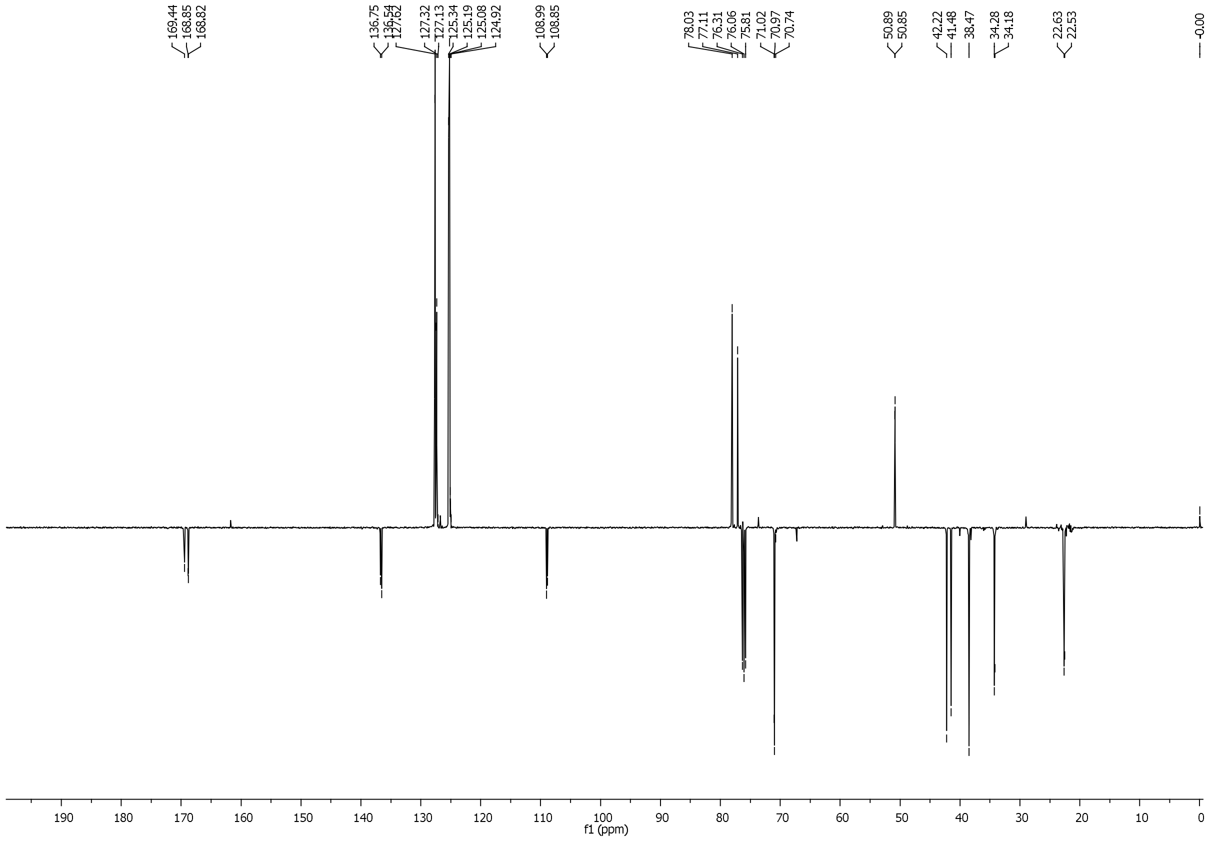

# 3.3. $^{1}$H- and $^{13}$C-NMR of compound 14a (400 MHz, $CDCl_3$)

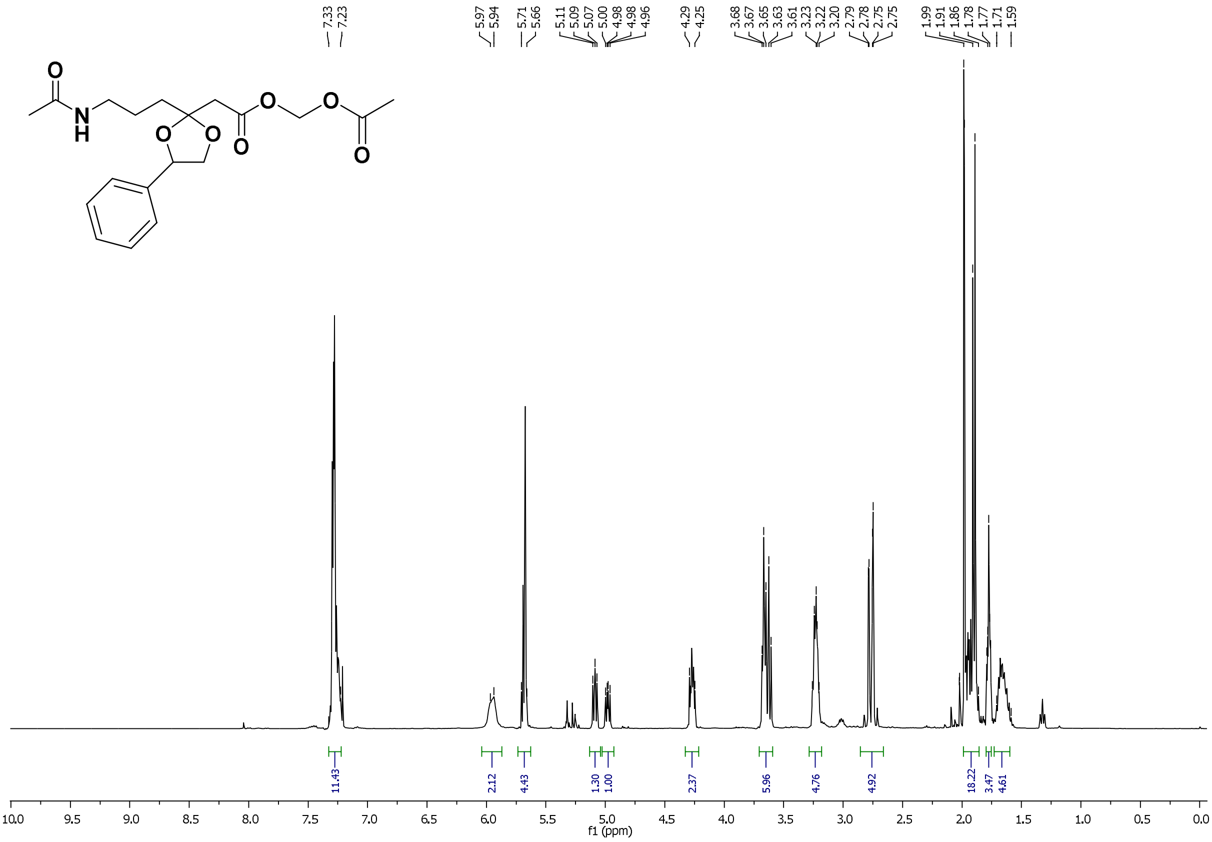


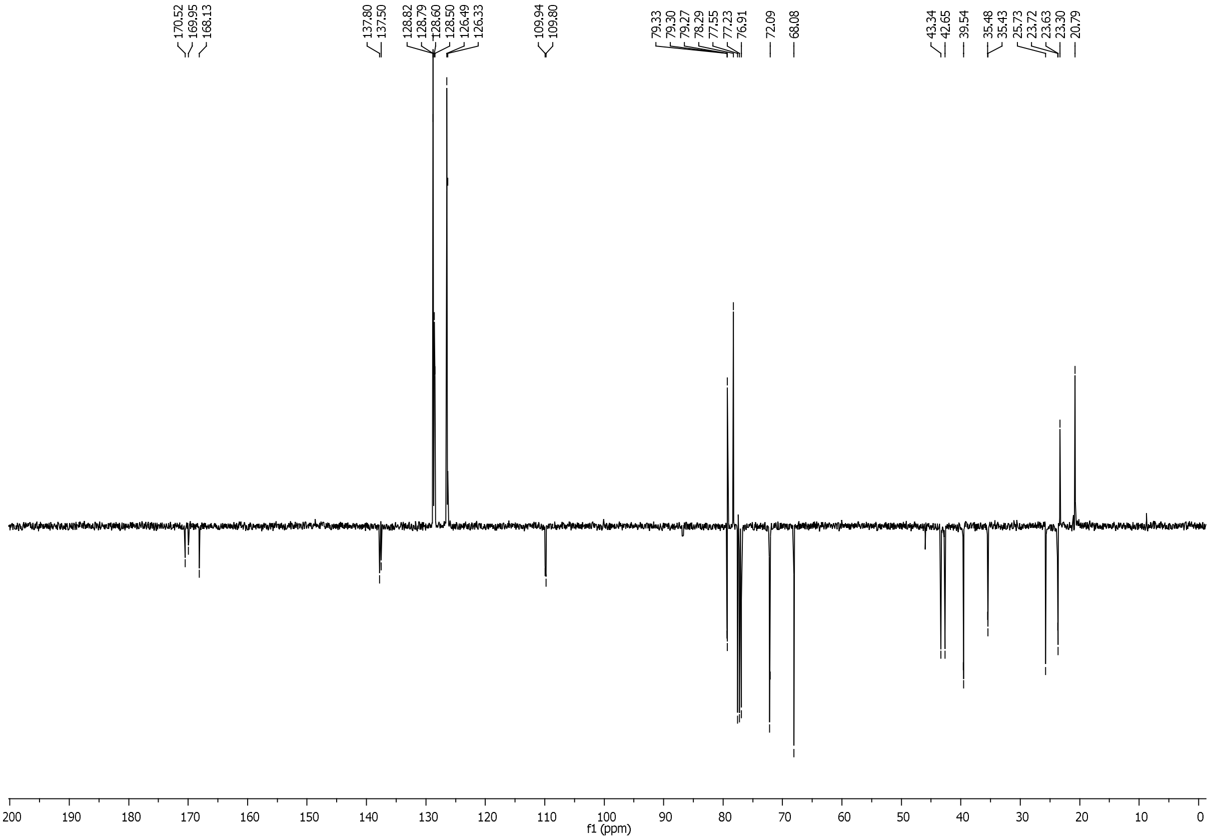

# 3.4. $^{1}$H- and $^{13}$C-NMR of compound 14b (400 MHz, $CDCl_3$)

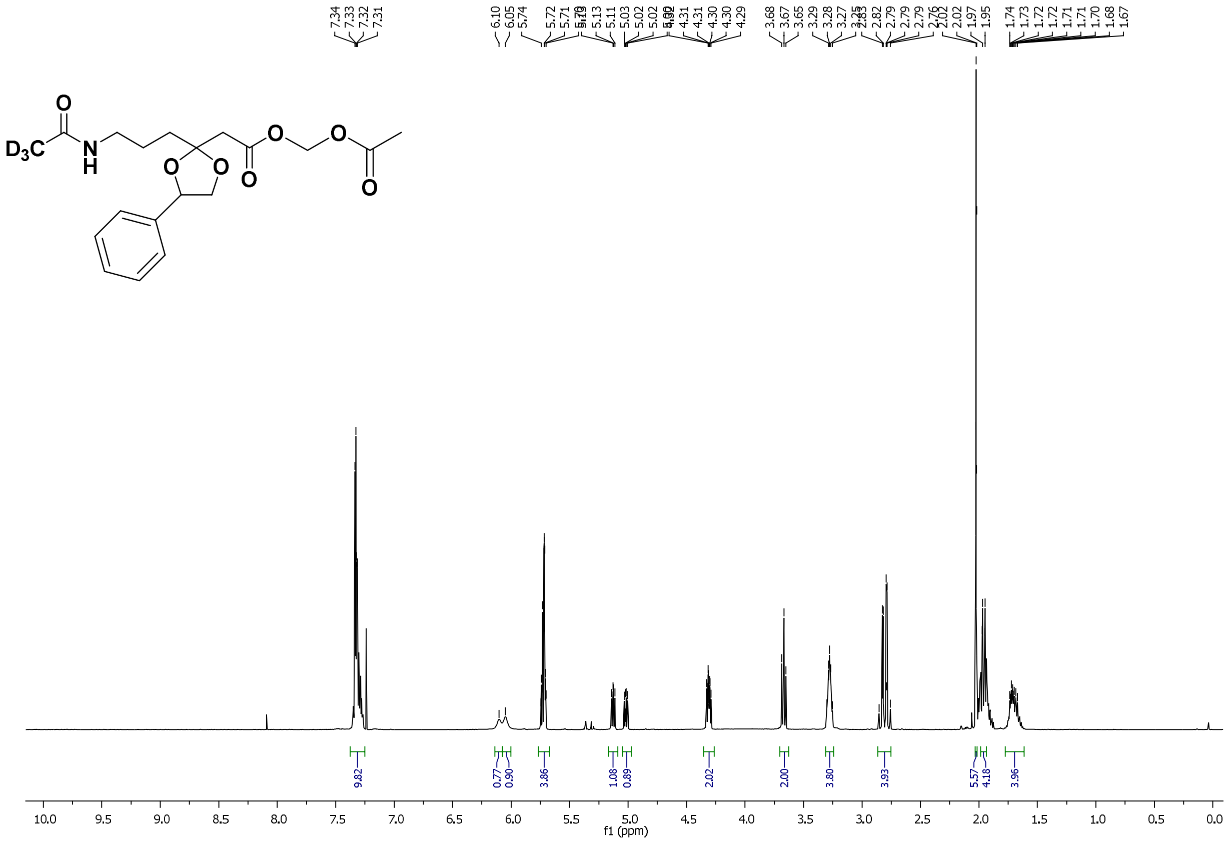

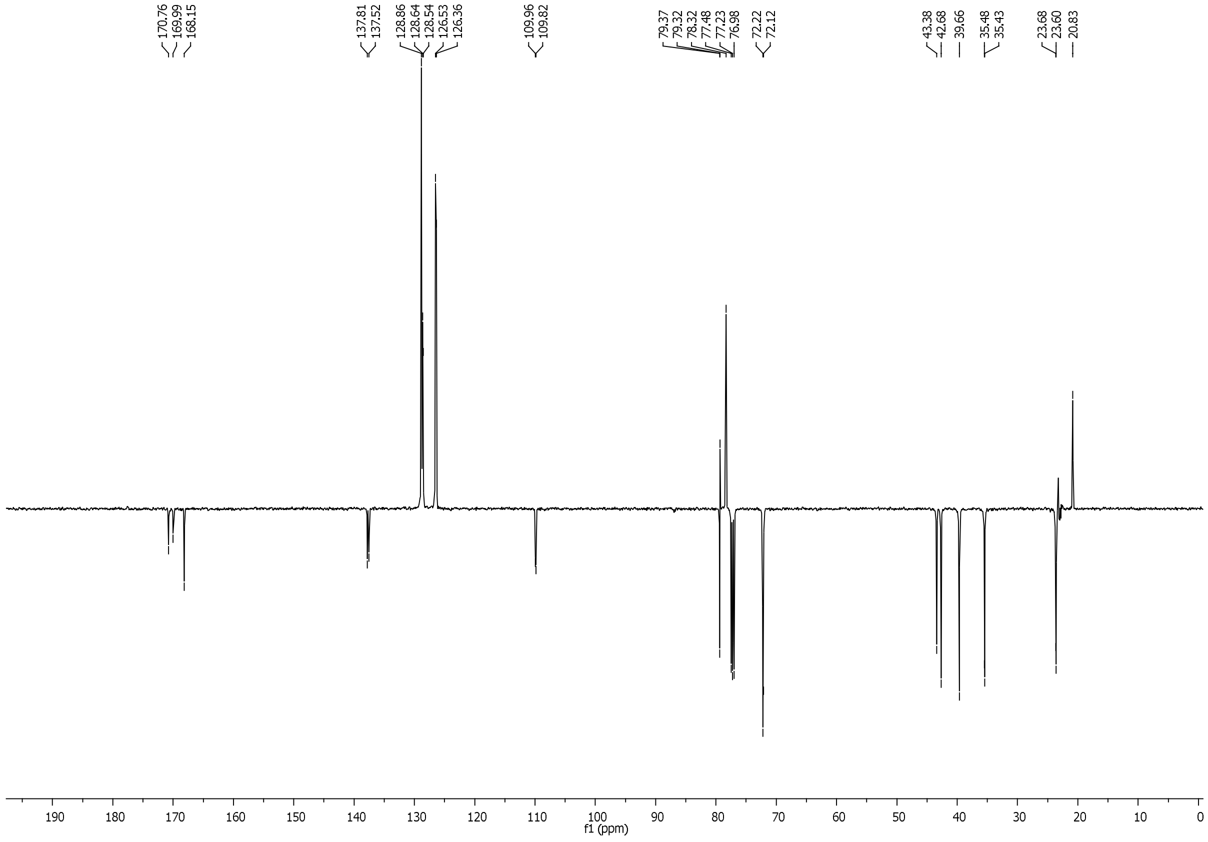

# 3.5. $^1$H- and $^{13}$C-NMR of compound 16a (400 MHz, $CDCl_3$)

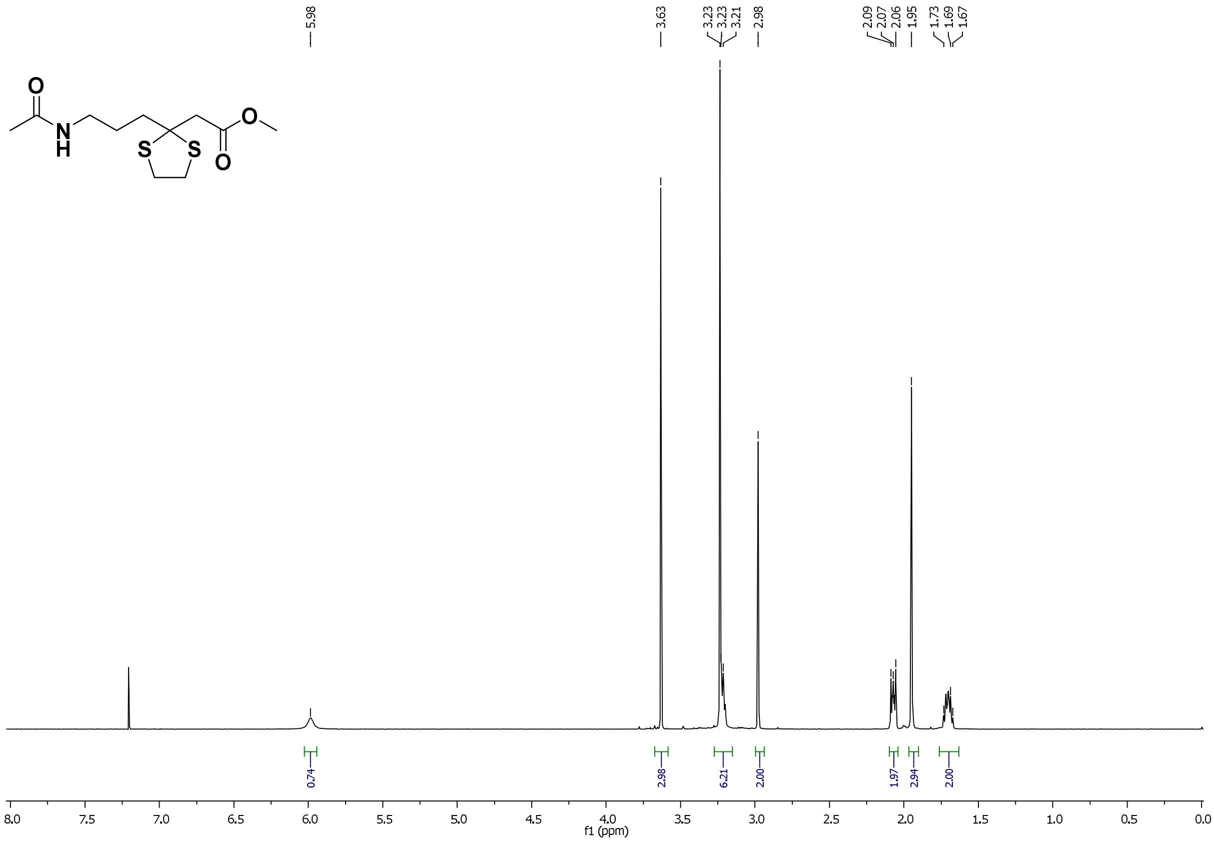


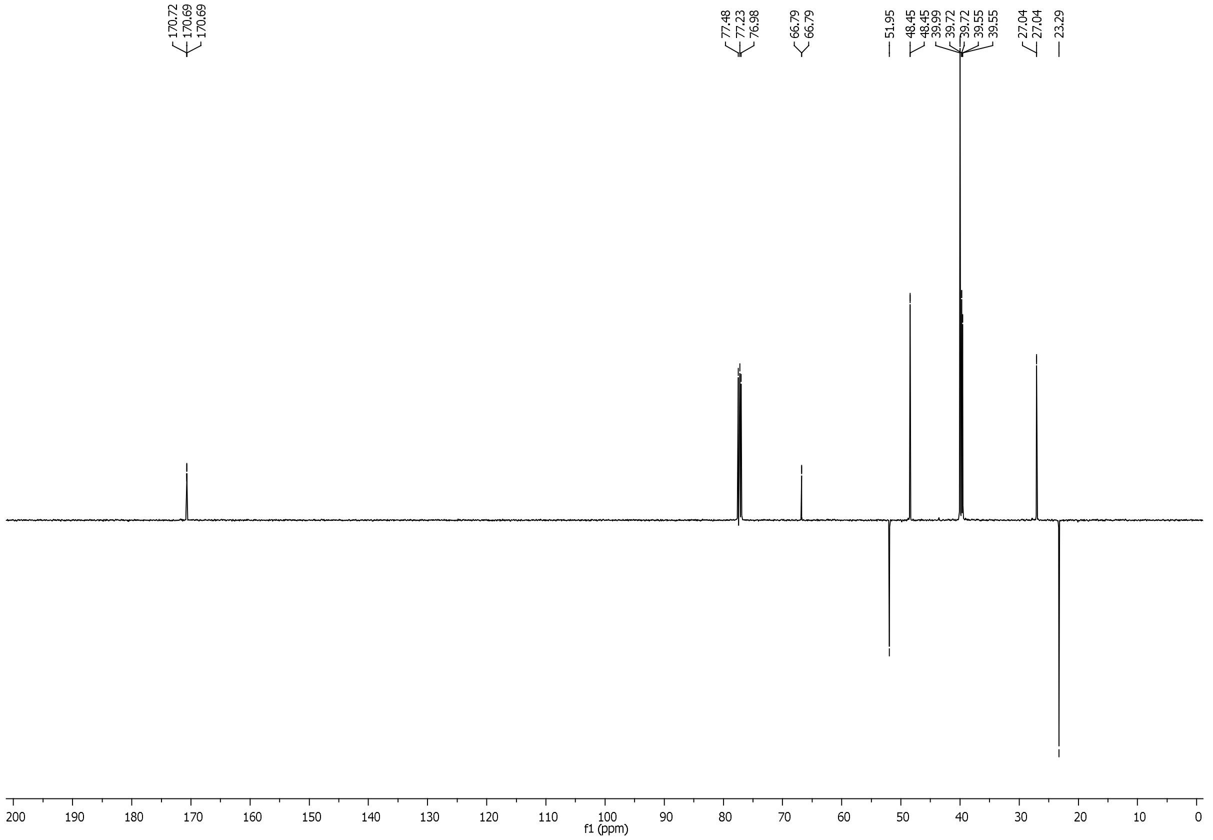

Ina Wilkening,* Silvia Gazzola,* Elena Riva, James S. Parascandolo, Lijiang Song and Manuela Tosin**

# 3.6. $^{1}$H- and $^{13}$C-NMR of compound 17a (400 MHz, $CDCl_3$)

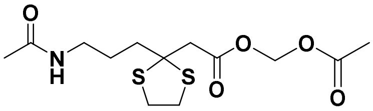

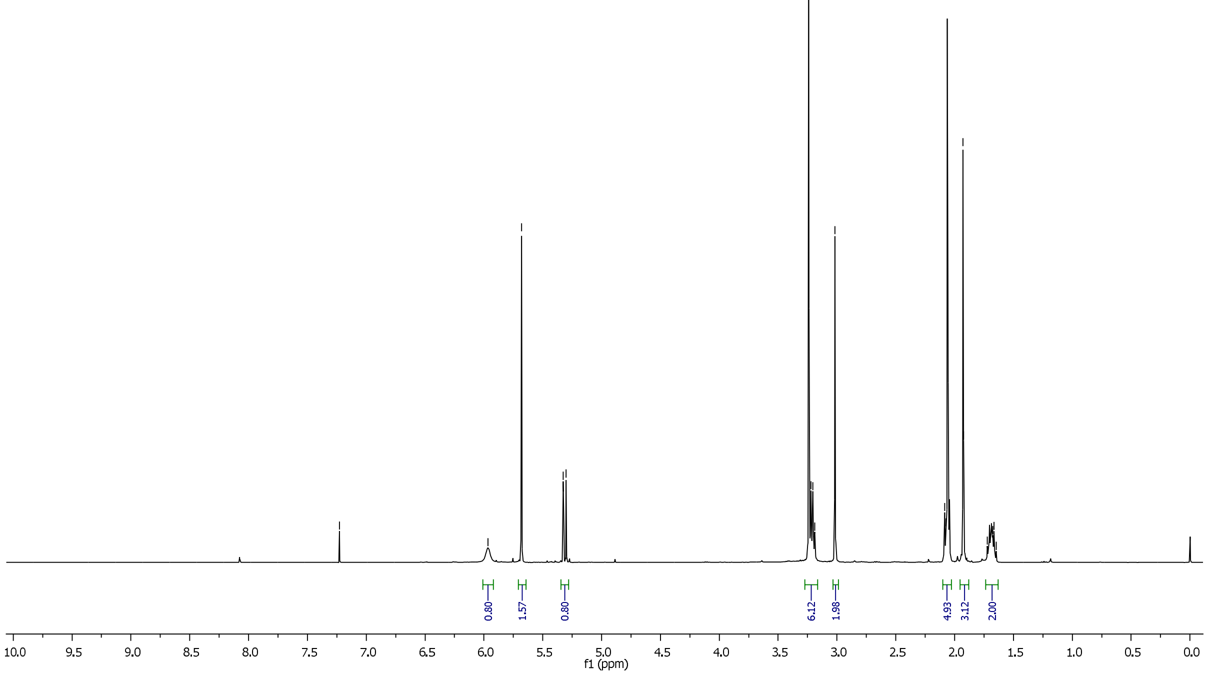


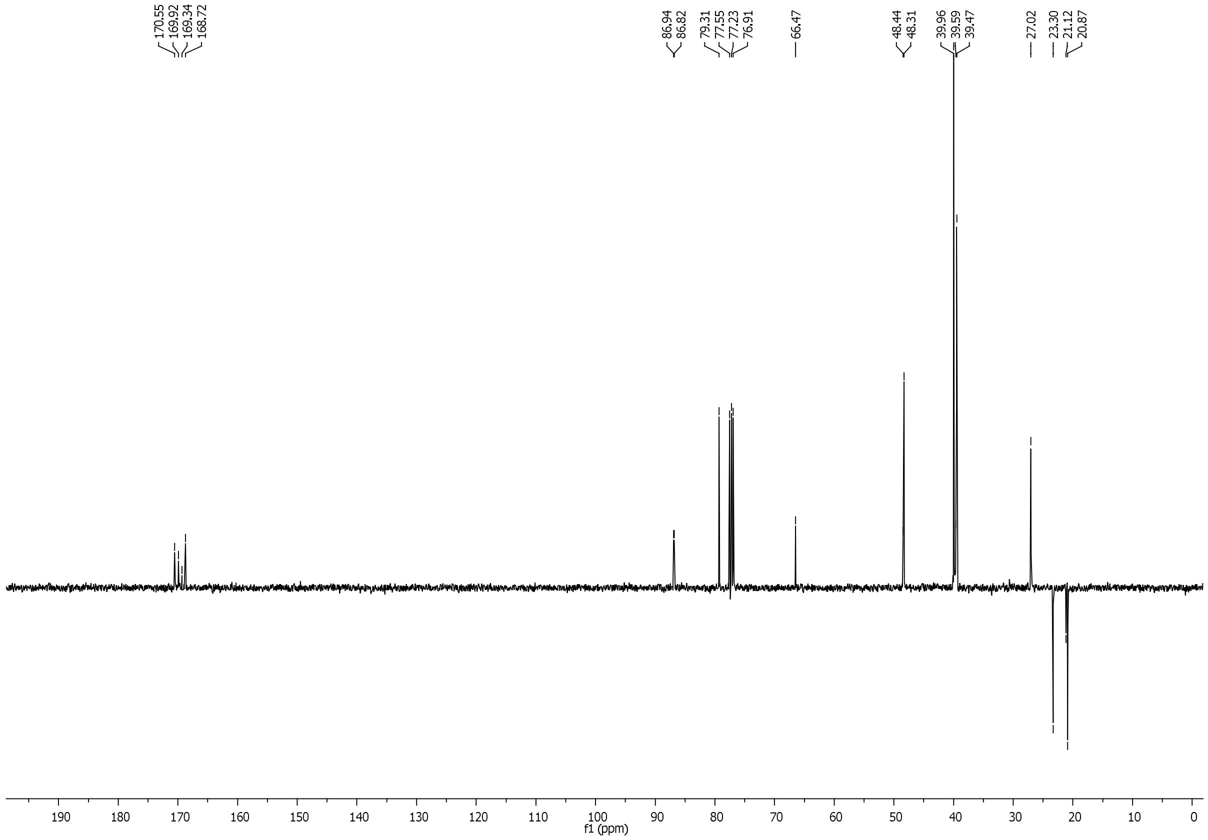

# 3.7. $^{1}$H- and $^{13}$C-NMR of compound 15a (400 MHz, $CDCl_3$)

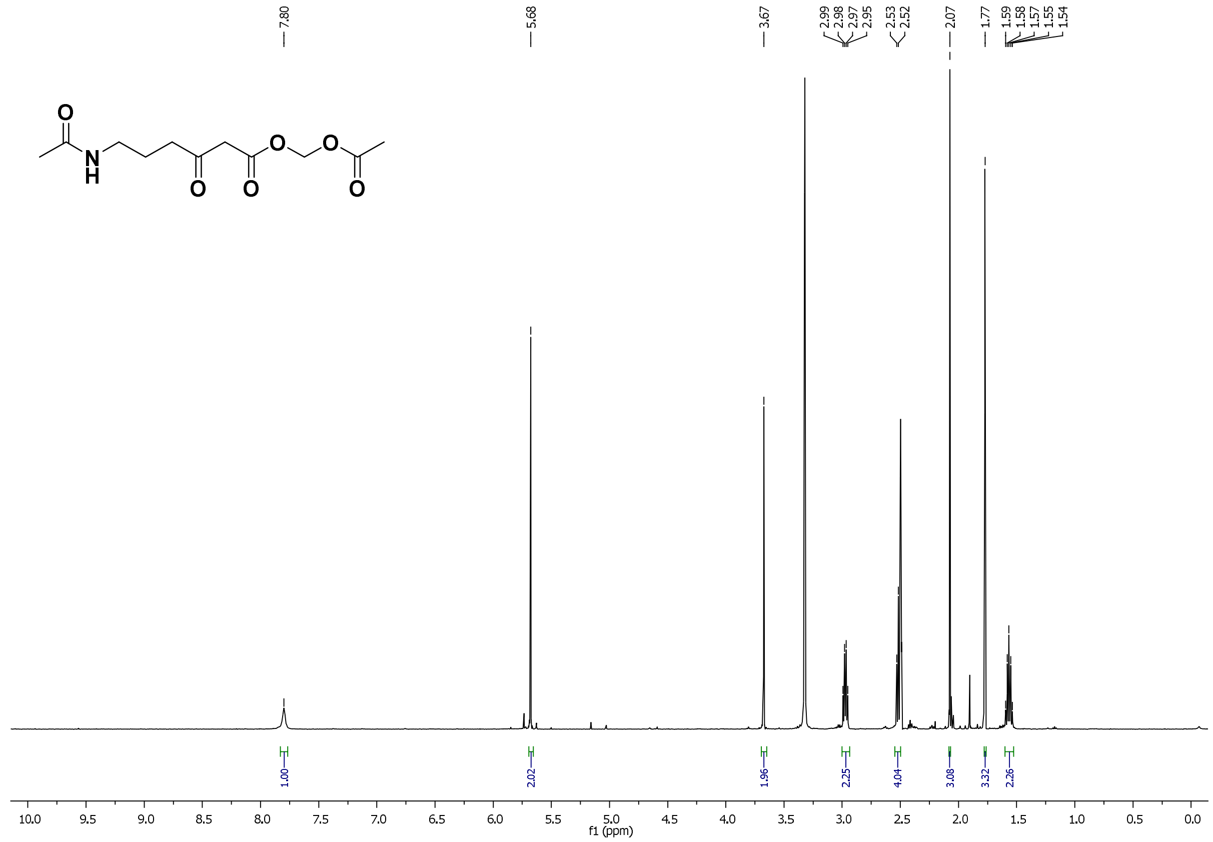


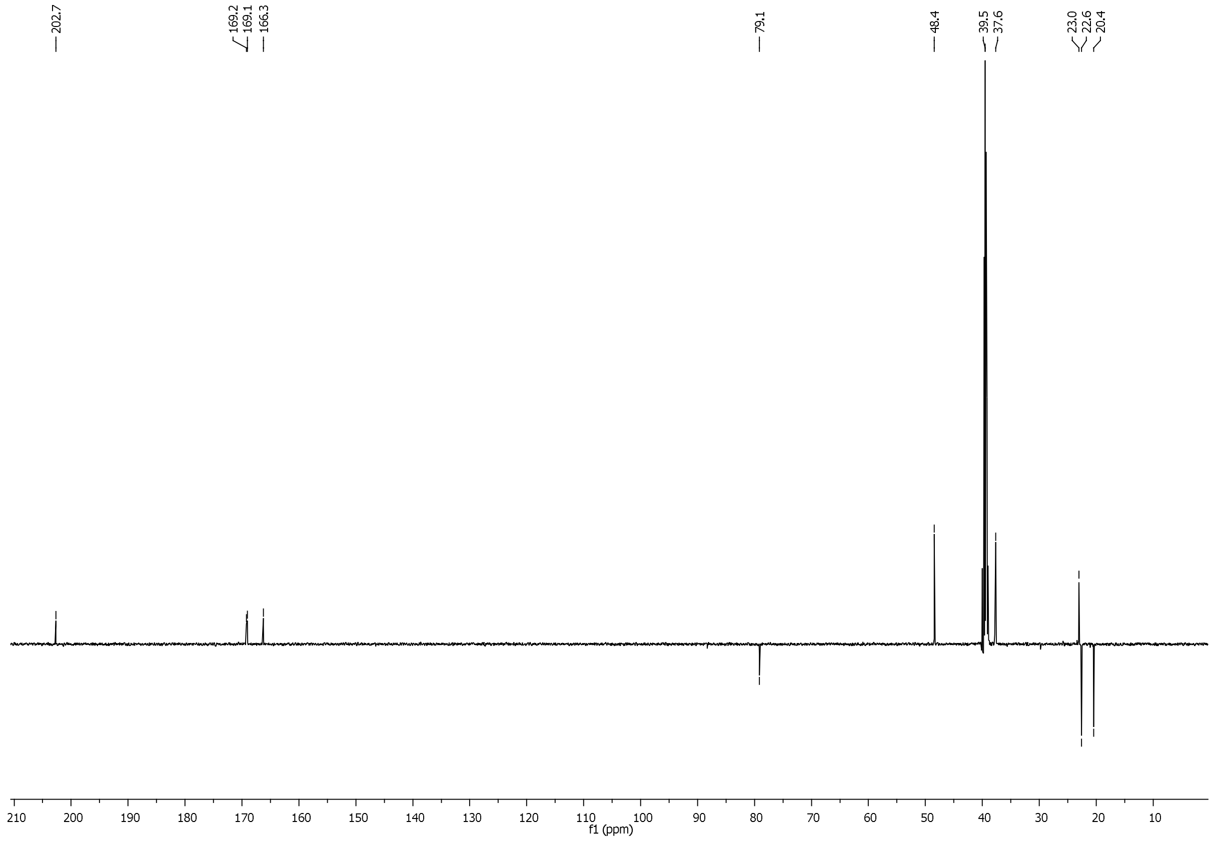

Ina Wilkening,* Silvia Gazzola,* Elena Riva, James S. Parascandolo, Lijiang Song and Manuela Tosin**

# 3.8. $^{1}$H- and $^{13}$C-NMR of compound 15b (400 MHz, $CDCl_3$)

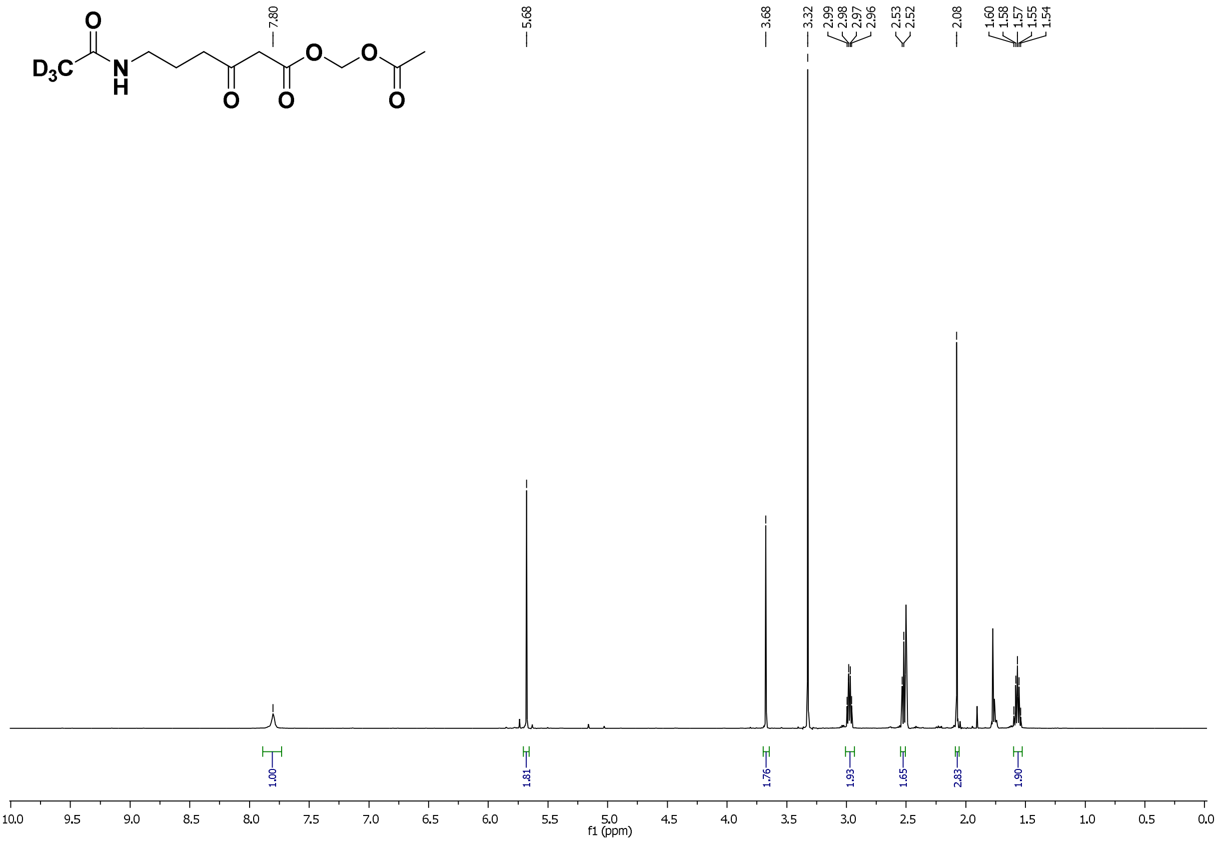


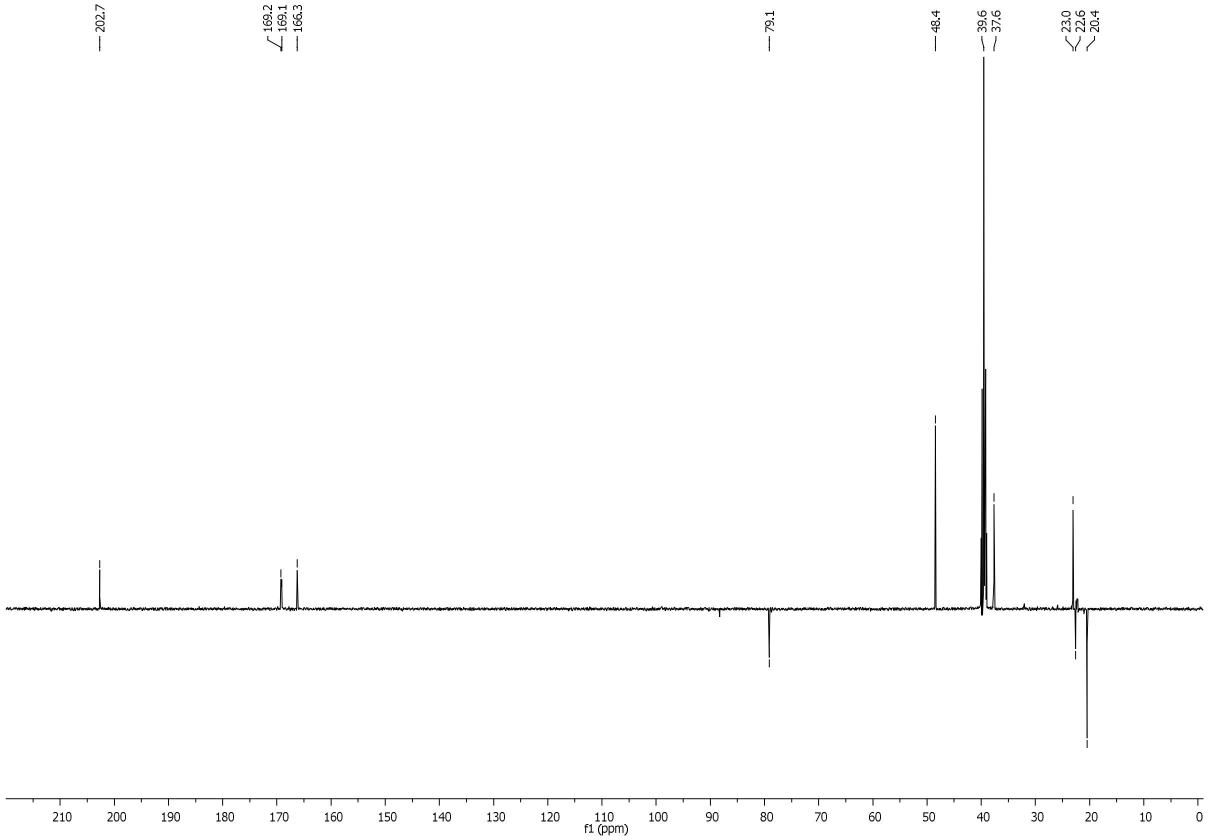

## 3.9. $^{1}$H- and $^{13}$C-NMR of compound 18 (400 MHz, $CDCl_3$)

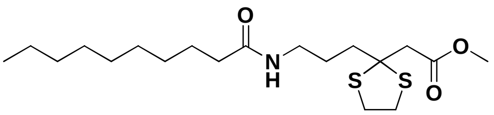


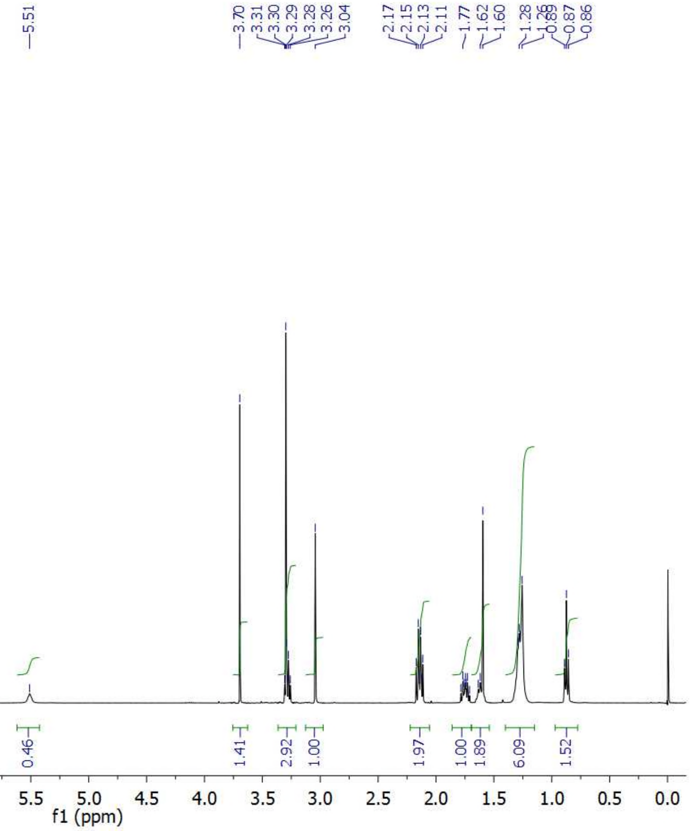


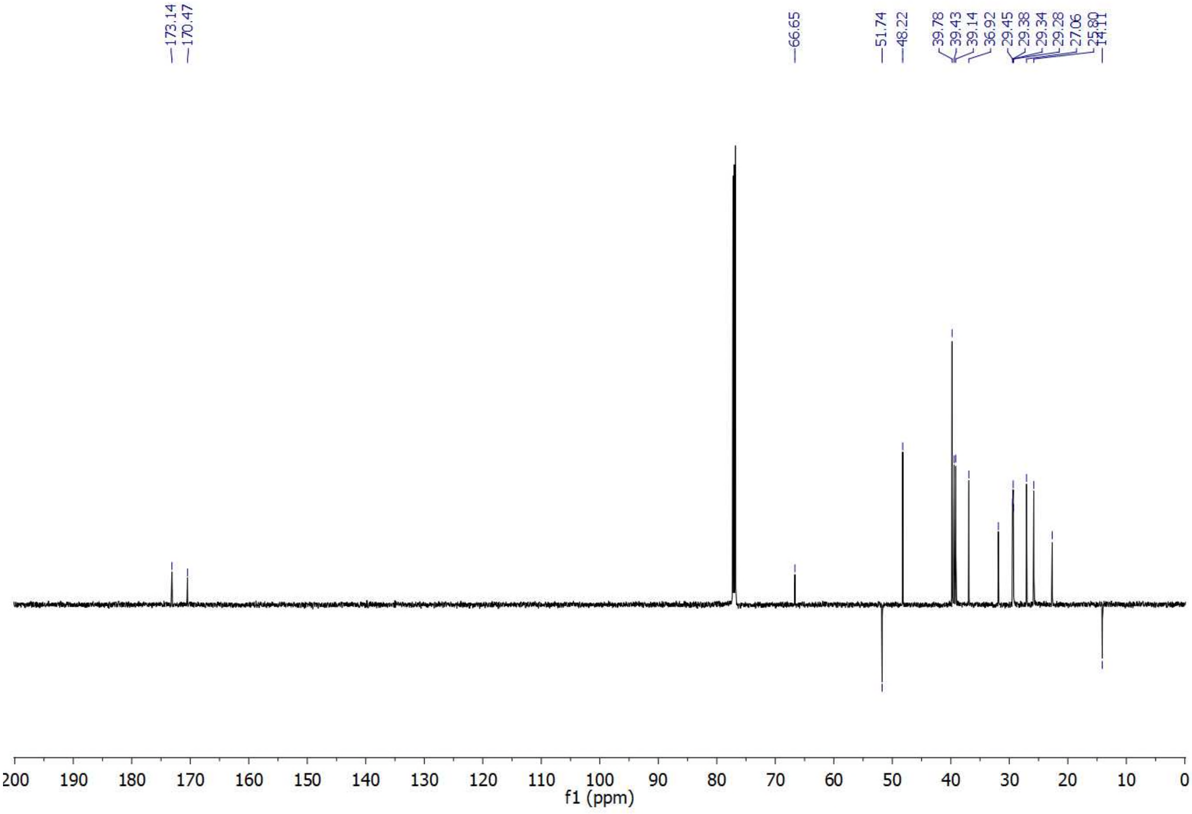

## 3.10. ¹H- and ¹³C-NMR of compound 24 (400 MHz, $D_2O$)

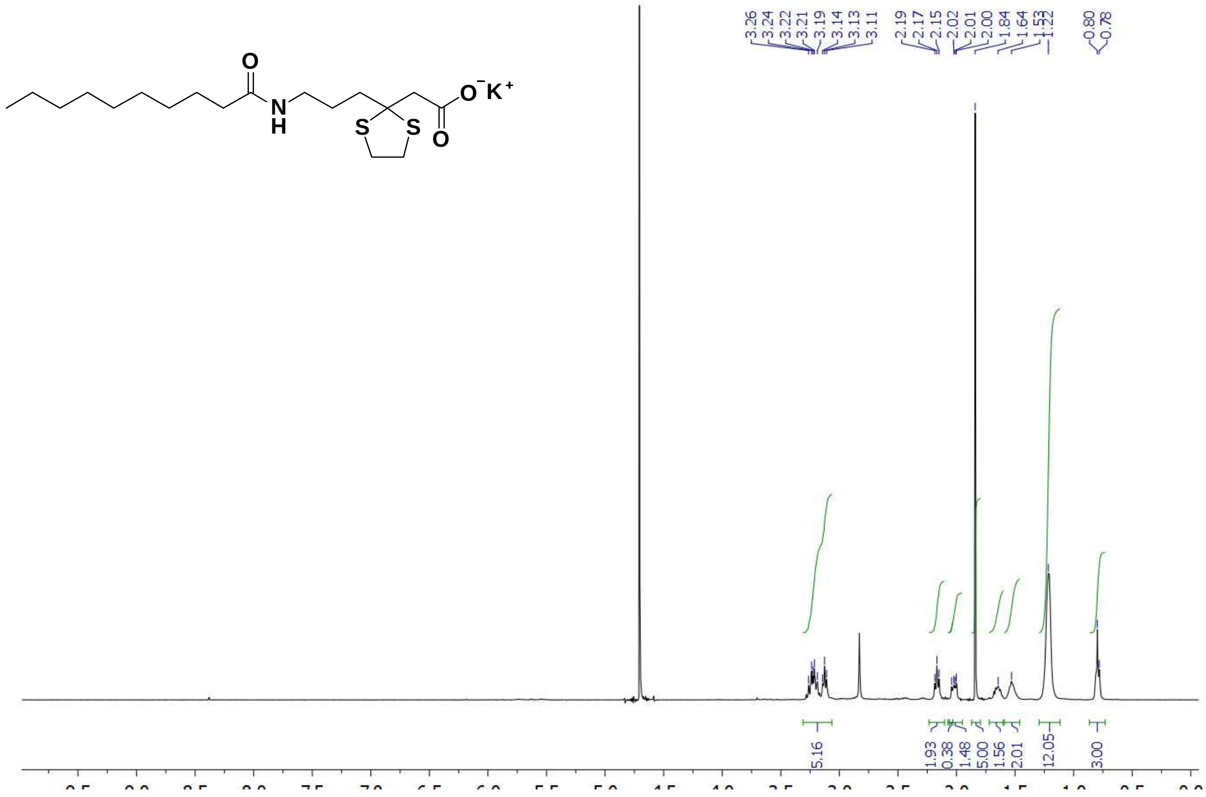


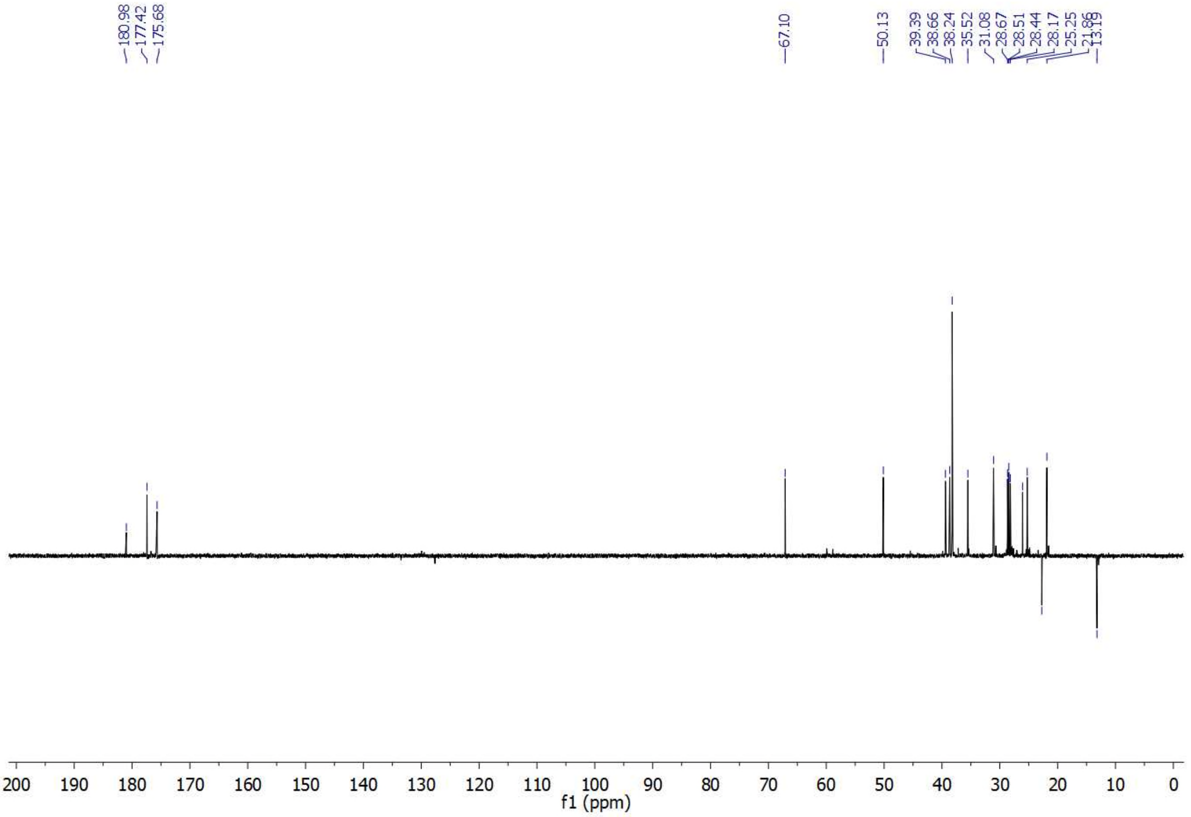

## 3.11. ¹H- and ¹³C-NMR of compound 19 (400 MHz, $CDCl_3$)

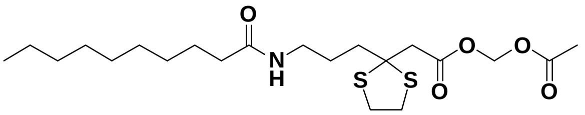


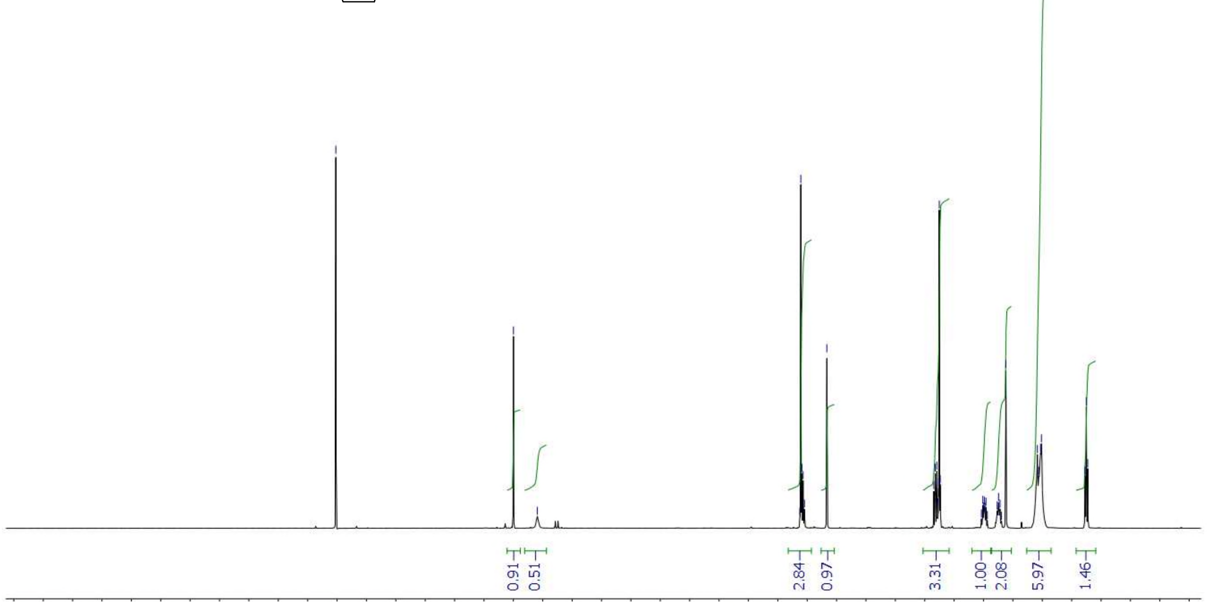


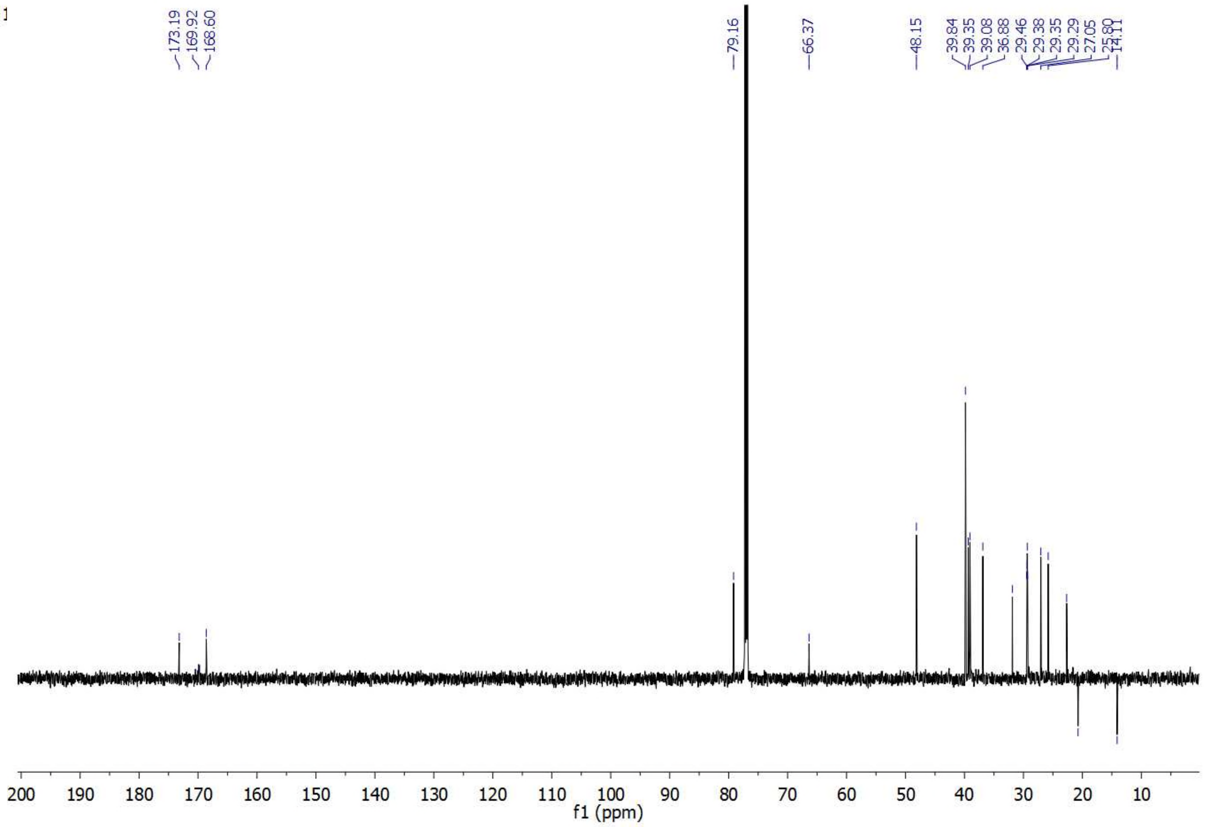

## 3.12. $^{1}$H- and $^{13}$C-NMR of compound 20

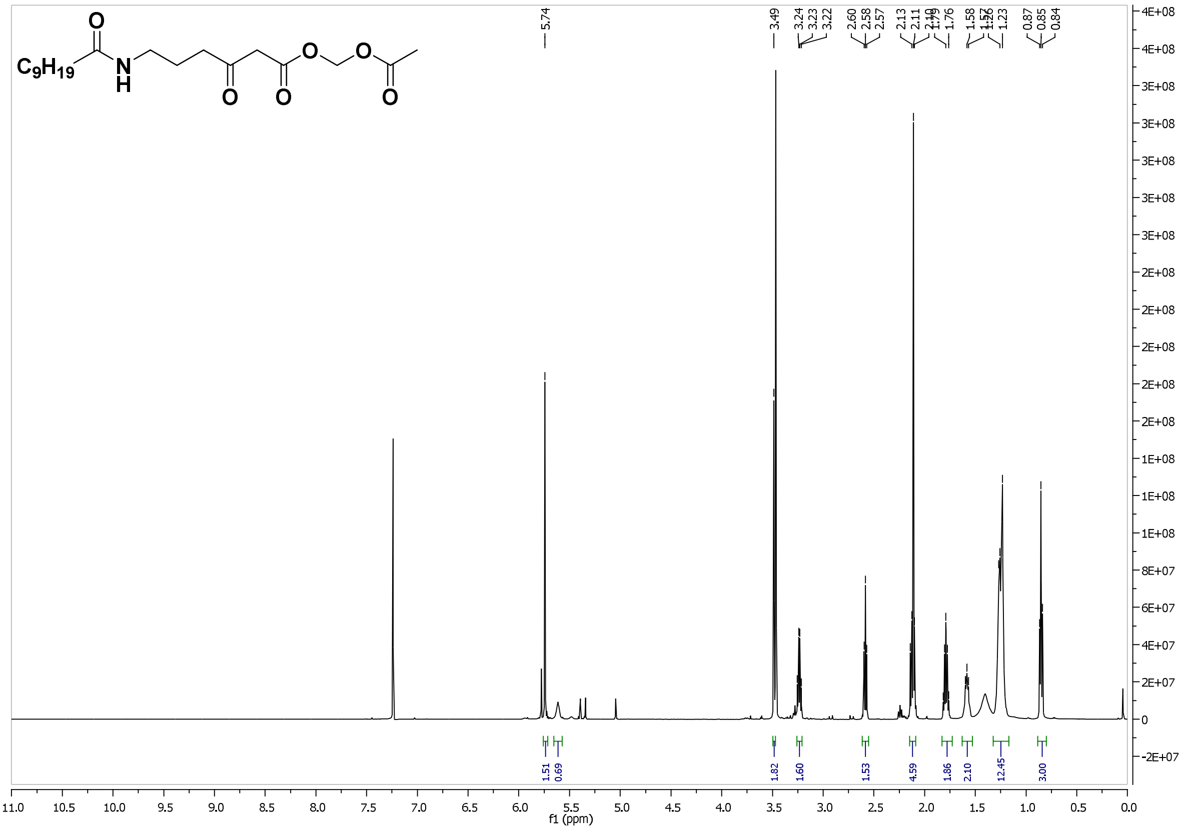


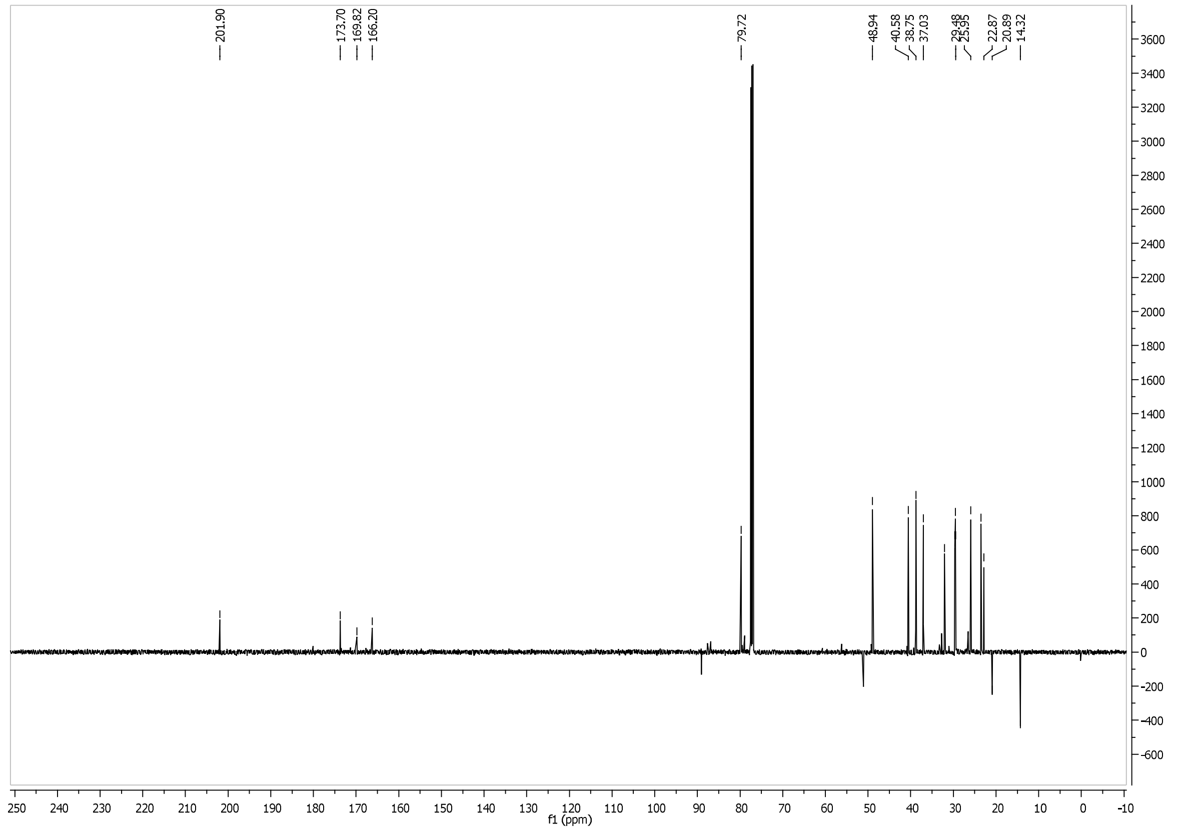

Supplement: Supplementary file 1 [file CC-052-C6CC04681A-s001.pdf]
